# Trogossitidae: A review of the beetle family, with a catalogue and keys

**DOI:** 10.3897/zookeys.366.6172

**Published:** 2013-12-31

**Authors:** Jiří Kolibáč

**Affiliations:** 1 Moravian Museum, Department of Entomology, Hviezdoslavova 29a, 627 00 Brno, Czech Republic

**Keywords:** Coleoptera, Cleroidea, Trogossitidae, key, catalogue

## Abstract

The family Trogossitidae (Coleoptera: Cleroidea) is reviewed to species level. Keys to its genera, tribes and subfamilies are presented for the first time. All known species and subspecies are listed, together with complete taxonomic references back to 1910, the date of issue of the last catalogue of Trogossitidae. Higher taxa reviews are accompanied by remarks on phylogeny, distribution and biology as well as a brief description of adults and larvae. All known fossil records of Trogossitidae are reviewed and discussed. The work includes maps of distribution, colour photographs of generic representatives, morphological illustrations, SEM photographs and phylogenetic trees.

## Introduction

The main purpose of the work is to introduce some modern order into current knowledge of the family Trogossitidae and extend knowledge of this relatively small but fascinating group of beetles, especially to both amateur entomologists and professional “non-cleroid” workers. It is deliberately written as a “compilation” of papers on the topic to date, especially because some of them were published in journals and books that are not easily accessible to all, and to bring various fragmented sources together.

Because of the character and purpose of the work, I have tried to avoid introducing any new thoughts and systematic changes, apart from a few minor ones mentioned in the “New taxonomic acts” section. A catalogue of species lies at the core of the work. I have not repeated references included in *Coleopterorum Catalogus of Temnochilidae* by Léveillé (1910); however, Léveillé’s reference always takes first place in any particular reference list. Taxonomic references follow, just as they have been excerpted from Zoological Records after 1910. The catalogues for some species are, without doubt, incomplete. Some references for biology and local distribution must also, perforce, be lacking – I beg, therefore, the kind reader’s patience and leniency.

The systematics of Trogossitidae is still in its infancy. There remains a great deal of work to be done in the higher taxonomy, as well as with regard to generic limits, especially in widespread, species-rich genera. *Ancyrona* is a good example of such a genus, distributed from tropical Africa, the Palaearctic, south-eastern Asia to Australia. *Tenebroides* and *Temnoscheila* are further complex taxa, each with more than a hundred described species distributed in both North and South America. On the other hand, there also exists a relatively rich modern material of trogossitids to be collected in various parts of the world, certainly containing plenty of new species. Unfortunately, only a few people are seriously interested in the family and only a few of them, in turn, try to gather and publish further information. Therefore, another purpose of the book is to encourage interest in this highly interesting group of beetles.

Keys to higher taxa may be considered a further major element of this contribution. With the exception of those for subfamilies, these have not been published to date. Although it is not always easy to recognize some species-rich and variable trogossitid genera, I have done my best to use simple and easily-visible features in the keys.

## New taxonomic acts

† Meligethiellinae Kireichuk & Ponomarenko, 1990 is resurrected. The subfamily is removed from synonymy with Peltinae: Thymalini and shifted from Cleroidea to Cucujoidea *sensu lato* (including genera † *Meligethiella* Medvedev, 1969 and † *Ostomalynus* Kireichuk & Ponomarenko, 1990; genus † *Juralithinus* Kireichuk & Ponomarenko, 1990 is classified within Trogossitidae: Peltinae *incertae sedis*).

† *Meligethiella* Medvedev, 1969 is removed from Trogossitidae and Cleroidea (species † *Meligethiella glabra* Kireichuk & Ponomarenko, 1990, † *Meligethiella kovalevi* Kireichuk & Ponomarenko, 1990, † *Meligethiella soroniiformis* Medvedev, 1969).

† *Ostomalynus* Kireichuk & Ponomarenko, 1990 is removed from Trogossitidae and Cleroidea (species † *Ostomalynus ovalis* Kireichuk & Ponomarenko, 1990).

† *Peltocoleops* Ponomarenko, 1990 is removed from Trogossitidae and Cleroidea and classified as Coleoptera *incertae sedis* (species † *Peltocoleops onokhojensis* Ponomarenko, 1990).

*Tenebroides bipustulatus* (Fabricius, 1801) (var. *impressifrons* Reitter, 1875 syn. n.).

*Tenebroides bonvouloiri* Léveillé, 1889 (var. *chontalensis* Sharp, 1891 syn. n.).

*Tenebroides maroccanus* Reitter, 1884 (var. *baillioti* Léveillé, 1903 syn. n.).

### Brief review of classification

The superfamily Cleroidea was established by Böving and Craighead (1931). Until that time, Trogossitidae had been classified within Clavicornia together with Nitidulidae (usually as Ostomidae but also as Ostomatidae, Peltidae and Temnochilidae). The names Trogositidae (from Trogositae Fabricius, 1801) and the correct spelling Trogossitidae (from Trogossitarii Latreille, 1802) are the most modern forms of the name (see Kolibáč and Leschen 2010 for details). The family Phloiophilidae is mentioned in Pic’s (1926) catalogue of Melyridae *sensu lato* (the family is sometimes referred to as Phloeophilidae as well). Crowson (1964a) discussed the classification of its only species in detail and classified it within modern Cleroidea. I have suggested a classification of Phloiophilidae as a tribe in Peltinae (Kolibáč 2008) but this has not found wide acceptance.

Reitter (1876) published an excellent world-wide review that is basic to the study of Trogossitidae. Similarly, the world catalogue by Léveillé (1910) is among the classic works. The number of genera and species has only slightly increased since the publication of the latter list, even on a world scale. Crowson (1964a, 1966, 1970) has changed the rank or status of subfamilies and families classified within Trogossitidae *sensu lato* several times. Subsequently, Barron (1971) and later Ślipiński (1992) integrated Crowsons’s families into the single family Trogossitidae. Several years ago, I established tribes in all trogossitid subfamilies on the basis of the morphological characters of adults and larvae (Kolibáč 2006, 2008). The 2008 system is employed throughout this work.

**Table 1.** Major classifications of Trogossitidae.

Reitter 1876	Trogositidae = Helotinae + Trogositinae (Nemozomini incl. present Egoliini; Trogositini; Leperini incl. present Calityini; Peltini incl. present Decamerini, Thymalini, Ancyronini) (*Phloiophilus* not addressed in the paper)
Léveillé 1910	Temnochilidae = Nemosominae (incl. present Egoliini) + Temnochilinae + Leperininae (incl. present Calityini) + Ostominae (incl. present Decamerini, Lophocaterini, Ancyronini) + *Lycoptis* (genus *incertae sedis*)
Reitter 1911	Malacodermata: Cantharidae: Dasytinae: *Phloiophilus*
	Clavicornia: Ostomidae
Crowson 1964	Phloiphilidae (included in Cleroidea by Crowson 1955)
	Peltidae = Decamerinae + Peltinae (incl. present Calityini) + Egoliinae
	Trogossitidae = Lophocaterinae + Trogossitinae
Crowson 1966	Peltidae = Egoliinae + Decamerinae + Peltinae + Rentoniinae (Rentoniini, Protopeltini) (Trogossitidae and *Calitys* not treated in the paper)
Crowson 1970	Phloiophilidae
	Trogossitidae = Trogossitinae + Calitinae + Egoliinae
	Peltidae = Decamerinae + Peltinae + Protopeltinae + Rentoniinae
	Lophocateridae (incl. present Lophocaterini, Ancyronini, *Lycoptis*)
Barron 1971	Trogossitidae = Trogossitinae + Peltinae (incl. present Decamerini, Peltini, Thymalini, Lophocaterinae, Calityini)
Ślipiński 1992 (followed by Lawrence and Newton 1995)	Trogossitidae = Peltinae + Lophocaterinae + Larinotinae (incl. present Colydiopeltini) + Protopeltinae + Decamerinae + Rentoniinae + Calitinae + Egoliinae + Trogossitinae
Kolibáč 2006 (followed by Bouchard et al. 2011, incl. † Lithostomatini)	Trogossitidae = Trogossitinae (Calitiyni, Larinotini, Egoliini, Gymnochilini, Trogossitini) + Peltinae (Peltini, Thymalini incl. Rentoniinae and Protopeltinae, Colydiopeltini, Decamerini, Ancyronini, Lophocaterini) Phloiophilidae not addressed in the paper.
Kolibáč 2008 (used herein)	Trogossitidae = Trogossitinae (Calityini, Larinotini, Egoliini, Gymnochilini, Trogossitini, †Lithostomatini) + Lophocaterinae (Decamerini, Lophocaterini, Ancyronini) + Peltinae (Peltini, Phloiophilini, Colydiopeltini, Thymalini incl. Rentoniinae and Protopeltinae)

### Annotations to the catalogue, keys and illustrations

Léveillé (1910) is always the first reference in catalogues. Reitter (1876), as the most important reference in some taxa, is also listed. The note “synonymized by author” refers to the author of the preceding reference.

Distribution abbreviations: AD = author of description, AL = A. Léveillé, JK = J. Kolibáč, JRB = J. R. Barron, RAC = R. A. Crowson, varA= other authors.

More than seven years have passed since I formulated theses on the higher classification of Trogossitidae. Although some opinions about the phylogeny have changed and the systematic placement of some genera has recently been called into question, the main purpose of the keys is confined to identification of the trogossitid genera. The keys are given for extant subfamilies, tribes and genera. Extinct taxa are listed in relevant sections, together with their descriptions and remarks on their classification. Generic names in parentheses in particular descriptions denote a similar character state occuring in another genus or genera.

The morphological descriptions of particular genera are largely based on several hundred detailed ink-drawings that have already been published by myself (Kolibáč 2005, 2006). PDF files of the papers that include them, as well as all other relevant publications since 2000, are available on request (see author’s address).

All scale bars in plates (Figs 1–12) express one millimetre. Beetles in colour plates (Figs 3–12) are pictured in approximate proportion (“large species” are larger than “small species”). White arrows in SEM photographs (Figs 13–18) denote important characters further mentioned in relevant captions. Numbers in parentheses in maps of distribution (Maps 1–13) denote the number of species within the given genus.

#### Family Trogossitidae

Latreille, 1802

http://species-id.net/wiki/Trogossitidae

Trogossitidae Latreille, P. A. 1802: 110.
Ostomidae Hallan, J. 2007–2012: http://insects.tamu.edu/research/collection/hallan/test/Arthropoda/Insects/Coleoptera/Family/Trogossitidae.txt (check-list). Barron, J. R. 1971: 14. Boosten, G. 1983: 290 (biology). Bouchard, P. et al. 2011: 56 (review of higher taxa). Burakowski, B. et al. 1986: 116. Chûjô, M. & Lee, C. E. 1994: 187 (Korean species). Crowson, R. A. 1955: 82. Crowson, R. A. 1967: 211. Gourves, J. 2006: 56 (biology). Gray, D. W. 2002: 1583 (systematics). Hayes, J. L. J. et al. 2008: 206 (biology). Hieke, F. & Pietrzeniuk, E. 1984: 315 (Baltic amber). Hunt et al. 2007: 1915 (molecular phylogeny). Kireichuk, A. G. & Ponomarenko, A. G. 1990: 79 (Mesozoic fossils). Klimaszewski, J. & Watt, J. C. 1997: 43 (key). Kohnle, U. & Vite, J. P. 1984: 504 (biology). Kolibáč, J. 1993a: 20. Kolibáč, J. 1993b: 89. Kolibáč, J. 2004: (phylogeny). Kolibáč, J. 2005: 39 (morphology of adults). Kolibáč, J. 2006: 117 (morphology of larvae, phylogeny). Kolibáč, J. 2007a: 363 (Palaearctic beetles catalogue). Kolibáč, J. 2009: 127 (nomenclatory). Kolibáč, J. et al. 2005: 25, 129 (Central Europe, key). Kolibáč, J. & Leschen, R. A. B. 2010: 241 (review). Larsson, S. G. 1978: 150 (Baltic amber). Lawrence, J. F. 1982: 519. Lawrence, J. F. et al. 2011: 72 (phylogeny). Lawrence, J. F. & Britton, E. B. 1994: 118. Lawrence, J. F. & Newton, A. F., Jr. 1982: 281 (phylogeny). Lawrence, J. F. & Newton, A. F., Jr. 1995: 867 (review of higher taxa). Lawrence, J. F. et al. 1993: CD ROM (identification of larvae). Lawrence, J. F. et al. 1999a: CD ROM (identification of adults). Lawrence, J. F. et al. 1999b: CD ROM (identification of larvae). Lawrence, J. F. in Stehr F. W. 1991: 448 . Leschen R. A. B. 2002: 263 (review, USA). Léveillé, A. 1910: 1. Lucht, W. 1981: 35. Luna de Carvalho, E. 1979: 80 (key). Majer, K. 1994: 384 (phylogeny, morphology). Merkl, O. 1993: 7 (key). Mitter, H. 1983: 52 (distribution). Nikitsky, N. B. 1980: 43 (key), 92 (larvae). Nikitsky, N. B. 1992: 80 (key). Ponomarenko, A. G. & Kireichuk, A. G. 2004–2008: http://www.zin.ru/animalia/Coleoptera/rus/paleosy2.htm (list of fossils). Paulian, R. 1998: 120 (key). Reitter, E. 1876: 3 (review, key). Reitter, E. 1922 (key). Schmied, H. et al. 2009: 23 (list of fossil taxa). Ślipiński, S. A. 1992: 440 (key to subfamilies, review of classification). Spahr, U. 1981: 74 ( , list of fossils). Stresemann, E. et al. 1989: 270 (key). Tsinkevich, V. A. 1997: 27 (review). Valcarcel, J. P. & Prieto Pilona, F. 2001: 109. Waltz, R. D. 2002: 177. Weidner, H. 1993: 126 (key). Winkler, A. 1927: (Palaearctic beetles catalogue). Zherichin, V. V. 1978: 29 (Mesozoic fossils, Baysa).

##### Morphology

**Figure 1.**
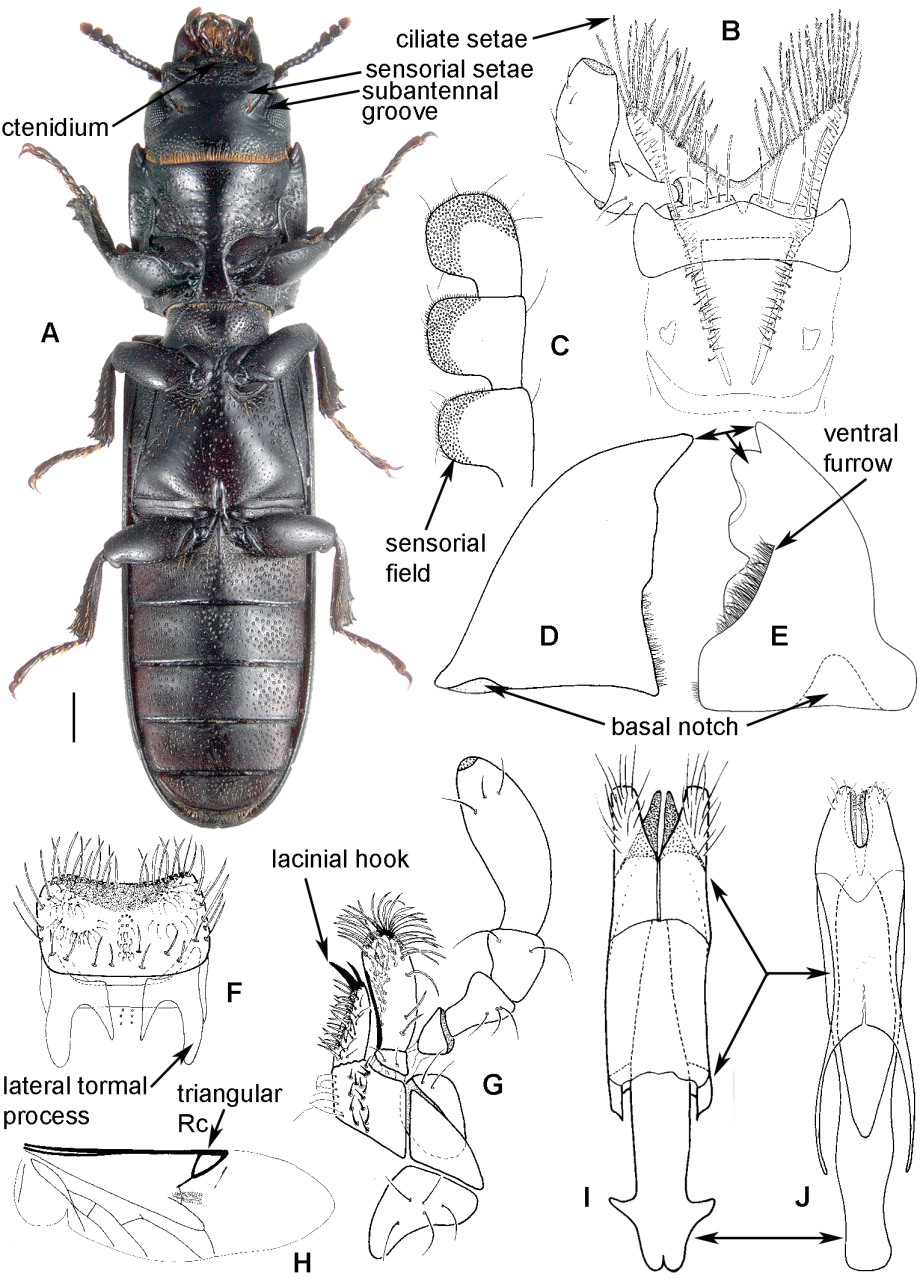
Adult morphology: **A** *Alindria* sp. from Laos, ventral surface **B** *Leipaspis lauricola*, labium **C** *Airora cylindrica*, antennal club **D** *Corticotomus cylindricus*, mandible dorsally **E** *Airora cylindrica*, mandible ventrally **F** *Acalanthis quadrisignata*, labium **G** *Acalanthis quadrisignata*, maxilla **H** *Acalanthis quadrisignata*, wing **I** *Peltonyxa deyrollei*, tegmen composed of 3 parts **J** *Airora cylindrica*, tegmen composed of 2 parts.

**Figure 2.**
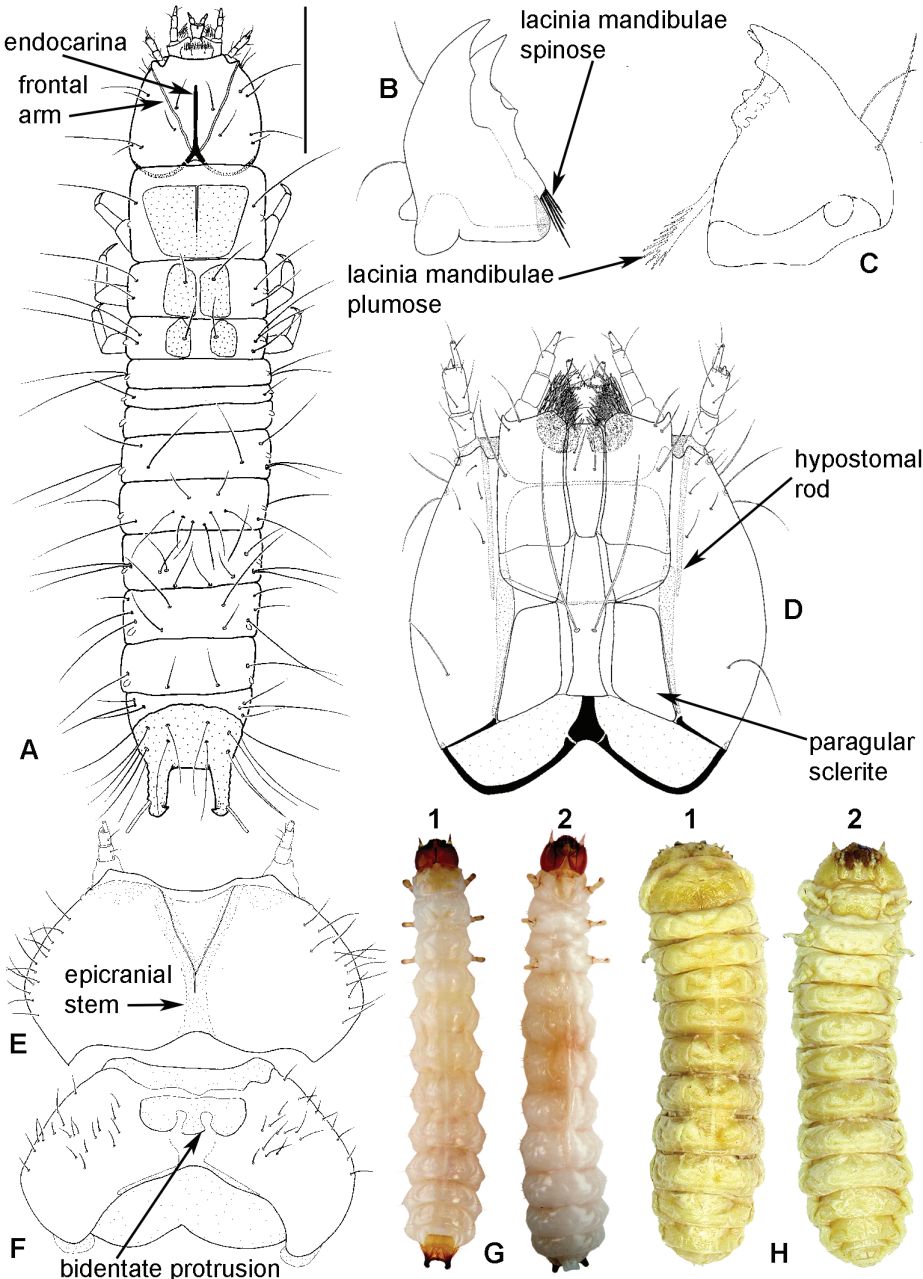
Larval morphology: **A** *Tenebroides* “*fuscus*”, dorsal surface **B** *Tenebroides* “*fuscus*”, mandible ventrally **C** *Lophocateres pusillus*, mandible ventrally **D** *Tenebroides* “*fuscus*”, head ventrally **E–F** *Peltis ferruginea*, head capsule (**E** dorsally **F** ventrally) **G** *Ancyrona diversa* (**1** dorsally **2** ventrally) **H** *Peltis ferruginea* (**1** dorsally **2** ventrally).

(Figs 1, 2). **Adults** (Fig. 1) (according to Kolibáč and Leschen 2010). Body size: 1.0–35.0 mm. Body wide, flattened in most Peltinae; elongate in most Trogossitinae; small and broadly oval in Larinotini and Decamerini; convex in Thymalini. Body mostly bare or sparsely pubescent but sometimes also with tufts of setae, scales, dense pubescence or long hairs. Head usually not declined, although many members of Thymalini are moderately to strongly conglobate (the *Rentonium* group). Anterior margin of frons straight to very deeply emarginate (*Nemozoma*). Gular sutures widely or narrowly separated or strongly convergent. Posterior edge of epicranium with two incisions or evenly rounded. Frontoclypeal suture present or absent. Antennal insertions partly covered by edge of frons or visible in dorsal view. Subantennal groove conspicuous (receiving from one to three antennomeres), reduced or absent. Eyes not emarginate or (rarely) posteriorly emarginate, flat to elevated; sometimes divided by canthus into dorsal and ventral eyes (as in Gyrinidae and some Cerambycidae). Labrum broadly oval to oblong, mostly slightly emarginate; epipharynx consisting mainly of a cordate sclerite; tormae connected, or not, at centre by tormal bridge. Antennae 8- to 11-segmented, with conspicuous 1- to 3-segmented club or with widened distal antennomeres that may be asymmetrical. Three to five apical antennomeres often bear sensorial fields. Mandible with one or two, rarely three, apical teeth; usually with ventral ciliate furrow; prostheca well-developed (brush of setae), reduced to absent; mola present, reduced or absent. Maxilla with distinct galea and lacinia; galea sometimes with ciliate or denticulate setae; lacinia with one to three apical hooks or with spine-like setae or with dense soft pubescence; basistipes more or less coalescent with lacinia or free; palpifer denticulate along outer margin (in Trogossitinae); apical palpomere conical or cylindrical. Male submentum with tuft of setae in some Trogossitinae; ligula membranous, rigid or coalescent with prementum; apex of ligula deeply or shallowly emarginate, often with ciliate setae; palpi conical or cylindrical, rarely weakly securiform. Tentorial arms connected by bridge or bridge reduced. Cervical sclerites present. Pronotum usually transverse; elongate only in some Trogossitinae. Lateral carinae almost always present, often denticulate; reduced only in some Trogossitinae (*Corticotomus*). Prosternal process apically dilated or narrowed. Procoxa slightly to strongly transverse. Coxal cavities internally open and externally closed or widely open. Notosternal suture complete. Trochantin elongate, exposed. Mesonotum distinct, with transverse scutum and well-developed scutellar shield. Elytra usually regularly punctate, with or without conspicuous carinae; epipleura well-developed or reduced in posterior half; elytra of Trogossitinae with interlocking mechanism along apical part of suture. Mesoventrite wide, with distinct prepectus. Mesocoxae usually projecting. Mesanepisternum triangular, not extending to mesocoxal cavity. Mesepimeron triangular, usually reaching coxal cavities, so that these are laterally open (cavities closed by meeting of mesoventrite and metaventrite in Egoliini). Metaventrite more or less flattened, with distinct discrimen and transverse katepisternal suture; subcoxal lines present in *Colydiopeltis*, *Larinotus*, and *Thymalus*. Metanepisternum longitudinal, often with carina at centre. Metacoxae extending laterally to meet elytral epipleura, often with longitudinal furrow at centre. Metendosternite with conspicuous lateral arms. Wings usually present, but missing in some species. Apical field sometimes with one or more small sclerites just beyond radial cell; RP2 sometimes present. Radial cell as long as wide or shorter than wide, sometimes very reduced or absent; cross-vein r3 usually absent. Basal portion of RP short or sometimes absent. Medial field with as many as four free veins, a wedge cell and no medial fleck (fewer veins and no wedge cell in smaller species); anal embayment usually notch-like, absent in some Trogossitini. Trochanters triangular. Femora sometimes clavate. Tibiae often with row of spines along outer side; apex of tibia with row of spines and two hooked spurs or only one spur hooked or spurs reduced (spines reduced in smaller taxa); tibial spurs pattern varies from 2-2-2 to 0-0-0. Tarsi 5-5-5; tarsomere 1 sometimes partially fused with 2 but always with conspicuous suture between them or very small and tarsal pattern seemingly 4-4-4 or 4-4-5; tarsomeres 1–4 never with membranous lobes; apical tarsomere usually as long as combined length of tarsomeres 1–4; claws large, without denticles (with the exception of some Decamerinae); empodium bisetose, strongly projecting. Abdomen with five or six ventrites. Ventrites I–III fused. Intercoxal process small, narrow. Spiculum of ventrite VIII sometimes present in males and always in females. Segment IX well-developed or reduced to “spicular fork”. Aedeagus sheath-like cucujiform type, with fixed or articulated parameres that may be partly or entirely fused together or absent. Tegmen usually with anterior ventral strut and two opposing dorsal struts (“double tegmen” of Crowson 1964a), but usually inverted (rarely uninverted or placed laterally) and often composed of two or three parts (undivided in some members of the *Rentonium* group). Penis with two anterior struts. Ovipositor lightly sclerotized, except for baculi, moderate in size with sparsely pubescent coxites and styli. Bursa copulatrix large, spherical. Spermatheca elongate or oval, with gland. Vagina without sclerites. Six malpighian tubules present in *Tenebroides* and *Lophocateres*.

**Larvae** (Fig. 2) (according to Kolibáč and Leschen 2010). Five to seven larval instars observed in Trogossitinae (*Temnoscheila*, *Tenebroides*), four instars in Lophocaterinae (*Lophocateres*), all beetles reared in laboratory conditions. Body elongate, only weakly flattened. Colour white or pale, but sclerotised areas distinctly pigmented (head capsule, thoracic and abdominal terga, and urogomphi). Vestiture consisting of setae; rarely with bristles or expanded setae; sometimes body with short and sparse pubescence or only with setae on the last segment. Head protracted. Posterior edge of capsule slightly emarginate. Epicranial stem absent or present and of variable length. Median endocarina usually present, of variable length and usually extending between frontal arms (absent in *Thymalus* and/or coincident with epicranial stem and frontal arms in Peltinae); paired endocarinae present or absent. Frontal arms V-shaped, straight or curved (nearly S-shaped). Five stemmata usually present and arranged in a pattern with two anteriorly and three in a posterior row; sometimes reduced to four, three, two or none. Frontoclypeal suture usually absent (distinct in the *Rentonium* group and *Thymalus*). Labrum free; epipharynx membranous; shape of tormae variable and lacking posterior extensions. Antennae 3-segmented, with short sensorium present at apex of segment 2. Mandibles with one or two apical teeth (serrate in the *Rentonium* group); mola absent or present; mesal edge of mandibular base with brush of hairs or rigid denticulate processes that may be hylaline. Ventral mouthparts retracted. Maxillary articulating area present or absent. Cardo typically undivided (divided in *Calitys*). Mala with apex usually simple, with large pedunculate seta in predatory species (Trogossitini and Egoliini); inner apical angle usually lacking small teeth (present in *Protopeltus* and Larinotini); palps with four, three (e.g., *Ancyrona* and *Lophocateres*), or two (*Rentonium* group) palpomeres. Labium consisting of prementum, mentum, and submentum, or pre- and postmentum (*Thymalus* and *Parapeltis*); mentum or postmentum free or connate with base of maxillae; prementum sclerotized and elongate; mentum mostly unsclerotized in some taxa; ligula absent or present; if present apex emarginate or not, or divided apically; palps usually 2-segmented (1-segmented in the *Rentonium* group). Gular region longer than wide, or wider than long; fused to labium or not. Hypostomal rods present, reduced or absent; sometimes extending to posterior edge of head; subparallel or diverging posteriorly. Ventral epicranial ridges present or absent. Prothorax usually with one large sclerite dorsally and one elongate sclerite ventrally. Protergum with or without sclerotized plate with a longitudinal median ecdysial line. Meso- and metathorax usually with pair of sclerites dorsally (absent in some taxa) and one weakly sclerotised, pale plate ventrally. Sometimes all thoracic sclerites indistinct. Coxae widely separated. Thoracic legs 5-segmented, including claw-like pretarsus with single seta. Nine abdominal segments visible from above. Abdominal ampullae present or absent. Segment IX shorter than or subequal to VIII. Segment X almost always concealed by segment IX (visible from above only in *Larinotus*). Urogomphi usually well-developed (sometimes reduced) and dorsally or posteriorly oriented; large, hook-shaped or nearly straight; strongly sclerotized and pigmented; often with spines or secondary processes; apically bifurcate or not; pit present between urogomphi in *Parapeltis*; median process present between urogomphi in Lophocaterini and urogomphi located at apex of median process in some members of the *Rentonium* group. Anal region posteriorly or posterioventrally oriented; paired pygopods on segment X absent. Six malpighian tubules in *Tenebroides*; four in *Lophocateres*.

##### Key to subfamilies.

Identification of the trogossitid subfamilies using the various determination keys published by a range of authors tends to be a complicated and frustrating process. Unfortunately, my “lumping” of nine former subfamilies (e.g., Ślipiński 1992) in two (Kolibáč 2006) rather complicated the identification of the individual specimen. In the traditional system for the trogossitids used in the 19th century (e.g. Erichson, Reitter, Léveillé), Peltinae were flat and fungivorous whereas Trogossitinae were cylindrical and predatory. Further study of such modified taxa as the rentoniins, decamerins or colydiopeltins revealed huge morphological and biological diversity within Peltinae (*sensu* Kolibáč 2006). The same situation holds in Trogossitinae, in which superficially different taxa (such as *Calitys*, *Larinotus*, the gymnochilins and egoliins) are classified together in one subfamily. The subfamily Lophocaterinae was established by Crowson (1964a) and synonymized with Peltinae by Kolibáč (2006) because of possible paraphyly of the latter subfamily. Later, in response to new observations, we suggested (Kolibáč 2008, Kolibáč and Zaitsev 2010) that Peltinae be split once more into Lophocaterinae and Peltinae. The latter is the system used in this book.

Similar ways of life (members of the both subfamilies tend to be predatory), reductions of morphological structures common to the whole order Coleoptera (e.g. wing venation, lateral edge of pronotum, mola), mosaic character patterns and probably some underlying synapomorphies complicate the definition of subfamilies even other higher taxa in Trogossitidae, in much the same way as they do in the related family Cleridae. The key that follows is therefore not based on absolutely inclusive synapomorphies. The most important, clearly-visible characters appear in **bold type**.

**Table:** 

1	Adult: labium with rigid ligula; epipharynx mostly with cordate sclerite along apex of labrum; **antennal club mostly conspicuously asymmetrical**, terminal antennomeres (antennal club) mostly with sensorial fields; **front coxal cavities externally closed**; body cylindrical or oval but not conglobate; end of elytral suture with distinct interlocking mechanism (“elytral lock”). Larva: **head capsule with distinct endocarina**, gular sutures and hypostomal rods; frontal arms mostly straight; **gular region mostly with paragular sclerites**. Mainly predatory, rarely fungivorous or phytophagous (e.g. feeding on grains)	Trogossitinae
–	Adult: labium with membranous ligula; epipharynx without cordate sclerite; **antennal club weakly asymmetrical or symmetrical**, terminal antennomeres (antennal club) without distinct sensorial fields; **front coxal cavities mostly externally open** (except for Lophocaterinae: Decamerini); body often oval and flat (but sometimes also convex or conglobate); elytral suture without distinct interlocking mechanism. Larva: **head capsule mostly without endocarina**; gular sutures and hypostomal rods reduced; frontal arms often curved; **gular region without paragular sclerites**	2
2	Adult: **frontoclypeal suture absent or inconspicuous**; gular sutures wide, subparallel; eyes moderate, not distinctly elevate; antennal club symmetrical; radial cell oblong, moderate; tibial spines along sides mostly reduced; body flat, convex or conglobate. Larva: **lacinia mandibulae tridentate, absent or minute**. Fungivorous or phytophagous	Peltinae
–	Adult: **frontoclypeal suture present, sometimes distinctly emarginate** (or concave); gular sutures wide, convergent at apex; eyes almost elevate, laterally situated; antennal club weakly asymmetrical; radial cell moved downwards, towards wing centre, sometimes small or reduced; tibial spines along sides present; body always flat. Larva: **lacinia mandibulae plumose, always distinct**. Primitive members fungivorous or phytophagous, advanced ones floricolous or predatory	Lophocaterinae

#### Subfamily Trogossitinae Latreille, 1802

Latreille, P. A. 1802: 110.

See “Family Trogossitidae” section for further references.

##### A key to the extant tribes of Trogossitinae

**Table:** 

1	Elytral interlocking mechanism absent or weak; antennal club loose, symmetrical; dorsal surface flat, with tufts of setae and tubercles. Fungivorous	Calityini
–	Elytral interlocking mechanism present; antennal club asymmetrical (or compact and symmetrical); dorsal surface convex, with scales or regularly pubescent or bare. Predatory, rarely phytophagous	2
2	Middle coxal cavities closed; dorsal surface mostly with very long hairs	3
–	Middle coxal cavities open; dorsal surface bare, with sparse pubescence or with scales	4
3	Antennal club compact and symmetrical; tibiae with reduced apical spurs; gular sutures wide, convergent at apex. Larva without paragular sclerites	Larinotini
–	Antennal club asymmetrical; tibiae with conspicuous apical spurs; gular sutures narrow, subparallel at apex. Known larvae with paragular sclerites	Egoliini
4	Eyes more/less dorsally situated, some genera with 2 pairs of eyes; body surface distinctly regularly sculptured or covered with scales or with short, thick setae; elytra with distinct carinae; anterior margin of pronotum always deeply emarginate	Gymnochilini
–	Eyes laterally situated, rather flat, always only single pair of eyes present; body surface finely punctate or wrinkled, without scales or thick setae; elytra without carinae or with weak carinae; anterior margin of pronotum emarginate or not	Trogossitini

##### Tribe Calityini

Reitter, 1922

Calityini Reitter, E. 1922: 66.

###### Type genus.

*Calitys* Thomson, 1859

Bouchard, P. et al. 2011: 57. Crowson, R. A. 1970: 13 (referred as Calitinae subfam.nov.). Ślipiński, S. A. 1992: 442 (Calitinae). Lawrence, J. F. & Newton, A. F., Jr. 1995: 869 (Calitinae). Kolibáč, J. 2006: 117 (Calityni Winkler, 1922; *sic*!) (diagnosis, new status). Kolibáč, J. 2007a: 364. Kolibáč, J. & Leschen, R. A. B. 2010: 242

###### Remarks.

The position of the single genus *Calitys* within the trogossitid system has changed many times over the past century or so. It has been classified within either Peltinae or Trogossitinae (compare, for example, Barron 1971 and Crowson 1964a *vs*. Crowson 1970), then treated as a separate subfamily (Crowson 1970 in Trogossitidae *s.str.* without Peltidae, Ślipiński 1992 in Trogossitidae *s.lat.*). Reitter’s early idea (1876) that it might be classified within the former Leperini or Leperinae (i.e. Gymnochilini herein) is also interesting and worthy of review. *Calitys* belongs among the primitive fungivorous groups and has several features shared with Peltinae, for example: robust mandibles with mola, flat body, wide pronotum, weakly asymmetrical antennal club, and absence of elytral interlocking mechanism. However, it also has bizarre sculptures on dorsal surface of body, wax scales, and tufts of rigid setae that together differentiate the genus from all other trogossitids. Its basal position in the trogossitine branch is based chiefly on procoxal cavities perfectly externally closed, presence of paragular sclerites in larval cranium and concave larval tergite IX. Wax scales and tufts of setae on head, antennae, elytra, and pronotum make it resemble Gymnochilini. Nonetheless, it remains possible to imagine *Calitys* also as a derived member of Peltinae or even Lophocaterinae.

##### Genus Calitys

Thomson, 1859

http://species-id.net/wiki/Calitys

Figs 3 , 13 ; Map 1

Calitys Thomson, C. G. 1859: 71.

###### Type species.

*Hispa scabra* Thunberg, 1784 [by original designation and monotypy]

Barron, J. R. 1971: 18. Crowson, R. A. 1964a: 296. Kolibáč, J. 2005: 49 (redescription). Kolibáč, J. 2006: 111 (phylogeny). Kolibáč, J. 2007a: 364. Kolibáč, J. 2008: 118–119 (phylogeny). Kolibáč, J. et al. 2005: 129 (key). Léveillé, A. 1910: 24. Reitter, E. 1911: 6, 7 (*Calytis* Thomson, 1859: misspelling; see Barron, J. R. 1971: 19). Reitter, E. 1922: 66. Spahr, U. 1981: 74 (amber and copal fossils)

*Nosodes* LeConte, 1861 [type species: no species included; see Barron 1971]

Barron, J. R. 1971: 19. Reitter, E. 1876: 43

*Peltidea* Motchulsky, 1858 [type species: *Peltidea dentata* Fabricius, 1787]

Barron, J. R. 1971: 18. (*nomen oblitum*)

###### Description

(*Calitys scabra*). Body size: about 10.0 mm. Adult: Body shape flat. Gular sutures wide, convergent at apex. Frontoclypeal suture present. Frons: longitudinal groove or depression absent. Cranium ventrally: tufts of long setae at sides absent. Submentum: ctenidium absent. Antennal groove present. Eyes: size moderate. Eyes number: two. Epicranial acumination deep. Lacinial hooks: two. Galea: shape sub-clavate. Galea: ciliate setae absent. Mediostipes-Lacinia partially fused. Palpifer: outer edge even. Mandibular apical teeth number: two, horizontally situated. Mola present. Penicillus (at base) present (fine, often membranous). Pubescence above mola or cutting edge absent. Ventral furrow present. Basal notch shallow or absent. Labrum-Cranium not fused. Epipharyngial sclerite absent. Lateral tormal process: projection curved downwards, processes with bridge (*Peltis*). Ligula: ciliate setae absent. Ligula rigid, strongly retroflexed, weakly emarginate. Hypopharyngeal sclerite H-shaped. Antenna 11-segmented. Antennal club symmetrical, sensorial fields absent. Front coxal cavities externally closed, internally open. Pronotum transverse. Prepectus present. Middle coxal cavities open. Elytra: long hairs absent. Epipleuron wide. Elytral interlocking mechanism absent, carinae conspicuous. Elytral punctation regular, scales present. Wing: radial cell oblong (or reduced), wedge cell small (*Peltis*), cross vein MP3-4 present, cross vein AA1+2-3+4 absent. Front tibiae: spines along side moderate. Hooked spur present. Claws: denticle absent. Parasternites number along ventrites III–VII: two. Spiculum gastrale present. Tegmen composed of three parts.

Larva: Frontal arms weakly curved. Epicranial stem absent. Endocarina present. Gular sutures conspicuous, parallel. Gula: anterior apodemes absent. Paragular sclerites present. Hypostomal rods absent. Stemmata number: five. Mandibular apical teeth number: two, horizontally situated. Lacinia mandibulae absent. Mola reduced. Maxillary palpi 3-segmented. Cardo-Stipes not fused. Cardo: size much smaller than stipes. Ligula present. Labial palpi 2-segmented. Antennal joints 1 and 2 elongate. Sensory appendix very small. Thoracic sclerites pattern (dorsally) 1-2-2. Thoracic sclerites pattern (ventrally) 0+0+0. Trochanter oblong. Abdominal segment IX not divided. Tergite IX flat. Urogomphi present, hooked; median process absent.

**Figure 3.**
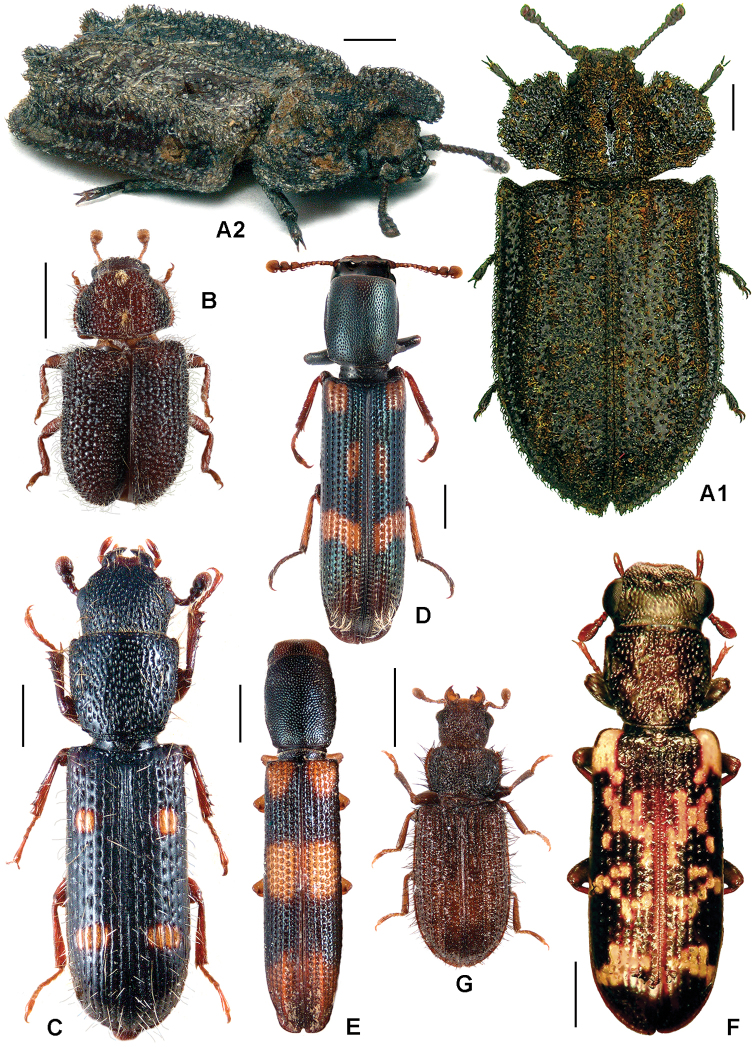
**A** *Calitys scabra* **B** *Larinotus umblicatus* **C** *Acalanthis quadrisignata* **D** *Calanthosoma flavomaculata* **E** *Calanthosoma* (syn. *Marnia*) *sipolisi* **F** *Egolia variegata* **G** *Necrobiopsis tasmanicus*.

###### Biology.

Fungivorous. Live under bark of old coniferous trees (fir, pine) and on tree fungi. *Calitys scabra* was observed together with its larvae, for example, in the old stump of a fir *Abies alba* in Slovakia (J. Vávra, pers. observ.). Brustel (2009) recorded it from *Antrodia* sp. polypore fungi in Pyrenées Mts.

###### Distribution.

Two species Holarctic. Two more species also reported from South Africa, of which *Calitys spinifera* is unknown to me. I studied a single *Calitys africana* non-type specimen in the Musée d’Histoire Naturelle in Geneva in 2003. It does not belong to Cleroidea.

**Map 1.**
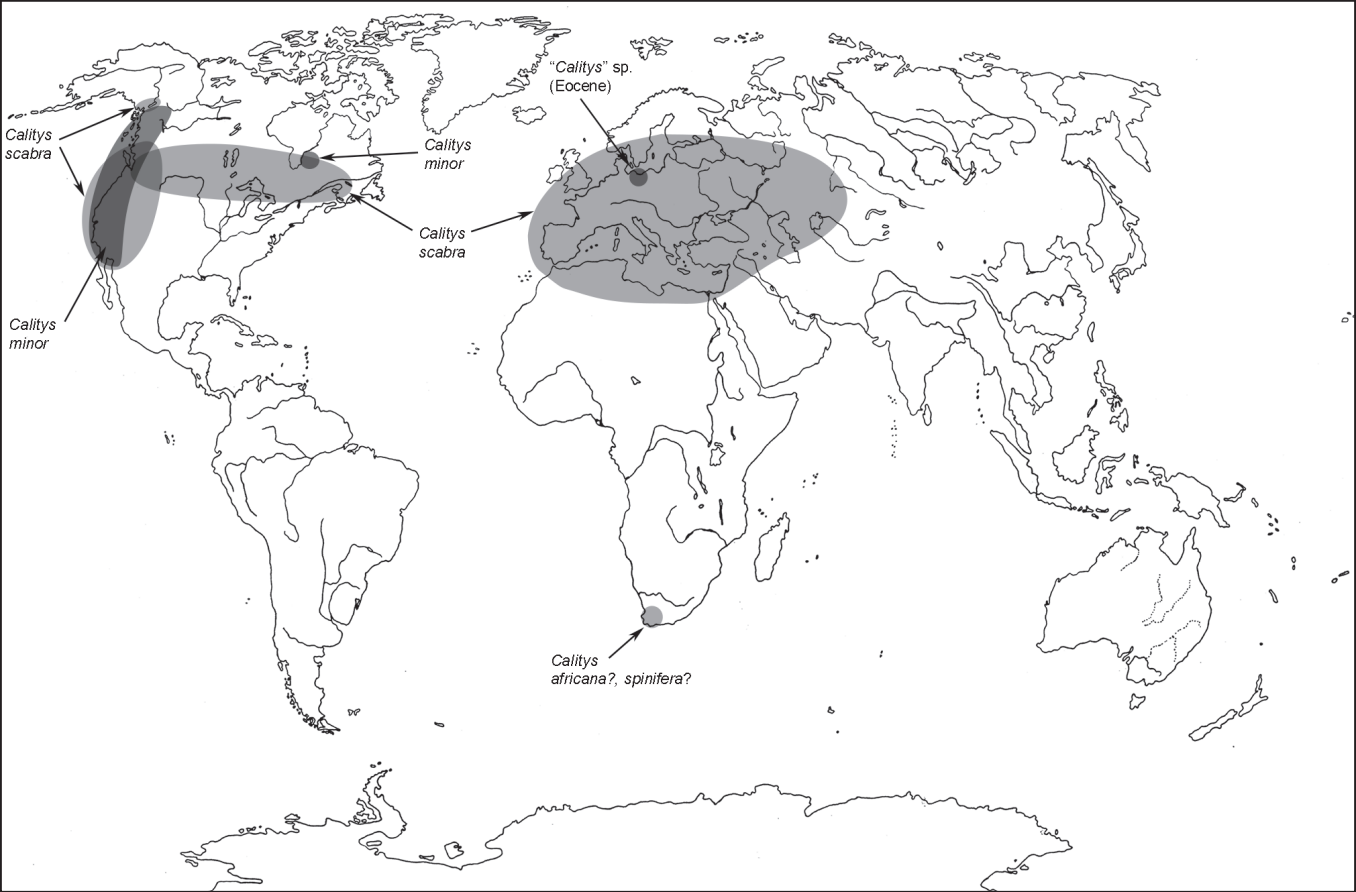
A distribution of the tribe Calityini.

###### Species:

*Calitys africana* Boheman, 1848; Caffraria (AL)

Léveillé, A. 1910: 24. Kolibáč, J., 2003: unpublished observation of non-type specimen in MHN Geneve: not Cleroidea. Reitter, E. 1876: 44. (*Nosodes africana*)

*Calitys minor* Hatch, 1962; USA, Canada (JRB)

Barron, J. R. 1971: 23. Hatch, M. H. 1962: 189

*Calitys scabra* Thunberg, 1784; Europe, Siberia to Far East, North Africa(?), USA, Canada (varA)

Léveillé, A. 1910: 24. Barron, J. R. 1971: 19. Barron, J. R. 1971: 20 (syn. *Bolitophagus silphides* Newman, 1838, synonymized by whom?). Barron, J. R. 1971: 20 (syn. *Peltis serrata* LeConte, 1859, synonymized by whom?). Barron, J. R. 1971: 20 (syn. *Silpha dentata* Fabricius, 1787, synonymized by whom?). Borowiec, L. 1983: 11. Brustel, H. 2009: 227–232 (biology). Burakowski, B. et al. 1986: 118. Dajoz, R. 1997: 44 (ecology). Hansen, S. O. & Borgersen, B. 1991: 40 (distribution in Norway). Klausnitzer, B. 1976: 5. Klausnitzer, B. 1996: 155 (larva). Klausnitzer, B. 1978: 176. Kolibáč, J. 1993a: 20 (key). Kolibáč, J. 1993b: 90. Kolibáč, J. 2005: 49 (redescription). Kolibáč, J. 2006: 106 (larva). Kolibáč, J. 2007a: 364. Kolibáč, J. et al. 2005: 132 (key). Mitter, H. 1998: 560. Pileckis, S. & V. Monsevičius 1995: 273. Reitter, E. 1876: 44 (*Nosodes*). Vogt, H. 1967: 16

*Calitys spinifera* Reitter, 1877; Cap (AL)

Léveillé, A. 1910: 24

*Calitys* sp.

Beutel, R. G. & Pollock, D. A. 2000: 826 (larva, morphology). Beutel, R. G. & Ślipiński, S. A. 2001: 219

† “*Calitys*” sp.

Larsson, S. G. 1978: 150 (fossil, Baltic amber)

##### Tribe Larinotini

Ślipiński, 1992

Larinotini Ślipiński, S. A. 1992: 443 (Larinotinae)

###### Type genus.

*Larinotus* Carter & Zeck, 1937

Bouchard, P. et al. 2011: 57. Lawrence, J. F. & Newton, A. F., Jr. 1995: 868 (Larinotinae). Kolibáč, J. 2006: 118 (Larinotini) (diagnosis, stat. n.). Kolibáč, J. 2008: 118–119 (phylogeny). Kolibáč, J. & Leschen, R. A. B. 2010: 242

###### Remarks.

The subfamily Larinotinae was originally established for the genera *Colydiopeltis*, *Parapeltis* and *Larinotus* (Ślipiński 1992). I removed the first two genera into the separate tribe Colydiopeltini under Peltinae (Kolibáč 2006), leaving Larinotini monotypic. Ślipiński (*l.c.*) remarked upon the similarity of the adult *Larinotus* and *Necrobiopsis* (Trogossitinae: Egoliini) but, on the other hand, he also pointed out common features of the *Larinotus* larva and some Peltinae (Rentoniinae, *Protopeltis* in his original text). Crowson (1970) also classified *Larinotus* (described as *Nebophilus hirsutus*) within Egoliini. All the opinions of the both these authors are in perfect agreement with my observations (Kolibáč 2006, 2008). Finally, character analyses (*l.c.*) also resulted in a close relationship between Larinotini and Egoliini. The relationship of adults appears to be so close that one might speculate about the paraphyly of Larinotini; it is, however, hard to justify such a hypothesis if one considers the monotypy of the latter (all derived features found in *Larinotus* are only autapomorphies).

The closed mesocoxal cavities are the single apomorphy distinguishing Larinotini from adult Egoliini. Larval characters connecting *Larinotus* with Peltinae are: (1) cranium with median endocarina, (2) gula with anterior apodemes, (3) strongly reduced urogomphi (minute, apices downturned). However, character states 1 and 2 are considered plesiomorphies while the third character state (reduction of urogomphi) could be a tendency observed in various cleroids living in tightly-confined surroundings (e.g. *Peltis*, Cleridae: *Dermestoides*).

##### Genus Larinotus

Carter & Zeck, 1937

http://species-id.net/wiki/Larinotus

Fig. 3 ; Map 2

Colydidae Carter, H. J. & Zeck, E. H. 1937: 186. (*sub* )

###### Type species.

*Larinotus umblicatus* Carter & Zeck, 1937 [by monotypy]

Kolibáč, J. 2005: 62 (redescription). Kolibáč, J. 2006: 111 (phylogeny). Ślipiński, S. A. 1992: 455 (redescription)

*Nebophilus* Crowson, 1970 [type species: *Nebophilus hirsutus* Crowson, 1970; designated by author]

Crowson, R. A. 1970: 14. Lawrence, J. F. 1980: 307 (synonymized with *Larinotus umblicatus*)

###### Description

(see also Crowson 1970, Ślipiński 1992). Body size: 3.5 mm. Body shape convex (not conglobate). Gular sutures wide, convergent at apex. Frontoclypeal suture present. Frons: longitudinal groove or depression absent. Cranium ventrally: tufts of long setae at sides absent. Submentum: ctenidium absent. Antennal groove present. Eyes: size moderate. Eyes number: two. Epicranial acumination moderate. Lacinial hooks: two. Galea: shape elongate. Galea: ciliate setae absent. Mediostipes-Lacinia not fused. Palpifer: outer edge even. Mandibular apical teeth number: two, horizontally situated. Mola present. Penicillus (at base) absent. Pubescence above mola or cutting edge absent. Ventral furrow absent. Basal notch shallow or absent. Labrum-Cranium not fused. Epipharyngial sclerite absent. Lateral tormal process: projection curved downwards, processes with bridge (*Peltis*). Ligula: ciliate setae absent. Ligula rigid, not retroflex, weakly emarginate. Hypopharyngeal sclerite consisting of two separate parts. Antenna 9-segmented. Antennal club symmetrical, sensorial fields absent. Front coxal cavities externally closed, internally open. Pronotum transverse. Prepectus absent. Middle coxal cavities closed. Elytra: long hairs present. Epipleuron moderate. Elytral interlocking mechanism present, carinae reduced. Elytral punctation regular, scales absent. Wing: radial cell triangular, wedge cell present, cross vein MP3-4 absent, cross vein AA1+2-3+4 present. Front tibiae: spines along side reduced, hooked spur present. Claws: denticle absent. Spiculum gastrale absent. Tegmen composed of two parts.

Larva: Frontal arms weakly curved. Epicranial stem reduced. Endocarina present. Gular sutures conspicuous, parallel. Gula: anterior apodemes present. Paragular sclerites absent. Hypostomal rods absent. Stemmata number: five. Mandible apical teeth number: two, horizontally situated. Lacinia mandibulae with several small spines. Mola absent. Maxillary palpi 3-segmented. Palpifer present. Pedunculate seta absent. Mala simple. Mala: bidentate protrusion absent. Cardo-Stipes not fused. Cardo: size much smaller than stipes. Ligula absent. Labial palpi 2-segmented. Prementum in single part, anterior margin projecting. Torma: two separate lateral sclerites. Antennal joints 1, 2 transverse. Sensory appendix larger than half of joint 3. Thoracic sclerites pattern (dorsally) 0+0+0. Thoracic sclerites pattern (ventrally) 0+0+0. Trochanter triangular. Abdominal segment IX not divided. Tergite IX depressed. Urogomphi minute; median process absent.

###### Biology.

The larvae and adults were found together by J. Doyen “*in rotten wood beneath resupinate fungi*” (Ślipiński 1992).

###### Distribution.

Australia: Northern Queensland.

**Map 2.**
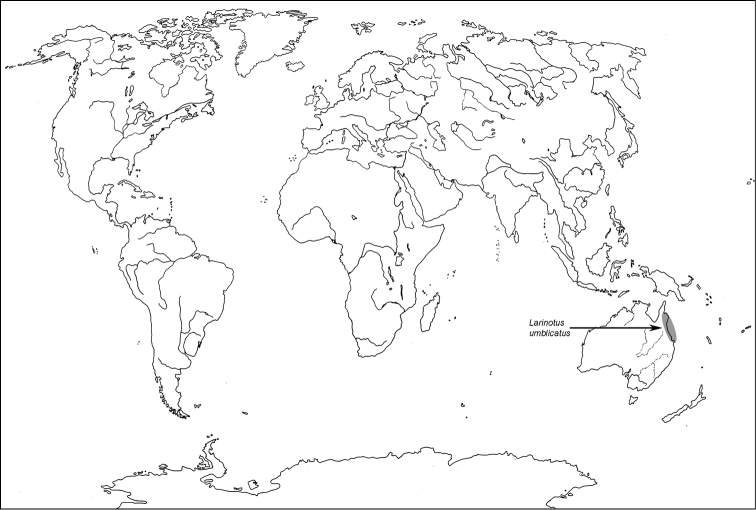
A distribution of the tribe Larinotini.

###### Species:

*Larinotus umblicatus* Carter & Zeck, 1937; Australia: Queensland (varA)

Carter, H. J. & Zeck, E. H. 1937: 186. Crowson, R. A. 1970: 16 (*Nebophilus hirsutus*). Lawrence, J. F. 1980: 307 (syn. *Nebophilus hirsutus* Crowson, 1970; synonymized by author). Ślipiński, S. A. 1992: 455 (redescription adult, description larva). Kolibáč, J. 2005: 62

##### Tribe Egoliini

Lacordaire, 1854

Egoliini Lacordaire, J. T. 1854: 334.

###### Type genus.

*Egolia* Erichson, 1842

Arias, E. et al. 2009: 37. Bouchard, P. et al. 2011: 57. Crowson, R. A. 1964a: 287 (Egoliinae). Lawrence, J. F. & Newton, A. F., Jr. 1995: 869 (Egoliinae). Ślipiński, S. A. 1992: 442 (Egoliinae). Kolibáč, J. 2006: 106, 119 (larval morphology; stat. n.; phylogeny). Kolibáč, J. 2008: 118–119 (phylogeny)

###### Remarks.

This tribe exhibits several primitive features (for example, mandibles with distinct mola) and belongs among the basal groups of Trogossitinae. While earlier entomologists always classified *Acalanthis*, *Egolia* and *Calanthosoma* together with *Nemozoma* and allied genera (for example, in the “Nemozomini” of Reitter 1876 and “Nemosomatinae” of Léveillé 1910), i.e. they always associated the present tribe with Trogossitinae, Crowson (1964a, 1966) was convinced of a relationship to Peltidae. His main reason was perhaps the relative primitiveness of adult egoliins with respect to trogossitids *s.lat.* Later, Crowson (1970) transferred it to Trogossitidae *s.str.*, together with his newly-established subfamily Calitinae (in fact, only a change of status for Calityini Reitter, 1922). Having examined larvae of Egoliinae (*Acalanthis*, *Paracalanthis*) I consider its classification with modern trogossitins undeniable.

###### Key to genera

**Table:** 

1	Pronotum distinctly elongate; antennae 11-segmented, club 3-segmented with sensorial fields; elytra bare, long hairs at elytral apex only	*Calanthosoma*
–	Pronotum cordate or transverse; antennae 8- or 10-segmented, club 1- or 2-segmented without sensorial fields; elytra and pronotum with long hairs or, rarely, perfectly bare	2
2	Pronotum transverse; frontoclypeal suture present; antennae 8-segmented, club 1-segmented (composed of 3 united segments)	*Necrobiopsis*
–	Pronotum cordate; frontoclypeal suture absent; antennae 10-segmented, club 2-segmented	3
3	Dorsal surface bare	*Egolia*
–	Dorsal surface with long, pale hairs	4
4	Head, pronotum and elytra black or elytral apex dark blue; each elytron with two orange spots or with two transverse bands composed of light pubescence	*Acalanthis*
–	Head, pronotum and elytra brown; elytra with X-shaped yellowish spot in anterior half and transverse yellowish stripe in apical half	*Paracalanthis*

##### Genus Acalanthis

Erichson, 1844

http://species-id.net/wiki/Acalanthis

**Map 3.**
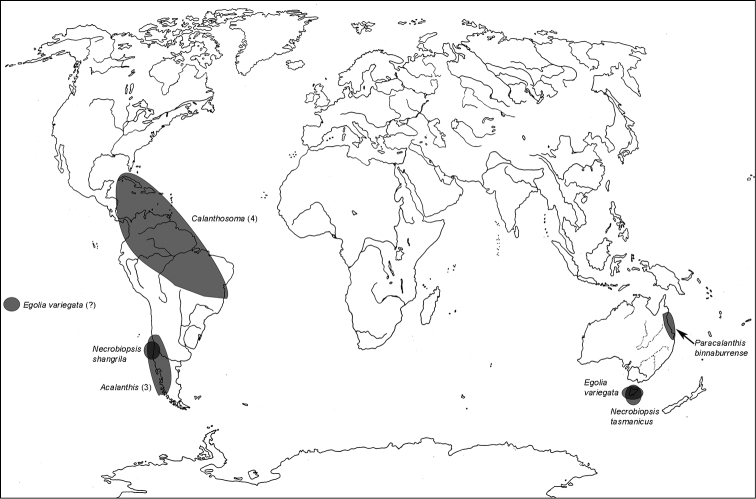
A distribution of the tribe Egoliini.

Figs 1 , 3 , 13 ; Map 3

Acalanthis Erichson, W. F. 1844: 446.

###### Type species.

*Acalanthis quadrisignata* Erichson, 1844 [by monotypy]

Léveillé, A. 1910: 4. Arias, E. et al. 2009: 39. Crowson, R. A. 1964a: 299 (larva, described as *Phanodesta*). Crowson, R. A. 1970: 13. Kolibáč, J. 2005: 40. Kolibáč, J. 2006: 106 (larva; see Crowson 1964a)

###### Remarks.

An antennal apex I figured (Kolibáč 2005: 89, Pl. 1-Fig. 5) was associated with *Acalanthis quadrisignata* in error, as correctly noted by Arias et al. (2009). The drawing probably belongs to *Calanthosoma flavomaculata*.

###### Description.

Body size: 6.0–9.0 mm. Body shape elongate. Gular sutures narrow, subparallel at apex. Frontoclypeal suture absent. Frons: longitudinal groove or depression absent. Cranium ventrally: tufts of long setae at sides present. Submentum: ctenidium absent. Antennal groove present. Eyes: size moderate Eyes number: two. Epicranial acumination moderate. Lacinial hooks: two. Galea: shape sub-clavate. Galea: ciliate setae absent. Mediostipes-Lacinia partially fused. Palpifer: outer edge even. Mandibular apical teeth number: two, horizontally situated. Mola reduced but present. Penicillus (at base) present (fine, often membranous). Pubescence above mola or cutting edge present. Ventral furrow present. Basal notch moderate. Labrum-Cranium not fused. Epipharyngial sclerite present. Lateral tormal process: projection curved downwards, processes not connected (*Airora*). Ligula: ciliate setae absent. Ligula rigid, weakly retroflex, deeply emarginate. Hypopharyngeal sclerite H-shaped. Antenna 10-segmented, Antennal club asymmetrical, sensorial fields present. Front coxal cavities externally closed, internally open. Pronotum cordate. Prepectus absent. Middle coxal cavities closed. Elytra: long hairs present. Epipleuron moderate. Elytral interlocking mechanism present, carinae reduced. Elytral punctation regular, scales absent. Wing: radial cell triangular, wedge cell present, cross vein MP3-4 present, cross vein AA1+2-3+4 absent. Front tibiae: spines along side moderate. Hooked spur present. Claws: denticle absent. Parasternites number along ventrites III–VII: one. Spiculum gastrale absent. Tegmen composed of three parts. Coxitae undivided.

Larva: Frontal arms V-shaped. Epicranial stem reduced. Endocarina present. Antennal joints 1 and 2 elongate. Thoracic sclerites pattern (dorsally) 1-2-2. Tergite IX depressed. Urogomphi present, hooked; median process absent.

###### Biology.

Predatory. Crowson (1964a) remarked “*numerous insect fragments in gut*” of an assumed *Phanodesta* larva but, as he noted later (1970: 1), the larva probably belongs to some species of *Acalanthis*. Its brief description in his key (1964a: 299) in fact agrees perfectly with the larval characters of Egoliini. The adult gut contents of *Acalanthis quadrisignata* included insect fragments (Crowson 1964a). Elizabeth Arias and colleagues (Arias et al. 2009) caught numerous *Acalanthis* specimens by canopy fogging and beating vegetation in forests, yielding a range of species including some *Nothophagus*, *Araucaria*, *Schinus*, and *Rhaphitamnus*.

###### Distribution.

Temperate forests of central Chile and Argentina (Arias et al. 2009).

###### Species:

*Acalanthis mirabilis* Reitter, 1876; Chile (AL, varA)

Léveillé, A. 1910: 4. Arias, E. et al. 2009: 40

*Acalanthis quadrisignata* Erichson, 1844; Chile (AL, varA)

Léveillé, A. 1910: 4. Arias, E. et al. 2009: 39. Kolibáč, J. 1999b: 12. Kolibáč, J. 2005: 40 (redescription)

*Acalanthis semimetallica* Fairmaire, 1861 (*Clerus*); Chile (AL, varA)

Léveillé, A. 1910: 4. Arias, E. et al. 2009: 41

##### Genus Calanthosoma

Reitter, 1876

http://species-id.net/wiki/Calanthosoma

Fig. 3 ; Map 3

Calanthosoma Reitter, E. 1876: 10.

###### Type species.

*Calanthosoma flavomaculatum* Reitter, 1876 [by monotypy]

Léveillé, A. 1910: 4. Arias, E. et al. 2009: 37. Kolibáč, J. 2005: 47 (redescription). Kolibáč, J. 2006: 111 (phylogeny)

*Marnia* Léveille, 1889 [Type species: *Marniasipolisi* Léveillé, 1889; designated by Kolibáč 2005]

Léveillé, A. 1910: 14. Kolibáč, J. 2005: 47 (synonymized with *Calanthosoma*)

###### Remarks.

A single genus, it lives in tropical South America beyond the temperate “Gondwanan” distribution of the other egoliine genera. *Calanthosoma* shows a character set intermediate between Egoliini and Trogossitini. Longitudinal wrinkles on the dorsal surface of head and pronotum, long hairs at apex of elytra, mandibles with mola, and lacinia with distinct hooked spine at apex are typical of the egoliins; however, the genus shares ciliate labial setae and terminal antennomeres with sensorial fields with most of trogossitins. Nevertheless, most of apomorphic characters are clearly egoliine and I have no doubts about its classification within that tribe. Common trogossitine features of *Calanthosoma* may provide evidence of a common ancestor for Egoliini and Trogossitini.

The synonymization of *Marnia* with *Calanthosoma* is beyond doubt; the representatives of the genera are very similar.

###### Description.

Body size: about 9.0 mm. Body shape elongate. Gular sutures narrow, subparallel at apex. Frontoclypeal suture absent. Frons: longitudinal groove or depression absent. Cranium ventrally: tufts of long setae at sides present. Submentum of males: ctenidium present. Antennal groove present. Eyes: size moderate. Eyes number: two. Epicranial acumination moderate. Lacinial hooks: one. Galea: shape clavate. Galea: ciliate setae present. Mediostipes-Lacinia partially fused. Palpifer: outer edge even. Mandibular apical teeth number: two, vertically situated. Mola present. Penicillus (at base) present (fine, often membranous). Pubescence above mola or cutting edge absent. Ventral furrow not ciliate. Basal notch moderate. Labrum-Cranium not fused. Epipharyngial sclerite present. Lateral tormal process: projection curved downwards, processes not connected (*Airora*). Ligula: ciliate setae absent. Ligula rigid, weakly retroflex, weakly emarginate. Hypopharyngeal sclerite consisting of two separate parts. Antenna 11-segmented. Antennal club asymmetrical, sensorial fields present. Front coxal cavities externally closed, internally open. Pronotum cordate. Prepectus absent. Middle coxal cavities closed. Elytra: long hairs present. Epipleuron moderate. Elytral interlocking mechanism present, carinae conspicuous. Elytral punctation regular, scales absent. Wing: radial cell oblong (or reduced), wedge cell present, cross vein MP3-4 present, cross vein AA1+2-3+4 absent. Front tibiae: spines along side moderate. Hooked spur present. Claws: denticle absent. Parasternites number along ventrites III–VII: one. Spiculum gastrale absent. Tegmen composed of three parts. Coxitae undivided.

###### Biology.

Unknown, probably predatory.

###### Distribution.

Only a few specimens is known to date: Venezuela, Brazil, Antilles.

###### Species:

*Calanthosoma flavomaculatum* Reitter, 1876; Antillae, Venezuela (AL, JK)

Léveillé, A. 1910: 4. Kolibáč, J. 2005: 47 (redescription). Reitter, E. 1876: 11

*Calanthosoma grouvellei* Léveille, 1899 (*Marnia*); Brazil (AL)

Léveillé, A. 1910: 14

*Calanthosoma sallei* Léveille, 1889 (*Marnia*); Venezuela (AL)

(J. Kolibáč, unpublished note: maybe synonymous with *Calanthosoma flavomaculatum*)

*Calanthosoma sipolisi* Léveille, 1889 (*Marnia*); Brazil: Minas Geraes (AL)

Léveillé, A. 1910: 14. Kolibáč, J. 2005: 47

##### Genus Egolia

Erichson, 1842

http://species-id.net/wiki/Egolia

Fig. 3 ; Map 3

Egolia Erichson, W. F. 1842: 150.

###### Type species.

*Egolia variegata* Erichson, 1842 [by monotypy]

Léveillé, A. 1910: 4. Crowson, R. A. 1970: 13. Kolibáč, J. 2005: 54. Kolibáč, J. 2006: 111 (phylogeny). Reitter, E. 1876: 8

###### Description.

Body size: about 9.0 mm. Body shape elongate. Gular sutures narrow, subparallel at apex. Frontoclypeal suture absent. Frons: longitudinal groove or depression absent. Submentum: ctenidium absent. Antennal groove present. Eyes: size moderate. Eyes number: two. Epicranial acumination moderate. Lacinial hooks: two. Galea: shape sub-clavate. Galea: ciliate setae absent. Mediostipes-Lacinia partially fused. Palpifer: outer edge even. Mandibular apical teeth number: two, horizontally situated. Mola present. Penicillus (at base) present (fine, often membranous). Pubescence above mola or cutting edge present. Ventral furrow present. Basal notch moderate. Labrum-Cranium not fused. Epipharyngial sclerite present. Lateral tormal process: projection curved downwards, processes not connected (*Airora*). Ligula: ciliate setae absent. Ligula rigid, weakly retroflexed, deeply emarginate. Hypopharyngeal sclerite H-shaped. Antenna 10-segmented. Antennal club asymmetrical, sensorial fields present. Front coxal cavities externally closed, internally open. Pronotum cordate. Prepectus absent. Middle coxal cavities closed. Elytra: long hairs absent. Epipleuron moderate. Elytral interlocking mechanism present, carinae reduced. Elytral punctation regular, scales absent. Wing: radial cell triangular, wedge cell present, cross vein MP3-4 present, cross vein AA1+2-3+4 absent. Front tibiae: spines along side reduced. Hooked spur absent, apical spurs not hooked or weakly hooked. Claws: denticle absent. Parasternites number along ventrites III–VII: one. Spiculum gastrale absent. Tegmen composed of three parts.

Larva: Frontal arms V-shaped. Epicranial stem absent. Endocarina present. Gular sutures conspicuous, parallel. Gula: anterior apodemes absent. Paragular sclerites present. Hypostomal rods absent. Stemmata number: 3. Mandibular apical teeth number: two, horizontally situated. Lacinia mandibulae absent. Mola absent. Maxillary palpi 3-segmented. Palpifer present. Pedunculate seta absent. Mala bilobed. Mala: bidentate protrusion absent. Cardo-Stipes not fused. Cardo: size much smaller than stipes. Ligula absent. Labial palpi 2-segmented. Prementum consists of two parts. Torma: single compact plate. Antennal joints 1 and 2 elongate. Sensory appendix medium-sized (reaching half-way along joint 3). Thoracic sclerites pattern (dorsally) 1-2-2. Trochanter triangular. Abdominal segment IX not divided. Tergite IX depressed. Urogomphi present, hooked; median process absent.

###### Biology.

Probably predatory.

###### Distribution.

Australia: Tasmania; Tahiti?

###### Species:

*Egolia variegata* Erichson, 1842; Tasmania, Tahiti (AL)

Léveillé, A. 1910: 4. Kolibáč, J. 2005: 54 (redescription). Reitter, E. 1876: 8

##### Genus Necrobiopsis

Crowson, 1964

http://species-id.net/wiki/Necrobiopsis

Fig. 3 ; Map 3

Necrobiopsis Crowson, R. A. 1964a: 293.

###### Type species.

*Necrobiopsis tasmanicus* Crowson, 1964 [by original designation and monotypy]

Arias, E. et al. 2009: 39. Crowson, R. A. 1970: 14. Kolibáč, J. 2005: 71 (redescription). Kolibáč, J. 2006: 111 (phylogeny)

###### Description.

Body size: 3.0–4.5 mm. Body shape convex (not conglobate). Gular sutures narrow, subparallel at apex. Frontoclypeal suture present. Frons: longitudinal groove or depression absent. Submentum: ctenidium absent. Antennal groove present. Eyes: size large, lateral. Eyes number: two. Epicranial acumination moderate. Lacinial hooks: two. Galea: shape elongate. Galea: ciliate setae absent. Mediostipes-Lacinia not fused. Palpifer: outer edge even. Mandibular apical teeth number: two, vertically situated. Mola reduced but present. Penicillus (at base) present (fine, often membranous). Pubescence above mola or cutting edge absent. Ventral furrow present. Basal notch shallow or absent. Labrum-Cranium not fused. Epipharyngial sclerite present. Lateral tormal process: projection curved downwards, processes not connected (*Airora*). Ligula: ciliate setae absent. Ligula membranous, not retroflexed, weakly emarginate. Hypopharyngeal sclerite H-shaped. Antenna 8-segmented, sensorial fields present. Front coxal cavities externally closed, internally open. Pronotum transverse. Prepectus present. Middle coxal cavities closed. Elytra: long hairs present. Epipleuron thin. Elytral interlocking mechanism present, carinae conspicuous. Elytral punctation regular, scales absent. Wing: radial cell triangular, wedge cell absent, cross vein MP3-4 present, cross vein AA1+2-3+4 absent. Front tibiae: spines along side reduced. Hooked spur absent, apical spurs not hooked or weakly hooked. Claws: denticle absent. Parasternites number along ventrites III–VII: two. Spiculum gastrale present. Tegmen composed of two parts.

###### Biology.

Unknown in *Necrobiopsis tasmanicus*. The recently described Chilean species *Necrobiopsis shangrila* wascollected by canopy fogging in *Nothophagus* forests, including fogging of *Cyttaria* fungi on *Nothophagus* (Arias et al. 2009).

###### Distribution.

Tasmania, central Chile (Region VIII, Biobío).

###### Species:

*Necrobiopsis tasmanicus* Crowson, 1964; Tasmania (RAC)

Crowson, R. A. 1964a: 293. Arias, E. et al. 2009: 38. Kolibáč, J. 2005: 71 (redescription)

*Necrobiopsis shangrila* Arias, Ślipiński, Lawrence & Elgueta, 2009; Chile (AD)

Arias, E. et al. 2009: 39

##### Genus Paracalanthis

Crowson, 1970

http://species-id.net/wiki/Paracalanthis

Map 3

Paracalanthis Crowson, R. A. 1970: 14.

###### Type species:

*Paracalanthis binnaburrense* Crowson, 1970 [by original designation and monotypy]

Kolibáč, J. 2005: 74. Kolibáč, J. 2006: 116 (phylogeny)

###### Description.

Body size: about 12.0 mm. Body shape elongate. Frontoclypeal suture absent. Frons: longitudinal groove or depression absent. Antennal groove present. Eyes: size moderate. Eyes number: two. Antenna 10-segmented. Front coxal cavities externally closed, internally open. Pronotum cordate. Middle coxal cavities closed. Elytra: long hairs present. Epipleuron thin, carinae reduced. Elytral punctation regular, scales absent. Front tibiae: spines along side moderate. Hooked spur absent in middle and hind tibiae. Claws: denticle absent.

###### Biology.

Unknown. Collected “*from decayed log*” (Crowson 1970).

###### Distribution.

Australia: Queensland.

###### Species:

*Paracalanthis binnaburrense* Crowson, 1970; Australia: Queensland (RAC)

Crowson, R. A. 1970: 14, 16 (larva). Kolibáč, J. 2005: 74. Kolibáč, J. 2006: 108 (larva)

##### Tribe Gymnochilini

Lacordaire, 1854

Gymnochilini Lacordaire, J. T. 1854: 344.

###### Type genus.

*Gymnochila* Erichson, 1844 (= *Gymnocheilis* Dejean, 1835)

Bouchard, P. et al. 2011: 57. Burakowski, B. et al. 1986: 118 (Gymnochilinae). Kolibáč, J. 2006: 119 (diagnosis, stat. n.). Kolibáč, J. 2007a: 364. Kolibáč, J. 2008: 118–119 (phylogeny). Leschen, R. A. B. & Lackner, T. 2013: 283

###### Remarks.

Five genera of Gymnochilini (namely *Anacypta*, *Gymnocheilis*, *Narcisa*, *Xenoglena*, *Leperina*) form, beyond doubt, a monophyletic group. Gymnochilins constitute an advanced group of Trogossitinae, adapted to a predatory way of life. They are rapid flyers, dwelling on fallen logs and hunting for bostrichids, scolytids and other insects, strongly resembling the jewel beetles (Buprestidae) in their body shape and movement. Two distinctly separated pairs of eyes in most of them and their ability to jump (*Anacypta*) characterize the tribe as one of the most advanced of all trogossitids. Some remarks about the independent status of the tribe Gymnochilini with regard to Trogossitini are made below, in the section relating the latter tribe.

The inclusion of *Phanodesta* from Juan Fernandez Isl. was more or less confirmed by two separate character analyses (Kolibáč 2006, 2008). However, the phylogeny of the genus was rendered unclear by the number of autapomorphies (e.g. winglessness, characteristic elytral structure) and separated distribution. I postulated a a sister group of *Leperina* with a “Gondwanan” distribution (Australia-Chile). Recently, a phylogeny and distribution of *Phanodesta*, together with descriptions and combinations of some more species from New Zealand and its vicinity have been addressed by Leschen and Lackner (2013) in detail. Two more genera occured in the gymnochiline clade in my second analysis (Kolibáč 2008): *Seidlitzella* and *Melambia*. The first genus, *Seidlitzella*, was considered related to the Palaearctic species of *Leperina*, as also pointed out by Schawaller (1993). However, his formal synonymization of *Seidlitzella* was not confirmed in a recent study by Leschen and Lackner (2013) who established the new genus *Kolibacia* for *Leperina tibialis* and *Leperina squamulata* instead.

In both analyses by Kolibáč (2006, 2008), the genera *Seidlitzella* and *Melambia* were considered primitive or basal among the Gymnochilini or Trogossitini. They were included in the trogossitins in my original tribe definition (Kolibáč 2006). A comparison made specifically for the current shows the heterogenity of *Melambia* species and the need for revision of the genus with respect to the systematic position of particular species. It cannot be excluded that there are some species of *Melambia* congeneric with *Alindria*.

###### Key to genera

**Table:** 

1	Head with 1 pair of eyes. Ventral part of cranium with long setae at sides. Radial cell triangular and moved down, or reduced. Elytra with conspicuous carinae	2
–	Head with 2 pairs of eyes. Ventral part of cranium without long setae at sides. Radial cell mostly oblong. Elytral carinae reduced or inconspicuous	5
2	Body bare, without setae. Window punctures absent. Unicolorous, black species	*Seidlitzella*
–	Body surface with vestiture consisting of scales or setae or both. Window punctures absent or present. Dorsal side brown or black-brown, with colour patterns formed by vestiture	3
3	Dorsal vestiture consisting of scales. Elytral carinae not beaded; intercarinal space punctate; window punctures present. Mucro absent	4
–	Dorsal vestiture consisting of setae, scales or both. Elytral carinae usually beaded; intercarinal space apunctate; window punctures absent. Mucro absent or weakly developed	*Phanodesta*
4	Elytral intercarinal space bipunctate; window punctures simple. Protibial edge smooth. Mandibles with mola	*Kolibacia*
–	Elytral intercarinal space multipunctate; window punctures tuberculate. Protibial edge spinate. Mandibles without mola	*Leperina*
5	Body rather flat, compact; smaller species (about 4–8 mm)	6
–	Body rather cylindrical, elongate; larger species (about 6–20 mm)	7
6	Body perfectly covered with large scales	*Narcisa*
–	Body without scales or pubescence, with metallic lustre	*Anacypta*
7	Body with colour pattern composed of scales and short thick setae; antennal club large	*Gymnocheilis*
–	Body without scales, spots on pronotum and elytra formed by short setae; antennal club smaller	*Xenoglena*

##### Genus Anacypta

Illiger, 1807

http://species-id.net/wiki/Anacypta

Figs 4 , 14 ; Map 4

Anacypta Illiger, J. K. W. 1807: 301.

###### Type species.

*Nitidula punctata* Fabricius, 1801 [designated by Kolibáč 2005]

Kolibáč, J. 2005: 44 (redescription). Kolibáč, J. 2006: 111 (phylogeny). Kolibáč, J. 2007a: 364

*Acrops* Dalman, 1824 [Type species: *Acrops metallica* Dalman, 1824 (= *Nitidula punctata* Fabricius, 1801)]

Léveillé, A. 1910: 23

###### Description.

Body size: about 4.5–7.0 mm. Body shape flat. Gular sutures wide, convergent at apex. Frontoclypeal suture absent. Frons: longitudinal groove or depression absent. Cranium ventrally: tufts of long setae at sides absent. Submentum: ctenidium present. Antennal groove present. Eyes: size large, dorsal. Eyes number: four. Epicranial acumination moderate. Lacinial hooks absent. Galea: shape clavate. Galea: ciliate setae absent. Mediostipes-Lacinia partially fused. Palpifer: outer edge denticulate. Mandibular apical teeth number: two, horizontally situated. Mola absent. Penicillus (at base) present (fine, often membranous). Pubescence above mola or cutting edge absent. Ventral furrow absent. Basal notch shallow or absent. Labrum-Cranium not fused. Epipharyngial sclerite absent. Lateral tormal process: projection projection not developed (all remaining). Ligula: ciliate setae absent. Ligula rigid, weakly retroflexed, weakly emarginate. Hypopharyngeal sclerite consisting of two separate parts. Antenna 10-segmented. Antennal club asymmetrical, sensorial fields present. Front coxal cavities externally closed, internally open. Pronotum transverse. Prepectus absent. Middle coxal cavities open. Elytra: long hairs absent. Epipleuron thin. Elytral interlocking mechanism present, carinae reduced. Elytral punctation regular, scales absent. Wing: radial cell oblong (or reduced), wedge cell absent, cross vein MP3-4 absent, cross vein AA1+2-3+4 present. Front tibiae: spines along side reduced. Hooked spur present. Claws: denticle absent. Parasternites number along ventrites III–VII: one. Spiculum gastrale absent. Tegmen composed of three parts. Coxitae undivided.

**Figure 4.**
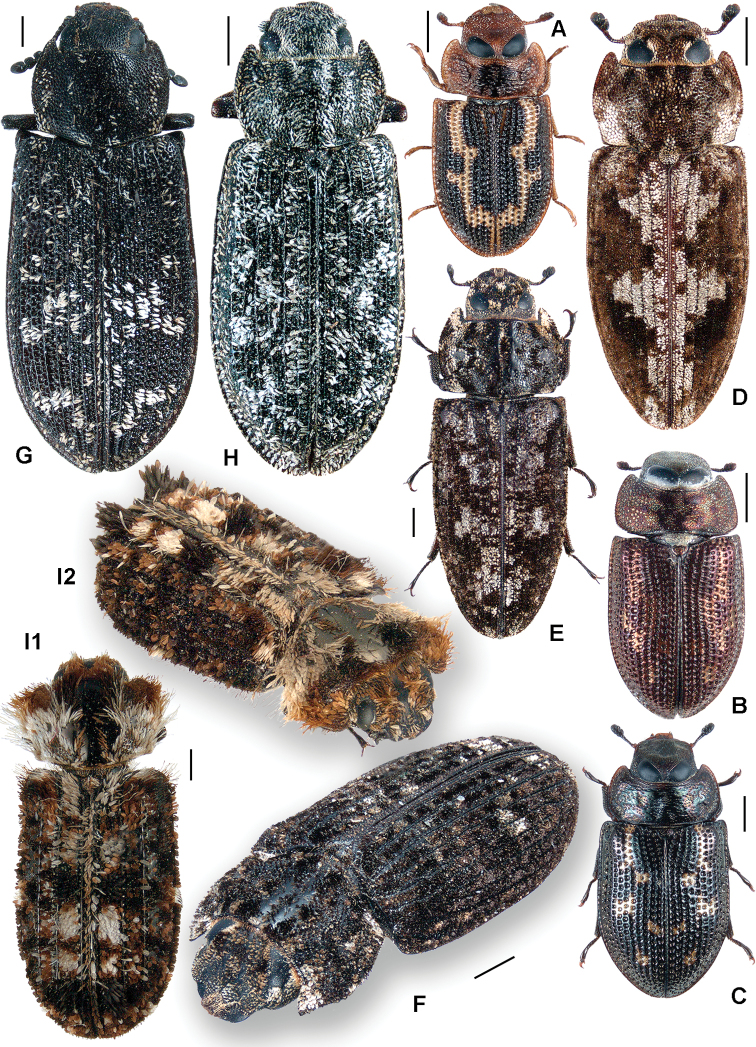
**A** *Anacypta* sp., Vietnam **B** *Anacypta punctata* **C** *Anacypta* sp., Laos **D** *Gymnocheilis subfasciata* **E** *Gymnocheilis* sp., Ghana **F** *Gymnocheilis varia* **G** *Kolibacia regularis* **H** *Kolibacia squamulata* **I** *Leperina cirrosa*.

###### Biology.

Predatory. Adults run rapidly on logs and branches of fallen trees, hunting for prey. If disturbed, they fly very quickly. Some beetle collectors remark (P. Pacholátko, pers. comm.; experience from southern India) that some species can even jump(!) before they fly off.

###### Distribution.

South-eastern Asia including Indonesia, Laos, Vietnam (numerous modern unpublished records). Often recorded together with species of *Xenoglena*.

**Map 4.**
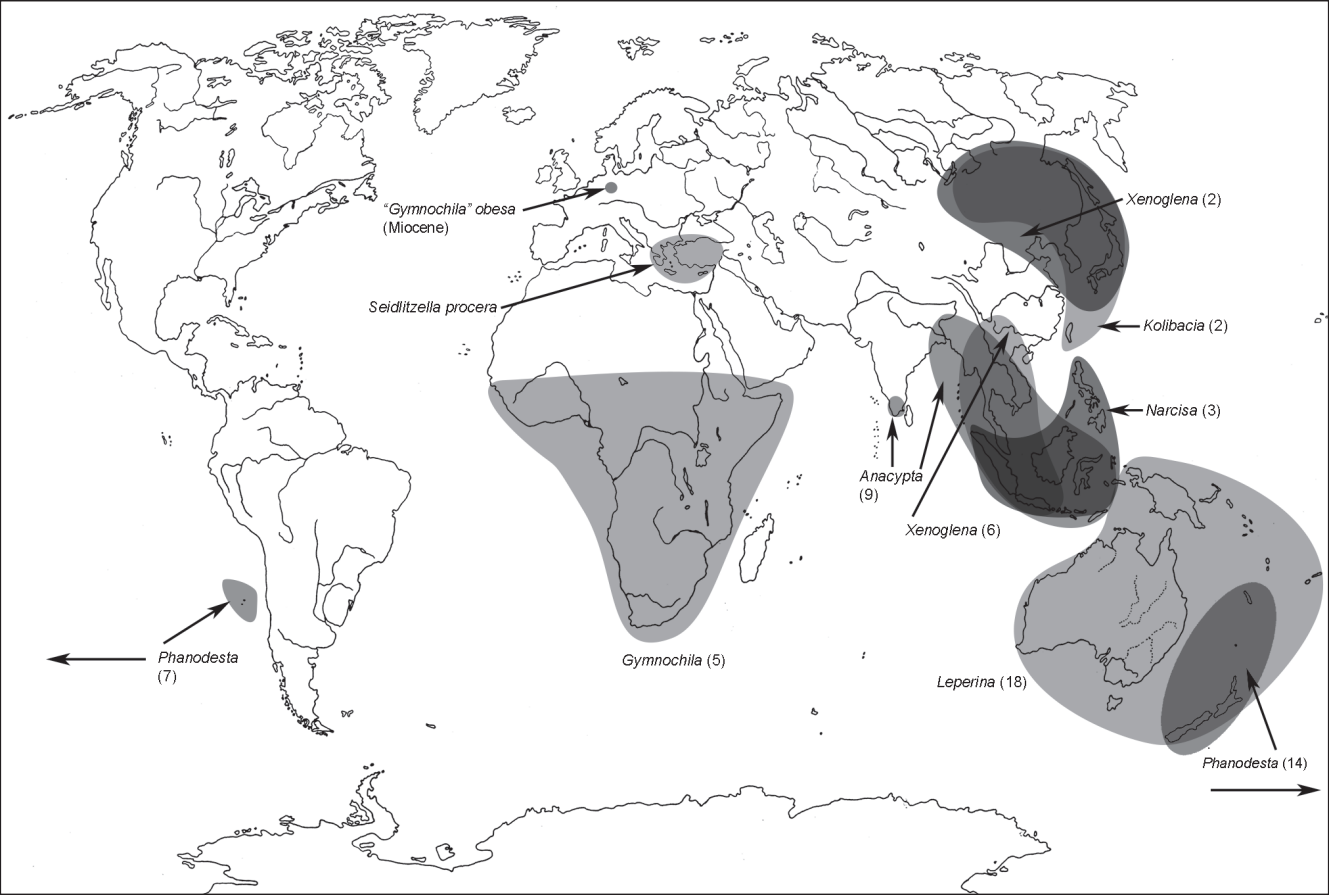
A distribution of the tribe Gymnochilini.

###### Species:

*Anacypta birmanica* Léveillé, 1888; Burma (AL)

Léveillé, A. 1910: 23 (*Acrops*)

*Anacypta cicatricosa* Reitter, 1880; „Himalaya” (AL)

Léveillé, A. 1910: 23 (*Acrops*). Léveillé, A. 1910: 23 (*Anacypta cicatricosa* var. *rugosa* Léveillé, 1899). Kolibáč, J. 2007a: 364 (syn. *Anacypta rugosa* Léveillé, 1899)

*Anacypta cyanea* Léveillé, 1899; Perak (AL)

Léveillé, A. 1910: 23 (*Acrops*)

*Anacypta feai* Léveillé, 1888; Tenasserim (AL)

Léveillé, A. 1910: 23 (*Acrops*)

*Anacypta gambeyi* Léveillé, 1890; “Cochinchina” (AL)

Léveillé, A. 1910: 23 (*Acrops*)

*Anacypta higonia* Lewis, 1888; Japan (AL, varA)

Léveillé, A. 1910: 23 (*Acrops*). Kolibáč, J. 2007a: 364. Nakane, T. et al. 1963: 181 (*Acrops*)

*Anacypta perraudierei* Léveillé, 1905; Tonkin (AL)

Léveillé, A. 1910: 23 (*Acrops*)

*Anacypta punctata* Fabricius, 1801; Sumatra, Moluccae, Borneo (AL)

Léveillé, A. 1910: 23

Léveillé, A. 1910: 23 (syn. *buprestoides* Weber, 1801); (Sumatra, Moluccae) (AL)

Léveillé, A. 1910: 23 (syn. *metallica* Dalman, 1824); (Borneo) (AL)

Léveillé, A. 1910: 23 (syn. *punctata* var. *dohrni* Reitter, 1876). Kolibáč, J. 2005: 44 (redescription)

*Anacypta weyersi* Léveillé, 1900; Sumatra (AL)

Léveillé, A. 1910: 23 (*Acrops*)

##### Genus Gymnocheilis

Dejean, 1835

http://species-id.net/wiki/Gymnocheilis

Figs 4 , 13 , 14 ; Map 4

Gymnocheilis Dejean, P. F. M. A. 1835: 314

###### Type species.

[Type species: *Peltis squamosa* Gray in Griffith and Pidgeon 1832; by monotypy]

Léveillé, A. 1910: 22 (*Gymnochila*). Kolibáč, J. 2005: 60 (*Gymnochila*, redescription). Kolibáč, J. 2006: 111 (*Gymnochila*, phylogeny). Reitter, E. 1876: 37 (*Gymnochila*). Leschen, R. A. B. & Lackner, T. 2013: 279, 289

*Lepidopteryx* Hope, 1840 [Type species: *Peltis squamosa* Gray in Griffith and Pidgeon 1832; not *Lepidopteryx squamosa* Hope, 1840] See below for explanation of this synonymy

*Gymnochila* Erichson, 1844 [Type species: *Trogosita vestita* Griffith, 1832 (= *Gymnochila varia* Fabricius, 1801); by monotypy]

###### Remarks.

Yves Bousquet (pers. comm., 2010) kindly checked the nomenclatoral problem of *Leperina* that also bears upon the name of *Gymnocheilis*: “*... the type species of* Lepidopteryx *is* Trogositas quamosa *Gray, 1832 described from ‘Melville’s Island’, not* Lepidopteryx squamosa *Hope, 1840. In his second and third catalogues, Dejean proposed the genus* Gymnocheilis *for* squamosa *Gray. So* Lepidopteryx *Hope, 1840 is a senior synonym of* Gymnochila *Erichson, 1844, which in turn is a junior synonym of* Gymnocheilis *Dejean, 1835.*” The correct name *Gymnocheilis* has already been used by Leschen and Lackner (2013).

###### Description.

Body size: about 9.0–14.0 mm. Body shape elongate. Gular sutures reduced. Frontoclypeal suture absent. Frons: longitudinal groove or depression absent. Cranium ventrally: tufts of long setae at sides absent. Submentum of males: ctenidium present. Submentum: ctenidium present. Antennal groove present. Eyes: size large, dorsal. Eyes number: four. Epicranial acumination deep. Lacinial hooks absent. Galea: shape clavate. Galea: ciliate setae absent. Mediostipes-Lacinia fused together. Palpifer: outer edge denticulate. Mandibular apical teeth number: one. Mola present. Penicillus (at base) present (fine, often membranous). Pubescence above mola or cutting edge absent. Ventral furrow absent. Basal notch deep. Labrum-Cranium not fused. Epipharyngial sclerite present. Lateral tormal process: projection projection not developed (all remaining). Ligula: ciliate setae absent. Ligula rigid, not retroflexed, weakly emarginate. Hypopharyngeal sclerite absent. Antenna 11-segmented. Antennal club asymmetrical, sensorial fields present. Front coxal cavities externally closed, internally open. Pronotum transverse. Prepectus absent. Middle coxal cavities closed. Elytra: long hairs absent. Epipleuron thin. Elytral interlocking mechanism present, carinae reduced. Elytral punctation regular, scales present. Wing: radial cell oblong (or reduced), wedge cell present, cross vein MP3-4 present, cross vein AA1+2-3+4 present. Front tibiae: spines along side moderate. Hooked spur present. Claws: denticle absent. Parasternites number along ventrites III–VII: one. Spiculum gastrale absent. Tegmen composed of three parts.

###### Biology.

Probably predatory. Sometimes collected at light.

###### Distribution.

Africa south of the equator.

###### Species:

*Gymnocheilis lepidoptera* Reitter, 1876; Abyssinia (AL)

Léveillé, A. 1910: 22. Reitter, E. 1876: 39

† *Gymnocheilis obesa* Heer, 1862; Germany: Öhningen; Tertiary: Middle Eocene (varA)

Ponomarenko, A. G. & Kireichuk, A. G. 2005–2008: http://www.zin.ru/animalia/Coleoptera/rus/paleosy2.htm. Schmied, H. et al. 2009: 23

*Gymnocheilis rugosa* Thunberg, 1808; Guinea (AL)

*Gymnocheilis sparsuta* J. Thomson, 1858; Gabon (AL)

Léveillé, A. 1910: 22. (syn. *Gymnochila angulicollis* J. Thomson, 1858). Kolibáč, J. 2005: 60 (redescription). Reitter, E. 1876: 38

*Gymnocheilis subfasciata* J. Thomson, 1858; Gabon (AL)

Léveillé, A. 1910: 22. Reitter, E. 1876: 39

*Gymnocheilis varia* Fabricius, 1801; South Africa (AL)

Léveillé, A. 1910: 22 (syn. *Gymnochila adspersa* Boheman, 1848) (Caffraria)

Léveillé, A. 1910: 22 (syn. *Gymnochila laticollis* Boheman, 1848) (Caffraria)

Léveillé, A. 1910: 22 (syn. *Gymnochila squamosa* Gray in Griffith 1832) (Cap)

Léveillé, A. 1910: 22. (syn. *Gymnochila vestita* Fabricius, 1844) (Cap, South Africa)

Kolibáč, J. 2005: 60 (redescription). Reitter, E. 1876: 38

##### Genus Kolibacia

Leschen & Lackner, 2013

http://species-id.net/wiki/Kolibacia

Fig. 4 ; Map 4

Kolibacia Leschen, R. A. B. & Lackner, T. 2013: 288.

###### Type species.

*Leperina tibialis* Reitter, 1889 [designated by Leschen and Lackner 2013]

Schawaller, W. 1993: 4 (*Leperina* and *Seidlitzella*, Palaearctic species). Yoshitomi, H. & Lee, C.-F. (in press)

###### Description

(according to Leschen and Lackner 2013). Body size: 7.2–9.1 mm. Colour of body black. Dorsal vestiture consisting of scales. Head extending beyond anterior angles of pronotum. Frons more or less horizontal with mandibles visible from above. Median lobe of clypeus absent. Edge of labrum straight. Eyes entire. Gena acute. Supraocular scales present. Antenna 11-segmented with a loose club; lengths of antennomere II and III equal; antennomere XI circular, about as long as wide. Prothorax with lateral carinae unevenly crenulate; anterior angles projecting or acute; posterior angles angulate. Surface of pronotal disc even; punctation uniform and bearing scales. Procoxae visible in lateral view. Hypomeron bearing scales; anterior portion rugose. Length of elytra 4 × as long as pronotum or even longer; seven elytral carinae present, not beaded and not rising significantly above surface of elytral disc; sublateral keel absent; intercarinal space bipunctate; window punctures present and simple; intercarinal scales never countersunk within punctures; lateral carina simple; epipleuron visible in lateral view. Hind wings present, fully developed; MP3 spur absent. Aedeagus (Kolibáč 2005) with parameres apically angulate; inner outline between parameres weakly sinuate or straight; length of parameres longer than base of tegmen. Protibial edge smooth, mucro absent; protibial spurs longer than tarsomere 2, anteriormost protibial spur greatly enlarged.

Larva (according to Kolibáč 2005): Frontal arms V-shaped. Epicranial stem reduced. Endocarina present. Mandibular apical teeth number: two, horizontally situated. Lacinia mandibulae with several small spines. Mola absent. Maxillary palpi 3-segmented. Mala: bidentate protrusion absent. Cardo: size much smaller than stipes. Ligula absent. Labial palpi 2-segmented. Prementum in single part. Antennal joints 1 and 2 elongate. Thoracic sclerites pattern (dorsally) 1-2-2. Abdominal segment IX not divided. Tergite IX flat. Urogomphi present, hooked; median process absent.

###### Biology.

The adults and larvae are probably predatory. An adult *Kolibacia squamulata* was, for example, found in rotten birch (Schawaller 1993). Mamaev (1976) remarked that *Kolibacia squamulata* develops under bark and sometimes within the trunks of various deciduous trees infested with the larvae of *Melandrya*, *Tremex fuscicornis*, *Mesosa*, *Plagionotus* and others. On the other hand, Leschen and Lackner (2013) consider both species mycophagous, citing Kryzhanovskij (1965) and Nikitsky (1992). The former source is unknown to me, but the latter author considers *Kolibacia squamulata* predatory. However, *Kolibacia squamulata* differs from *Leperina* in the presence of the mandibular mola which is definitely a feature of mycophagous cleroids, although it may also be considered a primitive (rudimentary) character, a trait seen in similar fashion in certain *Gymnocheilis* species (see figures in Kolibáč 2005).

###### Distribution.

Russian Far East, Mongolia, North Korea, North-eastern China, Japan: Hokkaido, Tsushima.

###### Species:

*Kolibacia okinawana* Yoshitomi & Lee (in press); Japan: Okinawa, Amami-Ôshima (AD)

Yoshitomi, H. & Lee, C.-F. (in press)

*Kolibacia regularis* Grouvelle, 1913; Taiwan, Vietnam (varA)

Grouvelle, A. 1913: 46 (*Leperina*). Yoshitomi, H. & Lee, C.-F. (in press; comb. *Kolibacia*, key)

*Kolibacia squamulata* Gebler, 1830 (*Peltis*); Russian Far East, Mongolia, North Korea, Northeastern China (varA, JK)

Léveillé, A. 1910: 22. Esaki, T. et al. 1951: 1061 (*Lepidopteryx squamulosa*). Kolibáč, J. 2006: 107 (*Lepidopteryx squamulosa*). Kolibáč, J. 2007a: 364. Löbl & Smetana 2010: 26 (*Lepidopteryx*). Mamaev, B. M. 1976: 1652 (larva, *Lepidopteryx*). Nakane, T. et al. 1963: 181 (*Lepidopteryx squamulosa*). Nikitsky, N. B. 1992: 81 (*Lepidopteryx squamulosa*). Leschen, R. A. B. & Lackner, T. 2013: 288. (comb. *Kolibacia*). Schawaller, W. 1993: 3 (key, *Leperina*). Yoshitomi, H. & Lee, C.-F. (in press; key)

*Kolibacia tsushimana* Nakane, 1985; Japan: Tsushima (varA)

Nakane, T. 1985: 162 (*Lepidopteryx squamulata tsushimana*). Kolibáč, J. 2009: 128 (*Lepidopteryx squamulata tsushimana*). Miyatake, M. 1985: 148 (*Leperina tsushimana*). Leschen, R. A. B. & Lackner, T. 2013: 288. (comb. *Kolibacia squamulata tsushimana*). Yoshitomi, H. & Lee, C.-F. (in press; *Kolibacia tsushimana*; key)

*Kolibacia tibialis* Reitter, 1889 (*Leperina*); Japan: Hokkaido (varA)

Léveillé, A. 1910: 22. Esaki, T. et al. 1951: 1061. Kolibáč, J. 2007a: 364. Löbl & Smetana 2010: 26 (*Lepidopteryx*). Nakane, T. et al. 1963: 181. Nikitsky, N. B. 1992: 81. Leschen, R. A. B. & Lackner, T. 2013: 288. (comb. *Kolibacia*). Yoshitomi, H. & Lee, C.-F. (in press; key)

##### Genus Leperina

Erichson, 1844

http://species-id.net/wiki/Leperina

Figs 4 , 5 ; Map 4

Leperina Erichson, W. F. 1844: 453.

###### Type species.

*Trogosita decorata* Erichson, 1842 [designated by Kolibáč 2005]

Léveillé, A. 1910: 21. Crowson, R. A. 1964a: 299. Kolibáč, J. 2005: 65 (redescription). Kolibáč, J. 2006: 111 (phylogeny). Kolibáč, J. 2007a: 364. Kolibáč, J. 2009: 127 (*Lepidopteryx*). Leschen, R. A. B. & Lackner, T. 2013: 289. Matthews, E. G. 1992: 3 (key, *Lepidopteryx*). Reitter, E. 1876: 35

*Onyschomorpha* Arrow, 1900 [Type species: *Onyschomorpha marmorata* Arrow, 1900; synonymized by Kolibáč, J. 2005: 65]

Léveillé, A. 1910: 23 (*Onyschomorpha*). Kolibáč, J. 2007a: 364 (*Onyschomorpha*, as a synonym)

###### Remarks.

There has been a lack of clarity about the names *Leperina* and *Lepidopteryx* in the last decade or so. *Leperina* tended to be used by European authors while their overseas, mainly antipodean, colleaguespreferred *Lepidopteryx*. I referred – correctly – to the genus as *Leperina* in 2005, 2006 and in *Catalogue of Palaearctic Beetles* edited by I. Löbl and A. Smetana (Kolibáč 2007a). Unfortunately, swayed by various sources, I mistakenly changed the name *Leperina* to *Lepidopteryx* in the Errata to Volume 4 of the “Catalogue” (Kolibáč 2009, Löbl and Smetana 2010).

Schawaller (1993: 2) considered *Lepidopteryx* a synonym of *Leperina* because of “*invalid description*” of the genus.

Most recently, Leschen and Lackner (2013: 289) justified the name *Leperina* in their review of the Pacific Gymnochilini thus: “[...] Gymnocheilis *Dejean, 1835 and* Lepidopteryx *Hope, 1840, are objective synonyms because they share type species (Dejean, 1835: 314 listed three species under his genus, but two are* nomina nuda*). The type species of* Lepidopteryx *and* Gymnocheilis *(*Trogositas quamosa *Gray in Griffith & Pidgeon, 1832 a synonym of* Trogositavaria *Fabricius, 1801: 151) was described from ‘Melville’s Island’, but the figure of this species matches African species of* Gymnocheilis *Dejean with split-eyes and, therefore, the taxa contained within* Gymnocheilis *are not relevant phylogenetically to true* Leperina *nor* Phanodesta *(all with normal eyes).* Gymnocheilis *Dejean, 1835 has priority of the later name* Gymnochila *Erichson, 1844 [type species:* Trogosita vestita *Griffith, 1832, by monotypy, a synonym of* Gymnochila varia *(Fabricius, 1801) according to Léveillé, 1910: 22], although Reitter (1876: 37) placed* Lepidopteryx *as a synonym of* Gymnochila*. Note that White (1846) misspelled* Gymnocheilis *as* Gymnocheila *and wrongly attributed the name to Gray.*”

###### Description

(according to Leschen and Lackner 2013). Body size: 5.5–15.6 mm. Colour of body black and red-brown, unicolorous to multicoloured. Dorsal vestiture consisting of scales. Head extending beyond anterior angles of pronotum. Frons more or less horizontal with mandibles visible dorsally. Median lobe of clypeus absent. Edge of labrum weakly emarginate or straight. Eyes entire. Gena acute. Supraocular scales present or absent. Antenna 11-segmented with loose antennal club, lengths of antennomeres II and III equal or not; antennomere XI distinctly longer than wide and circular, about as long as wide. Prothorax with lateral carinae simple, weakly or unevenly crenulate; anterior angles projecting or acute; posterior angles of prothorax angulate. Pronotal surface generally uneven, punctation uniform or not with or without median glabrous areas; centre of disc usually bearing scales. Procoxae visible in lateral view. Hypomeron with or without scales, setose or glabrous; anterior portion rugose. Length of elytra 2.5–4 × as long as pronotum or greater; disc with three simple carinae that are not beaded; sublateral keel absent; intercarinal space multipunctate; window punctures present and tuberculate; intercarinal scales of elytral disc variable, from very short and oval that may be countersunk within punctures to elongate with lengths at least 2.5 × longer than wide; lateral carina simple; epipleuron hidden in lateral view. Hind wings present, fully developed; MP3 spur present (Kukalová-Peck and Lawrence 1993). Aedeagus (Kolibáč 2005) with parameres apically rounded to acute, inner outline between parameres bisinuate, weakly sinuate, or straight, length of parameres variable, median strut acute. Protibial edge spinate, mucro absent or weakly developed, spurs longer than tarsomere 2 with anteriormost spur greatly enlarged.

A description by Kolibáč (2005) based on *Leperina decorata*: Body shape elongate. Gular sutures wide, convergent at apex. Frontoclypeal suture absent. Frons: longitudinal groove or depression absent. Cranium ventrally: tufts of long setae at sides present. Submentum: ctenidium present. Antennal groove present. Eyes: size large, dorsal. Eyes number: two. Epicranial acumination deep. Lacinial hooks absent. Galea: shape clavate. Galea: ciliate setae absent. Mediostipes-Lacinia fused together. Palpifer: outer edge even. Mandibular apical teeth number: two, horizontally situated. Mola reduced but present. Penicillus (at base) present (fine, often membranous). Pubescence above mola or cutting edge absent. Ventral furrow absent. Basal notch shallow or absent. Labrum-Cranium not fused. Epipharyngial sclerite present. Lateral tormal process: projection curved downwards, processes not connected (*Airora*). Ligula: ciliate setae absent. Ligula rigid, not retroflexed, weakly emarginate. Hypopharyngeal sclerite consisting of two separate parts. Antenna 11-segmented. Antennal club asymmetrical, sensorial fields present. Front coxal cavities externally closed, internally open. Pronotum transverse. Prepectus present. Middle coxal cavities open. Elytra: long hairs absent. Epipleuron moderate. Elytral interlocking mechanism present, carinae conspicuous. Elytral punctation regular, scales present. Wing: radial cell triangular, wedge cell present, cross vein MP3-4 present, cross vein AA1+2-3+4 present. Front tibiae: spines along side moderate. Hooked spur present. Claws: denticle absent. Parasternites number along ventrites III–VII: two. Spiculum gastrale absent. Tegmen composed of three parts.

**Figure 5.**
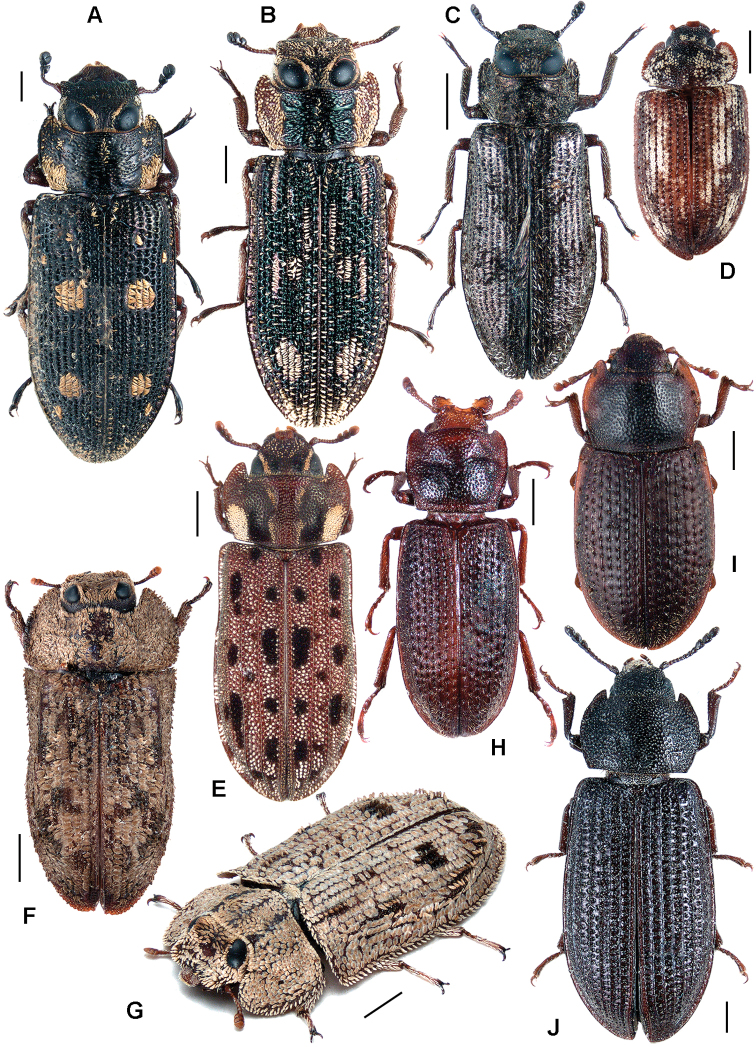
**A** *Xenoglena* sp.1, Vietnam **B** *Xenoglena* sp.2, Vietnam **C** *Xenoglena quadrisignata* (cf. *yunnanensis*) **D** *Leperina* (syn. *Onyschomorpha*) *marmorata* **E** *Leperina decorata* **F** *Narcisa decidua* **G** *Narcisa* sp., Seram **H** *Phanodesta cribraria*, “Chili” **I** *Phanodesta* sp., Juan Fernandez Isl. **J** *Seidlitzella procera*.

###### Biology.

Both larva and adult are predatory. *Leperina decorata* and *Leperina monilata* were found in stems of *Eucalyptus obliqua*, where they were preying on larvae of *Epithora dorsalis* (Bashford 1994). The latter author notes a single-year life-cycle for the *Leperina* species (referred as *Lepidopteryx*). Adults have also been reared on *Acacia dealbata* (Bashford 1991).

###### Distribution.

Australia incl. Tasmania, New Zealand, New Caledonia, New Guinea.

###### Species:

*Leperina adusta* Pascoe, 1860; Melbourne (AL)

Léveillé, A. 1910: 21

*Leperina burnettensis* MacLeay, 1871; Gayndah (AL)

Léveillé, A. 1910: 21

*Leperina cincta* Léveillé, 1889; New Zealand (AL)

Léveillé, A. 1910: 21

*Leperina cirrosa* Pascoe, 1860; Moreton Bay (AL)

Léveillé, A. 1910: 21. Hawkeswood, T. J. 1991: 159 (biology, *Lepidopteryx*). Hawkeswood, T. J. 1992: 229 (biology, *Lepidopteryx*)

*Leperina conspicua* Olliff, 1885; Australia (AL)

Léveillé, A. 1910: 21

*Leperina decorata* Erichson, 1842; Tasmania (AL)

Léveillé, A. 1910: 21. Kolibáč, J. 2005: 65 (redescription)

Léveillé, A. 1910: 21 (syn. *Leperina gayndahensis* MacLeay, 1871); (Gayndah)

*Leperina fraterna* Olliff, 1885; Australia (AL)

Léveillé, A. 1910: 21

*Leperina lacera* Pascoe, 1860; Viti Isl. (AL)

Léveillé, A. 1910: 21. Léveillé, A. 1910: 21 (syn. *Leperina signoreti* Reitter, 1876). Kukalová-Peck, J. & Lawrence, J. F. 1993: 243 (morphology of wing)

*Leperina lichenea* Fauvel, 1866; New Caledonia (AL)

Léveillé, A. 1910: 21

*Leperina lifuana* Fauvel, 1903; Lifu Isl. (AL)

Léveillé, A. 1910: 21

*Leperina loriae* Léveillé, 1893; New Guinea (AL)

Léveillé, A. 1910: 21

*Leperina marmorata* Arrow, 1900 (*Onyschomorpha*); Christmas Isl. (AL)

Léveillé, A. 1910: 23 (*Onyschomorpha* Arrow, 1900)

*Leperina mastersi* MacLeay, 1869; Gayndah (AL)

Léveillé, A. 1910: 21

*Leperina monilata* Pascoe, 1872 (= *moniliata* Blackburn, 1902); Australia (AL)

Léveillé, A. 1910: 22. Reitter, E. 1876: 31 (*Peltis*)

*Leperina opatroides* Léveillé, 1884; New Guinea (AL)

Léveillé, A. 1910: 22

*Leperina seposita* Olliff, 1885; Australia (AL)

Léveillé, A. 1910: 22

*Leperina spercheoides* Léveillé, 1878; New Caledonia (AL)

Léveillé, A. 1910: 22

*Leperina turbata* Pascoe, 1863; Australia (AL)

Léveillé, A. 1910: 22. Léveillé, A. 1910: 22 (syn. *Leperina fasciculata* Redtenbacher, 1868)

##### Genus Narcisa

Pascoe, 1863

http://species-id.net/wiki/Narcisa

Fig. 5 ; Map 4

Narcisa Pascoe, F. P. 1863: 28.

###### Type species.

*Narcisa decidua* Pascoe, 1863 [by monotypy]

Léveillé, A. 1910: 23. Kolibáč, J. 2005: 69 (redescription). Kolibáč, J. 2006: 111 (phylogeny). Reitter, E. 1876: 43

###### Remarks.

The genus is apparently related to *Anacypta* and *Xenoglena*. The body is larger than in *Anacypta* but not so slender as in *Xenoglena*, moreover perfectly covered in scales. Further to the three species described, I have also encountered some undescribed species, all of them distributed in the Indonesian islands.

###### Description.

Body size: about 7.0–9.0 mm. Body shape flat. Gular sutures narrow, subparallel at apex. Frontoclypeal suture absent. Frons: longitudinal groove or depression absent. Cranium ventrally: tufts of long setae at sides absent. Submentum: ctenidium present. Antennal groove present. Eyes: size large, dorsal. Eyes number: four. Epicranial acumination absent. Lacinial hooks absent. Galea: shape clavate. Galea: ciliate setae absent. Mediostipes-Lacinia fused together. Palpifer: outer edge even. Mandibular apical teeth number: two, vertically situated. Mola absent. Penicillus (at base) long setae. Pubescence above mola or cutting edge absent. Ventral furrow absent. Basal notch shallow or absent. Labrum-Cranium not fused. Epipharyngial sclerite present. Lateral tormal process: projection curved downwards, processes not connected (*Airora*). Ligula: ciliate setae absent. Ligula rigid, not retroflexed, weakly emarginate. Hypopharyngeal sclerite absent. Antenna 11-segmented. Antennal club asymmetrical, sensorial fields present. Front coxal cavities externally closed, internally open. Pronotum transverse. Prepectus absent. Middle coxal cavities open. Elytra: long hairs absent. Epipleuron moderate. Elytral interlocking mechanism present, carinae reduced. Elytral punctation regular, scales present. Wing: radial cell oblong (or reduced), wedge cell present, cross vein MP3-4 present, cross vein AA1+2-3+4 present. Front tibiae: spines along side reduced. Hooked spur present. Claws: denticle absent. Parasternites number along ventrites III–VII: one. Spiculum gastrale absent. Tegmen composed of three parts.

###### Biology.

Unknown, probably predatory like *Anacypta*.

###### Distribution.

Indonesia (species that remain formally undescribed are known from Sulawesi and other islands).

###### Species:

*Narcisa bimaculata* Gestro, 1879; Sumatra (AL)

Léveillé, A. 1910: 23

*Narcisa decidua* Pascoe, 1863; Batchian (AL)

Léveillé, A. 1910: 23. Kolibáč, J. 2005: 69 (redescription). Reitter, E. 1876: 43

*Narcisa lynceus* Olliff, 1883; Borneo (AL)

Léveillé, A. 1910: 23

##### Genus Phanodesta

Reitter, 1876

http://species-id.net/wiki/Phanodesta

Fig. 5 ; Map 4

Phanodesta Reitter, E. 1876: 31.

###### Type species.

*Phanodesta cordaticollis* Reitter, 1876 [designated by Kolibáč 2005]

Léveillé, A. 1910: 20. Crowson, R. A. 1964a: 299. Kolibáč, J. 2005: 77 (redescription). Kolibáč, J. 2006: 111 (phylogeny). Leschen, R. A. B. & Lackner, T. 2013: 290

###### Remarks.

As mentioned previously, six *Phanodesta* species from Juan Fernandez Island have been considered peculiar wingless beetles, with a form of elytral sculpture that is unknown in other trogossitids. Two analyses have shown a relationship for the genus within Gymnochilini, perhaps as a strongly-derived descendent of Australian *Leperina* (Kolibáč 2006, 2008). Most recently, Leschen and Lackner (2013) revised and redescribed the genus, established several new species from New Zealand, and combined some *Leperina* with *Phanodesta* species. They also discovered an uncommon distribution pattern comprising New Zealand (and adjacent islands) and Juan Fernandez Island.

###### Description

(according to Leschen and Lackner 2013). Body size: 5.4–11.5 mm. Colour of body black or red-brown. Dorsal vestiture consisting of setae, scales or both. Head extending beyond anterior angles of pronotum. Frons more or less horizontal with mandibles visible in dorsal view. Median lobe of clypeus absent. Edge of labrum weakly to strongly emarginate or straight. Eyes entire. Gena acute. Supra-ocular scales present or absent. Antenna 11-segmented with loose club; relative lengths antennomeres II and III variable; antennomere XI distinctly longer than wide and circular, about as long as wide or shorter. Prothorax with lateral carinae simple, or weakly to strongly and evenly or unevenly crenulate; anterior angles projecting or acute; posterior angles angulate. Pronotal surface even or uneven with impressions or shallow grooves; punctation uniform or not, with or without median area glabrous. Procoxae visible in lateral view. Hypomeron setose, glabrous, or bearing scales; anterior portion of hypomeron weakly to strongly rugose. Length of elytra 2.5–4 × or less than 2.5 × as long as pronotum; carinae present and usually beaded, with punctures located centrally within it, or adjacent and contacting carina; number of carinae variable, but usually 7–9; sublateral keel absent or present; intercarinal space apunctate; window punctures absent; intercarinal scales of disc never countersunk within puncture, elongate with lengths 2.5 × longer than wide or ovate to circular with lengths less than 2.5 × longer than wide; scales erect or not overlapping to strongly overlapping and adpressed; lateral carina simple; epipleuron visible or hidden in lateral view. Hind wings present and fully developed with MP3 spur present or vestigial and in the form of small buds, or absent. Aedeagus with parameres apically angulate, rounded or acute; inner outline between parameres bisinuate, weakly sinuate or straight; length of parameres and shape of median strut variable. Protibial edge smooth or crenulate; mucro absent or weakly developed; spurs longer than tarsomere 2 with anteriormost protibial spur greatly enlarged. Bursa and spermatheca bulbous, spermatheca about one fifth the size of the bursa and bearing a small tubulate spermathecal gland.

A description by Kolibáč (2005) based on *Phanodesta cribraria*: Body shape elongate. Gular sutures reduced. Frontoclypeal suture absent. Frons: longitudinal groove or depression absent. Cranium ventrally: tufts of long setae at sides present. Submentum: ctenidium absent. Antennal groove present. Eyes: size flat. Eyes number: two. Epicranial acumination deep. Lacinial hooks absent. Galea: shape clavate. Galea: ciliate setae absent. Mediostipes-Lacinia fused together. Palpifer: outer edge even. Mandibular apical teeth number: two, vertically situated. Mola reduced but present. Penicillus (at base) present (fine, often membranous). Pubescence above mola or cutting edge absent. Ventral furrow ciliate. Basal notch shallow or absent. Labrum-Cranium not fused. Epipharyngial sclerite present. Lateral tormal process: projection curved downwards, processes not connected (*Airora*). Ligula: ciliate setae absent. Ligula rigid, strongly retroflexed, weakly emarginate. Hypopharyngeal sclerite absent. Antenna 11-segmented. Antennal club asymmetrical, sensorial fields present. Front coxal cavities externally closed, internally open. Pronotum transverse. Prepectus absent. Middle coxal cavities open. Elytra: long hairs absent. Epipleuron moderate. Elytral interlocking mechanism present, carinae reduced. Elytral punctation regular, scales absent. Wings – present or absent. Front tibiae: spines along side moderate. Hooked spur present. Claws: denticle absent. Parasternites number along ventrites III–VII: two. Spiculum gastrale absent. Coxitae divided.

###### Biology.

Probably predatory. Adultsmay be collected at night on fungi and on the trunks of dead, dying and live trees, or on the ground (Leschen and Lackner 2013). Larvae of *Phanodesta* have been found in association with adults and are thought to be predatory beneath the bark of dead trees (Klimaszewski and Watt 1997). *Phanodesta nigrosparsa* is restricted to the *Phyllocladus trichomanoides* and *Agathis australis* trees, while *Phanodesta brounii* is a generalist (Kuschel 1990).

###### Distribution.

Chile: Juan Fernandez Island; New Caledonia, New Zealand mainland and offshore islands, Lord Howe Island.

###### Species:

*Phanodesta argentea* Montrouzier, 1860 (*Nitidula*); New Caledonia (AL)

Léveillé, A. 1910: 21 (*Leperina*). Leschen, R. A. B. & Lackner, T. 2013: 300 (comb. from *Leperina*; maybe conspecific with *Leperina spercheoides*)

*Phanodesta brevipennis* Reitter, 1876; Chile: Juan Fernandez Island (AL)

Léveillé, A. 1910: 20. Reitter, E. 1876: 33. Leschen, R. A. B. & Lackner, T. 2013: 300

*Phanodesta brounii* Pascoe, 1880; New Zealand (AL)

Léveillé, A. 1910: 21 (*Leperina*). Leschen, R. A. B. & Lackner, T. 2013: 291 (comb. from *Leperina*)

*Phanodesta carinata* Leschen & Lackner, 2013; New Zealand (AD)

Leschen, R. A. B. & Lackner, T. 2013: 292

*Phanodesta cribraria* Blanchard, 1851 (*Toxicum*); Chile: Juan Fernandez Island (AL)

Léveillé, A. 1910: 20 (syn. *Phanodesta cordaticollis* Reitter, 1876). Léveillé, A. 1910: 21 (syn. *Phanodesta picea* Germain, 1855). Kolibáč, J. 2005: 77 (redescription). Leschen, R. A. B. & Lackner, T. 2013: 300. Reitter, E. 1876: 32 (*Phanodesta cordaticollis* Reitter, 1876)

*Phanodesta cribrata* Germain, 1855; Chile: Juan Fernandez Island (AL)

Léveillé, A. 1910: 21 (syn. *Phanodesta angulata* Reitter, 1876). Leschen, R. A. B. & Lackner, T. 2013: 300. Reitter, E. 1876: 33 (*Phanodesta angulata*)

*Phanodesta francoisi* Léveillé, 1909; New Caledonia (AL)

Léveillé, A. 1910: 21 (*Leperina*). Leschen, R. A. B. & Lackner, T. 2013: 300 (comb. from *Leperina*; combination based on an image of the type)

*Phanodesta guerini* Montrouzier, 1860 (*Nitidula*); New Caledonia (AL)

Léveillé, A. 1910: 21 (*Leperina*). Leschen, R. A. B. & Lackner, T. 2013: 300 (comb. from *Leperina*). Reitter, E. 1876: 35 (*Phanodesta*)

*Phanodesta iohowi* Germain, 1898; Chile: Juan Fernandez Island (AL)

Léveillé, A. 1910: 21 (*Phanodesta johowi*). Leschen, R. A. B. & Lackner, T. 2013: 300

*Phanodesta manawatawhi* Leschen & Lackner, 2013; New Zealand (AD)

Leschen, R. A. B. & Lackner, T. 2013: 293

*Phanodesta oculata* Leschen & Lackner, 2013; New Zealand (AD)

Leschen, R. A. B. & Lackner, T. 2013: 295

*Phanodesta nigrosparsa* White, 1846 (*Gymnocheila*); New Zealand (AL)

Léveillé, A. 1910: 22 (*Leperina*). Klimaszewski, J. & Watt, J. C. 1997: 43 (*Lepidopteryx*). Leschen, R. A. B. & Lackner, T. 2013: 294 (comb. from *Leperina*). Reitter, E. 1876: 35 (*Phanodesta*)

*Phanodesta pubescens* Germain, 1898; Chile: Juan Fernandez Island (AL)

Léveillé, A. 1910: 21. Leschen, R. A. B. & Lackner, T. 2013: 300

*Phanodesta pudica* Olliff, 1889 (*Ostoma*); Lord Howe Island (varA)

Léveillé, A. 1910: 32 (*Ostoma* *incertae sedis*). Leschen, R. A. B. & Lackner, T. 2013: 299 (comb. from *Ostoma*)

*Phanodesta robusta* Pic, 1924; Chile: Juan Fernandez Island (varA)

Pic, M. 1924: 378

*Phanodesta shandi* Broun, 1909; New Zealand (varA)

Broun, 1909: 307 (*Leperina*). Leschen, R. A. B. & Lackner, T. 2013: 296 (comb. from *Leperina*)

*Phanodesta signoreti* Montrouzier, 1860 (*Leperina*); New Caledonia (AL)

Léveillé, A. 1910: 22 (*Leperina*). Leschen, R. A. B. & Lackner, T. 2013: 300 (comb. from *Leperina*)

*Phanodesta sobrina* White, 1846 (*Gymnocheila*); New Zealand (AL)

Léveillé, A. 1910: 22 (*Leperina*). Brookes, A. 1932: 28 (*Leperina interrupta*; syn. by Leschen, R. A. B. & Lackner, T. 2013: 296). Léveillé, A. 1910: 22 (syn. *Leperina fasciolata* Blanchard, 1853). Léveillé, A. 1910: 22 (syn. *Leperina nigro-sparsa* Blanchard, 1853; homonym with *Leperina nigrosparsa* White, 1846). Leschen, R. A. B. & Lackner, T. 2013: 296 (comb. from *Leperina*). Reitter, 1876: 35 (*Phanodesta*). Sharp, 1877: 266 (*Leperina farinosa*; syn. by Leschen, R. A. B. & Lackner, T. 2013: 296)

*Phanodesta tepaki* Leschen & Lackner, 2013; New Zealand (AD)

Leschen, R. A. B. & Lackner, T. 2013: 297

*Phanodesta variegata* Germain, 1855; Chile: Juan Fernandez Island (AL)

Léveillé, A. 1910: 21. Léveillé, A. 1910: 21. (syn. *Phanodesta costipennis* Reitter, 1876). Leschen, R. A. B. & Lackner, T. 2013: 300. Reitter, E. 1876: 34 (*Phanodesta costipennis*)

*Phanodesta wakefieldi* Sharp, 1877 (*Leperina*); New Zealand (AL)

Léveillé, A. 1910: 22 (*Leperina*). Leschen, R. A. B. & Lackner, T. 2013: 298 (comb. from *Leperina*)

##### Genus Seidlitzella

Jakobson, 1915

http://species-id.net/wiki/Seidlitzella

Fig. 5 ; Map 4

Seidlitzella Jakobson, G. G. 1915: 893.

###### Type species.

*Peltis procera* Kraatz, 1858 [by monotypy]

Kolibáč, J. 2005: 82 (redescription). Kolibáč, J. 2006: 111 (phylogeny). Kolibáč, J. 2007a: 365. Schawaller, W. 1993: 2 (synonymized with *Leperina*). Leschen, R. A. B. & Lackner, T. 2013: 279, 289

*Cymba* Seidlitz, 1875 (homonym) [type species: *Peltis procera* Kraatz, 1858; by original designation and monotypy]

Léveillé, A. 1910: 20. Kolibáč, J. 2007a: 365. Reitter, E. 1876: 30. Schawaller, W. 1993: 2

*Peltocymba* Reitter, 1920 [type species: *Peltis procera* Kraatz, 1858; by original designation and monotypy]

Kolibáč, J. 2007a: 365. Schawaller, W. 1993: 2

###### Remarks.

Schawaller (1993) synonymized *Seidlitzella* with *Leperina*. However, he based his observations on a comparison between *Seidlitzella procera* and two Palaearctic *Leperina* species, *Leperina squamosa* and *Leperina tibialis*. Australian species are often very different, although some their morphological details may also be similar (*Leperina decorata* is the type species of *Leperina*). Recently,Leschen and Lackner (2013) have established the new genus *Kolibacia* for the Palaearctic *Leperina squamosa* and *Leperina tibialis*. However, as their paper centred chiefly on *Phanodesta*, a differential diagnosis between *Seidlitzella* and *Kolibacia* was not addressed in detail. The main differences are explained in “A key to the species” of Gymnochilini, below.

###### Descripton.

Body size: 11.0–19.0 mm. Body shape elongate. Gular sutures wide, convergent at apex. Frontoclypeal suture absent. Frons: longitudinal groove or depression absent. Cranium ventrally: tufts of long setae at sides present. Submentum of males: ctenidium present. Antennal groove present. Eyes: size flat. Eyes number: two. Epicranial acumination moderate. Lacinial hooks absent. Galea: shape clavate. Galea: ciliate setae absent. Mediostipes-Lacinia not fused. Palpifer: outer edge denticulate. Mandibular apical teeth number: two, vertically situated. Mola absent. Penicillus (at base) present (fine, often membranous). Pubescence above mola or cutting edge absent. Ventral furrow absent. Basal notch shallow or absent. Labrum-Cranium not fused. Epipharyngial sclerite present. Lateral tormal process: projection curved downwards, processes not connected (*Airora*). Ligula: ciliate setae absent. Ligula rigid, weakly retroflexed, weakly emarginate. Hypopharyngeal sclerite absent. Antenna 11-segmented. Antennal club asymmetrical, sensorial fields present. Front coxal cavities externally closed, internally open. Pronotum transverse. Prepectus absent. Middle coxal cavities open. Elytra: long hairs absent. Epipleuron moderate. Elytral interlocking mechanism present, carinae conspicuous. Elytral punctation regular, scales absent. Wing: radial cell oblong (or reduced), wedge cell present or absent, cross vein MP3-4 present, cross vein AA1+2-3+4 absent. Front tibiae: spines along side moderate. Hooked spur present. Claws: denticle absent. Parasternites number along ventrites III–VII: one. Spiculum gastrale absent. Tegmen composed of two parts. Coxitae undivided.

###### Biology.

Predatory. Adults found on logs of various trees (e.g. the fir *Abies cilicia*), larvae found under pine bark (Schawaller 1993).

###### Distribution.

Greece, Cyprus, Turkey.

###### Species:

*Seidlitzella procera* Kraatz, 1858; Greece, Cyprus, Turkey (JK)

Léveillé, A. 1910: 20 (*Cymba*). Kolibáč, J. 2005: 82 (redescription). Kolibáč, J. 2007a: 365. Reitter, E. 1876: 31 (*Cymba*). Schawaller, W. 1993: 4 (larva)

##### Genus Xenoglena

Reitter, 1876

http://species-id.net/wiki/Xenoglena

Fig. 5 ; Map 4

Xenoglena Reitter, E. 1876: 40.

###### Type species.

*Xenoglena deyrollei* Reitter, 1876 [by monotypy]

Léveillé, A. 1910: 23. Kolibáč, J. 2005: 85 (redescription). Kolibáč, J. 2006: 111 (phylogeny). Kolibáč, J. 2007a: 364. Nikitsky, N. B. 1992: 81

###### Remarks.

Outer habitus resembles the jewel beetles (Buprestidae), especially those of the genus *Chrysobothris*. *Anacypta asahinai* Kono, 1938 was combined with *Xenoglena* by Kolibáč (2009). The combination is in accord with the opinion of Nikitsky (1992) and also my own independent study of the species. Nikitsky (*l.c.*) moreover suggested its synonymization with *Xenoglena quadrisignata*.

###### Description.

Body size: about 7.0–10.0 mm. Body shape elongate. Gular sutures narrow, subparallel at apex. Frontoclypeal suture absent. Frons: longitudinal groove or depression absent. Cranium ventrally: tufts of long setae at sides absent. Submentum: ctenidium present. Antennal groove present. Eyes: size large, dorsal. Eyes number: four. Epicranial acumination absent. Lacinial hooks absent. Galea: shape clavate. Galea: ciliate setae absent. Mediostipes-Lacinia fused together. Palpifer: outer edge even. Mandibular apical teeth number: two, vertically situated. Mola reduced but present. Penicillus (at base) long setae. Pubescence above mola or cutting edge absent. Ventral furrow absent. Basal notch shallow or absent. Labrum-Cranium not fused. Epipharyngial sclerite present. Lateral tormal process: projection curved downwards, processes not connected (*Airora*). Ligula: ciliate setae absent. Ligula rigid, not retroflexed, weakly emarginate. Hypopharyngeal sclerite absent. Antenna 11-segmented. Antennal club asymmetrical, sensorial fields present. Front coxal cavities externally closed, internally open. Pronotum transverse. Prepectus absent. Middle coxal cavities open. Elytra: long hairs absent. Epipleuron thin. Elytral interlocking mechanism present, carinae reduced. Elytral punctation regular, scales present. Wing: radial cell oblong (or reduced), wedge cell absent, cross vein MP3-4 present, cross vein AA1+2-3+4 absent. Front tibiae: spines along side reduced. Hooked spur present. Claws: denticle absent. Parasternites number along ventrites III–VII: one. Spiculum gastrale absent. Coxitae undivided.

###### Biology.

Predatory. Adults dwell on fallen trees and dry branches, hunting for xylophagous insects. They fly and run at great speed and appear very like some jewel beetles in body shape.

###### Distribution.

Indonesia, Malayan Peninsula, Russian Far East, Japan, northern China. A large body of material of perhaps-undescribed species is known to me from northern Laos.

###### Species:

*Xenoglena asahinai* Kono, 1938: 227 (*Acrops*); Japan (varA)

Nakane, T. et al. 1963: 181 (*Acrops*). Kolibáč, J. 2009: 128 (comb. with *Xenoglena*)

Note: maybe synonym of *Xenoglena quadrisignata*; opinion by Nikitsky 1992: 81.

*Xenoglena chrysobothroides* Léveillé, 1897; Malacca (AL)

Léveillé, A. 1910: 23

*Xenoglena deyrollei* Reitter, 1876; Java (AL)

Léveillé, A. 1910: 23. Kolibáč, J. 2005: 87 (redescription). Reitter, E. 1876: 41

*Xenoglena fryi* Léveillé, 1899; Perak (AL)

Léveillé, A. 1910: 23

*Xenoglena quadrisignata* Mannerheim, 1852; Siberia, Mongolia, Far East, Japan, North Korea, China: Northeast Territory (varA)

Léveillé, A. 1910: 22 (*Gymnochila*). Kolibáč, J. 2005: 86 (redescription). Kolibáč, J. 2007a: 364. Nikitsky, N. B. 1992: 81. Reitter, E. 1876: 40 (*Gymnochila*)

*Xenoglena tetrasigma* Léveillé, 1878; Malacca (AL)

Léveillé, A. 1910: 23

*Xenoglena vicina* Léveillé, 1897; Malacca (AL)

Léveillé, A. 1910: 23

*Xenoglena yunnanensis* Léveillé, 1907; China: Yunnan (AL)

Léveillé, A. 1910: 23. Kolibáč, J. 2007a: 364

##### Tribe Trogossitini

Latreille, 1802

Trogossitini Latreille, P. A. 1802: 110.

###### Type genus:

*Trogossita* Olivier, 1790 (= *Temnoscheila* Westwood, 1830)

Burakowski, B. et al. 1986: 115 (Nemosomatinae). Kolibáč, J. 2006: 120 (diagnosis, stat. n.). Kolibáč, J. 2007a: 364 (phylogeny). Kolibáč, J. 2008: 118–119 (phylogeny)

###### Remarks.

Two character analyses of Trogossitini (Kolibáč 2006, 2008) separate off a monophyletic group composed of the genera *Temnoscheila*, *Nemozoma*, *Tenebroides*, *Corticotomus*, and *Leipaspis*. The other two genera analyzed, *Airora* and *Alindria*, are more primitive. Their position in the cladogram of 2008 (p. 119) makes Trogossitini paraphyletic with reference to Gymnochilini but the original analysis (2006) unambiguously set the two groups appart as distinct monophyletic clades. A classification of *Seidlitzella* has been discussed above, in the “Remarks” section of the Gymnochilini entry.

There are also some genera that are not included in the two character analyses because of insufficient data sets, namely *Dupontiella*, *Elestora*, *Eupycnus*, *Euschaefferia*, and *Parallelodera*. The classification of all these rather advanced genera within Trogossitini is undeniable, apart from the monotypic *Elestora* which is obviously related to *Melambia*, for which the systematics are quite complicated and in need of revision.

Most of the members of Trogossitini lead the kind of life typical of predatory Cleridae, especially of the subfamilies Clerinae and Tillinae. Adults hunt for xylophagous insects (e.g. Curculionidae: Scolytinae, Bostrichidae) on branches and logs while larvae dwell and hunt under bark or in galleries. However, some trogossitine adults live in insect galleries together with their larvae (e.g. *Nemozoma*). The trogossitins are not as efficient in the air as the gymnochilins, and neither do they move so swiftly on the ground.

###### Key to the recent genera

**Table:** 

1	Frons with conspicuous sharp longitudinal groove	2
–	Frons without groove, with shallow depression at most	3
2	Anterior part of cranium (frons) with two large horn-like processes; body extremely elongate, small (about 3–6 mm)	*Nemozoma*
–	Anterior part of cranium (frons) without distinct processes; body not extremely elongate, larger (about 7–25 mm)	*Temnoscheila*
3	Pronotum conspicuously elongate, weakly narrowed at base; body elongate and cylindrical	4
–	Pronotum transverse or quadrate or narrowed at base; elytra widest in apical third and somewhat flattened	8
4	Pronotum somewhat cordate; elytra with carinae; large species (about 10–35 mm)	*Alindria*
–	Sides of pronotum nearly parallel; elytra without carinae; smaller species (about 2–15 mm)	5
5	Outer margins of all tibiae with large spines; antennae reach backwards anterior margin of pronotum; larger species (about 7–15 mm)	*Airora*
–	Outer margin of tibiae with 2–3 spines at apex at most; antennae reach back to beyond anterior margin of pronotum; smaller species (about 2–5 mm)	6
6	Pronotum conspicuously narrowed (constricted) at base	*Dupontiella*
–	Pronotum not narrowed at base, oblong	7
7	Pronotum with distinctly raised lateral margins; submentum distinctly separated from gula in front, outer angles not prominent; at least front tibiae with spines at apex	*Corticotomus*
–	Pronotum without distinctly raised lateral margins, apical angles obliterated; submentum not distinctly separated from gula in front, outer angles prominent and produced apically at least to base of mandibles; tibiae without spines	*Euschaefferia*
8	Elytra with conspicuous carinae and regular punctation	9
–	Elytra without carinae, with regular sculpture only	10
9	Dorsal body surface distinctly flattened; very wide, black species, elytra with four striking orange spots; mesonotum with long orange hairs	*Elestora*
–	Dorsal body surface not distinctly flattened, almost cylindrical; elongate, almost cylindrical, unicolorous (black or brown) species without colour pattern	*Melambia*
10	Body including head and pronotum distinctly elongate; pronotum constricted at base	*Leipaspis*
–	Body not so elongate; sides of pronotum subparallel or cordate	11
11	Tarsal pattern 4-4-4: 1st tarsomere coalescent with 2nd tarsomere in all pairs of legs; elytra rather convex	*Parallelodera*
–	Tarsal pattern 5-5-5; elytra rather flattened	12
12	All tibiae with about 3-6 conspicuous spines along outer margin; pronotum subparallel, elongate; labrum retracted, hardly visible; body more coarsely sculptured	*Eupycnus*
–	All tibiae with about 2–4 spines along outer margin; pronotum cordate, approximately as long as wide; labrum distinctly visible; body sculpture finer	*Tenebroides*

##### Genus Airora

Reitter, 1876

http://species-id.net/wiki/Airora

Figs 1 , 6 ; Map 5

Airora Reitter, E. 1876: 18.

###### Type species:

*Trogossita cylindrica* Serville, 1828 [designated by Barron 1971]

Léveillé, A. 1910: 7. Barron, J. R. 1971: 64. Kolibáč, J. 2005: 41 (redescription). Kolibáč, J. 2006: 111 (phylogeny)

*Temnochilodes* Léveillé, 1890 [type species: *Temnochilodes dugesi* Léveillé, 1890]

Léveillé, A. 1910: 9. Kolibáč, J. 2005: 41 (synonymized)

###### Description.

Body size: about 7.0–16.0 mm. Body shape elongate. Gular sutures narrow, subparallel at apex. Frontoclypeal suture absent. Frons: longitudinal groove or depression absent. Cranium ventrally: tufts of long setae at sides present. Submentum: ctenidium absent. Antennal groove present. Eyes: size flat. Eyes number: two. Epicranial acumination deep. Lacinial hooks absent. Galea: shape elongate. Galea: ciliate setae absent. Mediostipes-Lacinia not fused. Palpifer: outer edge denticulate. Mandibular apical teeth number: two, vertically situated. Mola reduced but present. Penicillus (at base) present (fine, often membranous). Pubescence above mola or cutting edge absent. Ventral furrow present. Basal notch moderate. Labrum-Cranium not fused. Epipharyngial sclerite present. Lateral tormal process: projection curved downwards, processes not connected (*Airora*). Ligula: ciliate setae absent. Ligula rigid, strongly retroflexed, deeply emarginate. Hypopharyngeal sclerite consisting of two separate parts. Antenna 11-segmented. Antennal club asymmetrical, sensorial fields present. Front coxal cavities externally closed, internally open. Pronotum elongate. Prepectus absent. Middle coxal cavities open. Elytra: long hairs absent. Epipleuron moderate. Elytral interlocking mechanism present, carinae reduced. Elytral punctation regular, scales absent. Wing: radial cell oblong (or reduced), wedge cell present, cross vein MP3-4 present, cross vein AA1+2-3+4 absent. Front tibiae: spines along side large. Hooked spur present. Claws: denticle absent. Parasternites number along ventrites III–VII: one. Spiculum gastrale absent. Tegmen composed of two parts. Coxitae undivided.

Larva: Frontal arms V-shaped. Epicranial stem reduced. Endocarina present. Gular sutures conspicuous, parallel. Gula: anterior apodemes absent. Paragular sclerites present. Hypostomal rods absent. Stemmata number: five. Mandibular apical teeth number: two, horizontally situated. Lacinia mandibulae absent. Mola absent. Maxillary palpi 3-segmented. Cardo: size much smaller than stipes. Labial palpi 2-segmented. Prementum in single part. Antennal joints 1 and 2 elongate. Sensory appendix very small. Thoracic sclerites pattern (dorsally) 1-2-2. Thoracic sclerites pattern (ventrally) 3+1+1. Abdominal segment IX not divided. Tergite IX flat. Urogomphi present, hooked; median process absent.

**Figure 6.**
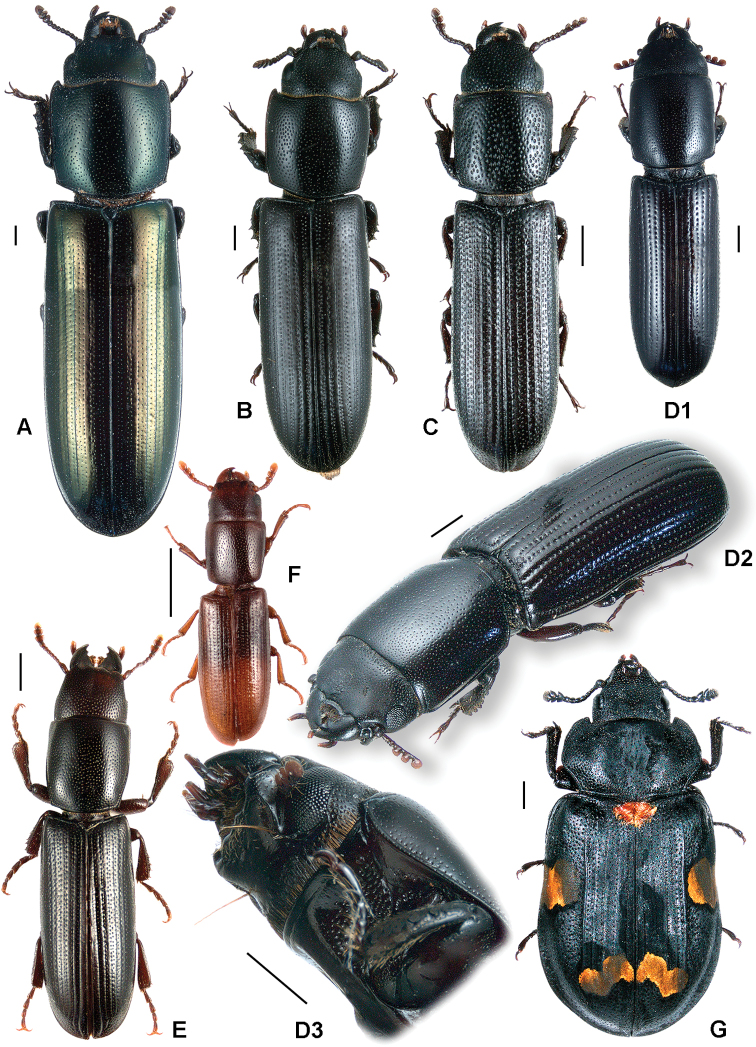
**A** *Alindria spectabilis* **B** *Alindria elongata* **C** *Alindria* sp., Thailand **D** *Airora cylindrica* **E** *Airora* (syn. *Temnochilodes*) *dugesi* **F** *Corticotomus* (syn. *Colydobius*) *divisus* **G** *Elestora fulgurata*.

###### Biology.

Predatory. In USA, adults dwell mostly on branches and logs of various species of pine. Some specimens were also collected from the bush *Cercidiumcorreyanum* and some emerged from the fungus *Fomesapplanatus*. *Airora minuta* adults were observed preying on the bark beetle *Hylocurus*. (All Barron 1971.)

###### Distribution.

From Brazil to Canada.

**Map 5.**
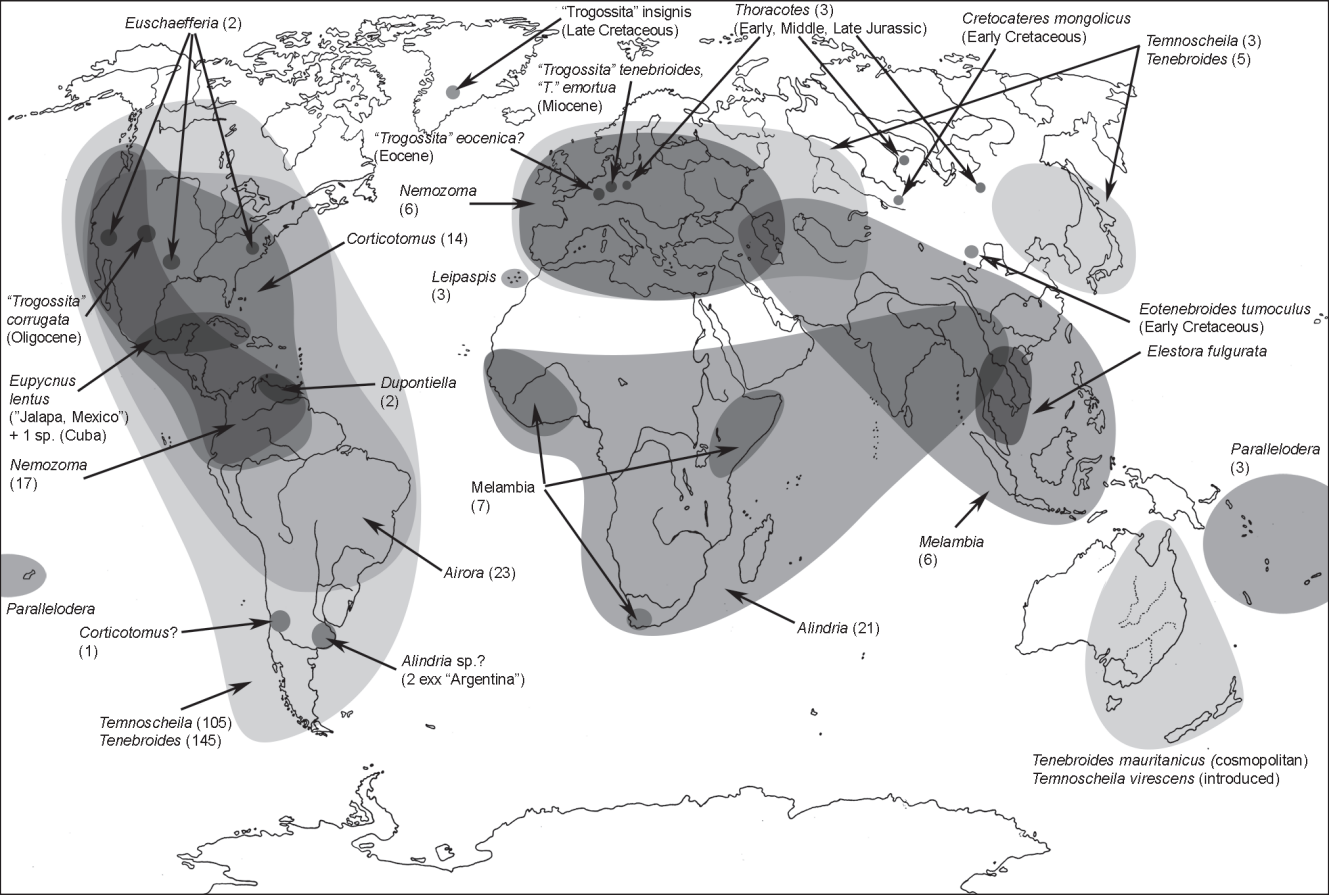
A distribution of the tribe Trogossitini.

###### Species:

*Airora aequalis* Reitter, 1876; Canada, USA, Mexico (JRB)

Barron, J. R. 1971: 65 (syn. *Airora bicolor* Casey, 1916; synonymized by Schaeffer 1920: 193?). Barron, J. R. 1971: 67 (syn. *Airora polita* Casey, 1916; synonymized by author). Barron, J. R. 1971: 67 (syn. *Airora punctiventris* Casey, 1916; synonymized by author)

*Airora apicalis* Reitter, 1876; Colombia (AL)

Léveillé, A. 1910: 7

*Airora bituberculata* Léveillé, 1905; Brazil (AL)

Léveillé, A. 1910: 7

*Airora canescens* Reitter, 1876; Central America (AL)

Léveillé, A. 1910: 7

*Airora centralis* Sharp, 1891; Mexico, Guatemala, Panama (AL)

Léveillé, A. 1910: 7

*Airora cylindrica* Serville, 1828; Canada, USA, Mexico (JRB)

Léveillé, A. 1910: 7. Barron, J. R. 1971: 65 (syn. *Airora nigellus* Melsheimer, 1846; synonymized by whom?). Barron, J. R. 1971: 65 (syn. *Airora teres* Melsheimer, 1846; synonymized by author). Böving, A. G. & Craighead, F. C. 1931: 273 (larva). Léveillé, A. 1910: 7 (*Airora teres* Melsheimer, 1846 = syn. *Airora aequalis* Reitter, 1877; synonymized by Léveillé 1910: 7). Kolibáč, J. 2005: 42 (redescription). Reitter, E. 1876: 21 (syn. *Hypophloeus teres* Melsheimer, 1846)

*Airora decipiens* Léveillé, 1899; Mexico (AL)

Léveillé, A. 1910: 7

*Airora dugesi* Léveillé, 1890; Mexico (AL)

Léveillé, A. 1910: 9 (*Temnochilodes*). Kolibáč, J. 2005: 43 (redescription, combination)

*Airora ferruginea* Léveillé, 1905; Venezuela (AL)

Léveillé, A. 1910: 7

*Airora grouvellei* Léveillé, 1889; Colombia (AL)

Léveillé, A. 1910: 7

*Airora humeralis* Léveillé, 1894; Brazil (AL)

Léveillé, A. 1910: 7

*Airora longicollis* Guérin, 1846; Central and South America (AL)

Léveillé, A. 1910: 7 (syn. *Airora clivinoides* Reitter, 1876; synonymized by author?)

*Airora mathani* Léveillé, 1878; Bolivia (AL)

Léveillé, A. 1910: 7

*Airora minuta* Schaeffer, 1918; USA: Arizona, California (JRB)

Barron, J. R. 1971: 69

*Airora modesta* Léveillé, 1907; Venezuela (AL)

Léveillé, A. 1910: 7

*Airora parallelicollis* Léveillé, 1894; Brazil, Venezuela (AL)

Léveillé, A. 1910: 7

*Airora pollens* Sharp, 1891; Mexico (AL)

Léveillé, A. 1910: 7

*Airora procera* Reitter, 1876; Bolivia, Paraguay (AL)

Léveillé, A. 1910: 7

*Airora striatopunctata* Reitter, 1876; West Indies, Brazil (AL)

Léveillé, A. 1910: 7

*Airora suturata* Léveillé, 1894; Brazil (AL)

Léveillé, A. 1910: 7

*Airora vicina* Léveillé, 1903; Brazil (AL)

Léveillé, A. 1910: 7

*Airora wagneri* Léveillé, 1907; Argentina (AL)

Léveillé, A. 1910: 8

*Airora yucatanica* Sharp, 1891; Mexico (AL)

Léveillé, A. 1910: 8

##### Genus Alindria

Erichson, 1844

http://species-id.net/wiki/Alindria

Figs 1 , 6 ; Map 5

Alindria Erichson, W. F. 1844: 451.

###### Type species.

*Trogossita grandis* Serville, 1828 [designated by Kolibáč 2005]

Léveillé, A. 1910: 8. Kolibáč, J. 2005: 43 (redescription). Kolibáč, J. 2006: 111 (phylogeny). Kolibáč, J. 2007a: 364

###### Description.

Body size: about 11.0–34.0 mm. Body shape elongate. Gular sutures reduced. Frontoclypeal suture absent. Frons: longitudinal groove or depression absent. Cranium ventrally: tufts of long setae at sides present. Submentum of males: ctenidium present. Antennal groove present. Eyes: size flat. Eyes number: two. Epicranial acumination deep. Lacinial hooks absent. Galea: shape elongate. Galea: ciliate setae absent. Mediostipes-Lacinia not fused. Palpifer: outer edge denticulate. Mandibular apical teeth number: two, horizontally situated. Mola reduced but present. Penicillus (at base) present (fine, often membranous). Pubescence above mola or cutting edge absent. Ventral furrow present. Basal notch shallow or absent. Labrum-Cranium not fused. Epipharyngial sclerite present. Lateral tormal process: projection curved downwards, processes not connected (*Airora*). Ligula: ciliate setae absent. Ligula rigid, strongly retroflexed, deeply emarginate. Hypopharyngeal sclerite consisting of two separate parts. Antenna 11-segmented. Antennal club asymmetrical, sensorial fields present. Front coxal cavities externally closed, internally open. Pronotum transverse. Prepectus absent. Middle coxal cavities open. Elytra: long hairs absent. Epipleuron moderate. Elytral interlocking mechanism present, carinae reduced. Elytral punctation regular, scales absent. Wing: radial cell triangular, wedge cell present, cross vein MP3-4 present, cross vein AA1+2-3+4 present. Front tibiae: spines along side large. Hooked spur present. Claws: denticle absent. Parasternites number along ventrites III–VII: two. Spiculum gastrale absent. Tegmen composed of two parts. Coxitae undivided.

###### Biology.

Predatory. Biology unknown; adults are sometimes collected at light.

###### Distribution.

Disjunctive distribution: most species distributed in tropical Africa and Madagascar; about four south-eastern Asian species are also congeneric. Two unidentified specimens from Argentina (Természettudományi Múzeum Budapest) may be mislabelled.

###### Species:

*Alindria alluaudi* Léveillé, 1894; Madagascar (AL)

Léveillé, A. 1910: 8

*Alindria alutacea* Murray, 1867; Old Calabar (AL)

Léveillé, A. 1910: 8

*Alindria angusta* Léveillé, 1898; Madagascar (AL)

Léveillé, A. 1910: 8

*Alindria auberti* Léveillé, 1905; China: Sichuan (AL)

Léveillé, A. 1910: 8. Kolibáč, J. 2007a: 364

*Alindria australis* Péringuey, 1885; South Africa: Cap, Transvaal (AL)

Léveillé, A. 1910: 8

*Alindria bicolor* Basilewsky, 1956; Rwanda (AD)

Basilewsky, P. 1956: 392

*Alindria bouvieri* Léveillé, 1898; Madagascar (AL)

Léveillé, A. 1910: 8

*Alindria chevrolati* Reitter, 1876; Senegal (AL)

Léveillé, A. 1910: 8

*Alindria cribrosicollis* Léveillé, 1888; Tenasserim (AL)

Léveillé, A. 1910: 8

*Alindria cyaneicornis* Fairmaire, 1887; Madagascar (AL)

Léveillé, A. 1910: 8

*Alindria docorsei* Léveillé, 1901; Madagascar (AL)

Léveillé, A. 1910: 8

*Alindria elongata* Guérin, 1846; Guinea (AL)

Léveillé, A. 1910: 8

*Alindria grandis* Serville, 1825; Senegal, Cap (varA)

Léveillé, A. 1910: 8 (syn. *Alindria cylindrica* Olivier, 1792; synonymized by author?). Kolibáč, J. 2005: 43 (redescription). Léveillé, A. 1910: 8 (syn. *Alindria ingenicula* Gistl & Bromme, 1850; synonymized by author?). Léveillé, A. 1910: 8 (syn. *Alindria major* Guérin, 1825; syn. by author?)

*Alindria lesnei* Léveillé, 1907; East Africa (AL)

Léveillé, A. 1910: 8

*Alindria orientalis* Redtenbacher, 1844; India: Kashmir (AL)

Léveillé, A. 1910: 8

Léveillé, A. 1910: 8 (syn. *Alindria parallela* Léveillé, 1889; synonymized by Kolibáč 2007a); Andamans (AL)

Kolibáč, J. 2007a: 364 (syn. *Alindria parallela* Léveillé, 1889; synonymized by author)

*Alindria ornata* Léveillé, 1898; Congo (AL)

Léveillé, A. 1910: 8

*Alindria ruandana* Basilewsky, 1956; Rwanda (AD)

Basilewsky, P. 1956: 390

*Alindria sedilloti* Léveillé, 1881; Madagascar (AL)

Léveillé, A. 1910: 8. Léveillé, A. 1910: 8 (syn. *Alindria sikorai* Kuwert, 1891; synonymized by author?)

*Alindria sericea* Léveillé, 1898; Madagascar (AL)

Léveillé, A. 1910: 8

*Alindria spectabilis* Klug, 1833; Madagascar (AL)

Léveillé, A. 1910: 8

*Alindria virescens* Léveillé, 1907; “India” (varA)

Léveillé, A. 1910: 9. Kolibáč, J. 2007a: 364

##### Genus Corticotomus

Sharp, 1891

http://species-id.net/wiki/Corticotomus

Figs 1 , 6 ; Map 5

Corticotomus Sharp, D. 1891: 390.

###### Type species.

*Corticotomus basalis* Sharp, 1891 [designated by Barron 1971]

Léveillé, A. 1910: 7. Downie, N. M. & Arnett, R. H. Jr. 1996: 936 (key). Kolibáč, J. 2005: 50 (redescription). Kolibáč, J. 2006: 111 (phylogeny). Lepesme, P. & Paulian, R. 1944: 138 (*Colydobius* Sharp, 1891) (key). Léveillé, A. 1910: 20 (*Colydobius* Sharp, 1891)

*Colydobius* Sharp, 1891 [type species: *Colydobius divisus* Sharp, 1891; designated by Kolibáč 2005]

Léveillé, A. 1910: 20. Kolibáč, J. 2005: 50 (synonymized)

*Leveillesoma* Lepesme & Paulian, 1944 [type species: *Nemosomia fulva* Léveillé, 1905; by original designation]

Lepesme, P. & Paulian, R. 1944: 139. Kolibáč, J. 2005: 50 (synonymized)

*Parafilumis* Casey, 1916 [type species: *Parafilumis estriata* Casey, 1916; by original designation and monotypy]

Barron, J. R. 1971: 53 (synonymized)

###### Description.

Body size: about 3.0–5.0 mm. Body shape elongate. Gular sutures narrow, subparallel at apex. Frontoclypeal suture absent. Frons: longitudinal groove or depression absent. Cranium ventrally: tufts of long setae at sides absent. Submentum: ctenidium absent. Antennal groove present. Eyes: size moderate. Eyes number: two. Epicranial acumination deep. Lacinial hooks absent. Galea: shape elongate. Galea: ciliate setae present. Mediostipes-Lacinia not fused. Palpifer: outer edge denticulate. Mandibular apical teeth number: two, horizontally situated. Mola reduced but present. Penicillus (at base) present (fine, often membranous). Pubescence above mola or cutting edge absent. Ventral furrow present. Basal notch shallow or absent. Labrum-Cranium not fused. Epipharyngial sclerite present. Lateral tormal process: projection curved downwards, processes not connected (*Airora*). Ligula: ciliate setae present. Ligula rigid, not retroflexed, weakly emarginate. Hypopharyngeal sclerite sickle shaped. Antenna 11-segmented. Antennal club asymmetrical, sensorial fields present. Front coxal cavities externally closed, internally open. Pronotum elongate. Prepectus absent. Middle coxal cavities open. Elytra: long hairs absent. Epipleuron moderate. Elytral interlocking mechanism present, carinae reduced. Elytral punctation regular, scales absent. Wing: radial cell open (outer vein present), wedge cell absent, cross vein MP3-4 absent, cross vein AA1+2-3+4 absent. Front tibiae: spines along side large. Hooked spur present. Claws: denticle absent. Spiculum gastrale absent. Tegmen composed of two parts. Coxitae undivided.

###### Biology.

Predatory. North American species have been both reared and collected from various species of pine and willow, also from *Rhus*, *Pseudotsuga*, and other trees (Barron 1971, Dajoz 1997). The *Corticotomus* species were mostly found under bark but also in the burrows of *Cryphalus* (Scolytinae) (Barron 1971). Dajoz (1997) noted *Corticotomus apicalis* associated with the bark beetle *Chaetophloeus parkinsoniae*. The way of life of *Corticotomus* is probably similar to that of *Nemozoma*.

###### Distribution.

Distributed from Brazil to Canada. One species, unknown to me, is described from Chile.

###### Species:

*Corticotomus apicalis* Van Dyke, 1944; Western USA (JRB)

Barron, J. R. 1971: 60. Dajoz, R. 1997: 42 (biology). Van Dyke, E. C. 1944: 151

*Corticotomus basalis* Sharp, 1891; Guatemala (AL)

Léveillé, A. 1910: 7. Kolibáč, J. 2005: 50

*Corticotomus bicolor* Léveillé, 1895; Chile (AL)

Léveillé, A. 1910: 7

*Corticotomus californicus* Van Dyke, 1915; Western USA (JRB)

Barron, J. R. 1971: 60 (syn. *Parafilumis estriata* Casey, T. L. 1916: 207, 283; synonymized by whom?). Van Dyke, E. C. 1915: 28. Schaeffer, C. F. A. 1920: 193

*Corticotomus caviceps* Fall, 1910; Western Canada and USA (JRB, varA)

Barron, J. R. 1971: 58. Dajoz, R. 1997: 41 (diagnosis). Barron, J. R. 1971: 58 (syn. *Corticotomus laeviventris* Casey, 1916; synonymized by whom?). Fall, H. C. 1910: Lepesme, P. & Paulian, R. 1944: 140 (*Nemosoma* s.str. *caviceps* Fall, 1910)

*Corticotomus cylidricus* LeConte, 1863; Eastern USA: Iowa (JRB)

Léveillé, A. 1910: 5 (*Nemosoma*). Barron, J. R. 1971: 57 (syn. *Corticotomus cylindricus* var. *texanus* Schaeffer, 1918). Schaeffer, C. F. A. 1920: 193 (*Corticotomus cylindricus* subsp. *texanus* Schaeffer, C. F. A. 1918: 192). Barron, J. R. 1971: 56. Van Dyke, E. C. 1915: 26. Kolibáč, J. 2005: 51 (redescription). Böving, A. G. & F. C. Craighead, 1931: 273 (larva). Reitter, E. 1876: 14 (*Nemosoma*). Lepesme, P. & Paulian, R. 1944: 140 (*Nemosoma* s.str.)

*Corticotomus depressus* Schaeffer, 1918; Eastern USA (JRB)

Barron, J. R. 1971: 54. Schaeffer, C. F. A. 1918: 192

*Corticotomus divisus* Sharp, 1891; Panama (AL)

Léveillé, A. 1910: 20 (*Colydobius*). Kolibáč, J. 2005: 51 (combination)

*Corticotomus dufaui* Léveillé, 1907; Guadeloupe (AL)

Léveillé, A. 1910: 20. Kolibáč, J. 2005: 52 (redescription)

*Corticotomus fulva* Léveillé, 1905; Brazil (AL)

Léveillé, A. 1910: 4 (*Nemosomia*). Kolibáč, J. 2005: 50 (combination). Lepesme, P. & Paulian, R. 1944: 139 (*Nemosomia*) (combined with *Leveillesoma*)

*Corticotomus parallelum* Melsheimer, 1844; Eastern USA (JRB)

Léveillé, A. 1910: 6 (*Nemosoma*). Barron, J. R. 1971: 55. Lepesme, P. & Paulian, R. 1944: 140 (*Nemosoma*). Reitter, E. 1876: 14 (*Nemosoma*)

*Corticotomus quadrimaculatus* Léveillé, 1894; Brazil (AL)

Léveillé, A. 1910: 7

*Corticotomus sharpi* Léveillé, 1905; Mexico (AL)

Léveillé, A. 1910: 7

*Corticotomus signatus* Sharp, 1891; Guatemala (AL)

Léveillé, A. 1910: 20

*Corticotomus testaceus* Dajoz, 1997; USA: Arizona (AD)

Dajoz, R. 1997: 40

##### † Genus Cretocateres

Ponomarenko, 1986

http://species-id.net/wiki/Cretocateres

Fig. 19 ; Map 5

Cretocateres Ponomarenko, A. G. 1986: iii (1–101).

###### Type species.

*Cretocateres mongolicus* Ponomarenko, 1986 [by monotypy and author’s designation]

Kolibáč, J. 2006: 116 (classification). Ponomarenko, A. G. 1986: iii (1–101) (Lophocateridae). Ponomarenko, A. G. 1990: 73, 74. Kolibáč, J. 2006: 116 (Trogossitidae: Trogossitini). Ponomarenko, A. G. & Kireichuk, A. G. (2005–2008): http://www.zin.ru/animalia/Coleoptera/rus/paleosy2.htm (Peltidae). Schmied, H. et al. 2009: 24

###### Diagnosis.

Body size: probably about 3.0 mm (no scale provided). Body elongate, terminal antennomeres conspicuously asymmetrical. Very similar to Siberian *Thoracotes* (see Ponomarenko 1990). Differences between the two genera are not known to me. Structure of coxae in combination with characteristic antennae allow a classification within Trogossitidae: Trogossitinae; shape of pronotum is closer to Trogossitini than Gymnochilini. See a reproduction of the original table in Fig. 19.

###### Distribution.

Mesozoic: Lower Cretaceous; West Mongolia.

###### Species:

† *Cretocateres mongolicus* Ponomarenko, 1986; W Mongolia; Lower Cretaceous (varA)

Ponomarenko, A. G. 1986: iii (1–101) (Lophocateridae). Ponomarenko, A. G. 1990: 73, 74. Kolibáč, J. 2006: 116 (Trogossitidae: Trogossitini). Ponomarenko, A. G. & Kireichuk, A. G. (2005–2008): http://www.zin.ru/animalia/Coleoptera/rus/paleosy2.htm (Peltidae). Schmied, H. et al. 2009: 24

##### Genus Dupontiella

Spinola, 1844

http://species-id.net/wiki/Dupontiella

Figs 20 –23 ; Map 5

Dupontiella Spinola, M. 1844 (II): 168.

###### Type species.

*Dupontiella ichneumonoides* Spinola, 1844 [designated by Kolibáč 2005]

Léveillé, A. 1910: 6. Kolibáč, J. 2005: 54. Kolibáč, J. 2006: 116 (phylogeny). Reitter, E. 1876: 15

###### Remarks.

Body size: about 4.0–5.0 mm. The genus was originally described in Cleridae. Lacordaire (1857: 493) transferred *Dupontiella* to Trogossitidae. Neither species was pinned in Cleridae in Spinola’s original collection and I found none in “collectio Chevrolat” (MNHN Paris) where *Dupontiella ichneumonoides* could have been placed. Figures 20–23 show a facsimile of Spinola’s (1844) original descriptions with figures as well as Reitter’s (1876) remarks on the genus.

The 11-segmented antennae with distinct 3-segmented asymmetrical club, as well as perhaps a tri-sinuate anterior margin to the frons, probably indicate proper classification within Trogossitinae, perhaps related to *Nemozoma* and allied genera. Spinola’s note *“*[...] *les mandibles de la forme ordinaire, la face antérieurement arrondie et non tri-échancrée*[...]” could mean that the mandibles are unidentate. Some species, possibly only specimens of *Corticotomus*, are the only known trogossitids with mandibles that may be unidentate (Kolibáč 2005: 51). On the other hand, the body shape and the complex colour pattern on the dorsal surface of of the body also resemble *Calanthosoma* (Egoliini). Because species of the latter genus are far larger, I tend to support a relationship to *Nemozoma* and *Corticotomus*. Hence the classification of *Dupontiella* within Trogossitini herein.

###### Distribution.

Venezuela.

###### Species:

*Dupontiella fasciatella* Spinola, 1844; Venezuela: Caracas (AL)

Léveillé, A. 1910: 6. Reitter, E. 1876: 16

*Dupontiella ichneumonoides* Spinola, 1844; Venezuela: Caracas (AL)

Léveillé, A. 1910: 6. Kolibáč, J. 2005: 54. Reitter, E. 1876: 15

##### Genus Elestora

Pascoe, 1868

http://species-id.net/wiki/Elestora

Fig. 6 ; Map 5

Elestora Pascoe, F. P. 1868: XI.

###### Type species.

*Elestora fulgurata* Pascoe, 1868 [by monotypy]

Léveillé, A. 1910: 20. Kolibáč, J. 2005: 55. Kolibáč, J. 2006: 116 (phylogeny). Reitter, E. 1876: 30

###### Remarks.

The phylogeny of the genus is unclear and in need of revision, together with *Melambia*. *Elestora* could be related to some species of the latter genus (see “Remarks” on *Melambia*). I presume a classification of *Elestora* within the trogossitins rather than with the gymnochilins.

###### Description.

Body size: 15.0 mm. Body shape flat. Gular sutures wide, convergent at apex. Frontoclypeal suture absent. Frons: longitudinal groove or depression absent. Cranium ventrally: tufts of long setae at sides absent. Submentum of males: ctenidium present. Antennal groove present. Eyes: size flat. Eyes number: two. Mandibular apical teeth number: one. Labrum-Cranium not fused. Antenna 11-segmented. Antennal club asymmetrical, sensorial fields present. Front coxal cavities externally closed, internally open. Pronotum transverse. Prepectus absent. Middle coxal cavities open. Elytra: long hairs absent. Epipleuron moderate. Elytral interlocking mechanism present, carinae conspicuous. Elytral punctation regular, scales absent. Wing: radial cell oblong (or reduced), wedge cell present, cross vein MP3-4 present, cross vein AA1+2-3+4 present. Front tibiae: spines along side moderate. Hooked spur absent in middle and hind tibiae. Claws: denticle absent.

###### Biology.

Probably predatory. A specimen was observed on a smouldering log at the margin of a tropical forest clearing in Laos. Its light orange spots on the elytra (much duller in a few museum specimens) perfectly imitate wood embers. Very rapid flier.

###### Distribution.

Distributed from Malaysia to northern Laos but perhaps very rare.

###### Species:

*Elestora fulgurata* Pascoe, 1868; Malaysia: Penang, Laos (varA)

Léveillé, A. 1910: 20. Kolibáč, J. 2005: 55. Kolibáč, J. 2006: 152. Reitter, E. 1876: 30

##### † Genus Eotenebroides

Ren, 1995

http://species-id.net/wiki/Eotenebroides

Map 5

Tenebroidinae Ren, D. 1995: 88 [in Chinese], 189 [in English] ( , = Trogossitidae, Trogossitinae).

###### Type species.

*Eotenebroides tumoculus* Ren, 1995

Ren, D. 1995: 89 [species description in Chinese only]. Kolibáč, J. & Huang, D.-Y. 2008: 141 (Trogossitini)

###### English description of the genus.

“*Head triangular, longer than wide. Eyes distinct, larger, situated laterally at median. Last 3 segments of antennae not thickened. Basal part of lateral margin of pronotum not narrowed. Scutellum larger. Coxa of middle and hind legs very close. Elytra not longer than abdomen, surface with 5 visible longitudinal streaks. Length 6.1 mm, width 2.4 mm.*” (Ren 1995: 189).

###### Translation of Chinese description of the species.

“*Head: head slightly longer than wide; mandible robust, slightly curved; antenna with 11 segments, scapus a littler longer than others, length of other segments similar to one another, last three segments not thickened; eyes large, around 1/3 as long as head (excluding mandible), not projecting, clearly separated from front edge of pronotum. Thorax: pronotum longer than wide, anterior edge concave, anterio-lateral angles projecting, sharp, posterio-lateral angles rounded, posterior edge straight, as long as anterior edge; connection of pronotum with elytra forming a neck; scutellum large, triangular; metepisternum visible, not making contact with mid-coxa; front coxae transverse, well-separated, femur thick; middle coxae rounded, close to one another, femur thicker than tibia; hind coxae nearly rounded, close to one another, hind femur as wide as middle femur. Elytra: elytra does not reach end of abdomen, its base as wide as pronotum, equipped with 5 longitudinal ridges. Abdomen: 6 sternites visible.*” (Ren 1995: 89).

###### Distribution.

China: SW of Beijing, Chongqing reservoir; Mesozoic: Lower Cretaceous, Lushangfen formation.

###### Species:

† *Eotenebroides tumoculus* Ren, 1995; China: SW of Beijing; Lower Cretaceous (AD)

Ren, D. 1995: 88 [species description in Chinese only]. Kolibáč, J. & Huang, D.-Y. 2008: 141

##### Genus Eupycnus

Sharp, 1891

http://species-id.net/wiki/Eupycnus

Fig. 7 ; Map 5

Eupycnus Sharp, D. 1891: 415.

###### Type species.

*Eupycnus lentus* Sharp, 1891 [by monotypy]

Léveillé, A. 1910: 14. Kolibáč, J. 2005: 56 (redescription). Kolibáč, J. 2006: 116 (phylogeny)

###### Description.

Body size: about 7.5 mm. Body shape elongate. Gular sutures wide, convergent at apex. Frons: longitudinal groove or depression absent. Cranium ventrally: tufts of long setae at sides present. Antennal groove present. Eyes: size flat. Eyes number: two. Epicranial acumination moderate. Mandibular apical teeth number: two, vertically situated. Labrum-Cranium not fused. Ligula: ciliate setae present. Ligula rigid, not retroflexed, weakly emarginate. Hypopharyngeal sclerite consisting of two separate parts. Antenna 11-segmented. Antennal club asymmetrical, sensorial fields present. Front coxal cavities externally closed, internally open. Pronotum subquadrate. Prepectus absent. Middle coxal cavities open. Elytra: long hairs absent. Epipleuron moderate. Elytral interlocking mechanism present, carinae reduced. Elytral punctation regular, scales absent. Wing: radial cell open (outer vein present), wedge cell absent, cross vein MP3-4 absent, cross vein AA1+2-3+4 absent. Front tibiae: spines along side large. Hooked spur present in all tibiae. Claws: denticle absent. Parasternites number along ventrites III–VII: one. Spiculum gastrale present. Tegmen composed of three parts.

**Figure 7.**
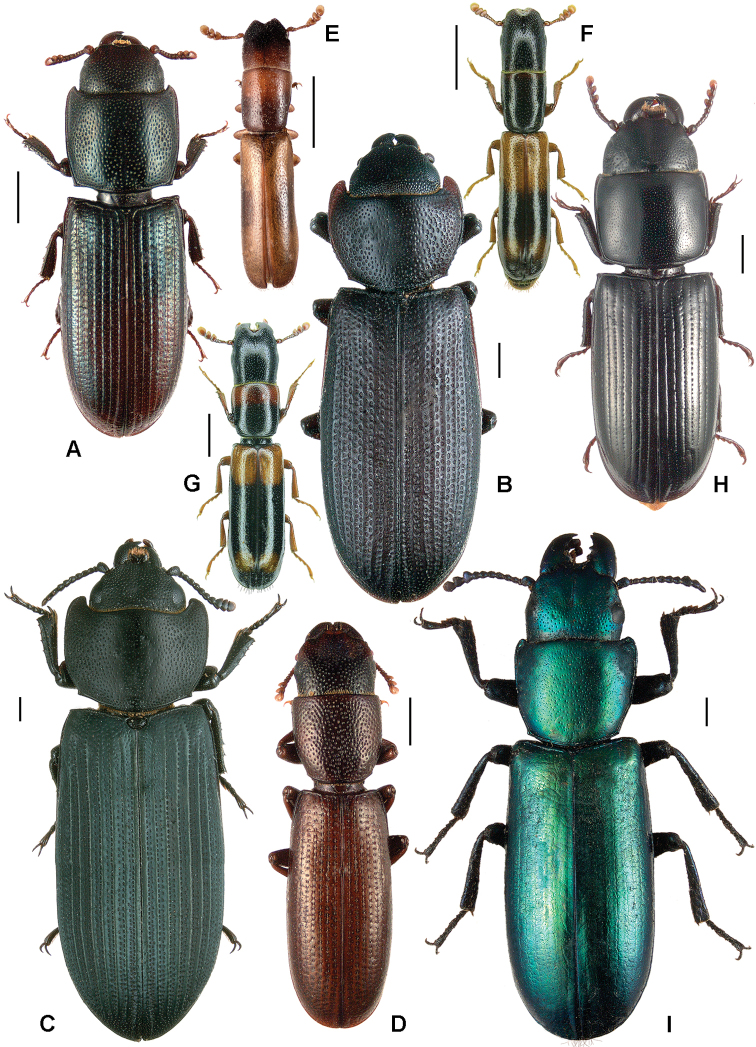
**A** *Eupycnus lentus* **B** *Melambia crenicollis* **C** *Melambia striata* **D** *Leipaspis lauricola* **E** *Nemozoma cornutum* **F** *Nemozoma elongatum* **G** *Nemozoma caucasicum* **H** *Parallelodera quadraticollis* **I** *Temnoscheila pini*.

###### Biology.

Predatory. The single described species probably has the same way of life as *Tenebroides* species.

###### Distribution.

Mexico. One undescribed species is known to me from Cuba.

###### Species:

*Eupycnus lentus* Sharp, 1891; Mexico (AL)

Léveillé, A. 1910: 14. Kolibáč, J. 2005: 56 (redescription)

##### Genus Euschaefferia

Leng, 1920

http://species-id.net/wiki/Euschaefferia

Map 5

Euschaefferia Leng, C. W. 1920: 193.

###### Type species.

*Stenodema hicoriae* Schaeffer, 1918 [by original designation and monotypy]

Barron, J. R. 1971: 62. Kolibáč, J. 2005: 57. Kolibáč, J. 2006: 116 (phylogeny)

*Pseudocotomus* Van Dyke, 1944 [type species: *Pseudocotomus mclayi* Van Dyke, 1944; by original designation]

Van Dyke, E. C. 1944: 153. Barron, J. R. 1971: 62 (synonymized)

*Stenodema* Schaeffer, 1918 (preoccupied) [type species: *Stenodema hicoriae* Schaeffer, 1918; by original designation]

Barron, J. R. 1971: 62. Leng, C. W. 1920: 193. Schaeffer, C. F. A. 1918: 193

###### Description.

Body size: 2.0–3.0 mm. Body shape elongate. Frons: longitudinal groove or depression absent. Labrum-Cranium not fused. Antenna 11-segmented. Antennal club asymmetrical. Pronotum elongate. Elytra: long hairs absent, carinae reduced. Elytral punctation irregular, scales absent. Front tibiae: spines along side reduced. Hooked spur absent in middle and hind tibiae. Claws: denticle absent.

###### Biology.

Predatory. It was recorded from the galleries of the bark beetle *Pseudothysanoes burtoni* and has also been reared on the plant *Vachellia farnesiana* (Barron 1971).

###### Distribution.

Belt of the southern states of the USA.

###### Species:

*Euschaefferia hicoriae* Schaeffer, 1918; USA: Texas, North Carolina (JRB)

Barron, J. R. 1971: 63. Kolibáč, J. 2005: 57. Schaeffer, C. F. A. 1918: 193

*Euschaefferia mclayi* Van Dyke, 1944; USA: California (JRB)

Barron, J. R. 1971: 63 (comb). Van Dyke, E. C. 1944: 153. (*Pseudocotomus*)

##### Genus Leipaspis

Wollaston, 1862

http://species-id.net/wiki/Leipaspis

Figs 1 , 7 ; Map 5

Leipaspis Wollaston, T. V. 1862: 140.

###### Type species.

*Leipaspis lauricola* Wollaston, 1862 [designated by Kolibáč 2005]

Léveillé, A. 1910: 14 (*Lipaspis*). Kolibáč, J. 2005: 63 (redescription). Kolibáč, J. 2006: 111 (phylogeny). Kolibáč, J. 2007a: 364. Reitter, E. 1876: 27 (*Lipaspis*)

###### Description.

Body size: about 8.5–9.5 mm. Body shape elongate. Gular sutures wide, convergent at apex. Frontoclypeal suture absent. Frons: longitudinal groove or depression absent. Cranium ventrally: tufts of long setae at sides absent. Submentum: ctenidium absent. Antennal groove present. Eyes: size flat. Eyes number: two. Epicranial acumination moderate. Lacinial hooks absent. Galea: shape elongate. Galea: ciliate setae present. Mediostipes-Lacinia not fused. Palpifer: outer edge denticulate. Mandibular apical teeth number: two, horizontally situated. Mola absent. Penicillus (at base) present (fine, often membranous). Pubescence above mola or cutting edge absent. Ventral furrow present. Basal notch shallow or absent. Labrum-Cranium not fused. Epipharyngial sclerite present. Lateral tormal process: projection curved downwards, processes not connected (*Airora*). Ligula: ciliate setae present. Ligula rigid, not retroflexed, deeply emarginate. Hypopharyngeal sclerite sickle shaped. Antenna 11-segmented. Antennal club asymmetrical, sensorial fields present. Front coxal cavities externally closed, internally open. Pronotum elongate. Prepectus absent. Middle coxal cavities open. Elytra: long hairs absent. Epipleuron moderate. Elytral interlocking mechanism present, carinae reduced. Elytral punctation regular, scales absent. Front tibiae: spines along side reduced. Hooked spur absent, apical spurs not hooked or weakly hooked. Claws: denticle absent. Parasternites number along ventrites III–VII: one. Spiculum gastrale absent. Coxitae divided.

###### Biology.

Predatory. The species was collected on the stems of various plants, such as *Euphorbia* sp., *Pinus* sp., *Suadea vermiculata*, *Arthrocnemum fruticosum*, *Laurus nobilis*, and *Nicotiana glauca*, probably preying on anobiids (*Xestobium*) or other xylophagous insects.

###### Distribution.

Canary Islands.

###### Species:

*Leipaspis caulicola* Wollaston, 1862;

Léveillé, A. 1910: 14 (*Lipaspis*). Plata-Negrache, P. & Prendes-Ayala, C. 1981: 227. Reitter, E. 1876: 27 (*Lipaspis*)

*Leipaspis caulicola caulicola* Wollaston, 1862; Canary Islands (varA)

Kolibáč, J. 2007a: 364

*Leipaspis caulicola oceanica* Wollaston, 1865; Madeira: Selvagens (varA)

Erber, D. & Wheater, C. P. 1987: 166 (*Leipaspis caulicola* var. *oceanica* Wollaston, 1865). Kolibáč, J. 2007a: 364 (subspecies)

*Leipaspis lauricola* Wollaston, 1862; Canary Islands (varA)

Léveillé, A. 1910: 14 (*Lipaspis*). Kolibáč, J. 2005: 63. Reitter, E. 1876: 27 (*Lipaspis*)

*Leipaspis lauricola lauricola* Wollaston, 1862; Canary Isl: La Palma, Tenerife (varA)

Plata-Negrache, P. & C. Prendes-Ayala 1981: 229. Kolibáč, J. 2007a: 364

*Leipaspis lauricola gomerensis* Plata & Prendes, 1981 Canary Isl.: La Gomera, El Hierro (varA)

Kolibáč, J. 2007a: 364. Machado, A. & Oromí, P. 2000: ii. Plata-Negrache, P. & Prendes-Ayala, C. 1981: 229

*Leipaspis pinicola* Wollaston, 1862; Canary Islands (varA)

Léveillé, A. 1910: 14 (*Lipaspis*). Kolibáč, J. 2007a: 364. Plata-Negrache, P. & Prendes-Ayala, C. 1981: 229. Reitter, E. 1876: 27

##### Genus Melambia

Erichson, 1844

http://species-id.net/wiki/Melambia

Fig. 7 ; Map 5

Melambia Erichson, W. F. 1844: 450.

###### Type species.

*Trogossita gigas* Fabricius, 1798 [designated by Kolibáč 2005]

Léveillé, A. 1910: 9. Kolibáč, J. 2005: 68 (redescription). Kolibáč, J. 2006: 111 (phylogeny). Kolibáč, J. 2007a: 364. Mamaev, B. M. 1976: 1652 (larva). Reitter, E. 1876: 24

###### Remarks.

The placement of the genus in Trogossitini should be revised because my analysis of 2008 disclosed a possible relationship of some its species with Gymnochilini. There are distinct differences in the body shape among the numerous species of *Melambia*, for example between *Melambia grandis* (a robust species with a cordate pronotum) and *Melambia orientalis* (an elongate species with pronotum shaped somewhat like that of *Tenebroides*). Consideration of a re-classification of *Seidlitzella* within Gymnochilini, similar in habitus to *Melambia*, needs species revision and new phylogenetic analysis with reference to Trogossitini and Gymnochilini, including special attention to the related trogossitine genus *Alindria*. As a preliminary opinion, I assume that both genera, *Melambia* and *Alindria*, form a basal group of Trogossitini.

###### Description.

Body size: about 20.0–30.0 mm. Body shape elongate. Gular sutures reduced. Frontoclypeal suture absent. Frons: longitudinal groove or depression absent. Cranium ventrally: tufts of long setae at sides absent. Submentum of males: ctenidium present. Antennal groove present. Eyes: size flat. Eyes number: two. Epicranial acumination moderate. Lacinial hooks absent. Galea: shape clavate. Galea: ciliate setae absent. Mediostipes-Lacinia fused together. Palpifer: outer edge denticulate. Mandibular apical teeth number: two, vertically situated. Mola absent. Penicillus (at base) present (fine, often membranous). Pubescence above mola or cutting edge absent. Ventral furrow present. Basal notch moderate. Labrum-Cranium not fused. Epipharyngial sclerite present. Lateral tormal process: projection curved downwards, processes not connected (*Airora*). Ligula: ciliate setae absent. Ligula rigid, weakly retroflexed, deeply emarginate. Hypopharyngeal sclerite absent. Antenna 11-segmented. Antennal club asymmetrical, sensorial fields present. Front coxal cavities externally closed, internally open. Pronotum transverse. Prepectus absent. Middle coxal cavities open. Elytra: long hairs absent. Epipleuron thin. Elytral interlocking mechanism present, carinae conspicuous. Elytral punctation regular, scales absent. Wing: radial cell oblong (or reduced), wedge cell present, cross vein MP3-4 present, cross vein AA1+2-3+4 absent. Front tibiae: spines along side moderate. Hooked spur present. Claws: denticle absent. Parasternites number along ventrites III–VII: two. Spiculum gastrale absent. Tegmen composed of two or three parts.

Larva: Frontal arms V-shaped. Epicranial stem present. Endocarina present. Stemmata number: five. Maxillary palpi 3-segmented. Palpifer absent. Mala simple. Mala: bidentate protrusion absent. Cardo-Stipes not fused. Cardo: size much smaller than stipes. Ligula absent. Labial palpi 2-segmented. Prementum in single part, anterior margin with notch. Thoracic sclerites pattern (dorsally) 2-0-0. Abdominal segment IX not divided. Tergite IX flat. Urogomphi minute; median process absent.

###### Biology.

Predatory. According to Mamaev (1976), *Melambia tekkensis* and *Melambia cardoni* larvae prey on larvae of jewel beetles and longhorn beetles (under the bark of, for example, apricot trees or *Grewia*).

###### Distribution.

South-eastern, southern and central Asia; also several species in Africa from Egypt to South Africa. Such a disjunctive distribution is possible, but the African species need to be checked because of possible confusion with the similar genus *Alindria*.

###### Species:

*Melambia cardoni* Léveillé, 1908; India: “Bengalia” (AL)

Léveillé, A. 1910: 9

*Melambia cordicollis* Reitter, 1876; Philippines (AL)

Léveillé, A. 1910: 9. Reitter, E. 1876: 25

*Melambia crenicollis* Guérin, 1846; India: “Bengalia” (AL)

Léveillé, A. 1910: 9

*Melambia funebris* Pascoe, 1862; Cambodia (AL)

Léveillé, A. 1910: 9. Reitter, E. 1876: 25

*Melambia gautardi* Tournier, 1872; Egypt (AL)

Léveillé, A. 1910: 9. Kolibáč, J. 2007a: 364. Reitter, E. 1876: 26

*Melambia gigas* Fabricius, 1798; Guinea, Senegal (AL)

Léveillé, A. 1910: 9. Kolibáč, J. 2005: 68 (redescription). Reitter, E. 1876: 25

*Melambia maura* Pascoe, 1862; South Africa: “N’gami”, Mauritania (varA)

Léveillé, A. 1910: 9. Mateu, J. 1972: 547

*Melambia memnonia* Pascoe, 1862; Sri Lanka (AL)

Léveillé, A. 1910: 9

*Melambia opaca* Reitter, 1876; South Africa: Cap (AL)

Léveillé, A. 1910: 9. Reitter, E. 1876: 25

*Melambia pumila* Léveillé, 1885; Burma (AL)

Léveillé, A. 1910: 9

*Melambia striata* Olivier, 1790; Senegal (varA)

Léveillé, A. 1910: 9. Mateu, J. 1972: 547. Reitter, E. 1876: 24

*Melambia subcyanea* Gerstaecker, 1871; Zanzibar (AL)

Léveillé, A. 1910: 9. Reitter, E. 1876: 26

Léveillé, A. 1910: 9 (syn. *Melambia coeruleata* Fairmaire, 1882) Somalia (AL)

*Melambia tekkensis* Koenig, 1889; „Transcaspia”, Turkmenistan (varA)

Léveillé, A. 1910: 9. Kolibáč, J. 2007a: 364. Mamaev, B. M. 1976: 1650 (larva)

##### Genus Nemozoma

Latreille, 1804

http://species-id.net/wiki/Nemozoma

Figs 7 , 15 ; Map 5

Nemozoma Latreille, P. A. 1804: 239.

###### Type species.

*Dermestes elongatus* Linnaeus, 1760 [by monotypy]

Léveillé, A. 1910: 5. Barron, J. R. 1971: 45. Crowson, R. A. 1964a: 299. Kolibáč, J. 2005: 72 (redescription). Kolibáč, J. 2006: 111 (phylogeny). Kolibáč, J. 2007a: 364. Kolibáč, J. et al. 2005: 31+135 (key). Lepesme, P. & Paulian, R. 1944: 139 (subgen. *Nemosoma* s.str.). Lucht, W. 1998: 207 (key). Mamaev, B. M. 1976: 1652 (larvae). Nikitsky, N. B. 1974: 563. Reitter, E. 1876: 13

*Aponemosoma* Lepesme & Paulian, 1944 (subgenus) [type species: *Nemosoma caucasicum* Ménétries, 1832; by original designation]

Barron, J. R. 1971: 45 (synonymized). Kolibáč, J. 2007a: 364. Lepesme, P. & Paulian, R. 1944: 139 (*Nemosoma* subgen. *Aponemosoma*)

*Cylidrella* Sharp, 1891 [type species: *Cylidrellamollis* Sharp, 1891; by monotypy]

Léveillé, A. 1910: 6. Barron, J. R. 1971: 52. Kolibáč, J. 2005: 72 (synonymized). Kolibáč, J. 2007a: 364

*Monesoma* Léveillé, 1894 (subgenus) [type species: *Nemosoma cornutum* Sturm, 1826; by original designation]

Léveillé, A. 1910: 6 (subgenus). Barron, J. R. 1971: 45 (=*Sturmia* Ragusa, 1892). Kolibáč, J. 2007a: 364. Lepesme, P. & Paulian, R. 1944: 139 (synonymized)

*Nemosomia* Reitter, 1876 [type species: *Nemosomia vorax* Reitter, 1876; by original designation]

Léveillé, A. 1910: 4. Barron, J. R. 1971: 45. Kolibáč, J. 2007a: 364. Lepesme, P. & Paulian, R. 1944: 139 (subgenus). Reitter, E. 1876: 11

*Paranemosoma* Lepesme & Paulian, 1944 (subgenus) [type species: *Nemosoma punctatum* Léveillé, 1886; by original designation]

Lepesme, P. & Paulian, R. 1944: 139. Kolibáč, J. 2007a: 364

*Sturmia* Ragusa, 1892 [type species: *Nemosoma cornutum* Sturm, 1826; by original designation]

Barron, J. R. 1971: 45 (preoccupied, synonymized with *Monesoma*). Kolibáč, J. 2007a: 364

*Pseudalindria* Fall, 1910 [type species: *Pseudalindria fissiceps* Fall, 1910; by original designation]

Barron, J. R. 1971: 45. Fall, H. C. 1910: 126. Kolibáč, J. 2007a: 364

###### Description.

Body size: about 2.5–5.0 mm. Body shape elongate. Gular sutures wide, convergent at apex. Frontoclypeal suture absent. Frons: longitudinal groove or depression present. Cranium ventrally: tufts of long setae at sides present. Submentum: ctenidium absent. Antennal groove present. Eyes: size flat. Eyes number: two. Epicranial acumination moderate. Lacinial hooks absent. Galea: shape elongate. Galea: ciliate setae present. Mediostipes-Lacinia fused together. Palpifer: outer edge even. Mandibular apical teeth number: two, vertically situated. Mola absent. Penicillus (at base) absent. Pubescence above mola or cutting edge absent. Ventral furrow present. Basal notch moderate. Labrum-Cranium not fused. Epipharyngial sclerite present. Lateral tormal process: projection projection not developed (all remaining). Ligula: ciliate setae present. Ligula membranous, not retroflexed, deeply emarginate. Hypopharyngeal sclerite absent. Antenna 11-segmented or 10-segmented. Antennal club asymmetrical, sensorial fields present. Front coxal cavities externally closed, internally open. Pronotum elongate. Prepectus absent. Middle coxal cavities open. Elytra: long hairs absent. Epipleuron thin. Elytral interlocking mechanism present, carinae reduced. Elytral punctation regular, scales absent. Wing: radial cell open (outer vein present), wedge cell absent, cross vein MP3-4 present, cross vein AA1+2-3+4 absent. Front tibiae: spines along side large. Hooked spur present. Claws: denticle absent. Parasternites number along ventrites III–VII: one. Spiculum gastrale absent. Tegmen composed of three parts. Coxitae undivided.

Larva: Frontal arms V-shaped. Epicranial stem reduced. Endocarina present. Gular sutures conspicuous, parallel. Gula: anterior apodemes absent. Paragular sclerites present. Stemmata number: five. Mandibular apical teeth number: one tooth. Lacinia mandibulae with several small spines. Mola absent. Maxillary palpi 3-segmented. Palpifer absent. Pedunculate seta present. Mala simple. Mala: bidentate protrusion absent. Cardo: size much smaller than stipes. Ligula absent. Labial palpi 2-segmented. Prementum in single part, anterior margin with notch. Antennal joints 1 and 2 elongate. Sensory appendix medium sized (to half of joint 3). Thoracic sclerites pattern (dorsally) 1-2-2. Thoracic sclerites pattern (ventrally) 3+1+1. Trochanter triangular. Abdominal segment IX not divided. Tergite IX flat. Urogomphi present, hooked; median process absent.

###### Biology.

Predatory. Adults live together with larvae under the bark of deciduous and coniferous trees and shrubs, hunting especially for bark beetles.

###### Distribution.

From Brazil to Canada, Europe including European part of Russia, Near East (Turkey, Syria), North Africa. No records are known from Asia east of Caucasus.

###### Species:

*Nemozoma alasanicum* Fursov, 1930; Georgia (varA)

Fursov, N. I. 1930: 183. Lepesme, P. & Paulian, R. 1944: 140 (subgen. *Nemosoma* s. str.)

*Nemozoma attenuatum* Van Dyke, 1915; USA: California, Washington (JRB)

Barron, J. R. 1971: 47. Lepesme, P. & Paulian, R. 1944: 140 (*Nemosoma* s.str.). Sakamoto, J. M. 2007: 342 (distribution). Van Dyke, 1915: 26

*Nemozoma brasiliense* Léveillé, 1900; Brazil: Jatahy (varA)

Lepesme, P. & Paulian, R. 1944: 138 (combined from *Monesoma*)

*Nemozoma breviatum* Peyerimhoff, 1918; Algeria (varA)

Peyerimhoff, P. M., de 1918: 329. Hallan, J. 2007–2012: http://insects.tamu.edu (syn. of *Nemozoma elongatum*?). Kolibáč, J. 2009: 128

Note: Brustel, H. (pers. comm., 2011) newly recorded 15 specimens from North Africa; he compared Palaearctic species and found *Nemozoma breviatum* valid.

*Nemozoma caucasicum* Ménétries, 1832; Austria, Poland, SW Russia, Slovakia, Ukraine, „Caucasus” (varA)

Léveillé, A. 1910: 6 (*Monesoma*). Hilszczanski, J. 2006: 29. Klausnitzer, B. 1996: 148 (larva). Kolibáč, J. 1993b: 90. Kolibáč, J. 1993a: 19, 21. Kolibáč, J. 2007a: 364 (syn. *Nemozoma fasciicole* Hampe, 1864). Lepesme, P. & Paulian, R. 1944: 141 (*Aponemosoma*). Mamaev, B. M. 1976: 1653 (larva). Milkowski, M. & Wojas, T. 2008: 172 (distribution). Nikitsky, N. B. 1974: 566 (larva). Pankow, W. 2010: 87 (distribution). Reitter, E. 1876: 13

Kolosov, ? 1931: 116 (syn. *Nemosoma curtulum* Fursov, 1930); „Chernyi les” (varA)

Fursov, N. I. 1930: 182 (*Nemosoma curtulum* Fursov, 1930; synonymized by Kolosov 1931: 116 after Hallan 2007–2012; reference not found)

*Nemozoma championi* Wickham, 1916; Western USA: Colorado, New Mexico (JRB)

Barron, J. R. 1971: 52 (*Cylidrella*). Wickham, 1916: 147

*Nemozoma cornutum* Sturm, 1826; SW Russia, Ukraine, „Caucasus” (varA)

Léveillé, A. 1910: 6. Klausnitzer, B. 1996: 148 (larva). Kolibáč, J. 2007a: 364. Lepesme, P. & Paulian, R. 1944: 140 (*Nemosoma* s.str.). Mamaev, B. M. 1976: 1654 (larva). Moragues, G. 1981: 262–263 (distribution). Nikitsky, N. B. 1974: 566 (larva). Reitter, E. 1876: 14. Sarikaya, O. & Avci, M. 2009: 253–264 (biology)

*Nemozoma cupressi* Van Dyke, 1944; USA: Nothern coast of California (JRB)

Barron, J. R. 1971: 51. Van Dyke, E. C. 1944: 147, 149

*Nemozoma cylindricolle* Fursov, 1930; Georgia (varA)

Fursov, N. I. 1930: 182. Lepesme, P. & Paulian, R. 1944: 140 (*Nemosoma* s.str.)

*Nemozoma elongatum* Linnaeus, 1761; Europe, Syria, Turkey, Tunisia (JK)

Léveillé, A. 1910: 5. Baader, E. J. 1989: 1 (biology). Bahillo de la Puebla P. & López-Colón, J. I. 2004: 129. Baier, P. 1994: 51 (biology). Baier, P. 1991: 421 (biology). Borowiec, L. 1983: 8. Burakowski, B., Mroczkowski, M. & Stefanska, J. 1986: 115. Conrad, R. 1995: 190–195 (contribution). Cunev, J. 1999: 76. Dippel, C. 1991: 473 (biology). Dippel, C. 1995: 67 (biology). Dippel, C. 1996: 391 (biology). Dippel, C. et al. 1997: 161 (biology). Gobbi, G. 1983: 52. Gueorguiev, B. et al. 2003: 107 (distribution). Heuer, H. & Vite, J. P. 1984a: 214 (biology). Heuer, H. & Vite, J. P. 1984b: 586 (biology). Hallan, J. 2007–2012: 4 (syn. *Nemosoma breviatum* Peyrimhoff, 1918?). Klausnitzer, B. 1976: 5. Klausnitzer, B. 1978: 176. Klausnitzer, B. 1996: 146 (larva). Kolibáč, J. 1993a: 21. Kolibáč, J. 1993b: 90. Kolibáč, J. 1996: 473. Kolibáč, J. 2005: 72 (redescription). Kolibáč, J. 2007a: 365 (syn. *Nemozoma fasciatum* Panzer, 1796; in *Colydium*). Kolibáč, J. 2007a: 364 (syn. *Nemozoma corsicum* Reitter, 1876). Kolibáč, J. 2007a: 365 (syn. *Nemozoma siculum* Ragusa, 1891). Kolibáč, J. 2007a: 365 (syn. *Nemozoma syriacum* Pic, 1901). Kolibáč, J. 2007a: 365 (syn. *Nemozoma elongatum* var. *tuniseum* Pic, 1900). Lepesme, P. & Paulian, R. 1944: 140 (*Nemosoma* s.str.). Mamaev, B. M. 1976: 1654 (larva). Mitter, H. 1998: 559. Nikitsky, N. B. 1974: 566 (larva). Peyrimhoff, 1918: 329 (*Nemozoma breviatum*; valid species: H. Brustel, pers. comm. 2011). Pileckis, S. & V. Monsevičius 1995: 271. Reitter, E. 1876: 13 (syn. *Nemozoma corsicum* Ratti, E. 1997: 179. Reitter, 1876; synonymized by Léveillé 1889: 8). Skatulla, U. & Feicht, E. 1992: 4 (biology). Szafraniec, S. 1997: 207 (distribution). Vogt, H. 1967: 15. Wigger, H. 1993: 68 (biology). Wigger, H. 1994: 8 (biology). Wigger, H. 1996: 55 (biology)

*Nemozoma fissiceps* Fall, 1910; USA: California, Oregon (JRB)

Barron, J. R. 1971: 49. Fall, H. C. 1910: 127

*Nemozoma gounellei* Léveillé, 1894; Brazil (AL)

Léveillé, A. 1910: 6

*Nemozoma landesi* Léveillé, 1901; Martinica (AL)

Léveillé, A. 1910: 4. Lepesme, P. & Paulian, R. 1944: 139 (subgen. *Nemosomia*)

*Nemozoma maculata* Dajoz, 1991; USA (AD)

Dajoz, R. 1991: 245 (*Cylidrella*)

*Nemozoma mollis* Sharp, 1891; Guatemala (AL)

Léveillé, A. 1910: 6 (*Cylidrella*). Kolibáč, J. 2005: 73 (redescription, combination)

*Nemozoma picta* Léveillé, 1889; Brazil (AL)

Léveillé, A. 1910: 4 (*Nemosomia*)

*Nemozoma pujoli* Léveillé, 1902; Brazil (AL)

Léveillé, A. 1910: 4

*Nemozoma punctatum* Léveillé, 1888; Brazil (AL)

Léveillé, A. 1910: 6. Lepesme, P. & Paulian, R. 1944: 140

*Nemozoma punctulata* Van Dyke, 1920; Canada: British Columbia to USA: California (JRB)

Barron, J. R. 1971: 49. Lepesme, P. & Paulian, R. 1944: 140. Van Dyke, E. C. 1916: 71, 72 (described as *punctatum*). Van Dyke, E. C. 1920: 85 (jun. homonym; renamed)

*Nemozoma schwarzi* Schaeffer, 1918; USA: Arizona, California (JRB)

Barron, J. R. 1971: 48. Lepesme, P. & Paulian, R. 1944: 140 (*Nemosoma* s.str.). Schaeffer, C. F. A. 1918: 191 (subgen. *Monesoma*)

*Nemozoma signatum* Sharp, 1891; Guatemala (AL)

Léveillé, A. 1910: 6

*Nemozoma simoni* Léveillé, 1889; Venezuela (AL)

Léveillé, A. 1910: 4 (*Nemosomia*). Lepesme, P. & Paulian, R. 1944: 140

*Nemozoma vorax* Reitter, 1876; Columbia (AL)

Léveillé, A. 1910: 4 (*Nemosomia*). Lepesme, P. & Paulian, R. 1944: 141. Reitter, E. 1876: 12 (*Nemosomia*)

##### Genus Parallelodera

Fairmaire, 1881

http://species-id.net/wiki/Parallelodera

Fig. 7 ; Map 5

Parallelodera Fairmaire, L. 1881: 256.

###### Type species.

*Parallelodera quadraticollis* Fairmaire, 1881 [by monotypy]

Léveillé, A. 1910: 8. Kolibáč, J. 2005: 74 (redescription). Kolibáč, J. 2006: 116 (phylogeny)

###### Description.

Body size: about 11.0–12.0 mm. Body shape elongate. Gular sutures wide, convergent at apex. Frontoclypeal suture present. Frons: longitudinal groove or depression present. Cranium ventrally: tufts of long setae at sides present. Submentum of males: ctenidium absent. Antennal groove present. Eyes: size flat. Eyes number: two. Mandibular apical teeth number: two, vertically situated. Antenna 11-segmented. Antennal club asymmetrical, sensorial fields present. Front coxal cavities externally closed, internally open. Pronotum subquadrate. Prepectus absent. Middle coxal cavities open. Elytra: long hairs absent. Epipleuron thin. Elytral interlocking mechanism present, carinae reduced. Elytral punctation regular, scales absent. Wing: radial cell open (outer vein present), wedge cell absent, cross vein MP3-4 absent, cross vein AA1+2-3+4 absent. Front tibiae: spines along side large. Hooked spur present in all tibiae. Claws: denticle absent.

###### Biology.

In the light of a presumable relationship with *Airora* and *Temnoscheila*, I suggest a predatory way of life.

###### Distribution.

Pacific islands as listed with the particular species.

###### Species:

*Parallelodera luteicornis* Fairmaire, 1881; Fiji, Viti Isl. (AL)

Léveillé, A. 1910: 8. Kolibáč, J. 2005: 75

*Parallelodera parallelus* Fairmaire, 1850; New Caledonia, Madera, Nuka-Hiva, Tahiti (JK)

Léveillé, A. 1910: 18 (*Tenebroides*). Léveillé, A. 1910: 18 (syn. *Tenebroides serratus* Wollaston, 1854). Kolibáč, J. 2005: 75 (combination)

*Parallelodera quadraticollis* Fairmaire, 1881; Fiji, Viti Isl. (AL)

Léveillé, A. 1910: 8. Kolibáč, J. 2005: 74 (redescription)

##### Genus Temnoscheila

Westwood, 1830

http://species-id.net/wiki/Temnoscheila

Figs 7 , 8 ; Map 5

Temnoscheila Westwood, J. O. 1830: 231.

###### Type species:

*Trogossita caerulea* Olivier, 1790 [by monotypy]

Downie, N. M. & Arnett, R. H., Jr. 1996: 937 (key). Kolibáč, J. 2005: 83 (redescription). Kolibáč, J. 2006: 109 (review of larvae), 111 (phylogeny). Kolibáč, J. 2007a: 365. Spahr, U. 1981: 74 (amber and copal fossils)

*Temnochila* Erichson, 1844 (unjustified emendation by Erichson 1844: 449)

Léveillé, A. 1910: 9. Barron, J. R. 1971: 70. Kolibáč, J. 2007a: 365 (unjustified emendation). Nikitsky, N. B. 1992: 80

*Trogossita* Olivier, 1790 [type species: *Trogossita caerulea* Olivier, 1790]

Barron, J. R. 1971: 70. Crowson, R. A. 1964a: 299. Mamaev, B. M. 1976: 1652 (larva). Matthews, E. G. 1992: 3. Reitter, E. 1876: 26 (*Trogosita*)

###### Description.

Body size: about 9.0–26.0 mm. Body shape elongate. Gular sutures reduced. Frontoclypeal suture absent. Frons: longitudinal groove or depression present. Cranium ventrally: tufts of long setae at sides present. Submentum of males: ctenidium present. Antennal groove present. Eyes: size flat. Eyes number: two. Epicranial acumination deep. Lacinial hooks absent. Galea: shape elongate. Galea: ciliate setae present. Mediostipes-Lacinia not fused. Palpifer: outer edge denticulate. Mandibular apical teeth number: two, vertically situated. Mola absent. Penicillus (at base) present (fine, often membranous). Pubescence above mola or cutting edge absent. Ventral furrow present. Basal notch shallow or absent. Labrum-Cranium not fused. Epipharyngial sclerite present. Lateral tormal process: projection curved downwards, processes not connected (*Airora*). Ligula: ciliate setae absent. Ligula rigid, weakly retroflexed, deeply emarginate. Hypopharyngeal sclerite sickle shaped. Antenna 11-segmented. Antennal club asymmetrical, sensorial fields present. Front coxal cavities externally closed, internally open. Pronotum elongate. Prepectus absent. Middle coxal cavities open. Elytra: long hairs absent. Epipleuron moderate. Elytral interlocking mechanism present, carinae reduced. Elytral punctation regular, scales absent. Wing: radial cell triangular, wedge cell present, cross vein MP3-4 present, cross vein AA1+2-3+4 absent. Front tibiae: spines along side moderate. Hooked spur present. Claws: denticle absent. Parasternites number along ventrites III–VII: one. Coxitae undivided.

Larva: Frontal arms V-shaped. Epicranial stem reduced. Endocarina present. Gular sutures conspicuous, parallel. Gula: anterior apodemes absent. Paragular sclerites present. Hypostomal rods present. Stemmata number: five. Mandibular apical teeth number: two, horizontally even, vertically situated. Lacinia mandibulae with several small spines. Mola absent. Maxillary palpi 3-segmented. Palpifer absent. Pedunculate seta present. Mala simple. Mala: bidentate protrusion absent. Cardo-Stipes partially fused. Cardo: size much smaller than stipes. Ligula present. Labial palpi 2-segmented. Prementum in single part, anterior margin projecting. Torma: two separate lateral sclerites. Antennal joints 1 and 2 elongate. Sensory appendix very small. Thoracic sclerites pattern (dorsally) 1-2-2. Thoracic sclerites pattern (ventrally) 3+1+1. Trochanter oblong. Abdominal segment IX not divided. Tergite IX flat. Urogomphi present, hooked; median process absent.

**Figure 8.**
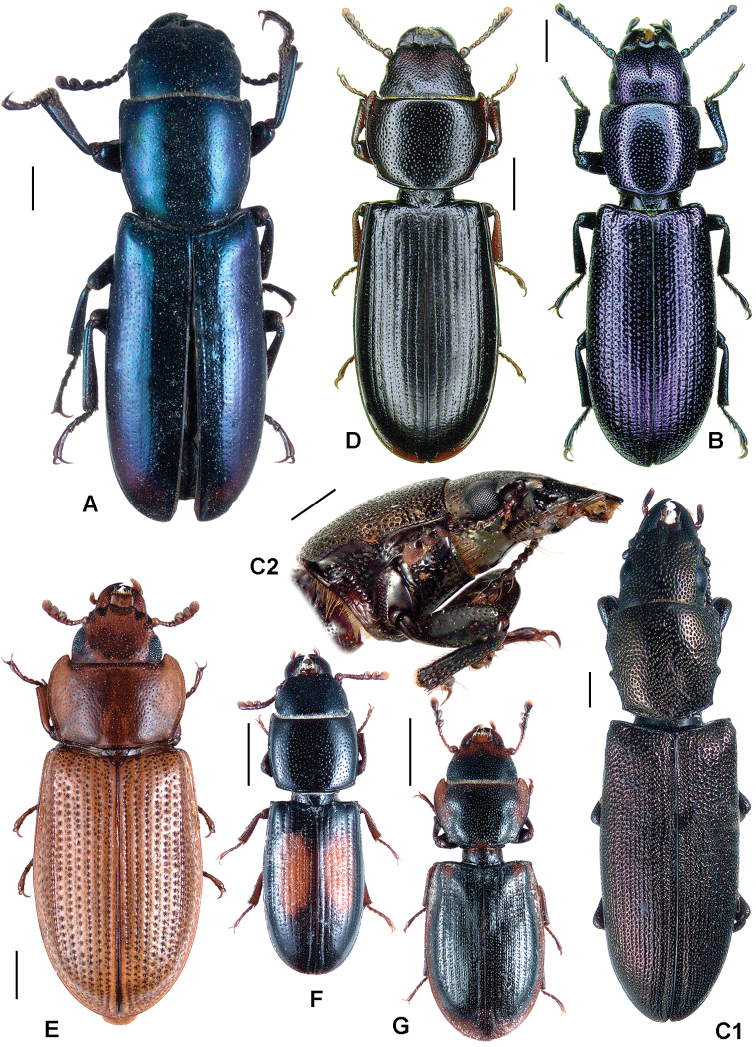
**A** *Temnoscheila smaragdina* **B** *Temnoscheila caerulea* **C** *Temnoscheila punctatissima* **D** *Tenebroides mauritanicus* **E** *Tenebroides ruber* **F** *Tenebroides bipustulatus* **G** *Tenebroides fossulatus*.

###### Biology.

Predatory. Adults hunt for xylophagous beetles on logs and branches of various trees and shrubs. Larvae live mostly under bark but sometimes dwell on the surface of wood as well.

###### Distribution.

The bulk of species are distributed in the two Americas. Only a few species spread through to the Palaearctic region. Several species tend to cosmopolitanism (*Temnoscheila caerulea*, *Temnoscheila virescens*).

###### Species:

*Temnoscheila acuta* LeConte, 1858; Texas (JRB)

Léveillé, A. 1910: 10 (*Temnochila*). Barron, J. R. 1971: 85 (*Temnochila*)

*Temnoscheila aenea* Olivier, 1790; Brazil, Porto Rico (AL)

Léveillé, A. 1910: 10 (*Temnochila*)

*Temnoscheila aerea* LeConte, 1858; Guatemala (JRB)

Léveillé, A. 1910: 10 (*Temnochila*). Barron, J. R. 1971: 78 (*Temnochila*). Barron, J. R. 1971: 78 (syn. *Temnochila virescens* var. *nyentia* Dow, 1912). Dow, R. P. 1912: 70

*Temnoscheila alticola* Sharp, 1891

Léveillé, A. 1910: 10 (*Temnochila*)

*Temnoscheila aureola* Reitter, 1875; Mexico (AL)

Léveillé, A. 1910: 10

*Temnoscheila aurora* Reitter, 1875; Brazil (AL)

Léveillé, A. 1910: 10 (*Temnochila*)

*Temnoscheila barbata* LeConte, 1863; “Cap S. Lucas” (JRB)

Léveillé, A. 1910: 10 (*Temnochila*). Barron, J. R. 1971: 73 (*Temnochila*)

*Temnoscheila bedeli* Léveillé, 1889; Venezuela (AL)

Léveillé, A. 1910: 10 (*Temnochila*)

*Temnoscheila belti* Sharp, 1891; Nicaragua (AL)

Léveillé, A. 1910: 10 (*Temnochila*)

*Temnoscheila biolleyi* Léveillé, 1903; Costa Rica (AL)

Léveillé, A. 1910: 10 (*Temnochila*)

*Temnoscheila boboensis* Sharp, 1891; Mexico (AL)

Léveillé, A. 1910: 10

*Temnoscheila boliviensis* Léveillé, 1903; Bolivia (AL)

Léveillé, A. 1910: 10 (*Temnochila*)

*Temnoscheila borrei* Reitter, 1875; Antilles, Colombia (AL)

Léveillé, A. 1910: 10 (*Temnochila*)

*Temnoscheila brevior* Léveillé, 1889; Colombia (AL)

Léveillé, A. 1910: 10 (*Temnochila*)

*Temnoscheila caerulea* Olivier, 1790; South Europe, Southwestern Asia, North Africa, China (JK)

Léveillé, A. 1910: 10. Bahillo de la Puebla P. & López-Colón, J. I. 1999: 13. Sarikaya, O. & Avci, M. 2009: 129. Borowiec, L. 1983: 10. Burakowski et al. 1986: 116. Crowson, R. A. 1972: 339 (*Trogossita*). Gobbi, G. 1983: 51 (*Temnochila*). Klausnitzer, B. 1976: 5 (*Temnochila*). Kolibáč, J. 1993a: 21 (*Temnochila*). Kolibáč, J. 1993b: 90 (*Temnochila*). Mitter, H. 1998: 560 (*Temnochila*). Kolibáč, J. 2005: 83 (redescription)

Kolibáč, J. 2007a: 36 (syn. *Temnoscheila gemella* Bedel, 1900; described as species; synonymized by Kolibáč 2007a); Algeria (JK)

Kolibáč, J. 2007a: 365 (syn. *Temnoscheila pini* Brullé, 1838); Canary Isl. (JK)

Kolibáč, J. 2007a: 365 (syn. *Temnoscheila rogenhoferi* Reitter, 1875) „India or.” (JK)

Klausnitzer, B. 1978: 176 (*Temnochila*). Léveillé, A. 1910: 11 (*Temnochila caerulea* var. *asiatica* Léveillé, 1908); Yunnan (AL)

Léveillé, A. 1910: 10 (syn. *Temnochila rogenhoferi* Reitter, 1875)

Léveillé, A. 1910: 11 (*Temnochila* var. *gemella* Bedel, 1900) Algeria (AL)

Mamaev, B. M. 1976: 1655 (*Trogossita*) (larva). Pajares, J. A. et al. 2004: 633 (biology). Whitehouse, N. J. 1997: 293 (biology). Vogt, H. 1967: 15 (*Temnochila*)

*Temnoscheila caerulea pini* Brullé, 1838; Canary Isl. (varA)

Plata-Negrache, P. & C. Prendes-Ayala 1981: 226

*Temnoscheila chalcea* Kirsch, 1873; Peru, America centr. (AL)

Léveillé, A. 1910: 10

*Temnoscheila championi* Sharp, 1891; America centr. (AL)

Léveillé, A. 1910: 10

*Temnoscheila chevrolati* Reitter, 1875; Brazil: „Cayenna”, America centr. (AL)

Léveillé, A. 1910: 10

*Temnoscheila chiriquensis* Sharp, 1891; Panama (AL)

Léveillé, A. 1910: 10

*Temnoscheila chlorodia* Mannerheim, 1843; Western USA, Western Canada (JRB)

Barron, J. R. 1971: 82 (syn. *Temnochila prosternalis* Schaeffer, 1918; synonymized by Barron 1971). Barron, J. R. 1971: 82 (syn. *Temnochila virescens chlorodia* ab. *melanica* Hatch, 1962). Barron, J. R. 1971: 82 (syn. *Temnochila viridicyanea* Mannerheim, 1843; synonymized by whom, Reitter 1875?). Barron, J. R. 1971: 82 (syn. *Temnochila virescens chlorodia* Mannerheim, 1843: Schaeffer 1918, 1920; Hatch 1962; Struble and Carpelan 1941)

Boone, C. K. S. et al. 2008: 411 (biology). Dahlsten, D. L. et al. 2004: 1554 (biology). DeMars, C. J. Jr. et al. 1986: 881 (biology). Dominguez-Sanchez, B. et al. 2008: 175 (biology). Gaylord, M. L. W. et al. 2008: 57 (biology). Goheen, D. J. et al. 1985: 1535 (biology). Fettig, Ch. J. & Dabney, Ch. P. 2006: 75 (biology). Fettig, Ch. J. et al. 2004: 490 (biology). Fettig, Ch. J. et al. 2005: 748 (biology). Fettig, Ch. J. et al. 2007: 141 (biology). Léveillé, A. 1910: 13 (*Temnoscheila virescens*). Marsden, M. A. et al. 1981: 1 (biology). Miller, D. R. et al. 1997: 2013 (biology). Ross, D. W. & Daterman, G. E. 1998: 500 (biology). Swezey, S. L. & Dahlsen, D. L. 1982: 142 (biology). Williams, K. K. et al. 2009: 351 (biology). Zhou, J. et al. 2001: 993 (biology)

*Temnoscheila chrysosterna* Reitter, 1875; Brazil: „Cayenna” (AL)

Léveillé, A. 1910: 10

*Temnoscheila colossus* Serville, 1828; Colombia, Brazil: „Cayenna” (AL)

Léveillé, A. 1910: 11

*Temnoscheila corinthia* Reitter, 1875; Mexico (AL)

Léveillé, A. 1910: 11

*Temnoscheila costaricensis* Sharp, 1891; Costarica (AL)

Léveillé, A. 1910: 11

*Temnoscheila curta* Léveillé, 1889; Cayenna (AL)

Léveillé, A. 1910: 11

*Temnoscheila davidi* Léveillé, 1898; Ecuador (AL)

Léveillé, A. 1910: 11

*Temnoscheila dendrobia* Gistl & Bromme, 1850; Colombia (AL)

Léveillé, A. 1910: 11

*Temnoscheila derasa* Sharp, 1891; Mexico, Guatemala (AL)

Léveillé, A. 1910: 11

*Temnoscheila diffinis* Sharp, 1891; Mexico (AL)

Léveillé, A. 1910: 11

*Temnoscheila digitata* Sharp, 1891; America centr. (AL)

Léveillé, A. 1910: 11

*Temnoscheila doumerci* Serville, 1828; Brazil: „Cayenna” (AL)

Léveillé, A. 1910: 11

*Temnoscheila dryadis* Reitter, 1875; Mexico (AL)

Léveillé, A. 1910: 11

*Temnoscheila ebenina* Blanchard, 1875; Bolivia, Uruguay (AL)

Léveillé, A. 1910: 11

*Temnoscheila edendata* Schaeffer, 1918; USA: Arizona, California, Mexico: Baja (JRB)

Barron, J. R. 1971: 76 (syn. *Temnochila sonorana* Barrett, 1932: 171; synonymized by Barron 1971)

Schaeffer, C. F. A. 1918: 194

*Temnoscheila exarata* Sharp, 1891; Mexico (AL)

Léveillé, A. 1910: 11

*Temnoscheila festiva* Serville, 1828; Brazil (AL)

Léveillé, A. 1910: 11 (syn. *Temnochila splendens* Gray in Griffith 1832 (*Temnoscheila*); synonymized by whom?)

*Temnoscheila foveicollis* Reitter, 1875; Brazil: „Cayenna”, „Para” (AL)

Léveillé, A. 1910: 11

*Temnoscheila fraudulenta* Sharp, 1891; Mexico (AL)

Léveillé, A. 1910: 11

*Temnoscheila fulgidovittata* Blanchard, 1875; Bolivia (AL)

Léveillé, A. 1910: 11

*Temnoscheila geminata* Sharp, 1891; Panama (AL)

Léveillé, A. 1910: 11

*Temnoscheila gigantea* Reitter, 1875; Brazil (AL)

Léveillé, A. 1910: 11

*Temnoscheila gloriosa* Reitter, 1875; Colombia (AL)

Léveillé, A. 1910: 11

*Temnoscheila grandis* Sharp, 1891; Guatemala (AL)

Léveillé, A. 1910: 11

*Temnoscheila grilloi* Léveillé, 1905; Brazil: „Paraná” (AL)

Léveillé, A. 1910: 11

*Temnoscheila grouvellei* Léveillé, 1889; America centr. (AL)

Léveillé, A. 1910: 12

*Temnoscheila guatemalana* Sharp, 1891; America centr. (AL)

Léveillé, A. 1910: 12

*Temnoscheila hubbardi* Léveillé, 1889; USA: Florida (JRB)

Léveillé, A. 1910: 12. Barron, J. R. 1971: 75

*Temnoscheila insignis* Reitter, 1875; Colombia (AL)

Léveillé, A. 1910: 12. Reitter, E. 1875: 10 (homonym with *Temnoscheila insignis* Heer, 1868)

† *Temnoscheila insignis* Heer, 1868 (*Trogosita*); Tertiary: Eocene; Greenland (JRB)

Barron, J. R. 1971: 120

*Temnoscheila iris* Reitter, 1875; America centr. (AL)

Léveillé, A. 1910: 12

*Temnoscheila jansoni* Léveillé, 1889; Brazil: “Minas Geraes” (AL)

Léveillé, A. 1910: 12

*Temnoscheila japonica* Reitter, 1875; Japan, North Korea, Russian Far East, Northeastern China (JK)

Léveillé, A. 1910: 12. Esaki, T. et al. 1951: 1060. Inouye, M. & Nobuchi, A. 1957: 194 (*Temnochila*) (larva). Klausnitzer, B. 1996: 146 (larva). Kolibáč, J. 2007a: 365. Mamaev, B. M. 1976: 1655 (*Trogossita*) (larva). Nakane, T. K. et al. 1963: 181. Nikitsky, N. B. 1992: 80

*Temnoscheila jekeli* Reitter, 1875; Colombia (AL)

Léveillé, A. 1910: 12 (syn. *Temnochila sennevillei* Léveillé, 1878)

*Temnoscheila kirschi* Reitter, 1875; Colombia: Bogota (AL)

Léveillé, A. 1910: 12

*Temnoscheila laevicollis* Reitter, 1875; Brazil: „Cayenna” (AL)

Léveillé, A. 1910: 12

*Temnoscheila laticollis* Reitter, 1875; Mexico (AL)

Léveillé, A. 1910: 12

*Temnoscheila lebasi* Reitter, 1875; Colombia (AL)

Léveillé, A. 1910: 12

*Temnoscheila leveillei* Sharp, 1891; Panama (AL)

Léveillé, A. 1910: 12

*Temnoscheila lucens* Reitter, 1875; Brazil (AL)

Léveillé, A. 1910: 12

*Temnoscheila metallica* Percheron, 1835?; Mexico (AL)

Léveillé, A. 1910: 12 (syn. *Temnochila mexicana* Reitter, 1875; synonymized by Salle 1877)

*Temnoscheila mirabilis* Reitter, 1875; Colombia (AL)

Léveillé, A. 1910: 12

*Temnoscheila miranda* Sharp, 1891; Mexico (AL)

Léveillé, A. 1910: 12

*Temnoscheila nigritarsis* Léveillé, 1889; Brazil (AL)

Léveillé, A. 1910: 12

*Temnoscheila obscura* Reitter, 1875; North America? (AL)

Léveillé, A. 1910: 12

*Temnoscheila obsoleta* Reitter, 1875; Mexico (AL)

Léveillé, A. 1910: 12

*Temnoscheila obtusicollis* Reitter, 1875; Venezuela (AL)

Léveillé, A. 1910: 12

*Temnoscheila olivacea* Reitter, 1875; Colombia (AL)

Léveillé, A. 1910: 12

*Temnoscheila olivicolor* Léveillé, 1889; Ecuador (AL)

Léveillé, A. 1910: 12

*Temnoscheila omolopha* Barron, 1971; USA: Arizona, New Mexico (JRB)

Barron, J. R. 1971: 77

*Temnoscheila parva* Léveillé, 1889; Santo Domingo (AL)

Léveillé, A. 1910: 12

*Temnoscheila patricioi* Kirsch, 1881; „S. Thomé Isl.” (AL)

Léveillé, A. 1910: 12

*Temnoscheila peruviana* Léveillé, 1907; Peru (AL)

Léveillé, A. 1910: 12

*Temnoscheila planicollis* Sharp, 1891; Guatemala (AL)

Léveillé, A. 1910: 12

*Temnoscheila planipennis* Léveillé, 1889; Mexico (AL)

Léveillé, A. 1910: 12 (syn. *Temnochila metallica* Reitter, 1875; synonymized by whom?)

*Temnoscheila polita* Chevrolat, 1833; America centr. (AL)

Léveillé, A. 1910: 12

*Temnoscheila pollens* Sharp, 1891; Mexico (AL)

Léveillé, A. 1910: 12

*Temnoscheila polygonalis* Léveillé, 1899; Brazil (AL)

Léveillé, A. 1910: 12

*Temnoscheila portoricensis* Léveillé, 1907; Porto Rico (AL)

Léveillé, A. 1910: 12

*Temnoscheila praeterita* Sharp, 1891; Mexico (AL)

Léveillé, A. 1910: 12

*Temnoscheila punctatissima* Reitter, 1875; Brazil (AL)

Léveillé, A. 1910: 12

*Temnoscheila punicea* Reitter, 1875; Brazil (AL)

Léveillé, A. 1910: 12

*Temnoscheila quadricollis* Reitter, 1875; America centr. (AL)

Léveillé, A. 1910: 13

*Temnoscheila querula* Sharp, 1891; America centr. (AL)

Léveillé, A. 1910: 13

*Temnoscheila reitteri* Kirsch, 1885; Colombia (AL)

Léveillé, A. 1910: 13

*Temnoscheila reversa* Sharp, 1891; Mexico (AL)

Léveillé, A. 1910: 13

*Temnoscheila rhyssa* Barron, 1971; USA: California, Idaho (JRB)

Barron, J. R. 1971: 77

*Temnoscheila rugulosa* Kirsch, 1873; Peru (AL)

Léveillé, A. 1910: 13

*Temnoscheila sallei* Léveillé, 1889; Guatemala, Yucatan (AL)

Léveillé, A. 1910: 13

*Temnoscheila salvini* Sharp, 1891; Panama (AL)

Léveillé, A. 1910: 13

*Temnoscheila sculpturata* Reitter, 1875; Colombia (AL)

Léveillé, A. 1910: 13

*Temnoscheila sharpi* Léveillé, 1894; Bogota (AL)

Léveillé, A. 1910: 13

*Temnoscheila smithi* Sharp, 1891; Mexico (AL)

Léveillé, A. 1910: 13

*Temnoscheila splendida* Gory, 1831; Brazil: Cayenna (AL)

Léveillé, A. 1910: 13

*Temnoscheila steinheili* Reitter, 1875; Colombia (AL)

Léveillé, A. 1910: 13

*Temnoscheila stipes* Sharp, 1891; Mexico (AL)

Léveillé, A. 1910: 13

*Temnoscheila subcylindrica* Léveillé, 1907; Brazil (AL)

Léveillé, A. 1910: 13

*Temnoscheila sulcifrons* Sharp, 1891; America centr. (AL)

Léveillé, A. 1910: 13

*Temnoscheila sulcisternum* Léveillé, 1889; Jamaica (AL)

Léveillé, A. 1910: 13

*Temnoscheila suturata* Reitter, 1875; Mexico, Brazil (AL)

Léveillé, A. 1910: 13

*Temnoscheila telemanensis* Sharp, 1891; Guatemala (AL)

Léveillé, A. 1910: 13

*Temnoscheila tristis* Mulsant & Rey, 1853; Italia, Argentina, Colombia, Brazil (JK)

Léveillé, A. 1910: 13 (syn. *Temnochila cribricollis* Reitter, 1875; synonymized by whom?). Kolibáč, J. 2007a: 365

*Temnoscheila urbensis* Sharp, 1891; Mexico (AL)

Léveillé, A. 1910: 13

*Temnoscheila varians* Guérin, 1846; Brazil: Cayenna (AL)

Léveillé, A. 1910: 13

*Temnoscheila variicolor* Léveillé, 1889; Colombia (AL)

Léveillé, A. 1910: 13

*Temnoscheila virescens* Fabricius, 1775; Guayana, Central America, USA, introduced to Australia (varA)

Léveillé, A. 1910: 13. Abbott, I. 1993: 35 (biology). Barron, J. R. 1971: 79. Barron, J. R. 1971: 80 (syn. *Temnochila cyanea* Reitter, 1875; syn. by Léveillé, 1888). Barron, J. R. 1971: 80 (syn. *Temnochila cyanea* Reitter, 1875; may be synonym of *Temnoscheila chlorodia*! Note Barron 1971). Billings, R. F. 1985: 483 (biology). Billings, R. F. & Cameron, R. S. 1984: 1542 (biology). Böving, A. G. & Craighead, F. C. 1931: 273 (larva). Klausnitzer, B. 1978: 176. Klausnitzer, B. 1996: 146 (larva). Lawson, S. A. & Morgan, F. D. 1992: 225 (biology). Lawson, S. A. & Morgan, F. D. 1993: 139 (biology). Massey, C. L. et al. 1977: 1 (biology). Matthews, E. G. 1992: 3. McCravy, K. W. et al. 2000: 77 (biology). Page, J. M. 1981: 217 (biology). Reeve, J. D. S. et al. 2009: 183 (biology)

*Temnoscheila yuccae* Crotch, 1874; USA: California, Nevada; Mexico: Baja (JRB)

Léveillé, A. 1910: 13. Barron, J. R. 1971: 74

*Temnoscheila* sp.

Beutel, R. G. & Ślipiński, S. A. 2001: 219 (phylogeny, morphology). Costa, C. et al. 1988: 177 (larva)

##### Genus Tenebroides

Piller & Mitterpacher, 1783

http://species-id.net/wiki/Tenebroides

Figs 2 , 8 ; Map 5

Tenebroides Piller, M. & Mitterpacher, L. 1783: 87.

###### Type species.

*Tenebrio mauritanicus* Linnaeus, 1758 [designated by Westwood 1838]

Léveillé, A. 1910: 14. Barron, J. R. 1971: 88. Crowson, R. A. 1964a: 299. Downie, N. M. & Arnett, R. H. Jr. 1996: 937 (key). Kobayashi, K. 1980: 1–6 (ecology). Kolibáč, J. 2005: 84 (redescription). Kolibáč, J. 2006: 111 (phylogeny). Kolibáč, J. et al. 2005: 30 + 134 (key). Mamaev, B. M. 1976: 1652 (larva). Matthews, E. G. 1992: 3. Merkl, O. 1993: 12 (key). Nikitsky, N. B. 1992: 80. Ratti, E. 1997: 178 (*Tenebroides* sp.). Reitter, E. 1876: 28. Spahr, U. 1981: 74 (amber and copal fossils). Uchida, A. 1980: 61–73 (biology)

*Trogossita* Olivier, 1790 [type species: *Trogossita caerulea* Olivier, 1790]

Barron, J. R. 1971: 88 (list of references)

###### Description.

Body size: about 3.5–12.0 mm. Body shape elongate. Gular sutures wide, convergent at apex. Frontoclypeal suture absent. Frons: longitudinal groove or depression absent. Cranium ventrally: tufts of long setae at sides present. Submentum: ctenidium absent. Antennal groove present. Eyes: size flat. Eyes number: two. Epicranial acumination moderate. Lacinial hooks absent. Galea: shape elongate. Galea: ciliate setae present. Mediostipes-Lacinia fused together. Palpifer: outer edge denticulate. Mandibular apical teeth number: two, horizontally situated. Mola absent. Penicillus (at base) present (fine, often membranous). Pubescence above mola or cutting edge absent. Ventral furrow present. Basal notch moderate. Labrum-Cranium not fused. Epipharyngial sclerite present. Lateral tormal process: projection curved downwards, processes not connected (*Airora*). Ligula: ciliate setae present. Ligula rigid, weakly retroflexed, weakly emarginate. Hypopharyngeal sclerite consisting of two separate parts. Antenna 11-segmented. Antennal club asymmetrical, sensorial fields present. Front coxal cavities externally closed, internally open. Pronotum transverse. Prepectus absent. Middle coxal cavities open. Elytra: long hairs absent. Epipleuron moderate. Elytral interlocking mechanism present, carinae reduced. Elytral punctation regular, scales absent. Wing: radial cell open (outer vein present), wedge cell absent, cross vein MP3-4 absent, cross vein AA1+2-3+4 absent. Front tibiae: spines along side moderate. Hooked spur present. Claws: denticle absent. Spiculum gastrale absent. Tegmen composed of three parts. Coxitae undivided.

Larva: Frontal arms V-shaped. Epicranial stem reduced. Endocarina present. Gular sutures conspicuous, parallel. Gula: anterior apodemes absent. Paragular sclerites present. Hypostomal rods absent. Stemmata number: five. Mandibular apical teeth number: two, horizontally situated. Lacinia mandibulae with several small spines. Mola absent. Maxillary palpi 3-segmented. Palpifer absent. Pedunculate seta present. Mala simple. Mala: bidentate protrusion absent. Cardo-Stipes partially fused. Cardo: size much smaller than stipes. Ligula present. Labial palpi 2-segmented. Prementum in single part, anterior margin with notch. Torma: two separate lateral sclerites. Antennal joints 1 and 2 elongate. Sensory appendix medium sized (to half of joint 3). Thoracic sclerites pattern (dorsally) 1-2-2. Thoracic sclerites pattern (ventrally) 3+1+1. Trochanter oblong. Abdominal segment IX not divided. Tergite IX flat. Urogomphi present, hooked; median process absent.

###### Biology.

The species of the genus are mostly predatory, occasionally feeding on grains (e.g. the cadelle beetle, *Tenebroides mauritanicus*, which is adapted to a synanthropic way of life and is a serious pest of stored grain). It is probably originally European. The wild population, sometimes designated as the separate species *Tenebriodes fuscus* Goeze, 1777, lives in forests and its adults and larvae may be found under the bark of deciduous trees, where they feed on other insects. Supposed differences between adults of the both species can be summarized as follows: (1) *Tenebriodes fuscus* – antennae dilated from antennomere 6; elytra weakly glabrous, densely transversely wrinkled; frons narrower than that in the following species. (2) *Tenebroides mauritanicus* – antennae dilated from antennomere 8; elytra rather dull, sparsely transversely wrinkled; frons wider than that in *Tenebriodes fuscus*.

###### Distribution.

The bulk of species is distributed in the two Americas. Only a few species live in the Palaearctic region. Synanthropic *Tenebroides mauritanicus* is cosmopolitan.

###### Species:

*Tenebroides aenelpennis* Reitter, 1875; Brazil (AL)

Léveillé, A. 1910: 14

*Tenebroides aeneus* Reitter, 1875; Colombia (AL)

Léveillé, A. 1910: 14

*Tenebroides albomaculatus* Reitter, 1875; Colombia, America centr. (AL)

Léveillé, A. 1910: 14

*Tenebroides albonotatus* Reitter, 1875; Brazil: Cayenna (AL)

Léveillé, A. 1910: 15

*Tenebroides alticola* Sharp, 1891; Guatemala (AL)

Léveillé, A. 1910: 15

*Tenebroides alutaceus* Léveillé, 1905; Brazil (AL)

Léveillé, A. 1910: 15

*Tenebroides americanus* Kirby, 1837; USA, Canada (JRB)

Léveillé, A. 1910: 15 (syn. *Tenebroides castaneus* Melsheimer, 1844; synonymized by LeConte 1863). Barron, J. R. 1971: 103 (syn. *Trogosita castanea* Melsheimer, 1844). Barron, J. R. 1971: 103 (syn. *Trogosita nigrita* Horn, 1862; synonymized by LeConte 1863)

*Tenebroides anceps* Léveillé, 1889; America centr. (AL)

Léveillé, A. 1910: 15

*Tenebroides antennalis* Reitter, 1875; Colombia (AL)

Léveillé, A. 1910: 15

*Tenebroides auriculatus* Sharp, 1891; Guatemala (AL)

Léveillé, A. 1910: 15

*Tenebroides australis* Boisduval, 1835; Tasmania (?) (AL)

Léveillé, A. 1910: 15

*Tenebroides bimaculatus* Melsheimer, 1844; USA, „Pennsylvania” (JRB)

Léveillé, A. 1910: 15. Barron, J. R. 1971: 102

*Tenebroides bipustulatus* Fabricius, 1801; Brazil: Cayenna (AL)

Léveillé, A. 1910: 15 (syn. var. *impressifrons* Reitter, 1875: “Amer. mer.”; syn. n.)

*Tenebroides boggianii* Léveillé, 1905; Paraguay (AL)

Léveillé, A. 1910: 15

*Tenebroides bonvouloiri* Léveillé, 1889; Mexico (AL)

Léveillé, A. 1910: 15 (var. *chontalensis* Sharp, 1891: Nicaragua; syn. n.)

*Tenebroides brevis* Léveillé, 1889; Brazil (AL)

Léveillé, A. 1910: 15

*Tenebroides breviusculus* Reitter, 1875; America centr., Brazil (AL)

Léveillé, A. 1910: 15

*Tenebroides brunneovittatus* Léveillé, 1894; America centr., Brazil (AL)

Léveillé, A. 1910: 15

*Tenebroides brunneus* Léveillé, 1889; Brazil: Cayenna (AL)

Léveillé, A. 1910: 15

*Tenebroides bugnioni* Léveillé, 1903; Colombia (AL)

Léveillé, A. 1910: 15

*Tenebroides carbonarius* Léveillé, 1889; Brazil: Cayenna (AL)

Léveillé, A. 1910: 15

*Tenebroides carinatus* Léveillé, 1894; Brazil (AL)

Léveillé, A. 1910: 15

*Tenebroides celatus* Sharp, 1891; America centr. (AL)

Léveillé, A. 1910: 15

*Tenebroides chevrolati* Reitter, 1875; Mexico (AL)

Léveillé, A. 1910: 15

*Tenebroides circumcinctus* Léveillé, 1889; America centr. (AL)

Léveillé, A. 1910: 15

*Tenebroides collaris* Sturm, 1807; Canada: Ontario, E USA to Michigan and E Texas (JRB)

Léveillé, A. 1910: 15 (syn. *Tenebroides nigripennis* Sturm, 1826). Barron, J. R. 1971: 97 (*Trogosita nigripennis* Sturm, 1826; *nomen nudum*). Barron, J. R. 1971: 97 (*Trogosita nigripennis* Dejean, 1836; *nomen nudum*)

*Tenebroides complicatus* Sharp, 1891; America centr. (AL)

Léveillé, A. 1910: 15

*Tenebroides cordicollis* Léveillé, 1889; Brazil (AL)

Léveillé, A. 1910: 15

† *Tenebroides corrugata* Wickham, 1913; USA: Colorado, Florissant; Early Oligocene (JRB)

Barron, J. R. 1971: 120. Ponomarenko, A. G. & Kireichuk, A. G. 2005–2008: http://www.zin.ru/animalia/Coleoptera/rus/paleosy2.htm. Schmied, H. et al. 2009: 23. Wickham, H. F. 1913: 291

*Tenebroides corticalis* Melsheimer, 1844; Guatemala, Mexico, USA, Canada to Alaska (JRB)

Léveillé, A. 1910: 15 (syn. *Tenebroides dubius* Melsheimer, 1844; synonymized by Barron 1971). Léveillé, A. 1910: 16 (syn. *Tenebroides intermedius* Horn, 1862; synonymized by Barron 1971). Léveillé, A. 1910: 17 (syn. *Tenebroides limbalis* Melsheimer, 1844; synonymized by Barron 1971). Barron, J. R. 1971: 115 (syn. *Trogosita limbalis* Melsheimer, 1844; synonymized by LeConte 1863). Barron, J. R. 1971: 115 (syn. *Trogosita dubia* Melsheimer, 1844; synonymized by LeConte 1863). Barron, J. R. 1971: 115 (*Trogosita conformis* Dejean, 1836; *nomen nudum*). Barron, J. R. 1971: 115 (syn. *Trogosita intermedia* Horn, 1862; synonymized by LeConte 1863)

*Tenebroides crassicornis* Horn, 1862; Western states of Canada, USA (JRB)

Léveillé, A. 1910: 15. Barron, J. R. 1971: 95 (syn. *Trogosita californica* Horn, 1862; synonymized by LeConte 1863). Barron, J. R. 1971: 95 (syn. *Trogosita pleuralis* Horn, 1862; synonymized by LeConte 1863). Léveillé, A. 1910: 18. (*Tenebroides pleuralis* Horn, 1862: California)

*Tenebroides cribratus* Léveillé, 1894; Mexico (AL)

Léveillé, A. 1910: 15

*Tenebroides cucujoides* Reitter, 1875; Colombia (AL)

Léveillé, A. 1910: 15

*Tenebroides delicatus* Léveillé, 1899; Brazil (AL)

Léveillé, A. 1910: 16

*Tenebroides depressior* Palisot de Beauvois, 1811 (*Trogossita*) incertae sedis; N America (JRB)

Léveillé, A. 1910: 16. Barron, J. R. 1971: 119 (*Trogossita depressior* Palisot de Beauvois, 1811; *incertae sedis*)

*Tenebroides depressus* Guérin, 1846; Brazil, America centr. (AL)

Léveillé, A. 1910: 16

*Tenebroides difficilis* Léveillé, 1889; Honduras (AL)

Léveillé, A. 1910: 16

*Tenebroides dilatatus* Erichson, 1847; Peru (AL)

Léveillé, A. 1910: 16

*Tenebroides donckieri* Léveillé, 1902; Brazil (AL)

Léveillé, A. 1910: 16

*Tenebroides elongatulus* Jacquelin du Val, 1857; Cuba (AL)

Léveillé, A. 1910: 16

† *Tenebroides emortua* Germar, 1849; Germany: Orsberg; Tertiary: Upper Oligocene (varA)

Mörs, T. 1995: ii. Ponomarenko, A. G. & Kireichuk, A. G. 2005–2008: http://www.zin.ru/animalia/Coleoptera/rus/paleosy2.htm. Schmied, H. et al. 2009: 23

† *Tenebroides eocenica* Meunier, 1921; Germany: Messel; Tertiary: middle Eocene (varA)

Meunier, F. 1921: ii. Ponomarenko, A. G. & Kireichuk, A. G. 2005–2008: http://www.zin.ru/animalia/Coleoptera/rus/paleosy2.htm. Schmied, H. et al. 2009: 23

*Tenebroides excellens* Sharp, 1891; Panama (AL)

Léveillé, A. 1910: 16

*Tenebroides explanatus* Reitter, 1875; Colombia (AL)

Léveillé, A. 1910: 16

*Tenebroides facilis* Sharp, 1891; Mexico (AL)

Léveillé, A. 1910: 16

*Tenebroides farmairei* Léveillé, 1889; Tonga-Tabu (AL)

Léveillé, A. 1910: 16

*Tenebroides fenestratus* Léveillé, 1889; Mexico (AL)

Léveillé, A. 1910: 16

*Tenebroides flaviclavis* Reitter, 1875; Cuba (AL)

Léveillé, A. 1910: 16

*Tenebroides floridanus* Schaeffer, 1918; USA: Florida, Louisiana, centr. America (JRB)

Barron, J. R. 1971: 111. Schaeffer, C. F. A. 1918: 199

*Tenebroides fossulatus* Léveillé, 1899; Bolivia (AL)

Léveillé, A. 1910: 16

*Tenebroides fryi* Léveillé, 1898; Brazil (AL)

Léveillé, A. 1910: 16

*Tenebroides fulgens* Sharp, 1891; Panama (AL)

Léveillé, A. 1910: 16

*Tenebroides fulvolineatus* Léveillé, 1889; Brazil (AL)

Léveillé, A. 1910: 16

*Tenebroides germaini* Léveillé, 1895; Bolivia (AL)

Léveillé, A. 1910: 16

*Tenebroides godmani* Sharp, 1891; Panama (AL)

Léveillé, A. 1910: 16

*Tenebroides gounellei* Léveillé, 1889; Brazil: “Minas Geraes” (AL)

Léveillé, A. 1910: 16

*Tenebroides gracilipes* Sharp, 1891; Panama (AL)

Léveillé, A. 1910: 16

*Tenebroides harpaloides* Léveillé, 1889; Mexico (AL)

Léveillé, A. 1910: 16

*Tenebroides helophorus* Sharp, 1891; Mexico (AL)

Léveillé, A. 1910: 16

*Tenebroides humeralis* Léveillé, 1889; Colombia (AL)

Léveillé, A. 1910: 16

*Tenebroides importunus* Léveillé, 1905; Brazil (AL)

Léveillé, A. 1910: 16

*Tenebroides incertus* Léveillé, 1889; Mexico (AL)

Léveillé, A. 1910: 16

† *Tenebroides insignis* Heer, 1883; Greenland: Atanrkerdluk; Mesozoic: Upper Cretaceous, Maastrichtian (varA)

Ponomarenko, A. G. & Kireichuk, A. G. 2005–2008: http://www.zin.ru/animalia/Coleoptera/rus/paleosy2.htm. Schmied, H. et al. 2009: 23

*Tenebroides insinuans* Walker, 1858; Ceylon (?) (AL)

Léveillé, A. 1910: 16

*Tenebroides instabilis* Sharp, 1891; Mexico (AL)

Léveillé, A. 1910: 16

*Tenebroides iteratus* Sharp, 1891; Mexico (AL)

Léveillé, A. 1910: 16

*Tenebroides jatahyensis* Léveillé, 1902; Brazil (AL)

Léveillé, A. 1910: 16

*Tenebroides latens* Wollaston, 1862; Canary Isl: Teneriffa (JK)

Léveillé, A. 1910: 16. Kolibáč, J. 2007a: 365. Plata-Negrache, P. & Prendes-Ayala, C. 1981: 230

*Tenebroides laticollis* Horn, 1862; Eastern USA, Canada (JRB)

Léveillé, A. 1910: 16. Barron, J. R. 1971: 105 (syn. *Trogosita obscura* Horn, 1862; synonymized by Leconte 1863)

*Tenebroides latus* Léveillé, 1889; Mexico (AL)

Léveillé, A. 1910: 17

*Tenebroides lineolatus* Reitter, 1877; Colombia (AL)

Léveillé, A. 1910: 17

*Tenebroides litigiosus* Reitter, 1875; Brazil (AL)

Léveillé, A. 1910: 17

*Tenebroides longicornis* Léveillé, 1889 – Brazil (AL)

Léveillé, A. 1910: 17

*Tenebroides longulus* Sharp, 1891; Guatemala (AL)

Léveillé, A. 1910: 17

*Tenebroides lucidus* Sharp, 1891; Panama (AL)

Léveillé, A. 1910: 17

*Tenebroides marginatus* Palisot de Beauvois, 1811; S and middle E USA (JRB)

Léveillé, A. 1910: 17. Barron, J. R. 1971: 99 (syn. *Tenebroides cucujiformis* Horn, 1862; synonymized by LeConte 1863). Barron, J. R. 1971: 99. Léveillé, A. 1910: 15 (*Tenebroides cucujiformis* Horn, 1862). Léveillé, A. 1910: 16 (homonym *Tenebroides marginatus* Latreille, 1833 replaced by *Tenebroides latreillei* Léveillé, 1889: “Amer. aequin.”; synonymized by Barron 1971?)

*Tenebroides marginicollis* Sharp, 1891; Guatemala (AL)

Léveillé, A. 1910: 17

*Tenebroides maroccanus* Reitter, 1884; N Africa, Azores, Corsica, Spain (JK)

Léveillé, A. 1910: 17 (var. *baillioti* Léveillé, 1903: Madrid; syn. n.). Audisio, P. et al. 1995: 14. Kolibáč, J. 2007a: 365. Villemant, C. & Ramzi, H. 1997: 441 (biology)

*Tenebroides marseuli* Reitter, 1875; St. Catharina (AL)

Léveillé, A. 1910: 17

*Tenebroides mauritanicus* Linnaeus, 1758 (*Tenebrio*); cosmopolitan; type locality: “Algiriae” (varA)

(syn. *Lucanus dubius* Scriba, 1790; *Carabus bucephalus* Herbst, 1783; *Lucanus fuscus* Goeze, 1777; *Lucanus fuscus* Preyssler, 1790; *Platycerus fuscus* Geoffroy, 1762; *Platycerus striatus* Fourcroy, 1785; *Tenebrio caraboides* Linnaeus, 1758: type locality: “Europa”; *Tenebrio piceus* Schaller, 1783; *Tenebrio planus* Quensel, 1790; *Tenebroides complanatus* Piller & Mitterpacher, 1783; *Tenebrio piceus* Schaller, 1783; *Trogosita affinis* White, 1846)

Léveillé, A. 1910: 17 (syn. *Tenebrioides fuscus* Goeze, 1777). Léveillé, A. 1910: 17 (var. *nitidus* Horn, 1862: 83 synonymized by Kolibáč 2007). Arbogast, R. T. & Byrd, R. V. 1982: 61 (biology). Audisio, P. et al. 1995: 14 (*Tenebroides fuscus* Goeze, 1777). Bahillo de la Puebla P. & López-Colón, J. I. 1999: 13. Bahillo de la Puebla P. & López-Colón, J. I. 1999: 13 (*Tenebroides fuscus* Goeze, 1777). Bahillo de la Puebla P. & López-Colón, J. I. 2004: 129. Sarikaya, O. & Avci, M. 2009: 129 (*Tenebroides fuscus* Goeze, 1777). Barron, J. R. 1971: 92–93. Barron, J. R. 1971: 93 (syn. *Trogosita nitida* Horn, 1862; synonymized by LeConte 1863). Borowiec, L. 1983: 11. Borowiec, L. 1983: 11 (*Tenebroides fuscus* Goeze, 1777). Burakowski, B. et al. 1986: 117. Burakowski, B. et al. 1986: 117 (*Tenebroides fuscus* Goeze, 1777). Dziadik-Turner, C. et al. 1981: 546 (biology). Esaki, T. et al. 1951: 1061. Faustini, D. I. & D. G. H. Halstead 1982: 45. Gollkowski, V. 1988: 42. Herger, P. 1998: 105 (*Tenebroides fuscus* Goeze, 1777; distribution). Holzer, E. 1995: 30. Huber, Ch. & Kobel, E. 1994: 1. Kampsu, G. 2005: 17 (biology). Klausnitzer, B. 1976: 8. Klausnitzer, B. 1976: 8 (*Tenebrioides fuscus* Goeze, 1777). Klausnitzer, B. 1978: 176. Klausnitzer, B. 1996: 146. Klausnitzer, B. 1996: 151 (*Tenebroides fuscus* Goeze, 1777). Kolibáč, J. 1993a: 21. Kolibáč, J. 1993a: 21 (*Tenebroides fuscus* Goeze, 1777). Kolibáč, J. 1993b: 90. Kolibáč, J. 1993b: 90 (syn. *Tenebroides fuscus* Goeze, 1777). Kolibáč, J. 1996b: 473. Kolibáč, J. 1999b: 12. Kolibáč, J. 2005: 84 (redescription). Kolibáč, J. 2006: 109 (*Tenebroides fuscus* Goeze, 1777; larva). Kolibáč, J. 2007a: 365 (*Tenebroides fuscus* Goeze, 1777). Kolibáč, J. 2007a: 365 (syn. *Tenebroides nitidus* Horn, 1862). Kolibáč, J. 2007a: 365. Kolibáč, J. 2007a: 365 (syn. *Tenebroides piceus* Dalla Torre, 1879). Mamaev, B. M. 1976: 1654. Matthews, E. G. 1992: 3. Mitter, H. 1998: 560. Mitter, H. 1998: 560 (*Tenebroides fuscus* Goeze, 1777). Nakane, T. et al. 1963: 181. Nikitsky, N. B. 1992: 80. Pileckis, S. & V. Monsevičius 1995: 272. Plata-Negrache, P. & Prendes-Ayala, C. 1981: 230. Pospischil, R. 2003: 4 (biology). Purrini, K. & Ormieres, R. 1979: 437. Ratti, E. 1997: 178. Šefrová, H. & Laštůvka, Z. 2005: 162. Szwalko, P. & Kubisz, D. 1994: 46 (*Tenebroides fuscus* Goeze, 1777; distribution). Teson, A. & Dagoberto, E. L. 1979: 127 (biology). Vogt, H. 1967: 16. Vogt, H. 1967: 16 (*Tenebriodes fuscus* Preyssler, 1790). Leschen, R. A. B. & Lackner, T. 2013: 301 (syn. *Trogosita affinis* White, 1846; New Zealand)

*Tenebroides metallescens* Reitter, 1875

Léveillé, A. 1910: 18; Brazil (AL)

*Tenebroides moerens* Sharp, 1891; Panama (AL)

Léveillé, A. 1910: 18

*Tenebroides mordax* Sharp, 1891; Costarica (AL)

Léveillé, A. 1910: 18

*Tenebroides murinus* Reitter, 1875; Colombia (AL)

Léveillé, A. 1910: 18

*Tenebroides muticus* Palisot de Beauvois, 1811 (*incertae sedis*); N America (JRB)

Léveillé, A. 1910: 18. Léveillé, A. 1888: 439 (syn. *Trogosita punctata* Dejean, 1836). Barron, J. R. 1971: 120 (*Trogosita punctata* Dejean, 1836; nomen nudum). Barron, J. R. 1971: 119. (incertae sedis, syn. with *Tenebroides nanus* Melsheimer, 1844 by Horn 1862)

*Tenebroides nanus* Melsheimer, 1844; E USA, Mexico (JRB)

Léveillé, A. 1910: 18. Barron, J. R. 1971: 98. Barron, J. R. 1971: 119. (syn. *Tenebroides muticus* Palisot de Beauvois, 1811 syn. by Horn 1862). Klausnitzer, B. 1996: 150. Kolibáč, J. 2006: 109

*Tenebroides nemosomiaeformis* Léveillé, 1905; Brazil (AL)

Léveillé, A. 1910: 18

*Tenebroides nigrocyaneus* Léveillé, 1905; Paraguay (AL)

Léveillé, A. 1910: 18

*Tenebroides nigroviridis* Léveillé, 1889; Mexico (AL)

Léveillé, A. 1910: 18

*Tenebroides oblongus* Sharp, 1891; Mexico, Panama (AL)

Léveillé, A. 1910: 18

*Tenebroides obtusus* Horn, 1862; Eastern coastal states of USA (JRB)

Léveillé, A. 1910: 18. Barron, J. R. 1971: 108

*Tenebroides occidentalis* Fall, 1910; W Canada and USA, Mexico (JRB)

Barron, J. R. 1971: 117. Fall, H. C. 1910: 128

*Tenebroides ocularis* Lewis, 1894; Japan (AL)

Léveillé, A. 1910: 18

*Tenebroides opacus* Reitter, 1875 (incertae sedis); N America (?), Colombia (JRB)

Léveillé, A. 1910: 18. Barron, J. R. 1971: 120 (incertae sedis)

*Tenebroides ornatus* Léveillé, 1889; Brazil (AL)

Léveillé, A. 1910: 18

*Tenebroides passeti* Léveillé, 1905; Brazil (AL)

Léveillé, A. 1910: 18

*Tenebroides patruelis* Reitter, 1875 (incertae sedis); Brazil, “Carol. mer.” (JRB)

Léveillé, A. 1910: 18. Barron, J. R. 1971: 120 (incertae sedis)

*Tenebroides politus* Sharp, 1891; Guatemala (AL)

Léveillé, A. 1910: 18

*Tenebroides pollens* Sharp, 1891; America centr. (AL)

Léveillé, A. 1910: 18

*Tenebroides pulchellus* Reitter, 1875; “Nov. Grenada” (AL)

Léveillé, A. 1910: 18

*Tenebroides pumilus* Léveillé, 1889; Colombia (AL)

Léveillé, A. 1910: 18

*Tenebroides punctatolineatus* Fairmaire, 1850; Polynesia (?) (AL)

Léveillé, A. 1910: 18

*Tenebroides punctulatus* Reitter, 1875; Cuba, Portorico (AL)

Léveillé, A. 1910: 18

*Tenebroides pusillimus* Mannerheim, 1843 (incertae sedis); USA: Alaska (JRB)

Léveillé, A. 1910: 18. Barron, J. R. 1971: 120 (incertae sedis)

*Tenebroides quadridens* Palisot de Beauvois, 1811; “Oware” (?) (AL)

Léveillé, A. 1910: 18

*Tenebroides quadriguttatus* Reitter, 1875; Brazil, Argentina (AL)

Léveillé, A. 1910: 18

*Tenebroides rectus* Wollaston, 1862; Canary Isl.: Lanzarote (JK)

Léveillé, A. 1910: 18. Kolibáč, J. 2007a: 365. Plata-Negrache, P. & Prendes-Ayala, C. 1981: 230

*Tenebroides reflexus* Reitter, 1875; Colombia (AL)

Léveillé, A. 1910: 18

*Tenebroides reitteri* Léveillé, 1889; Panama, Brazil (AL)

Léveillé, A. 1910: 18

*Tenebroides repetitus* Sharp, 1891; Mexico, Guatemala (AL)

Léveillé, A. 1910: 19

*Tenebroides ritsemae* Léveillé, 1889; Colombia, Costarica (AL)

Léveillé, A. 1910: 19

*Tenebroides ruber* Reitter, 1875; America centr., Brazil (AL)

Léveillé, A. 1910: 19

*Tenebroides rubromarginatus* Reitter, 1875; Brazil (AL)

Léveillé, A. 1910: 19

*Tenebroides ruficollis* Reitter, 1875; Bogota (AL)

Léveillé, A. 1910: 19

*Tenebroides rufipes* Léveillé, 1889; Brazil (AL)

Léveillé, A. 1910: 19

*Tenebroides rufiventris* Reitter, 1875; Colombia, Argentina (AL)

Léveillé, A. 1910: 19

*Tenebroides rufolimbatus* Léveillé, 1889; Mexico (AL)

Léveillé, A. 1910: 19

*Tenebroides rugosipennis* Horn, 1862; E USA to Arizona (JRB)

Léveillé, A. 1910: 19. Barron, J. R. 1971: 108 (syn. *Tenebroides arizonensis* Schaeffer, 1918; synonymized by Barron 1971)

*Tenebroides sallei* Sharp, 1891; Mexico (AL)

Léveillé, A. 1910: 19

*Tenebroides scaberrimus* Léveillé, 1905; Brazil (AL)

Léveillé, A. 1910: 19

*Tenebroides schaufussi* Reitter, 1875; Venezuela: Caracas (AL)

Léveillé, A. 1910: 19

*Tenebroides sculpturatus* Reitter, 1875; Brazil (AL)

Léveillé, A. 1910: 19

*Tenebroides semicylindricus* Horn, 1862; Eastern coast of USA to Mexico (JRB)

Barron, J. R. 1971: 109 (syn. *Tenebrioides subaenea* Reitter, 1875; synonymized by whom?). Barron, J. R. 1971: 109 (syn. *Tenebroides foveatus* Blatchley, 1917; synonymized by Barron 1971). Barron, J. R. 1971: 110 (syn. *Tenebroides helophorus* Sharp, 1891; synonymized by Schaeffer 1918; syn. uncertain)

*Tenebroides semicylindricus* Horn, 1862

Léveillé, A. 1910: 19

*Tenebroides sennevillei* Léveillé, 1889; America centr. (AL)

Léveillé, A. 1910: 19

*Tenebroides sericatus* Sharp, 1891; Guatemala (AL)

Léveillé, A. 1910: 19

*Tenebroides serraticollis* Léveillé, 1907; Argentina (AL)

Léveillé, A. 1910: 19

*Tenebroides sharpi* Léveillé, 1891; Panama (AL)

Léveillé, A. 1910: 19

Léveillé, A. 1910: 19 (syn. *Tenebroides bimaculatus* Sharp, 1891; syn by Léveillé 1910?) (AL)

*Tenebroides similis* Léveillé, 1905; Brazil

Léveillé, A. 1910: 19

*Tenebroides sinuatus* LeConte, 1861; W USA, Canada; to Kansas, Montana, Texas (JRB)

Léveillé, A. 1910: 19 (syn. *Tenebroides sinuatus* var. *californicus* Horn, 1862; synonymized by Barron 1971?). Barron, J. R. 1971: 91

*Tenebroides sonorensis* Sharp, 1891; SW USA, Mexico, Cuba (?) (JRB)

Léveillé, A. 1910: 19 (distribution in Cuba). Barron, J. R. 1971: 112 (syn. *Tenebroides debilis* Fall, 1910; synonymized by whom?). Dajoz, R. 1997: 42 (ecology)

*Tenebroides soror* Jacquelin du Val, 1857; USA: Florida, Bahamas, N Cuba (JRB)

Léveillé, A. 1910: 19. Barron, J. R. 1971: 101

*Tenebroides spectator* Sharp, 1891; Guatemala (AL)

Léveillé, A. 1910: 19

*Tenebroides steinheili* Reitter, 1875; Colombia (AL)

Léveillé, A. 1910: 19

*Tenebroides stultus* Léveillé, 1907; Argentina (AL)

Léveillé, A. 1910: 19

*Tenebroides sublaevis* Palisot de Beauvois, 1811; ‘Oware’ (AL)

Léveillé, A. 1910: 19

Note: species not mentioned by Barron (1971).

*Tenebroides subnigra* Palisot de Beauvois, 1811 (incertae sedis); USA: Pennsylvania (JRB)

Léveillé, A. 1910: 19 (*Tenebroidessubniger*; misspelling). Barron, J. R. 1971: 119 (incertae sedis)

*Tenebroides subplanus* Reitter, 1875; Mexico (AL)

Léveillé, A. 1910: 19

*Tenebroides subruber* Léveillé, 1899; Brazil (AL)

Léveillé, A. 1910: 19

*Tenebroides subvirescens* Léveillé, 1889; Brazil (AL)

Léveillé, A. 1910: 19

*Tenebroides sulcifrons* Jacquelin du Val, 1857; Cuba (AL)

Léveillé, A. 1910: 19

† *Tenebroides tenebrioides* Germain, 1837; Germany: Rott, Siebengebirge; Tertiary: Upper Oligocene (varA)

Léveillé, A. 1910: 19. Ponomarenko, A. G. & Kireichuk, A. G. 2005–2008: http://www.zin.ru/animalia/Coleoptera/rus/paleosy2.htm. Schmied, H. et al. 2009: 23

*Tenebroides tenuistriatus* Fall, 1910; USA: Colorado, New Mexico, Arizona; Mexico (JRB)

Barron, J. R. 1971: 114

*Tenebroides transversicollis* Jacquelin du Val, 1857; Cuba (AL)

Léveillé, A. 1910: 20

*Tenebroides turkestanicus* Ballion, 1870; Central Asia (JK)

Léveillé, A. 1910: 20. Kolibáč, J. 2006: 109. Kolibáč, J. 2007a: 365. Mamaev, B. M. 1976: 1654 (larva)

*Tenebroides undulatus* Sharp, 1891; Guatemala (AL)

Léveillé, A. 1910: 20

*Tenebroides viridescens* Léveillé, 1889; Brazil (AL)

Léveillé, A. 1910: 20

*Tenebroides yucatanicus* Léveillé, 1889; Yucatan, Honduras (AL)

Léveillé, A. 1910: 20

*Tenebroides zapotensis* Sharp, 1891; Guatemala (AL)

Léveillé, A. 1910: 20

*Tenebroides zunilensis* Sharp, 1891; Guatemala (AL)

Léveillé, A. 1910: 20

##### † Genus Thoracotes

Handlirsch, 1906

http://species-id.net/wiki/Thoracotes

Figs 24 , 25 ; Map 5

Thoracotes Handlirsch, A. 1906–1908: 438–439, Taf. XLI-9 (1906).

###### Type species.

*Thoracotes dubius* Handlirsch, 1906 [by monotypy]

Kolibáč, J. 2006: 121 (classification). Ponomarenko, A. G. 1985: 68. Ponomarenko, A. G. 1990: 74. Ponomarenko, A. G. & Kireichuk, A. G. 2005–2008: http://www.zin.ru/animalia/Coleoptera/rus/paleosy2.htm. Schmied, H. et al. 2009: 24

###### Description

(*Thoracotes dubius*): “*Ein 8 mm langer Käfer von ähnlicher Gestalt wie* Parnidium *Geinitzi. Der Prothorax is aber anders geformt un nähert sich mehr der Kreisform, Auch der Kopf scheint anders gewesen zu sein. Flügeldecken punktiert, 3,5 mal so lang als breit. Geinitz vergleicht diese Form mit Latridiites Schaumi, mit dem sie allerdings auch einige Ähnlichkeit hat.*”

This is potentially the oldest fossil record of Trogossitidae. Unfortunately, its description is inadequate and the illustration very poor. It is unclear to me why two more Mesozoic species from Russia were assigned to this dubious genus. It was not indicated in the original papers describing the two new species if the type of *Thoracotes dubius* was studied *in situ* (Ponomarenko 1985, 1990). The specimen appears to be housed in Ernst Moritz Arndt University of Greifswald, Germany. (A facsimile of the original description and illustration in Figs 24, 25.)

###### Description

(*Thoracotes glabrus*, translation from Russian). “*Head sligthtly longer than wide, narrowed in front of eyes; eyes relatively large, situated at sides of head; cheeks*[genae] *short; temples* [tempora] *slightly shorter than eyes. The first antennomere large, second transverse, third 1.5 times longer than second, fourth to seventh as long as wide, eighth longer than wide, nineth to eleventh asymmetric. Pronotum with lateral margins rounded, its corners not acute, 1.5 times longer than wide. Front coxae not large, spherical, with exposed trochantins. Prosternal process short and blunt. Middle coxae transverse, nearly touching each other. Metathorax trapezoidal, narrowed anteriad, its length 1.8 times lesser than wide at hind* [basal] *margin. Hind coxae touch each other, transverse, not concave posteriorly* [sic] *and without coxal plates, at lateral sides shorter than at mid-point. Last abdominal sternite distinctly longer than penultimate one. Ovipositor long, with sclerotized palpifers* [sic] *and pairs of appendages* [coxitae] *with pubescent cerci* [styli]. *Legs relatively long, tibiae and tarsi thin. Elytra smooth. Length of beetle 3.6, width 1.6; length of elytra 2.3 mm.*” “*The beetle of Pavlovka distinctly smaller (length 3.0, width 1.0 mm).*” (Ponomarenko 1990: 74.)

###### Distribution.

Germany: Dobbertin in Mecklenburg, Russia: central Siberia; Transbaikalia: Pavlovka, Onokhoy; Mesozoic: from Lower to Upper Jurassic.

###### Species:

† *Thoracotes dubius* Handlirsch, 1906; Germany; Lower Jurassic: Upper Lias (varA)

Kolibáč, J. 2006: 121 (classification). Schmied, H. et al. 2009: 24

† *Thoracotes glabrus* Ponomarenko, A., 1990; Russia: Transbaikalia; Upper Jurassic (varA)

Ponomarenko, A. G. 1990: 74. Kolibáč, J. 2006: 121. Ponomarenko, A. G. & Kireichuk, A. G. 2005–2008: http://www.zin.ru/animalia/Coleoptera/rus/paleosy2.htm. Schmied, H. et al. 2009: 24

† *Thoracotes sibiricus* Ponomarenko, 1985; Russia: Siberia; Middle Jurassic (varA)

Ponomarenko, A. G. 1985: 68. Kolibáč, J. 2006: 121. Ponomarenko, A. G. & Kireichuk, A. G. 2005–2008: http://www.zin.ru/animalia/Coleoptera/rus/paleosy2.htm. Schmied, H. et al. 2009: 24

##### † Tribe Lithostomatini

Kolibáč & Huang, 2008

Lithostomini Kolibáč, J. & Huang, D.-Y. 2008: 142 (as ). Bouchard, P. et al. 2011: 57 (emendation to Lithostomatini)

###### Type genus.

*Lithostoma* Martynov, 1926 [by monotypy and author’s designation]

Kolibáč, J. & Huang, D.-Y. 2008: 142. Yu, Y. et al. 2012: 250

###### Remarks.

This fossil differs from all other Mesozoic Trogossitidae described to date. If Martynov (1926) interpreted the shapes of the head and antennal segments well, it is the first known member of Trogossitidae without a distinct antennal club and with the head narrowed towards its base. The following features of Trogossitidae appear in the fossil: (1) general shape and size of body, (2) distinctly flattened sides of pronotum and elytra, (3) double rows of punctures/tubercles among elytral carinae, (4) robust bidentate mandibles, (5) extremely large scapus, and (6) dilated antennal segments with what are perhaps sensorial fields in the enlarged parts of each segment. The classification within Trogossitinae is based on the presence of the sensorial fields in the enlarged parts of the antennomeres alone. Small tubercles occurring in pronotum and elytra are known in trogossitine genera *Calitys* and *Phanodesta* only; no peltine representative possesses such structures. The tribe differs from the recent and fossil members of Trogossitinae in broadly oval body (this occurs in some Gymnochilini only), pronotum narrowed anteriad, antennae without conspicuous club and asymmetrical segments in flagellum, head narrowed towards base. The shape of antennal segments 10 and 11 is unknown because they are missing (only a trace of segment 10 is visible). The antennae may be only 10-segmented with the last segment enlarged (as in e.g. Egoliini). The new tribe is probably isolated from other tribes of Trogossitinae and may be considered a sister group to them. The ventral part of the fossil is unfortunately unknown, so a classification of *Lithostoma* remains uncertain, chiefly based on the distinctly enlarged scapus.

On the other hand, the concept of Lithostomatini was justifiably called into question by Yu et al. (2012) who argued that insufficient morphological information existed for the establishment of a higher taxon.

##### † Genus Lithostoma

Martynov, 1926

http://species-id.net/wiki/Lithostoma

Map 6

Lithostoma Martynov, A. V. 1926: 13 (in Russian), 32 (in English)

###### Type species.

*Lithostoma expansum* Martynov, 1926 [by monotypy]

Kolibáč, J. & Huang, D.-Y. 2008: 142 (Remarks, English description and translation of Russian description). Ponomarenko, A. G. & Kireichuk, A. G. 2005–2008: http://www.zin.ru/animalia/Coleoptera/rus/paleosy2.htm. Schmied, H. et al. 2009: 26

###### Original description of the genus

(translation from Russian). “*Head free, dilated between the eyes* [Russ.: and narrowed backwards]; *mandibulae strong, sharply bent inwards, with perhaps two teeth at the apices; antennae resembling those in living Ostomatinae* [Russ.: in Ostoma and other genera]; *basal joint large, bulbous anteriorly, second much smaller, third still smaller, short, fourth joint of the same width but elongated, 5–7th joints elongated but becoming gradually thicker towards the tip* [Russ.: joints 10 and 11 not preserved], *last 8–11 joints only slightly thicker, without distinct apical club (clavus). Pronotum broadening posteriorly, apparently furnished with marginal dilatations* [Russ.: as in Ostoma]; *covered all over with numerous point-like pits. Elytra broad, rounded at postero-lateral margins* [Russ.: as in Ostoma], *with perhaps 8 longitudinal stripes, each containing two rows of raised black points; intermediate narrow stripes barely elevated; both marginal dilatations also with pits and points* [Russ.: Body size about 6 mm.].” (Martynov 1926: 32.)

###### Original description of the species.

English text: “*Head and antennae as in generic description; sides of pronotum convex, points distinct; elytra broad, rounded at the postero-lateral margins; marginal dilatations rather broad; the dividing stripes not elevated. Length of the body 6 mm.*” (Martynov 1926: 32)

Translation of Russian text: “*Head free, strongly projecting anteriorly (partly artificial condition in compressed specimen). Mandibles robust, left mandible bidentate (right inconspicuous). Antennae as described above, each joint weakly dilated at apex; each dilated area with thin* [sic], *dark, round rim. Final two joints torn off, only a trace of joint 10 present. Pronotum widened towards base, with shallow punctures interspersed with small tubercles. Elytra wide, rounded apically and dorsally* [sic]; *flattened sides well-developed and probably lighter than dark brown convex portion of elytra. Elytra, including flattened sides, with rows of well-developed small, black tubercles* [orig.: “convex punctures”]; *carinae among them not higher than tubercles. Length of body from anterior margin of labrum to apex of elytra – 6 mm.*” (Martynov 1926: 13.)

###### Note:

The Russian and English texts vary somewhat from one another; the Russian is more comprehensive.

###### Distribution.

Southern Kazakhstan: paper-shales near Galkino (approximately 42.15N, 70.02E), Chimkentskaya oblast district. Mesozoic: Upper Jurassic, probably Oxfordian.

**Map 6.**
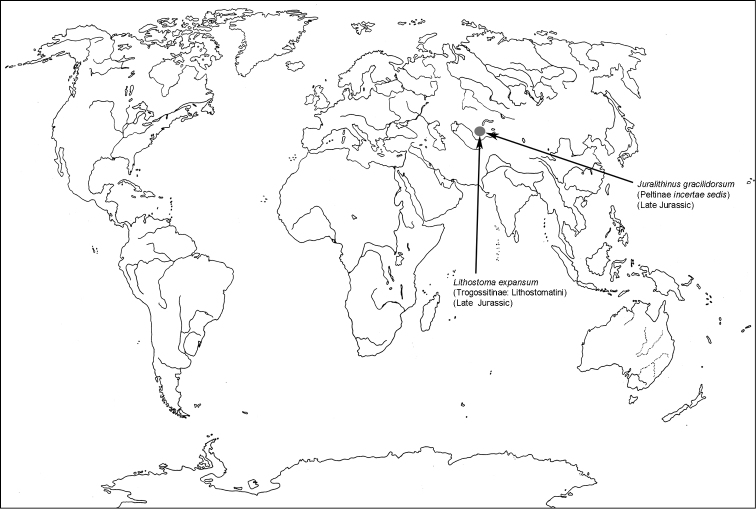
A distribution of the tribe Lithostomatini and the genus † *Juralithinus* (Peltinae *incertae sedis*).

###### Species:

† *Lithostoma expansum* Martynov, 1926; Kazakhstan; Upper Jurassic (varA)

Martynov, A. V. 1926: 13 (in Russian), 32 (in English). Kolibáč, J. & Huang, D.-Y. 2008: 143 (Remarks, English description and translation of Russian description). Ponomarenko, A. G. & Kireichuk, A. G. 2005–2008: http://www.zin.ru/animalia/Coleoptera/rus/paleosy2.htm. Schmied, H. et al. 2009: 26

#### Subfamily Peltinae

Latreille, 1806

Peltinae Latreille, P. A. 1806: 8, 1825.
Peltinae Crowson, R. A. 1955: 82. Zherichin, V. V. 1978: 78 (unidentified remnants of two fossils, Turon; Kazachstan: Kzyl-Dzhar). Burakowski, B., Mroczkowski, M. & Stefanska, J. 1986: 121. Lawrence, J. F. & Newton, A. F., Jr. 1995: 868 (Kirby, W. 1837: 104 is considered the author of the family rank name). Klausnitzer, B. 1996: 145. Kolibáč, J. 2006: 125 (diagnosis). Barron, J. R. 1971: 11.

##### Key to tribes of Peltinae

**Table:** 

1	Antenna 8-segmented; body surface with stout scale-like setae; wingless species with convex body	Colydiopeltini
–	Antenna 10- or 11-segmented; body surface without scales or scale-like setae; winged or wingless species with flat or convex or conglobate body	2
2	Body flat, relatively large (more than 8 mm). Larva without distinct urogomphi	Peltini
–	Body convex or conglobate, smaller (mostly less than 7 mm). Larva with distinct urogomphi	3
3	Adult: mandible without mola, with membranous penicillus; front coxae distinctly projecting. Larva: mandible without mola, with tridentate lacinia mobilis	Phloiophilini
–	Adult: mandible with mola, membranous penicillus absent; front coxae indistinctly projecting. Larva: mandible with mola, without distinct lacinia mobilis	Thymalini

#### Peltinae *incertae sedis*

##### † Genus Juralithinus

Kireichuk & Ponomarenko, 1990

http://species-id.net/wiki/Juralithinus

Map 6

Peltidae Kireichuk, A. G. & Ponomarenko, A. G. 1990: 79 ( : Meligethiellinae).

###### Type species:

*Juralithinus gracilidorsum* Kireichuk & Ponomarenko, 1990 [by monotypy and original designation]

Kolibáč, J. 2006: 126 (Thymalini). Ponomarenko, A. G. & Kireichuk, A. G. 2005–2008: http://www.zin.ru/animalia/Coleoptera/rus/paleosy2.htm. Schmied, H. et al. 2009: 24

###### Remarks.

The genus was originally described in Peltidae and classified within the extinct subfamily Meligethiellinae along with *Meligethiella* and *Ostomalynus* (Kireichuk and Ponomarenko 1990). I accepted the classification within Trogossitidae: Peltinae. However, I synonymized Meligethiellinae and moved all three genera into the extant Thymalini (Kolibáč 2006). Features of *Juralithinus* agree with a general definition of Peltinae, although after re-examination of its description and illustrations, I found the evidence too weak to classify the genus within any of the extant tribes. Therefore, it is listed herein as Peltinae *incertae sedis*. It may be considered an ancestor of Thymalini or Peltini or of both tribes

###### Original description of the genus

(translation from Russian). “*Diagnosis: Body conspicuously longitudinal, with prothorax relatively elongate and narrow, coxae narrowly separated in all pairs of legs; metathorax without femoral lines* [paracoxal sutures? – they are drawn in *l.c.*, Fig. 1a]; *elytra distinctly wider than pronotum; first visible abdominal sternite approximately as large as the second to fourth, the last sternite longer.*”

###### Original description of the species

(translation from Russian). “*Elongate-oval beetle* [sic]. *Head with moderately protruded bidentate mandibles, very small transverse mentum and developed gular sutures. Pronotum with anterior margin deeply emarginate and widely rounded anterior corners. Transverse front coxae strongly* [distinctly] *narrowly separated, process between them not observed. Mesothorax convex at centre, middle coxae narrowly separated. Mesonotum wide, with widely rounded scutellum. Metathorax with conspicuous discrimen and paracoxal sutures, outer apices of metepisterna strongly anteriorly projecting. Hind coxae oval, very narrowly separated and obliquely situated towards centre. Elytra, with medium sized epipleurons, seem not to cover abdomen perfectly; their apices possibly artificially* [sic] *broken off. Femora medium-dilated, all with oval outlines. Tibiae weakly dilated towards apices, in middle, strangely enough, relatively thin with elongate apodemes* [orig.: arrows] *at apex. Hind tarsi relatively long, comprised of simple segments* [i.e., without lobes]. *Length – 9.3; width – 4.6; length of elytra – 2.3* [mm].”

###### Distribution.

Kazakhstan: Chimkentskaya obl., Mikhailovka; Mesozoic: Upper Jurassic, Karabastayskaya formation.

###### Species:

† *Juralithinus gracilidorsum* Kireichuk & Ponomarenko, 1990; Kazakhstan; Upper Jurassic (varA)

Kireichuk, A. G. & Ponomarenko, A. G. 1990: 80. Kolibáč, J. 2006: 126. Ponomarenko, A. G. & Kireichuk, A. G. 2005–2008: http://www.zin.ru/animalia/Coleoptera/rus/paleosy2.htm. Schmied, H. et al. 2009: 24

##### † Genus Sinopeltis

Yu, Leschen, Ślipiński, Ren & Pang, 2012

http://species-id.net/wiki/Sinopeltis

Map 6

Sinopeltis Yu, Y., Leschen, R. A. B., Ślipiński, A., Ren, D. & Pang, H. 2012: 246.

###### Type genus.

*Sinopeltis jurassica* Yu, Leschen, Ślipiński, Ren & Pang, 2012

###### Remarks.

This genus, containing two species, has only recently been established. The fossils are well preserved, with both part and counterpart. The species are relatively large, body shape perfectly appropriate to Peltinae or Lophocaterinae. The eyes are distinctly elevate, much more so than those in extant Ancyronini. The 3-segmented antennal club of *Sinopeltis jurassica* is “weakly asymmetrical” (quite symmetrical in the original picture), that of *Sinopeltis amoena* is “strongly asymmetrical” (inconspicuously so in the picture). The mesocoxae appear contiguous (unknown state in Trogossitidae) in *Sinopeltis jurassica*, whereas they are narrowly separated in *Sinopeltis amoena*. Both discrepancies mentioned may be the results of different positions of body parts (coxae, antennae) assumed during fossilization. Unfortunately, neither the ends of the tibiae nor the tarsi and mouthparts are described in either species, which leads to a lack of direct evidence for classification. Both *Sinopeltis jurassica* and *Sinopeltis amoena* are about 165 million years old and belong, if their classification is correct, together with species of *Thoracotes*, among the oldest known fossil members of Trogossitidae.

###### Description.

“*Body broadly ovoid and parallel-sided. Antenna distinctly clubbed with antennomeres symmetrical* [S. jurassica: “*weakly asymmetrical*”; S. amoena: “*strongly asymmetrical*”]; *antennal insertions visible in dorsal view. Eyes convex. Frontoclypeal suture present. Antennal grooves present and parallel. Anterior pronotal angles well developed and subrounded. Mesoventrite not vaulted. Mesocoxae widely separated* [see “note” in Remarks]. *Metaventrite lacking axillary space and metakatepisternal suture. Metacoxae not excavate and narrowly separated. Elytra with seriate punctation (in one fossil). Abdominal ventrite 1 about as long as 2, intercoxal process narrow. Body length 7.5–7.6 mm, width 4.5–4.8 mm.*” (Diagnosis of the genus according to Yu, Leschen, Ślipiński, Ren & Pang 2012.)

###### Distribution.

China: Inner Mongolia, Daohugou. Middle Jurassic, Jiulongshan formation.

###### Species:

† *Sinopeltis jurassica* Yu, Leschen, Ślipiński, Ren & Pang, 2012; China: Inner Mongolia; Middle Jurassic (AD)

Yu, Y., Leschen, R. A. B., Ślipiński, A., Ren, D. & Pang, H. 2012: 247

† *Sinopeltis amoena* Yu, Leschen, Ślipiński, Ren & Pang, 2012; China: Inner Mongolia; Middle Jurassic (AD)

Yu, Y., Leschen, R. A. B., Ślipiński, A., Ren, D. & Pang, H. 2012: 247

##### Tribe Peltini

Latreille, 1806

Peltini Latreille, P. A. 1806: 8, 1825.

###### Type genus.

*Peltis* O. F. Müller, 1764

Hunt, T. et al. 2007: 1915 (Peltinae) (molecular phylogeny). Kolibáč, J. 2006: 125 (diagnosis, phylogeny). Kolibáč, J. 2007a: 366. Lawrence, J. F. & Newton, A. F., Jr. 1995: 868 (Kirby, W. 1837: 104 is considered the author of the family rank name). Ślipiński, S. A. 1992: 442 (Peltinae)

###### Remarks.

The single genus *Peltis* exhibits a noteworthy mixture of the both advanced and primitive morphological features. However, some of the derived, especially larval, character states may considered as various kinds of reduction. A few species of *Peltis* are highly adapted to a hidden way of life under tree bark or in rotten wood: they are flattened and slow-moving, their robust mandibles have distinct mola, and the larval urogomphi are strongly reduced. The outer appearance is similar to that in *Calitys*, while some details of both adult and larval morphology (for example the gular appendages in the larval cranium) resemble *Thymalus*. The synonymization of *Zimioma* with *Peltis* is undeniable. Apart from body size, there is no morphological character to distinguish between the two genera.

##### Genus Peltis

O. F. Müller, 1764

http://species-id.net/wiki/Peltis

**Figure 9.**
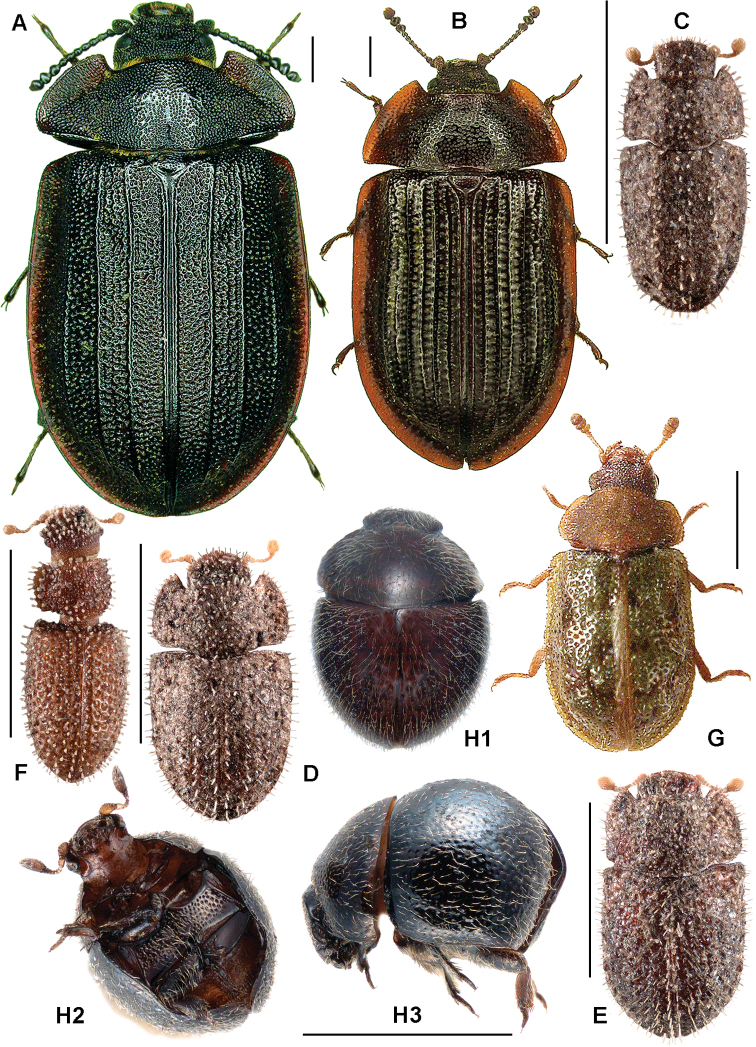
**A** *Peltis* (syn. *Zimioma*, *Ostoma*) *grossa* **B** *Peltis* (syn. *Ostoma*) *ferruginea* **C** *Colydiopeltis loebli* **D** *Colydiopeltis compactum* **E** *Colydiopeltis burckhardti* **F** *Parapeltis australicum* **G** *Protopeltis viridescens* **H** *cf.* *Globorentonium plaumanni*, Brazil (**1–3**: three different specimens from the same locality).

**Map 7.**
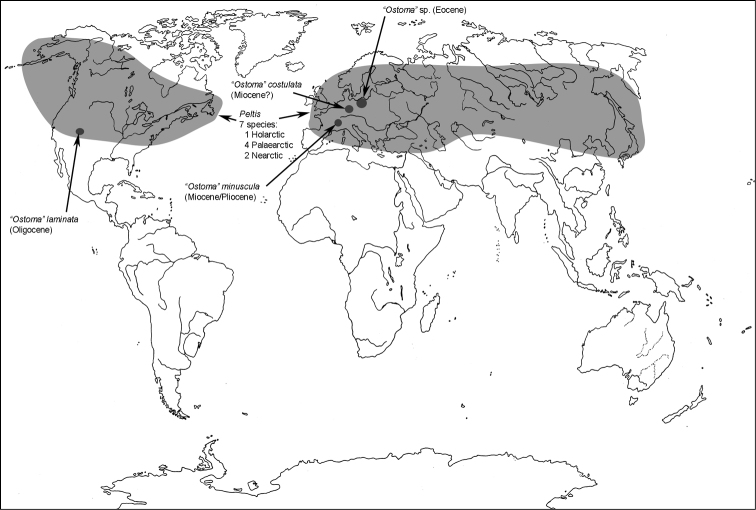
A distribution of the tribe Peltini.

Figs 2 , 9 , 15 ; Map 7

Peltis Müller, O. F. 1764: 13.

###### Type species.

*Silpha grossa* Linnaeus, 1758 [designated by Hope 1840]

Bacianskas, V. 2009: 30 (biology). Barron, J. R. 1971: 24. Barron, J. R. 1996: 193 (Nearctic species). Kolibáč, J. 2005: 76. Kolibáč, J. 2006: 111. Kolibáč, J. 2007a: 366. Noreika, N. 2009: 68 (distribution). Spahr, U. 1981: 74 (amber and copal fossils)

*Gaurambe* C. G. Thomson, 1862 [type species: *Silpha ferruginea* Linnaeus, 1758]

Barron, J. R. 1971: 24. Kolibáč, J. 2007a: 366. Lafer, G. Sh. 1992: 83. Silfverberg, H. 1978: 117

*Ostoma* Laicharting, 1781 [type species: *Silpha ferruginea* Linnaeus, 1758]

Léveillé, A. 1910: 30. Downie, N. M. & Arnett, R. H. Jr. 1996: 935 (key). Barron, J. R. 1971: 23. Crowson, R. A. 1964a: 295 (*Peltis* Kugel, 1791). Kolibáč, J. 2005: 76 (synonymized). Kolibáč, J. 2007a: 366. Larsson, S. G. 1978: 150 (fossil, Baltic amber). Lafer, G. Sh. 1992: 83. Reitter, E. 1876: 61

*Zimioma* Gozis, 1886 [type species: *Silpha grossa* Linnaeus, 1758]

Léveillé, A. 1910: 29 [*Ostoma (Zimioma)* Gozis, 1886]. Kolibáč, J. 2007a: 366

###### Description.

Body size: about 7.5–23.0 mm. Body shape flat. Gular sutures wide, subparallel. Frontoclypeal suture broadly emarginate. Frons: longitudinal groove or depression absent. Cranium ventrally: tufts of long setae at sides absent. Submentum: ctenidium absent. Antennal groove present. Eyes: size moderate. Eyes number: two. Epicranial acumination moderate. Lacinial hooks: one. Galea: shape elongate. Galea: ciliate setae absent. Mediostipes-Lacinia partially fused. Palpifer: outer edge even. Mandibular apical teeth number: two, horizontally situated. Mola present. Penicillus (at base) absent. Pubescence above mola or cutting edge absent. Ventral furrow absent. Basal notch moderate. Labrum-Cranium not fused. Epipharyngial sclerite absent. Lateral tormal process: projection curved downwards, processes with bridge (*Peltis*). Ligula: ciliate setae absent. Ligula membranous, not retroflexed, deeply emarginate. Hypopharyngeal sclerite consisting of two separate parts. Antenna 11-segmented. Antennal club symmetrical, sensorial fields absent. Front coxal cavities externally open, internally open. Pronotum transverse. Prepectus present. Middle coxal cavities open. Elytra: long hairs absent. Epipleuron moderate. Elytral interlocking mechanism absent, carinae conspicuous. Elytral punctation regular, scales absent. Wing: radial cell oblong (or reduced), wedge cell small (*Peltis*), cross vein MP3-4 present, cross vein AA1+2-3+4 absent. Front tibiae: spines along side moderate. Hooked spur present. Claws: denticle absent. Spiculum gastrale absent. Tegmen composed of three parts. Coxitae divided.

Larva: Frontal arms Y-shaped. Epicranial stem present. Endocarina absent. Gular sutures inconspicuous. Gula: anterior apodemes present. Paragular sclerites absent. Hypostomal rods present. Stemmata number: 3. Mandibular apical teeth number: two, horizontally situated. Lacinia mandibulae absent. Mola reduced. Maxillary palpi 3-segmented. Palpifer present. Pedunculate seta absent. Mala simple. Mala: bidentate protrusion present. Cardo-Stipes not fused. Cardo: size much smaller than stipes. Ligula present. Labial palpi 2-segmented. Prementum in two parts, anterior margin even. Torma: two separate lateral sclerites. Antennal joints 1 and 2 elongate. Sensory appendix very small. Thoracic sclerites pattern (dorsally) 0+0+0. Thoracic sclerites pattern (ventrally) 0+0+0. Trochanter oblong. Abdominal segment IX not divided. Tergite IX flat. Urogomphi minute; median process absent.

###### Biology.

Fungivorous. The adults live under the bark of dead or dying deciduous and coniferous trees and feed on fungi. Larvae can be found in rotten or decaying wood, for example at the base of old trees or inside stumps.

###### Distribution.

Holarctic temperate zones: North America from Arizona to Alaska, Europe including British Isles and Scandinavia, Siberia, Korea, Japan.

###### Species:

*Peltis columbiana* Casey, 1924; widespread USA, Canada (from Alaska) (JRB)

Barron, J. R. 1971: 30

† *Peltis constulata* Heyden, 1862; Germany: Rott, Siebengebirge; Tertiary: Upper Oligocene (varA)

Ponomarenko, A. G. & Kireichuk A. G. 2005–2008: http://www.zin.ru/animalia/Coleoptera/rus/paleosy2.htm. Schmied, H. et al. 2009: 24

*Peltis ferruginea* Linnaeus, 1758; Europe, Siberia, Japan, North Korea, USA, Canada (JK)

Léveillé, A. 1910: 30 (*Ostoma*). Bahillo de la Puebla P. & López-Colón, J. I. 1999: 13 (*Ostoma*). Sarikaya, O. & Avci, M. 2009: 129 (*Ostoma*)

Barron, J. R. 1971: 28 (syn. *Ostoma cassidoides* Lepechin, 1774); Russia (JRB)

Barron, J. R. 1971: 28 (syn. *Ostoma cimicoides* Degeer, 1774; not binominal). Barron, J. R. 1971: 28 (*Ostoma*)

Barron, J. R. 1971: 28 (syn. *Ostoma fraternus* Randall, 1838); USA: Maine (JRB)

Barron, J. R. 1971: 28 (syn. *Ostoma nigricans* Dalla Torre, 1879); Oberösterreich (JRB)

Barron, J. R. 1971: 28 (syn. *Ostoma nigrina* Casey, 1916); Canada: British Columbia (JRB)

Barron, J. R. 1971: 28 (syn. *Ostoma rubicunda* Laicharting, 1781); Germania (JRB)

Barron, J. R. 1971: 28 (syn. *Ostoma septentrionalis* Randall, 1838); USA: Maine (JRB)

Borowiec, L. 1983: 13 (*Ostoma*). Burakowski, B. et al. 1986: 122 (*Ostoma*)

Klausnitzer, B. 1976: 7 (*Ostoma*). Klausnitzer, B. 1996: 155 (*Ostoma*, larva). Kolibáč, J. 1993a: 21 (*Ostoma*). Kolibáč, J. 1993b: 90 (*Ostoma*). Kolibáč, J. 1999b: 12 (*Ostoma*). Kolibáč, J. 2006: 108 (larva). Kolibáč, J. 2007a: 366 (syn. *Peltis cimicoides* DeGeer, 1774). Kolibáč, J. 2007a: 366 (syn. *Peltis fraterna* Randall, 1838). Kolibáč, J. 2007a: 366 (syn. *Peltis nigricans* Dalla Torre, 1879). Kolibáč, J. 2007a: 366 (syn. *Peltis nigrina* Casey, 1916). Kolibáč, J. 2007a: 366 (syn. *Peltis rubicunda* Laicharting, 1781). Kolibáč, J. 2007a: 366 (syn. *Peltis septentrionalis* Randall, 1838). Krasutskii, B. V. 1996: 274 (*Ostoma*). Lafer, G. Sh. 1992: 84 (*Ostoma*). Mitter, H. 1998: 561 (*Ostoma*). Nakane, T. et al. 1963: 181 (*Ostoma*). Pileckis, S. & V. Monsevičius 1995: 272 (*Ostoma*). Reitter, E. 1876: 62 (*Ostoma*). Vogt, H. 1967: 17 (*Ostoma*)

*Peltis gigantea* Reitter, 1882; East Siberia, Far East, Japan, China: Northeast Territory (JK)

Léveillé, A. 1910: 29 (*Ostoma* subgen. *Zimioma*). Esaki, T. et al. 1951: 1062 (*Ostoma*). Kolibáč, J. 2007a: 366. Lafer, G. Sh. 1992: 84 (*Zimioma*). Nakane, T. et al. 1963: 181 (*Zimioma*)

*Peltis grossa* Linnaeus, 1758; Europe (JK)

Léveillé, A. 1910: 29 (*Ostoma* subgen. *Zimioma*)

Léveillé, A. 1910: 30 (syn. *Ostoma (Zimioma) lunata* Fabricius, 1787); Europe (AL)

Bahillo de la Puebla P. & López-Colón, J. I. 1999: 13 (*Zimioma*). Sarikaya, O. & Avci, M. 2009: 129 (*Ostoma*). Borowiec, L. 1983: 12. Burakowski, B. et al. 1986: 121 (*Zimioma*). Cunev, J. 1999: 76. Fjellberg, A. & Hansen, S. O. 1997: 77 (biology). Karalius, S. & Monsevičius, V. 1992: 5 (distribution). Klausnitzer, B. 1976: 7 (*Zimioma*). Klausnitzer, B. 1978: 176. Klausnitzer, B. 1996: 156 (larva). Kolibáč, J. 1993a: 21. Kolibáč, J. 1993b: 90. Kolibáč, J. 2005: 139 (redescription). Kolibáč, J. 2007a: 366. Krasutskii, B. V. 2006: 763 (biology). Mamaev, B. M. 1976: 1656 (larva). Mitter, H. 1998: 560 (*Zimioma*). Pileckis, S. & Monsevičius, V. 1995: 272. Ratti, E. 1997: 178. Reitter, E. 1876: 62 (*Ostoma*). Šablevičius, B. & Ferenca, R. 1995: 146. Svitra, G. & Aliukonis, A. 2009: 72 (distribution, biology). Vogt, H. 1967: 17 (*Zimioma*)

*Peltis jakowlewi* Semenov, 1898; Russia: South European Territory (JK)

Léveillé, A. 1910: 30 (*Ostoma* subgen. *Zimioma*). Kolibáč, J. 2007a: 366

† *Peltis laminata* Wickham, 1910; USA: Colorado, Florissant; Tertiary: Lower Oligocene (JRB, varA)

Barron, J. R. 1971: 120. Ponomarenko, A. G. & Kireichuk, A. G. 2005–2008: http://www.zin.ru/animalia/Coleoptera/rus/paleosy2.htm. Schmied, H. et al. 2009: 24. Wickham, H. F. 1910: 48

† *Peltis minuscula* Pilton, 1935; France: Cantal; Tertiary: Upper Miocene, Messinian (varA)

Deuve, P. 1988: ii (exact reference unknown). Ponomarenko, A. G. & Kireichuk, A. G. 2005–2008: http://www.zin.ru/animalia/Coleoptera/rus/paleosy2.htm. Pilton, 1935: ii (exact reference unknown). Schmied, H. et al. 2009: 24

*Peltis pippingskoeldi* Mannerheim, 1852; western states of USA, Canada: British Columbia (JRB)

Léveillé, A. 1910: 31 (*Ostoma*). Barron, J. R. 1971: 25 (*Ostoma*). Dajoz, R. 1997: 44 (*Ostoma*) (biology). Reitter, E. 1876: 62 (*Ostoma*)

*Peltis valida* Lewis, 1894; Japan (JK)

Léveillé, A. 1910: 30 (*Ostoma* subgen. *Zimioma*). Kolibáč, J. 2007a: 366

##### Tribe Colydiopeltini

Kolibáč, 2006

###### Type genus.

*Colydiopeltis* Ślipiński, 1992

Kolibáč, J. 2006: 126

###### Remarks.

A record by Ivan Löbl and Daniel Burckhardt (Muséum d’Histoire Naturelle Genève) of three minute wingless *Colydiopeltis* species from a forest litter in Thailand, together with their scientific processing (Ślipiński 1992), was one of the most surprising discoveries within Cleroidea to occur in recent decades, rather like the discovery of the rentoniins in the 1960s. The latter author also added a second genus, *Parapeltis*, extracted from litter in Queensland, and he originally classified the two genera together with *Larinotus* within Larinotini (Ślipiński 1992). In a 2006 paper, I split the latter tribe into the monotypic Larinotini related to Egoliini and the newly-established Colydiopeltini. While the nominotypical *Colydiopeltis* shares basic thoracic and mouthpart characters with other trogossitids, *Parapeltis* shows several unusual features: (1) extended clypeus, (2) maxillary lacinia without hooked spine(s), (3) procoxal cavities externally closed, (4) all coxae (especially pro- and metacoxae) short and small (metacoxae do not reach elytral margin), (5) metepisterna extremely wide (see excellent drawings and description by Ślipiński *l.c.*). Labrum, aedeagus and tarsi are of common “trogossitid” structure, although some of these features may be shared with certain non-cleroid Cucujiformia. On the other hand, it should be noted that aptery can have a profound influence on thoracic morphology. I have never studied *Parapeltis australicum*, the single species, first-hand, so the classification here follows that by Ślipiński (1992).

###### Key to genera

**Table:** 

1	Rather elongate species, pronotum narrower than elytra; front coxal cavities externally closed; mandible with two apical teeth	*Parapeltis*
–	More compact species, pronotum at base as wide as elytra; front coxal cavities externally open; mandible with one apical tooth	*Colydiopeltis*

##### Genus Colydiopeltis

Ślipiński, 1992

http://species-id.net/wiki/Colydiopeltis

Figs 9 , 16 ; Map 8

Colydiopeltis Ślipiński, S. A. 1992: 444.

###### Type species.

*Colydiopeltis burckhardti* Ślipiński, 1992 [by original designation]

Kolibáč, J. 2005: 50. Kolibáč, J. 2006: 111 (phylogeny)

###### Description.

Body size: 1.5–2.0 mm. Body shape convex (not conglobate). Gular sutures wide, convergent at apex. Frontoclypeal suture absent. Frons: longitudinal groove or depression absent. Cranium ventrally: tufts of long setae at sides absent. Submentum: ctenidium absent. Antennal groove present. Eyes: size moderate. Eyes number: two. Epicranial acumination moderate. Lacinial hooks: two or 1. Galea: shape very small. Galea: ciliate setae absent. Mediostipes-Lacinia fused together. Palpifer: outer edge even. Mandibular apical teeth number: one. Mola present. Penicillus (at base) present (fine, often membranous). Pubescence above mola or cutting edge present. Ventral furrow absent. Basal notch shallow or absent. Labrum-Cranium not fused. Epipharyngial sclerite absent. Lateral tormal process: projection projection curved upwards (*Colydiopeltis*). Ligula: ciliate setae absent. Ligula membranous, not retroflexed, weakly emarginate. Hypopharyngeal sclerite absent. Antenna 8-segmented, sensorial fields absent. Front coxal cavities externally open, internally open. Pronotum transverse. Prepectus present. Middle coxal cavities open. Elytra: long hairs absent. Epipleuron wide. Elytral interlocking mechanism absent, carinae reduced. Elytral punctation regular, scales present. Front tibiae: spines along side reduced. Hooked spur absent, apical spurs not hooked or weakly hooked. Claws: denticle absent. Parasternites number along ventrites III–VII: two. Spiculum gastrale present. Tegmen composed of three parts. Coxitae undivided.

###### Biology.

Unknown. Species of the genus were collected from forest litter by mass-sampling methods in the dry season.

###### Distribution.

Thailand.

**Map 8.**
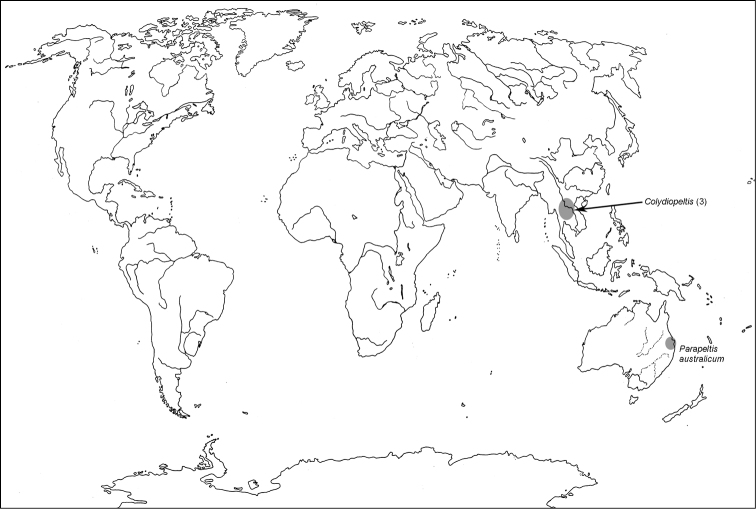
A distribution of the tribe Colydiopeltini.

###### Species:

*Colydiopeltis burckhardti* Ślipiński, 1992; Thailand: Chiang Mai (AD)

Ślipiński, S. A. 1992: 449. Kolibáč, J. 2005: 49

*Colydiopeltis compactum* Ślipiński, 1992; Thailand: NE of Bangkok (AD)

Ślipiński, S. A. 1992: 450

*Colydiopeltis loebli* Ślipiński, 1992; Thailand: Phetchaburi, Kanchanburi (AD)

Ślipiński, S. A. 1992: 451

##### Genus Parapeltis

Ślipiński, 1992

http://species-id.net/wiki/Parapeltis

Fig. 9 ; Map 8

Parapeltis Ślipiński, S. A. 1992: 451.

###### Type species.

*Parapeltis australicum* Ślipiński, 1992 [by original designation and monotypy]

Kolibáč, J. 2005: 75. Kolibáč, J. 2006: 111 (phylogeny)

###### Description.

Body size: 1.2 mm. Body shape convex (not conglobate). Gular sutures wide, convergent at apex. Frontoclypeal suture absent. Frons: longitudinal groove or depression absent. Cranium ventrally: tufts of long setae at sides absent. Submentum: ctenidium absent. Antennal groove present. Eyes: size moderate. Eyes number: two. Epicranial acumination moderate. Lacinial hooks absent. Galea: shape elongate. Galea: ciliate setae absent. Mediostipes-Lacinia fused together. Palpifer: outer edge even. Mandibular apical teeth number: two, horizontally situated. Mola present. Pubescence above mola or cutting edge present. Ventral furrow absent. Basal notch moderate. Labrum-Cranium not fused. Epipharyngial sclerite absent. Lateral tormal process: projection projection curved upwards (*Colydiopeltis*). Ligula: ciliate setae absent. Ligula membranous, not retroflexed, weakly emarginate. Hypopharyngeal sclerite absent. Antenna 8-segmented, sensorial fields absent. Front coxal cavities externally closed, internally open. Pronotum transverse. Prepectus present. Middle coxal cavities open. Elytra: long hairs absent. Epipleuron moderate. Elytral interlocking mechanism absent, carinae conspicuous. Elytral punctation regular, scales present. Front tibiae: spines along side reduced. Hooked spur absent, apical spurs not hooked or weakly hooked. Claws: denticle absent. Spiculum gastrale present. Tegmen composed of three parts.

###### Biology.

Unknown. Collected from leaf litter in *Eucalyptus* woodland.

###### Distribution.

Australia: Queensland.

###### Species:

*Parapeltis australicum* Ślipiński, 1992; Australia: Queensland

Ślipiński, S. A. 1992: 454. Kolibáč, J. 2005: 75

##### Tribe Phloiophilini

Kiesenwetter, 1863

Phloeophilidae Kiesenwetter, E. A. H. von 1863: 666 ( ).

###### Type genus:

*Phloiophilus* Stephens, 1830

Bouchard, P. et al. 2011: 56 (as Phloiophilidae). Klausnitzer, B. 1996: 145. Kolibáč, J. 1987: 110. Kolibáč, J. 2004a: 242. Kolibáč, J. 2008: 123 (phylogeny; stat. n. *sub* Trogossitidae). Lohse, G. A. 1979: 83 (as Phloeophilinae; *sub* Melyridae). Lawrence, J. F. 1982: 519 (morphology, systematics). Lawrence et al. 1993: CD-ROM (morphology of larvae). Lawrence et al. 1999a: CD-ROM (morphology of larvae). Lawrence et al. 1999b: CD-ROM (morphology of larvae). Majer 1994. Lawrence, J. F. & Newton, A. F., Jr. 1995: 867 (phylogeny). Mayor, A. 2007: 363 (distribution). Pic, M. 1926: 2

###### Remarks.

*Phloiophilus* has been classified within Dasytidae or Melyridae *sensu lato* (Reitter 1911, Lohse 1979) or as a part of an independent family, i.e. Phloiophilidae (= Phloeophilidae), formerly in conjunction with the genera *Xerasia* Lewis (now Byturidae) and *Acanthocnemus* Perris (Pic 1926). The latter genus is now classified in the monotypic family Acanthocnemidae Crowson, 1970 within the melyrid branch of Cleroidea or as a sister group of the rest of Cleroidea, whereas Phloiophilidae is generally considered a relative of Trogossitidae (Crowson 1964a, Gunter et al. 2013, Kolibáč 2004, Klausnitzer 1996, Lawrence et al. 1993, 1999a, b, Majer 1994). Recently, Hunt et al. (2007) published a comprehensive study based exclusively on molecular data, according to which *Phloiophilus edwardsi* is related to Biphyllidae and Byturidae. Both the families are situated in a basal position of Cleroidea, near to Trogossitidae. On the other hand, preliminary outcomes of work by the Tree of Life team (McKenna et al. 2012) as well as by Gunter et al. (2013), which are also based on molecular data, show a close relationship between *Phloiophilus* and Trogossitidae as well. Also believing in this relationship from a morphological point of view, I attempted to put its morphological characters into the trogossitid character matrix of 2006 (Kolibáč 2008). A computer analysis within the NONA program indicated a relationship between *Phloiophilus* and basal Peltinae but, to be honest, a detailed analysis of character states shows that the supposed relationship is at least partly based on shared plesiomorphies as well as “reductions” common in all Cleroidea. Thus, the position and status of the single genus of Phloiophilidae/Phoiophilini remains uncertain. It is included herein at the rank of tribe but, in the light of future discoveries, it may also be shifted from Cleroidea to a group within traditional “Cucujoidea”. The genus *Phloiophilus* was unfortunately not included in the most modern morphological analysis by Lawrence et al. (2011).

##### Genus Phloiophilus

Stephens, 1830

http://species-id.net/wiki/Phloiophilus

**Figure 10.**
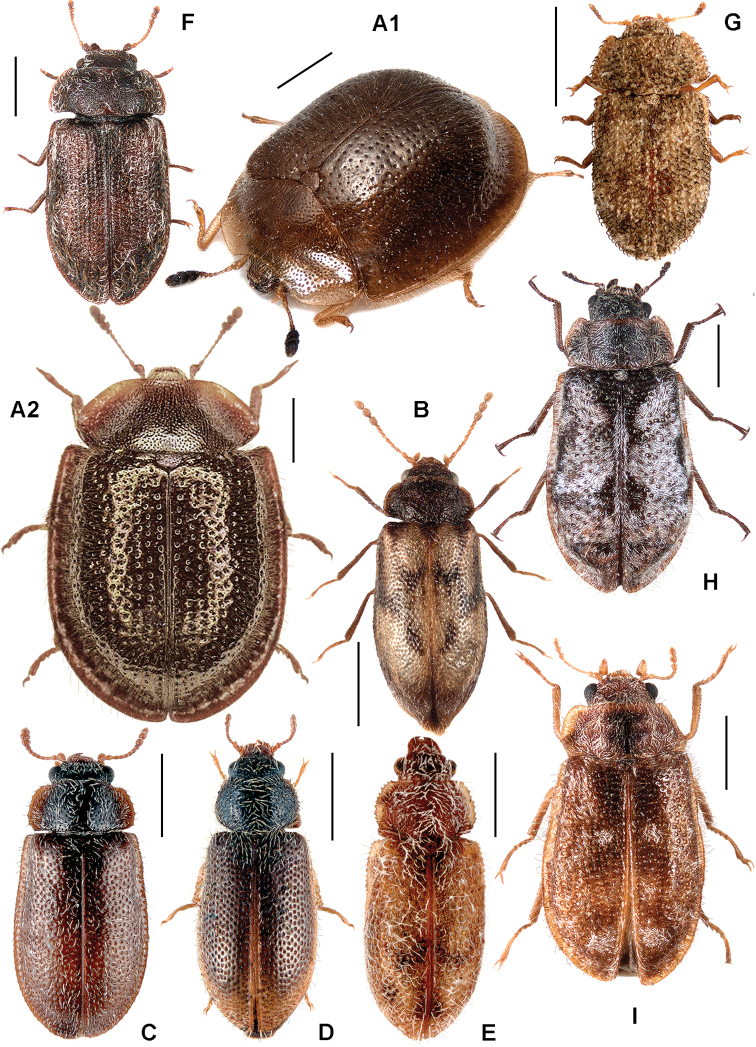
**A** *Thymalus limbatus* **B** *Phloiophilus edwardsi* **C** *Eronyxa marginicollis* **D** *Decamerus haemorhoidalis* **E** *Diontolobus punctatipennis* **F** *Afrocyrona ciskeiensis* **G** *Afrocyrona dwesae* **H** *Grynoma* sp., New Zealand **I** *Grynoma diluta*.

**Map 9.**
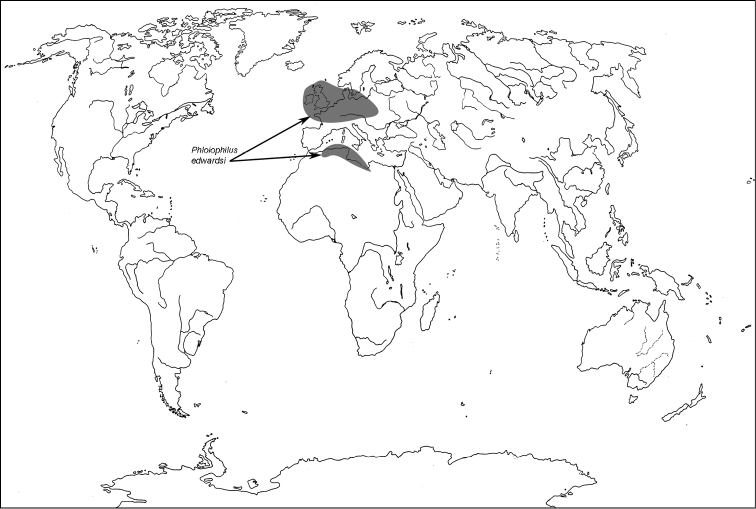
A distribution of the tribe Phloiophilini.

Figs 10 , 15 ; Map 9

Phloiophilus Stephens, J. F. 1830: 81.

###### Type species.

*Phloiophilus edwardsii* Stephens, 1830 [by monotypy]

Lohse, G. A. 1979: 83 (*Phloeophilus*, *sub* Melyridae). Majer, K. 1986: 114. Kolibáč, J. 2008: 105

###### Description.

Body size: about 3.0 mm. Body shape convex (not conglobate). Gular sutures wide, subparallel. Frontoclypeal suture absent. Frons: longitudinal groove or depression absent. Cranium ventrally: tufts of long setae at sides absent. Submentum: ctenidium absent. Antennal groove absent. Eyes: size moderate. Eyes number: two. Epicranial acumination absent. Lacinial hooks: two. Galea: shape sub-clavate. Galea: ciliate setae absent. Mediostipes-Lacinia fused together. Palpifer: outer edge even. Mandibular apical teeth number: two, horizontally situated. Mola absent. Penicillus (at base) present (fine, often membranous). Pubescence above mola or cutting edge present. Ventral furrow absent. Basal notch shallow or absent. Labrum-Cranium not fused. Epipharyngial sclerite absent. Lateral tormal process: projection projection not developed (all remaining). Ligula: ciliate setae absent. Ligula membranous, not retroflexed, deeply emarginate. Hypopharyngeal sclerite absent. Antenna 11-segmented. Antennal club symmetrical, sensorial fields absent. Front coxal cavities externally open, internally open. Pronotum transverse. Prepectus present. Middle coxal cavities open. Elytra: long hairs absent. Epipleuron thin. Elytral interlocking mechanism absent, carinae reduced. Elytral punctation irregular, scales absent. Wing: radial cell oblong (or reduced), wedge cell present, cross vein MP3-4 present, cross vein AA1+2-3+4 absent. Front tibiae: spines along side reduced. Hooked spur absent, apical spurs not hooked or weakly hooked. Claws: denticle absent. Tegmen composed of only one part. Coxitae divided.

Larva: Frontal arms curved (cucujoid). Epicranial stem reduced. Endocarina present. Gular sutures inconspicuous. Gula: anterior apodemes present. Paragular sclerites absent. Hypostomal rods absent. Stemmata number: five. Mandibular apical teeth number: two, horizontally even, vertically situated. Lacinia mandibulae tridentate. Mola absent. Maxillary palpi 3-segmented. Pedunculate seta absent. Mala simple. Mala: bidentate protrusion present. Ligula present. Labial palpi 2-segmented. Antennal joints 1, 2 transverse. Sensory appendix larger than half of joint 3. Thoracic sclerites pattern (dorsally) 1-0-0. Thoracic sclerites pattern (ventrally) 1+1+1. Abdominal segment IX not divided. Tergite IX flat. Urogomphi present, hooked; median process absent.

###### Biology.

Fungivorous. Crowson (1964b) noted adults and larvae from Great Britain: Adult and larva fungivorous, larvae feed beneath the thin and fleshy fruiting bodies of the basidiomycete *Phlebia radiata* Fr. of the Meruliaceae, which occurs on the dead wood of various deciduous trees (oak, beech, hazel), occasionally also conifers (pine). Adults are active in the warm days of autumn and winter (approximately, from late September to March). They have not been observed outside that period. They can be collected by sweeping from dry or decaying branches. Larvae may be found at all seasons, under the fruiting bodies of the fungus or under bark in spring and summer. Wielink et al. (2010) observed the species in the Netherlands and found adults active only by night. They live together with larvae on dead oak branches infested by the fungus *Peniophora quercina*.

###### Distribution.

Europe, North Africa.

###### Species:

*Phloiophilus edwardsii* Stephens, 1830; Austria, Belgium, Czechia, Denmark, France, Great Britain, Germany, Hungary, Ireland, the Netherlands, Poland, North Africa (JK)

Audisio, P. et al. 1995: 14. Beutel, R. G. & Pollock, D. A. 2000: 826 (larva, morphology). Crowson, R. A. 1964b: 151 (biology). Gurlich, S. et al. 1995: 49. Klausnitzer, B. 1996: 161. Kolibáč, J. 1999: 12. Kolibáč, J. 2008: 120. Lohse, G. A. 1979: 83 (*Phloeophilus*, *sub* Melyridae). Majer, K. 1986: 114. Mayor, A. 2007: 363 (syn. *Phloiophilus bimaculatus* Stephens, 1830; synonymized by whom?). Mayor, A. 2007: 364 (syn. *Phloiophilus cooperi* Stephens, 1830; synonymized by whom?). H. Vogt 1967: 13. Wielink van, P. et al. 2010: 17 (biology)

##### Tribe Thymalini

Léveillé, 1888

Thymalini Léveillé, A. 1888: 444.

###### Type genus.

*Thymalus* Latreille, 1802

Kireichuk, A. G. & Ponomarenko, A. G. 1990: 79 (Meligethiellinae, new Mesozoic subfamily). Kolibáč, J. 2006: 126 (diagnosis, stat. n.). Kolibáč, J. 2007a: 366. Krivosheina, N. P. & Mamaev, B. M. 1981: 50. Lawrence, J. F. & Newton, A. F. Jr. 1995: 868 (Protopeltinae; Rentoniinae). Ślipiński, S. A. 1992: 442 (Protopeltinae, Rentoniinae)

Meligethiellinae Kireichuk & Ponomarenko, 1990

Kolibáč, J. 2006: 126 (synonymized). revalid. subfam. Note: Removed from synonymy; see the “Remarks” section for explanation of this synonymy

Protopeltini Crowson, 1966; Protopeltinae Crowson, 1970

Kolibáč, J. 2006: 126 (synonymized)

Rentoniini Crowson, 1966; Rentoniinae Crowson, 1966 (Rentoniinae = Protopeltini + Rentoniini; all Crowson 1966: 120); Rentoniinae Crowson, 1970 (= new status for Rentoniini, i.e. without *Protopeltis*)

Kolibáč, J. 2006: 126 (synonymized)

###### Remarks.

A recent and extensive morphological study by Lawrence et al. (2011) considered Trogossitidae and even Cleroidea polyphyletic. In their phylogenetic tree, *Thymalus* is included in a cluster of the nitidulid genera along with *Cyclaxyra* and *Lamingtonium*, whereas the position of *Rentonellum* lies in a different part of the traditional Cucujoidea, near *Smicrips*, *Propalticus* and *Laemophloeus*. Leschen et al. (2012) used a restricted data set adopted from the Lawrence et al. (2011) study and found Cleroidea monophyletic but Trogossitidae polyphyletic: *Rentonellum* was a part of the melyrid group and *Thymalus* formed a sister group to all the remaining cleroids. The latter authors also suggested that the subfamily Rentoniinae be re-established. It is beyond the scope of the work in hand to assess this matter; it was prepared “in-group”, without using the extensive cucujiform outgroups. The morphological evidence used in the both above papers indicates the need to check Thymalini classification again, in the context of the wider cucujiform dataset, using molecular data. A new species of “*Rentonium*-group” has recently been discovered in South Africa (Leschen et al. 2012). Further new species is just described by Gimmel and Leschen (in press) together with its associated larva. It appears that the features of this larval record confirm Crowson’s earlier identification of the rentoniine larva from New Zealand (Crowson 1966).

Since the extinct Mesozoic genera *Meligethiella* and *Ostomalynus* are excluded from Thymalini, Peltinae and Trogossitidae herein, the synonymization of the subfamily Meligethiellinae is not valid henceforth and the taxon should not be further listed in synonymy of the tribe Thymalini.

###### Key to genera

(*Globorentonium* Lawrence & Ślipiński, 2013 not included)

**Table:** 

1	Body convex but not conglobate; elytra with conspicuous punctation	2
–	Body conglobate; elytra without sculpture (smooth) or with very fine irregular punctures or shagreened	3
2	Body extremely bulged; head including eyes covered by pronotum when viewed from above (only clypeus and labrum visible); larger species (about 4–7 mm)	*Thymalus*
–	Body convex but not extremely bulged; head protruding (not covered by pronotum when viewed from above); smaller species (about 2.5 mm)	*Protopeltis*
3	Elytra with light and dark spots; pubescence formed by short decumbent and long erect hairs	*Australiodes*
–	Elytra unicolorous, without spots; pubescence absent or made up of short hairs only	4
4	Elytra smooth, without pubescence, strongly involute; middle tibiae with row of spines at apex; wingless	*Rentonellum*
–	Elytra pubescent, not involute; middle tibiae probably without row of spines at apex; mostly winged	5
5	Middle coxal cavities widely separated; ventrites II–V with deeply transversely impressed anterior margins; last antennal segment transverse and truncate at apex; pubescence of elytra with shot-silk effect	*Parentonium*
–	Middle coxal cavities narrowly separated; ventrites II–V with or without weakly impressed anterior margins; last antennal segment not truncate at apex; pubescence of elytra sparse, directed backwards, without shot-silk effect	6
6	Head strongly transverse; front margin of clypeus with marked emargination; first ventrite with median keel at least in front; ventrites II–IV each with transverse line of strong backward-projecting setae near its hind border	*Rentonidium*
–	Head not transverse or only slightly so; front margin of clypeus almost straight; first ventrite without median keel; ventrites II–IV without such apical lines of setae	*Rentonium*

##### Genus Australiodes

Endrödy-Younga, 1960

http://species-id.net/wiki/Australiodes

Map 10

Liodidae Endrödy-Younga, S. 1960: 239 [*sub* ]

###### Type species:

*Clambus vestitus* Broun, 1886 [by monotypy]

Crowson, R. A. 1966: 120 (transferred from Liodidae to Trogossitidae: Peltinae). Kolibáč, J. 2005: 47. Kolibáč, J. 2006: 116 (phylogeny)

###### Description.

Body size: 1.4 mm. Body shape conglobate. Frons: longitudinal groove or depression absent. Cranium ventrally: tufts of long setae at sides absent. Eyes number: two. Lacinial hooks absent. Galea: shape partially fused with lacinia. Galea: ciliate setae absent. Mediostipes-Lacinia fused together. Labrum-Cranium not fused. Front coxal cavities externally open, internally closed. Pronotum subquadrate. Elytra: long hairs absent. Tegmen composed of a single unit (Kolibáč 2005).

“*Maxilla with galea and lacinia largely fused; erect setae among pubescence of upper surface; metendosternite with elongate oblique arms, without lamina; elytra with pattern of light and dark patches*” (ex Crowson 1966).

###### Biology.

Adults were collected by Crowson (1966: 123) “*in* Leucopogon *flowers and on male catkins of the introduced* Pinus insignis*. Adults were also found under loose bark of a dead* Hoheria.” [...] “*Adults doubtless feed on pollen, as shown by the gut-contents of a dissected specimen; the breeding sites are probably somewhere about dead trees...*” Crowson (1966).

###### Distribution.

New Zealand: Port Nicholson, Wellington; Waipoua State Forest, Northland; Western Hills, Whangarei.

**Map 10.**
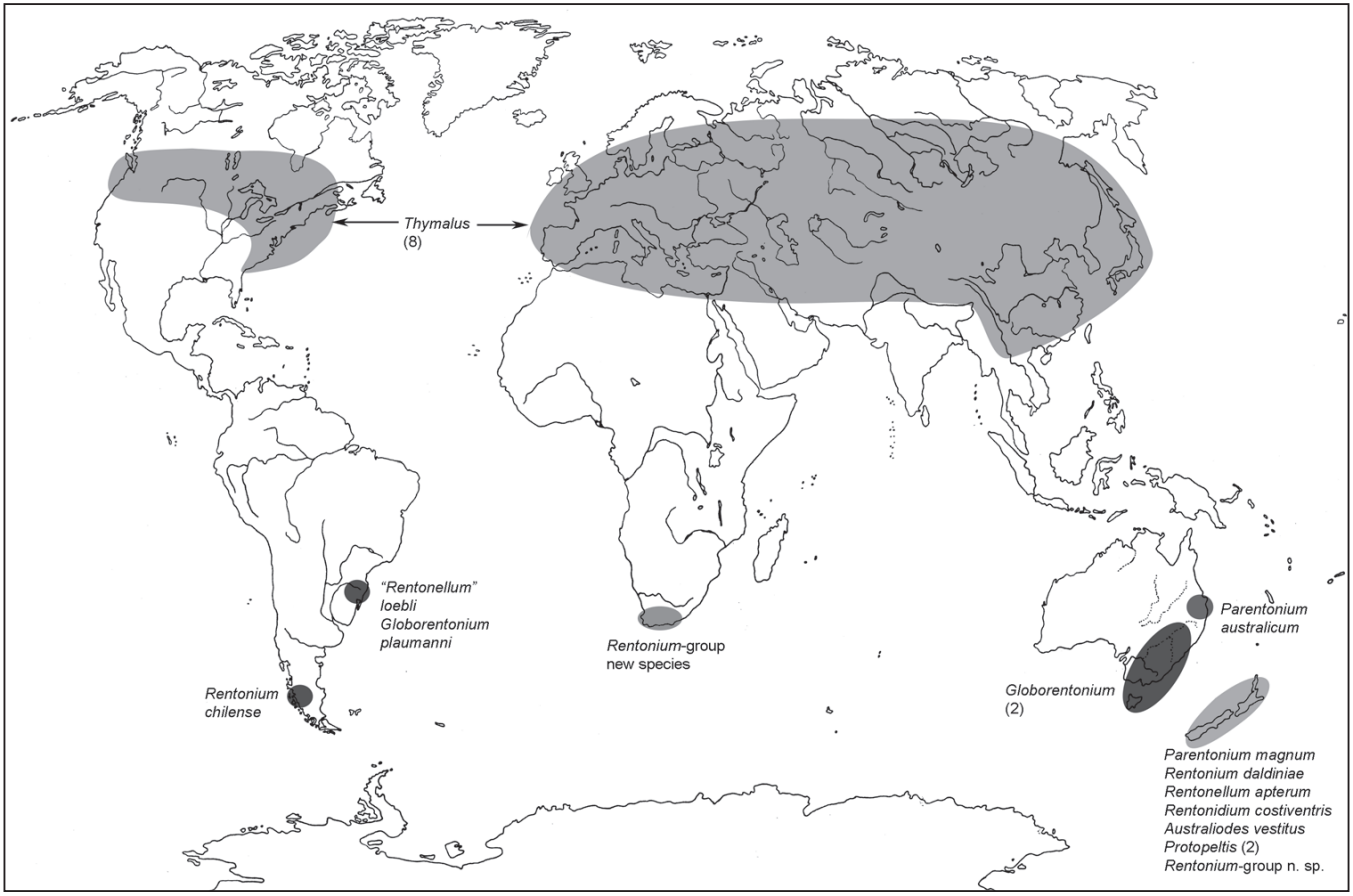
A distribution of the tribe Thymalini including *Rentonium*-group.

###### Species:

*Australiodes vestitus* Broun, 1886; New Zealand (varA)

Crowson, R. A. 1966: 120. Endrödy-Younga, S. 1960: 239 (Liodidae). Kolibáč, J. 2005: 47

##### Genus Globorentonium

Lawrence & Ślipiński, 2013

http://species-id.net/wiki/Globorentonium

Figs 9 , 17 ; Map 10

Globorentonium Lawrence, J. F. & Ślipiński, S. A. 2013: 258.

###### Type species.

*Globorentonium globulum* Lawrence & Ślipiński, 2013 [by original designation]

###### Remarks.

The genus has been just recently described, therefore, it is not included in a generic key to Thymalini. A key to the all genera of *Rentonium*-group (denoted as Rentoniini Crowson, 1966) is provided by authors (Lawrence and Ślipiński 2013: 270). *Globorentonium* includes three newly described species. A key to their recognition is also provided by the authors of original descriptions (Lawrence and Ślipiński 2013: 259). Four supposed specimens of *Globorentonium plaumanni* from the same locality as the type series (Brazil: Santa Catarina, Nova Teutonia, leg. F. Plaumann 1972–77) are figured here.

###### Distribution.

Australia: Victoria, New South Wales, Tasmania; Brazil: Santa Catarina.

###### Species:

*Globorentonium globulum* Lawrence & Ślipiński, 2013; Australia: Victoria, NSW, Tasmania (AD)

Lawrence, J. F. & Ślipiński, S. A. 2013: 260

*Globorentonium lescheni* Lawrence & Ślipiński, 2013; Australia: NSW (AD)

Lawrence, J. F. & Ślipiński, S. A. 2013: 264

*Globorentonium plaumanni* Lawrence & Ślipiński, 2013; Brazil: Santa Catarina (AD)

Lawrence, J. F. & Ślipiński, S. A. 2013: 268

##### Genus Parentonium

Crowson, 1970

http://species-id.net/wiki/Parentonium

Map 10

Parentonium Crowson, R. A. 1970: 6.

###### Type species.

*Rentonium magnum* Crowson, 1966 [by original designation]

Kolibáč, J. 2005: 76. Kolibáč, J. 2006: 116 (phylogeny)

###### Description.

Body size: 1.3–2.0 mm. Body shape conglobate. Gular sutures wide, subparallel. Frontoclypeal suture present. Frons: longitudinal groove or depression absent. Cranium ventrally: tufts of long setae at sides absent. Submentum of males: ctenidium absent. Antennal groove present. Eyes: size large, lateral. Eyes number: two. Epicranial acumination absent. Lacinial hooks absent. Galea: shape partially fused with lacinia. Galea: ciliate setae absent. Mediostipes-Lacinia fused together. Palpifer: outer edge denticulate. Front coxal cavities externally open, internally closed. Pronotum transverse. Elytra: long hairs absent. Epipleuron wide. Elytral interlocking mechanism absent, scales absent. Front tibiae: spines along side reduced. Claws: denticle absent.

###### Biology.

*Parentonium australicum* was found under the bark of a fallen *Nothofagus* trunk at an altitude of about 1200 m. *Parentonium magnum* was collected in mixed leaf-litter at about 250 m (Crowson 1966). It is presumed that the species are fungivorous.

###### Distribution.

Australia: Queensland, Lamington National Park; New Zealand: Grampian Hill, Nelson.

###### Species:

*Parentonium australicum* Crowson, 1970; Australia: Queensland (RAC)

Crowson, R. A. 1970: 6

*Parentonium magnum* Crowson, 1966; New Zealand (RAC)

Crowson, R. A. 1966: 121 (*Rentonium*). Crowson, R. A. 1970: 6 (*Parentonium*, combination). Kolibáč, J. 2005: 76

##### Genus Protopeltis

Crowson, 1964

http://species-id.net/wiki/Protopeltis

Fig. 9 ; Map 10

Protopeltis Crowson, R. A. 1964a: 287, 295.

###### Type species.

*Grynoma viridescens* Broun, 1886 [by original designation]

Crowson, R. A. 1964a: 295. Crowson, R. A. 1966: 120. Kolibáč, J. 2005: 79. Kolibáč, J. 2006: 111 (phylogeny)

###### Description.

Body size: about 2.5 mm. Body shape flat. Gular sutures wide, subparallel. Frontoclypeal suture absent. Frons: longitudinal groove or depression absent. Cranium ventrally: tufts of long setae at sides absent. Submentum: ctenidium absent. Antennal groove present. Eyes: size large, lateral. Eyes number: two. Lacinial hooks: three. Galea: shape elongate. Galea: ciliate setae absent. Palpifer: outer edge even. Mandibular apical teeth number: two, horizontally situated. Mola present. Penicillus (at base) absent. Pubescence above mola or cutting edge present. Ventral furrow present. Basal notch moderate. Labrum-Cranium not fused. Ligula: ciliate setae absent. Ligula membranous, not retroflexed. Antenna 11-segmented. Antennal club symmetrical, sensorial fields absent. Front coxal cavities externally open, internally open. Pronotum transverse. Middle coxal cavities open. Elytra: long hairs absent. Epipleuron moderate. Elytral interlocking mechanism absent, carinae reduced. Elytral punctation irregular, scales absent. Wing: radial cell oblong (or reduced), wedge cell absent, cross vein MP3-4 absent, cross vein AA1+2-3+4 absent. Front tibiae: spines along side moderate. Claws: denticle absent. Tegmen composed of two parts.

Larva: Frontal arms curved (cucujoid). Epicranial stem present. Endocarina present. Gular sutures conspicuous, parallel. Gula: anterior apodemes present. Paragular sclerites absent. Hypostomal rods absent. Stemmata number: five. Mandibular apical teeth number: two, horizontally situated. Lacinia mandibulae tridentate. Mola absent. Maxillary palpi 3-segmented. Palpifer present. Pedunculate seta absent. Mala simple. Mala: bidentate protrusion present. Cardo-Stipes not fused. Cardo: size much smaller than stipes. Labial palpi 2-segmented. Prementum in single part, anterior margin even. Antennal joints 1, 2 transverse. Sensory appendix larger than half of joint 3. Thoracic sclerites pattern (dorsally) 1-2-2. Thoracic sclerites pattern (ventrally) 1+0+0. Trochanter triangular. Abdominal segment IX not divided. Tergite IX flat. Urogomphi present, hooked; median process absent.

###### Biology.

Larvae of *Protopeltis viridescens* were collected, according to Crowson’s note, at “*fungusy bark of dead* Nothofagus”. The adult gut he examined contained “*abundant fungal material*”, determined as “*probably* Hymenochaete *sp.*” (Crowson 1964a). Larva and adult are probably fungivorous.

###### Distribution.

New Zealand (for example, Arthur’s Pass National Park).

###### Species:

*Protopeltis pulchella* Broun, 1915; New Zealand (RAC)

Broun, T. 1915: 314 (*Promanus*). Crowson, R. A. 1964a: 289

*Protopeltis viridescens* Broun, 1886; New Zealand (RAC)

Léveillé, A. 1910: 29 (*Grynoma*). Crowson, R. A. 1964a: 290 (larva). Iablokoff-Khnzorian, S. M. 1975: 147. Kolibáč, J. 2005: 78. Kolibáč, J. 2006: 109

##### Genus Rentonellum

Crowson, 1966

http://species-id.net/wiki/Rentonellum

Figs 9 , 17 ; Map 10

Rentonellum Crowson, R. A. 1966: 120.

###### Type species.

*Rentonellum apterum* Crowson, 1966 [by original designation and monotypy]

Kolibáč, J. 2005: 80. Kolibáč, J. 2006: 111 (phylogeny)

###### Description.

Body size: about 1.0 mm. Body shape conglobate. Gular sutures wide, subparallel. Frontoclypeal suture present. Frons: longitudinal groove or depression absent. Cranium ventrally: tufts of long setae at sides absent. Submentum: ctenidium absent. Antennal groove present. Eyes: size large, lateral. Eyes number: two. Epicranial acumination absent. Lacinial hooks absent. Galea: shape partially fused with lacinia. Galea: ciliate setae absent. Mediostipes-Lacinia fused together. Palpifer: outer edge even. Mandibular apical teeth number: two, horizontally situated. Mola present. Penicillus (at base) absent. Pubescence above mola or cutting edge absent. Ventral furrow absent. Basal notch shallow or absent. Labrum-Cranium not fused. Epipharyngial sclerite absent. Lateral tormal process: projection projection not developed (all remaining). Ligula: ciliate setae absent. Ligula rigid, not retroflexed, weakly emarginate. Hypopharyngeal sclerite consisting of two separate parts. Antenna 10-segmented. Antennal club symmetrical, sensorial fields absent. Front coxal cavities externally open, internally closed. Pronotum transverse. Prepectus absent. Middle coxal cavities closed. Elytra: long hairs absent. Epipleuron wide. Elytral interlocking mechanism absent, carinae reduced. Elytral punctation irregular, scales absent. Front tibiae: spines along side reduced. Hooked spur absent, apical spurs not hooked or weakly hooked. Claws: denticle absent. Parasternites number along ventrites III–VII: one. Coxitae undivided.

###### Biology.

*Rentonellum apterum* was found in *Nothophagus* and *Podocarpus* leaf-litter at an altitude of 900 m (Crowson 1966). The circumstances of the second species record are not known.

###### Distribution.

New Zealand; Brazil: “Nova Teutonia”.

###### Species:

*Rentonellum apterum* Crowson, 1966; New Zealand (RAC)

Crowson, R. A. 1966: 120. Kolibáč, J. 2005: 80

*Rentonellum loebli* Kolibáč, 2005; Brazil: “Nova Teutonia” (JK)

Kolibáč, J. 2005: 80

Note: It has been recently proposed that *Rentonellum loebli* might be a ciide rather than a rentoniine species (Lawrence and Ślipiński 2013).

##### Genus Rentonidium

Crowson, 1966

http://species-id.net/wiki/Rentonidium

Figs 9 , 17 ; Map 10

Rentonidium Crowson, R. A. 1966: 123.

###### Type species.

*Rentonidium costiventris* Crowson, 1966 [by original designation and monotypy]

Kolibáč, J. 2005: 81. Kolibáč, J. 2006: 116 (phylogeny)

###### Description

(according to Crowson 1966, modified). Body size: 1.3 mm. Adult: Body conglobate. General form short ovate and very convex, Byrrhid-like; length not more than 2 mm. Head strongly transverse; front margin of clypeus with a marked emargination; galea and lacinia separate; lacinia without spines or hooks; no erect setae on upper body surface; metendosternite with slender lamina. Front coxal cavities internally closed, coxae very elongate, separated by a very narrow process; first ventrite with a median keel at least in front; ventrites II–IV each with a transverse line of strong backward-projecting setae near its hind border; tegmen undivided.

###### Biology.

Collected in “*male flower of* Pinus insignis” (Crowson 1966). Probably feeding on pollen grains as do the adults of *Australiodes*.

###### Distribution.

New Zealand: Northland, Waipoua State Forest.

###### Species:

*Rentonidium costiventris* Crowson, 1966; New Zealand (RAC)

Crowson, R. A. 1966: 123. Kolibáč, J. 2005: 81

##### Genus Rentonium

Crowson, 1966

http://species-id.net/wiki/Rentonium

Figs 9 , 17 ; Map 10

Rentonium Crowson, R. A. 1966: 121.

###### Type species.

*Rentonium daldiniae* Crowson, 1966 [by original designation and monotypy]

Crowson, R. A. 1970: 6. Kolibáč, J. 2005: 81 (redescription). Kolibáč, J. 2006: 111 (phylogeny)

###### Description.

Body size: 1.3–1.5 mm. Body shape conglobate. Gular sutures wide, subparallel. Frontoclypeal suture absent. Frons: longitudinal groove or depression absent. Cranium ventrally: tufts of long setae at sides absent. Submentum: ctenidium absent. Antennal groove present. Eyes: size large, lateral. Eyes number: two. Epicranial acumination absent. Mandibular apical teeth number: one. Mola reduced but present. Penicillus (at base) absent. Pubescence above mola or cutting edge absent. Ventral furrow absent. Basal notch shallow or absent. Labrum-Cranium not fused. Epipharyngial sclerite absent. Lateral tormal process: projection projection not developed (all remaining). Ligula: ciliate setae absent. Ligula rigid, not retroflexed, weakly emarginate. Hypopharyngeal sclerite H-shaped. Antenna 10-segmented. Antennal club symmetrical, sensorial fields absent. Front coxal cavities externally open, internally closed. Pronotum transverse. Epipleuron thin. Elytral interlocking mechanism absent, carinae reduced. Elytral punctation irregular, scales absent. Wing: radial cell oblong (or reduced), wedge cell absent, cross vein MP3-4 absent, cross vein AA1+2-3+4 absent. Front tibiae: spines along side moderate. Hooked spur absent, apical spurs not hooked or weakly hooked. Claws: denticle absent. Spiculum gastrale present. Tegmen composed of only a single unit.

Larva: Frontal arms V-shaped. Epicranial stem absent. Endocarina present. Hypostomal rods absent. Stemmata number: five. Mandibular apical teeth number: two, horizontally situated. Lacinia mandibulae with several small spines. Mola absent. Maxillary palpi 2-segmented. Labial palpi 1-segmented. Torma: two separate lateral sclerites. Antennal joints 1, 2 transverse. Sensory appendix larger than half of joint 3. Thoracic sclerites pattern (dorsally) 1-2-2. Abdominal segment IX not divided. Tergite IX flat. Urogomphi present, hooked; median process absent.

###### Biology.

The single known larva described (Crowson speculated that it might be identified as *Rentonium daldiniae*) was extracted from a forest litter sample (mainly *Nothofagus*) at an elevation of about 500m (Crowson 1966). An adult *Rentonium daldiniae* was found in *Daldinia* sp. fungus growing on a dead tree (Crowson 1966). *Rentonium chilense* was collected in *Nothofagus* forest at around sea level.

###### Distribution.

New Zealand: Canterbury, Waimate. South Chile: Isla Bertrand.

###### Species:

*Rentonium chilense* Crowson, 1970; Chile (RAC)

Crowson, R. A. 1970: 7. Kolibáč, J. 2005: 81 (redescription)

*Rentonium daldiniae* Crowson, 1966; New Zealand (RAC)

Crowson, R. A. 1966: 121, 123 (supposed larva). Crowson, R. A. 1970: 7. Kolibáč, J. 2005: 81 (redescription). Kolibáč, J. 2006: 109

##### Genus Thymalus

Latreille, 1802

http://species-id.net/wiki/Thymalus

Figs 10 , 16 , 18 ; Map 10

Thymalus Latreille, 1802: 133.

###### Type species.

*Peltis brunnea* Thunberg, 1794 (= *Cassida limbata* Fabricius, 1787) [by original designation and monotypy]

Léveillé, A. 1910: 32. Barron, J. R. 1971: 35. Crowson, R. A. 1964a: 296. Kolibáč, J. 2005: 85 (redescription). Kolibáč, J. 2006: 111 (phylogeny). Kolibáč, J. 2007a: 366. Nikitsky, N. B. et al. 1998: 29 (key). Lafer, G. Sh. 1992: 83. Reitter, E. 1876: 64

*Thymalops* Iablokoff-Khnzorian, 1962 [Type species: *Cassida limbata* Fabricius, 1787]

Barron, J. R. 1971: 35. Iablokoff-Khnzorian, S. M. 1962: 421

###### Remarks.

Comparing the larvae as well as adults of *Thymalus* and *Protopeltis*, I found some interesting similarities, which led me to consideration of their phylogenetic relationship. Later character analysis (Kolibáč 2006) showed a relationship of *Thymalus* and *Protopeltis* with the former Rentoniini Crowson, 1966. This in turn led to the establishment of the tribe Thymalini for the group. However, Crowson (1966, 1970) also associated the former monotypic tribe Protopeltini Crowson, 1966 with the rentoniins. Recently, such a classification was called into question by Lawrence et al. (2011) and Leschen et al. (2012), who found Trogossitidae polyphyletic in their character analyses; however, both analyses were based on the same character states. Their model genera *Thymalus* and *Rentonellum* are classified outside Cleroidea in Lawrence et al. (2011) trees whereas Leschen et al. (2012), using a restricted character set, removed them only from Trogossitidae and/or suggested subfamily rank for rentoniins again, without necessarily believing in a mutual relationship between the two genera. Some more detail appears in “Remarks” with the tribe Thymalini.

Léveillé (1877) described the Caucasian species *Thymalus aubei* as *Thymalus fulgidus* var. *aubei* Léveillé, 1877. However, *Thymalus fulgidus* Erichson, 1844 was originally described from North America and Barron (1971) synonymized this species with *Thymalus marginicollis* Chevrolat, 1842. That is perhaps why the latter author also synonymized the taxon *aubei* as a synonym of *marginicollis*, probably without examination of the holotype or even Caucasian specimens. Russian entomologists, for example Nikitsky et al. (1998), consider *Thymalus aubei* a valid species, with the synonym *Thymalus subtilis* Reitter, 1889.

###### Description.

Body size: 4.3–7.5 mm. Body shape convex (not conglobate). Gular sutures wide, subparallel. Frontoclypeal suture absent. Frons: longitudinal groove or depression absent. Cranium ventrally: tufts of long setae at sides absent. Submentum: ctenidium absent. Antennal groove absent. Eyes: size large, lateral. Eyes number: two. Epicranial acumination moderate. Lacinial hooks: two. Galea: shape elongate. Galea: ciliate setae absent. Mediostipes-Lacinia not fused. Palpifer: outer edge even. Mandibular apical teeth number: two, horizontally situated. Mola present. Penicillus (at base) absent. Pubescence above mola or cutting edge absent. Ventral furrow absent. Basal notch moderate. Labrum-Cranium not fused. Epipharyngial sclerite absent. Lateral tormal process: projection projection not developed (all remaining). Ligula: ciliate setae absent. Ligula rigid, not retroflexed, weakly emarginate. Hypopharyngeal sclerite H-shaped. Antenna 11-segmented. Antennal club symmetrical, sensorial fields absent. Front coxal cavities externally open, internally closed. Pronotum transverse. Prepectus absent. Middle coxal cavities open. Elytra: long hairs absent. Epipleuron wide. Elytral interlocking mechanism absent, carinae reduced. Elytral punctation regular, scales absent. Wing: radial cell oblong (or reduced), wedge cell present, cross vein MP3-4 present, cross vein AA1+2-3+4 absent. Front tibiae: spines along side reduced. Hooked spur absent, apical spurs not hooked or weakly hooked. Claws: denticle absent. Parasternites number along ventrites III–VII: one. Spiculum gastrale absent. Tegmen composed of three parts.

Larva: Frontal arms V-shaped. Epicranial stem reduced. Endocarina absent. Gular sutures inconspicuous. Gula: anterior apodemes present. Paragular sclerites absent. Hypostomal rods present. Stemmata number: five. Mandibular apical teeth number: two, horizontally situated. Lacinia mandibulae with several small spines. Mola absent. Maxillary palpi 3-segmented. Palpifer present. Pedunculate seta absent. Mala simple. Mala: bidentate protrusion present. Cardo-Stipes partially fused. Cardo: size much smaller than stipes. Ligula present. Labial palpi 2-segmented. Prementum in single part, anterior margin even. Torma: two separate lateral sclerites. Antennal joints 1, 2 transverse. Sensory appendix larger than half of joint 3. Thoracic sclerites pattern (dorsally) 2-0-0. Thoracic sclerites pattern (ventrally) 0+0+0. Trochanter triangular. Abdominal segment IX not divided. Tergite IX flat. Urogomphi present, hooked; median process absent.

###### Biology.

The beetles are not associated with any particular tree species and are found on both deciduous and coniferous trees. *Thymalus limbatus* is known from the trunks of birch, beech, linden, and spruce, mostly under bark. It is assumed that the larvae feed on fungi in rotten or decaying wood (Kolibáč et al. 2005). *Thymalus marginicollis* has been collected from the fungi *Polyporus betulinus*, *Polyporus versicolor*, *Daedalea confragosa*, on the trunks of birch and also on “*wild flowers in plant press*” (Barron 1971).

###### Distribution.

Holoarctic: Northern states of USA, Canada, Europe, North Africa, Siberia to China and Japan. Some specimens, probably a new species, have recently been collected in Chinese Sichuan and Yunnan and also in northern Thailand.

###### Species:

*Thymalus aubei* Léveillé, 1877; “Batum”, Caucasus (varA)

Léveillé, A. 1910: 32. Klausnitzer, B. 1996: 156. Barron, J. R. 1971: 36 (syn. *Thymalus aubei* Léveillé, 1877 with *Thymalus marginicollis* Chevrolat, 1842). Barron, J. R. 1971: 36 (syn. *Thymalus fulgidus* var. *aubei* Léveillé, 1877 with *Thymalus marginicollis* Chevrolat, 1842). Kolibáč, J. 2007a: 366 (syn. *Thymalus subtilis* Reitter, 1889). Lafer, G. Sh. 1992: 86 (*Thymalus subtilis* Reitter, 1889). Léveillé, A. 1910: 33 (*Thymalus subtilis* Reitter, 1889). Nikitsky, N. B. et al. 1998: 28 (syn. *Thymalus subtilis* Reitter, 1889; lectotype designated). Nikitsky, N. B. & Semenov, V. B. 2001: 49

*Thymalus chinensis* Fairmaire, 1900; China: Fujian (JK)

Léveillé, A. 1910: 32. Kolibáč, J. 2007a: 366

*Thymalus laticeps* Lewis, 1894; Japan (varA)

Léveillé, A. 1910: 32. Esaki, T. et al. 1951: 1063. Kolibáč, J. 2007a: 366. Lafer, G. Sh. 1992: 86. Nakane, T. et al. 1963: 181

*Thymalus limbatus* Fabricius, 1787; Europe, North Africa: Tunisia (JK)

Léveillé, A. 1910: 32. Alexander, K. N. A. 1996: 90 (biology). Bahillo de la Puebla, P. & López-Colón, J. I. 1999: 13. Bahillo de la Puebla, P. & López-Colón, J. I. 2004: 129. Bercedo, P. et al. 2006: 180 (distribution). Borowiec, L. 1983: 15. Burakowski, B. et al. 1986: 123. Cunev, J. 1999: 76. Franz, H. 1981: 51–52 (distribution). Klausnitzer, B. 1976: 8. Klausnitzer, B. 1978: 176. Klausnitzer, B. 1996: 155. Kolibáč, J. 1993a: 20. Kolibáč, J. 1993b: 90. Kolibáč, J. 2002: 55 (larva). Kolibáč, J. 2005: 85 (redescription). Kolibáč, J. 2006: 110 (larva). Kolibáč, J. 2007a: 366 (distribution). Kolibáč, J. 2007a: 366 (syn. *brunneus* Thunberg, 1794). Kolibáč, J. 2007a: 366 (syn. *rubiginosus* Gmelin, 1790). Krasutskii, B. V. 1996: 274. Mitter, H. 1998: 561. Pileckis, S. & Monsevičius, V. 1995: 273. Ratti, E. 1997: 178. Reitter, E. 1876: 64. Theunert, R. 2006: 113–114 (distribution). Vogt, H. 1967: 18

*Thymalus marginicollis* Chevrolat, 1842; Canada, USA (JRB)

Barron, J. R. 1971: 36 (syn. *Thymalus aubei* Léveillé, 1877). Barron, J. R. 1971: 36 (syn. *Thymalus fulgidus* Erichson, 1844). Barron, J. R. 1971: 36 (syn. *Thymalus fulgidus* var. *aubei* Léveillé, 1877). Böving, A. G. & Craighead, F. C. 1931: 273 (larva). Dajoz, R. 1997: 44 (biology). Reitter, E. 1876: 64 (*Thymalus fulgidus* Erichson, 1844: “Amer. bor.”)

*Thymalus oblongus* Reitter, 1889; Russia: North and Central Europea teritorries, Sweden, East Siberia (JK)

Léveillé, A. 1910: 33. Lafer, G. Sh. 1992: 86. Kolibáč, J. 2007a: 366. Krasutskii, B. V. 2006: 763 (biology). Nikitsky, N. B. et al. 1998: 28 (lectotype designated)

*Thymalus parviceps* Lewis, 1894; Japan (varA)

Léveillé, A. 1910: 33. Esaki, T. et al. 1951: 1063. Kolibáč, J. 2007a: 366. Lafer, G. Sh. 1992: 86. Nakane, T. et al. 1963: 181

*Thymalus punctidorsum* Latreille, 1894; Japan (varA)

Léveillé, A. 1910: 33. Kolibáč, J. 2007a: 366. Lafer, G. Sh. 1992: 86. Nakane, T. et al. 1963: 181

#### Subfamily Lophocaterinae

Crowson, 1964

Lophocaterinae Crowson, R. A. 1964a: 297 ( ).
Lophocateridae Barron, J. R. 1971: 11, 12 (syn. = Peltinae). Burakowski, B. et al. 1986: 119 (Lophocateridae). Crowson, R. A. 1970: Hunt, T. et al. 2007: 1915 (molecular phylogeny). Klausnitzer, B. 1996: 145. Kolibáč, J. 2006: 125 (diagnosis). Kolibáč, J. & Zaitsev, A. A. (2010): 55 (subfamily status suggested). Lawrence, J. F. & Newton, A. F., Jr. 1995: 868 (Kirby, W. 1837: 104 is considered the author of the family rank name). Ślipiński, S. A. 1992: 442 (Lophocaterinae Crowson, 1964).

##### Remarks.

The subfamily Lophocaterinae was established by Crowson (1964a) in Trogossitidae (minus Peltidae) and later re-classified at family rank (Crowson 1970). As Crowson remarked (1970: 9), “*the group is very distinct from Peltidae in larval structure, but not easily separable from that family by skeletal characters of the adults; the larvae show apparent affinities to Trogossitidae* [...], *from which Lophocateridae are easily separable by characters of the adults.*” This is exactly the reason why taxonomists sometimes consider lophocaterins (the tribe cluster Decamerini-Lophocaterini-Ancyronini in this context) relatives of Peltinae, and sometimes Trogossitinae. The results of my two character analyses are also uncertain. In Kolibáč (2006), lophocaterins are unambiguously determined as a sister group of Peltinae but the improved analysis (Kolibáč 2008) is not so definitive. The second analysis resulted in 48 most parsimonious trees of which 16 supported a sister relationship between the lophocaterins and Trogossitinae but 32 trees supported a relationship with Peltinae. Further, molecular analyses published by Hunt et al. (2007), Bocáková et al. (2011) and Gunter et al. (2013) show closer relationships between a number of representatives of Peltinae and the lophocaterins than the latter and Trogossitinae. We may therefore consider the sister relationship Lophocaterinae-Peltinae more probable than Lophocaterinae-Trogossitinae. However, it is better to use subfamily rank for all three monophyletic clades to avoid a peltine polyphyly. This has already been explained by Kolibáč and Zaitsev (2010) in detail: “*There are three monophyletic branches in modern morphological analyses of Trogossitidae: (1) the subfamily Trogossitinae, (2) the tribe cluster Decamerini-Lophocaterini-Ancyronini, and (3) the subfamily Peltinae* sensu stricto *(Peltini-?Phloiophilini-Colydiopeltini-Thymalini). A sister relation of the lophocaterine cluster and Peltinae* s.str. *as recognized by Kolibáč (2006) and Hunt et al. (2007) was called into question by Kolibáč (2008) and by results of the present communication. Therefore, to rule out the potential polyphyly of Peltinae* s.lat. *(*sensu *Kolibáč 2006), we recommend to refer* [sic] *the tribe cluster Decamerini-Lophocaterini-Ancyronini again* [sic] *as the separate subfamily Lophocaterinae as aforementioned* [sic] *by Kolibáč (2008: 125).*”

##### Key to tribes of Lophocaterinae

**Table:** 

1	Tarsal claws with denticle. Larva: median process between urogomphi absent	Decamerini
–	Tarsal claws without denticle. Larva: median process between urogomphi present	2
2	Penicillus mostly composed of tuft of long setae. Frontoclypeal suture absent or weak and straight	Ancyronini
–	Penicillus membranous or composed of short, fine setae. Frontoclypeal suture conspicuous, mostly broadly emarginate	Lophocaterini

#### † Genus Cretamerus

Peris, Kolibáč & Delclòs (in press)

http://species-id.net/wiki/Cretamerus

Map 11

Cretamerus Peris D., Kolibáč J. & Delclòs X. (in press): iii.

##### Type species.

† *Cretamerus vulloi* Peris, Kolibáč & Delclòs, (in press) [by monotypy and author’s designation]

##### Remarks.

The fossil is the oldest known record confirmed for the entire superfamily Cleroidea on the European continent. Due to the fine state of preservation, certain morphological character states of the fossil were inserted into a character matrix of Trogossitidae genera. The resulting tree reveals the basal position of *Cretamerus vulloi* within the lophocaterine clade. It may form an extinct branch of the recent Decamerini.

In view of its fine state of preservation, certain morphological character states of the fossil to be inserted into a character matrix of Trogossitidae genera. The resulting tree reveals the basal position of *Cretamerus vulloi* within the lophocaterine clade. It may form an extinct branch of the recent Decamerini.

##### Original diagnosis.

Body very small, less than 2 mm. Antenna 10-segmented, 3-segmented club nearly symmetrical. Prothorax with dentate lateral margin; procoxal cavities externally closed. Elytra without carinae, punctation irregular or less than obviously regular. Tibiae with two straight (i.e. not hooked) spurs. Abdomen with six visible ventrites.

##### Distribution.

France: Charente-Maritime (Fouras/Bois-Vert); Cretaceous: early Cenomanian.

**Map 11.**
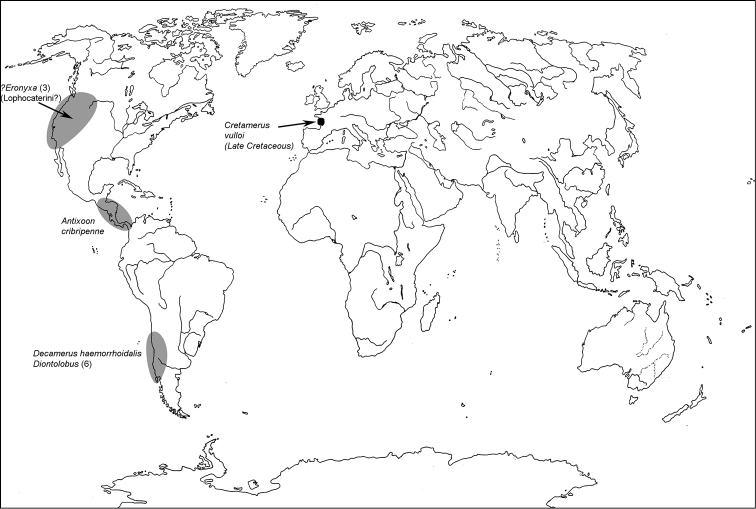
A distribution of the tribe Decamerini including the newly described genus † *Cretamerus*. A position of *Eronyxa* is indicated.

##### Species:

† *Cretamerus vulloi* Peris, Kolibáč & Delclòs (in press); France; Cretaceous (AD)

Peris D., Kolibáč J. & Delclòs X. (in press): iii

#### Tribe Decamerini

Crowson, 1964

Decamerini Crowson, R. A. 1964a: 287.

##### Type genus:

*Decamerus* Solier, 1849

Barron, J. R. 1975: 12 (syn. Decamerinae = Peltinae). Kolibáč, J. 2006: 127 (diagnosis, stat. n.). Lawrence, J. F. & Newton, A. F., Jr. 1995: 868 (Decamerinae). Ślipiński, S. A. 1992: 442, 460 (key) (Decamerinae)

##### Remarks.

Since it was established (Crowson 1964a), Decamerinae has always been connected with Peltidae (= Peltinae) in the works of a variety of authors. Unfortunately, the single larva described to date (Crowson 1964a) was not associated with adults. It possesses some general features of Lophocaterinae (mandible with long lacinia mandibulae, cranium with characteristic gular area) but it differs in having curved (cucujoid) frontal arms and in the absence of a median process between the urogomphi. The larva of *Eronyxa expansus* is evidently lophocaterine (Tait et al. 1990); however, the adults of *Eronyxa* and the present decamerins are similar in morphology and chiefly of floricolous habit. Two character analyses (Kolibáč 2006, 2008) placed *Eronyxa* on the border between Lophocaterini and Decamerini. Although the latter genus is classified within Lophocaterini herein, I feel its true relationship lies with Decamerini. This issue should be re-examined, although not before an indisputable larva of Chilean Decamerini is found and/or reared.

##### Key to genera

**Table:** 

1	Front coxal cavities completely externally closed; tarsal claws split into two almost identical parts	*Antixoon*
–	Front coxal cavities externally open or not completely closed; tarsal claws with large denticle (but shorter than outer claw)	2
2	Antenna 10-segmented; tegmen composed of one part; front coxal cavities open (or closed to 3/4)	*Decamerus*
–	Antenna 11-segmented; tegmen composed of three parts; front coxal cavities nearly closed	*Diontolobus*

#### Genus Antixoon

Gorham, 1886

http://species-id.net/wiki/Antixoon

Map 11

Antixoon Gorham, H. S. 1886: 332.

##### Type species.

*Antixoon cribripenne* Gorham, 1886 (*sub* Melyridae) [by monotypy] (not included in Léveillé 1910)

Crowson, R. A. 1964a: 291 (key). Kolibáč, J. 2005: 47. Kolibáč, J. 2006: 116 (phylogeny)

##### Remarks.

In consideration of a recent re-classification of *Eronyxa* within Lophocaterini (Tait et al. 1990), the systematic position of *Antixoon* needs to be checked. However, the bifid tarsal claws correspond with Crowson’s (1964a) opinion about a close relationship between *Antixoon*, *Diotolobus* and *Decamerus*.

##### Description.

Body size: about 3.0 mm. Body shape flat. Frons: longitudinal groove or depression absent. Cranium ventrally: tufts of long setae at sides absent. Antennal groove absent. Eyes number: two. Mola present. Penicillus (at base) absent. Pubescence above mola or cutting edge absent. Ventral furrow present. Basal notch moderate. Labrum-Cranium not fused. Antennal club symmetrical, sensorial fields absent. Front coxal cavities externally closed. Pronotum transverse. Elytra: long hairs absent. Claws: denticle distinct.

##### Biology.

Floricolous: “*collected in the flowers of bushes in more or less open country*” Crowson (1964a).

##### Distribution.

(Map 11.) Central America: Panama.

##### Species:

*Antixoon cribripenne* Gorham, 1886; Central America (Panama) (RAC)

Gorham, H. S. 1886: 332

#### Genus Decamerus

Solier, 1849

http://species-id.net/wiki/Decamerus

Figs 10 , 18 ; Map 11

Decamerus Solier, A. J. J. 1849: 369.

##### Type species.

*Decamerus haemorrhoidalis* Solier, 1849 [by monotypy]

Léveillé, A. 1910: 28. Crowson, R. A. 1964a: 291. Kolibáč, J. 2005: 52 (redescription). Kolibáč, J. 2006: 111 (phylogeny)

*Peltostoma* Reitter, 1877 [Type species: *Peltostoma unguicularis* Reitter, 1877; by monotypy]

Léveillé, A. 1910: 28 (synonymized?). Reitter, E. 1877: 173

##### Description.

Body size: about 3.0 mm. Body shape flat. Gular sutures wide, convergent at apex. Frontoclypeal suture present. Frons: longitudinal groove or depression absent. Cranium ventrally: tufts of long setae at sides absent. Submentum: ctenidium absent. Antennal groove absent. Eyes: size large, lateral. Eyes number: two. Epicranial acumination absent. Lacinial hooks absent. Galea: shape elongate. Galea: ciliate setae absent. Mediostipes-Lacinia partially fused. Palpifer: outer edge even. Mandibular apical teeth number: two, horizontally situated. Mola present. Penicillus (at base) present (fine, often membranous). Pubescence above mola or cutting edge absent. Ventral furrow present. Basal notch shallow or absent. Labrum-Cranium not fused. Epipharyngial sclerite absent. Lateral tormal process: projection projections extending laterally and downwards (*Eronyxa*). Ligula: ciliate setae absent. Ligula membranous, not retroflexed, deeply emarginate. Hypopharyngeal sclerite H-shaped. Antenna 10-segmented. Antennal club weakly asymmetrical, sensorial fields absent. Front coxal cavities externally open, internally open. Pronotum transverse. Prepectus present. Middle coxal cavities open. Elytra: long hairs absent. Epipleuron thin. Elytral interlocking mechanism absent, carinae reduced. Elytral punctation irregular, scales absent. Wing: radial cell moved down, often small, wedge cell absent, cross vein MP3-4 absent, cross vein AA1+2-3+4 absent. Front tibiae: spines along side moderate. Hooked spur absent, apical spurs not hooked or weakly hooked. Claws: denticle distinct. Parasternites number along ventrites III–VII: absent. Spiculum gastrale absent. Tegmen composed of only a single part.

##### Biology.

The species occur on the flowers of various bushes. Floricolous, found together with *Diontolobus*.

##### Distribution.

Central part of Chile, approximately regions IV to VI.

##### Species:

*Decamerus haemorrhoidalis* Solier, 1849; Chile (AL)

Léveillé, A. 1910: 28. Kolibáč, J. 2005: 52 (redescription). Reitter, E. 1877: 174 (syn. *Peltostoma unguicularis* Reitter, 1877; synonymized by Léveillé 1910?)

#### Genus Diontolobus

Solier, 1849

http://species-id.net/wiki/Diontolobus

Fig. 10 ; Map 11

Diontolobus Solier, A. J. J., 1849: 367.

##### Type species.

*Diontolobus punctipennis* Solier, 1849 [by monotypy]

Léveillé, A. 1910: 27. Crowson, R. A. 1964a: 291. Kolibáč, J. 2005: 53 (redescription). Kolibáč, J. 2006: 111 (phylogeny)

*Micropeltis* Redtenbacher, 1867 [Type species: *Micropeltis serraticollis* Redtenbacher, 1867; by monotypy]

Léveillé, A. 1910: 27 (synonymized?). Reitter, E. 1876: 58

##### Description.

Body size: about 3.0–4.0 mm. Body shape flat. Gular sutures wide, convergent at apex. Frontoclypeal suture present. Frons: longitudinal groove or depression absent. Cranium ventrally: tufts of long setae at sides absent. Submentum: ctenidium absent. Antennal groove absent. Eyes: size large, lateral. Eyes number: two. Epicranial acumination absent. Lacinial hooks absent. Galea: shape sub-clavate. Galea: ciliate setae absent. Mediostipes-Lacinia not fused. Palpifer: outer edge even. Mandibular apical teeth number: two, horizontally situated. Mola present. Penicillus (at base) present (fine, often membranous). Pubescence above mola or cutting edge present. Ventral furrow present. Basal notch moderate. Labrum-Cranium not fused. Epipharyngial sclerite absent. Lateral tormal process: projection projection curved upwards (*Colydiopeltis*). Ligula: ciliate setae absent. Ligula membranous, not retroflexed, deeply emarginate. Hypopharyngeal sclerite H-shaped. Antenna 11-segmented. Antennal club weakly asymmetrical, sensorial fields absent. Front coxal cavities externally closed, internally open. Pronotum transverse. Prepectus present. Middle coxal cavities open. Elytra: long hairs absent. Epipleuron thin. Elytral interlocking mechanism absent, carinae reduced. Elytral punctation irregular, scales absent. Wing: radial cell moved down, often small, wedge cell absent, cross vein MP3-4 absent, cross vein AA1+2-3+4 absent. Front tibiae: spines along side moderate. Hooked spur absent, apical spurs not hooked or weakly hooked. Claws: denticle distinct. Parasternites number along ventrites III–VII: absent. Spiculum gastrale absent. Tegmen composed of three parts.

Larva: Frontal arms curved (cucujoid). Epicranial stem absent. Endocarina absent. Gular sutures conspicuous, parallel. Paragular sclerites absent. Hypostomal rods absent. Stemmata number: five. Mandibular apical teeth number: two, horizontally situated. Lacinia mandibulae plumose. Mola present. Maxillary palpi 3-segmented. Pedunculate seta absent. Mala simple. Cardo-Stipes not fused. Ligula present. Labial palpi 2-segmented. Prementum in single part. Antenna 1st transverse, 2nd elongate. Sensory appendix medium sized (to half of joint 3). Thoracic sclerites pattern (dorsally) 0+0+0. Abdominal segment IX not divided. Tergite IX flat. Urogomphi present, hooked; median process absent.

##### Biology.

The species occur on flowers. Pollen grains were found in the gut of *Diontolobus punctipennis* (Crowson 1964a).

##### Distribution.

As same as in *Decamerus*, the central part of Chile, approximately regions IV to VI.

##### Species:

*Diontolobus costulata* Reitter, 1876; Chile (AL)

Léveillé, A. 1910: 27. Reitter, E. 1876: 60 (*Micropeltis costulata* Reitter, 1876)

*Diontolobus flavolimbata* Reitter, 1877; Chile (AL)

Léveillé, A. 1910: 27

*Diontolobus inaequalis* Reitter, 1877; Chile (AL)

Léveillé, A. 1910: 28

*Diontolobus incostata* Reitter, 1876; Chile (AL)

Léveillé, A. 1910: 28. Reitter, E. 1876: 59 (*Micropeltis incostata* Reitter, 1876)

*Diontolobus lanuginosa* Léveille, 1895; Chile (AL)

Léveillé, A. 1910: 28

*Diontolobus punctipennis* Solier, 1849; Chile (AL)

Léveillé, A. 1910: 28. Kolibáč, J. 2005: 53 (redescription). Reitter, E. 1876: 59 (syn. *Micropeltis serraticollis* Redtenbacher, 1867, synonymized by Léveillé 1910?)

*Diontolobus punctipennis* var. *lateritius* Fairmaire, 1883: 488

*Diontolobus* sp. (supposed larva); Chile (RAC)

Crowson, R. A. 1964a: 291. Kolibáč, J. 2006: 106

#### Tribe Ancyronini

Kolibáč, 2006

Ancyronini Kolibáč, J. 2006: 127.

##### Type genus.

*Ancyrona* Reitter, 1876 [designated by Kolibáč 2006]

Kolibáč, J. 2007a: 365. Kolibáč, J. & Zaitsev, A. A. 2010: 59 (phylogeny, larval morphology)

##### Remarks.

Ancyronini are undeniably related to Lophocaterini. As observations made upon the recently recorded *Ancyrona diversa* larva have shown (Kolibáč and Zaitsev 2010), the major morphological features of known larvae of the two tribes are nearly identical. In fact, it is likely that *Ancyrona* and allied genera are simply “advanced lophocaterins” adapted to a predatory way of life. Such a life-style is clearly demonstrated by their carnivorous mouthparts in both adults and larvae (sharp mandibles without mola), different life habits (they dwell on bark, branches or logs hunting for other insects) and well as their excellent capacity for rapid flight and swift reactions to intrusive stimuli. From the point of view of phylogenetic taxonomy, it appears that Lophocaterini are paraphyletic in relation to Ancyronini; both tribes are therefore in acute need of further study.

##### Key to genera

**Table:** 

1	Head quite hypognathous; tarsal pattern 4-4-4 or 5-5-5; body convex	2
–	Head quite prognathous; tarsal pattern 5-5-5; body more/less flattened	3
2	Antennae 10- or 11-segmented with loose 3-segmented club; mandible with penicillus formed by membranous appendage with short setae; eyes distinctly elevate	*Afrocyrona*
–	Antennae 10-segmented with compact 2-segmented club, segments 9 and 10 coalescent; mandible with penicillus composed of tuft of long setae; eyes not very elevate	*Neaspis*
3	Eyes distinctly elevate; labrum sometimes fused with cranium (males); lacinia without hooked spurs; mediostipes fused with lacinia	4
–	Eyes not very elevate; labrum free; lacinia with hooked pigmented spurs; mediostipes not fused with lacinia	*Ancyrona*
4	Mandible enlarged, male mandible monstrous; galea and lacinia without spines	*Leptonyxa*
–	Mandible not enlarged; lacinia with several pale spines	*Grynoma*

#### Genus Afrocyrona

Kolibáč, 2007

http://species-id.net/wiki/Afrocyrona

Fig. 10 ; Map 12

Afrocyrona Kolibáč, J. 2007b: 60.

##### Type species.

*Afrocyrona dwesae* Kolibáč, 2007 [designated by Kolibáč 2007b]

##### Description.

Body size: 2.9–5.0 mm. Body shape flat. Gular sutures narrow, subparallel at apex. Frontoclypeal suture present. Frons: longitudinal groove or depression absent. Cranium ventrally: tufts of long setae at sides present. Submentum: ctenidium absent. Antennal groove present. Eyes: size large, lateral. Eyes number: two. Epicranial acumination absent. Lacinial hooks: two. Galea: shape sub-clavate. Galea: ciliate setae absent. Mediostipes-Lacinia partially fused. Palpifer: outer edge even. Mandibular apical teeth number: two, horizontally situated. Mola absent. Penicillus (at base) long setae. Pubescence above mola or cutting edge absent. Ventral furrow absent. Basal notch shallow or absent. Labrum-Cranium not fused. Epipharyngial sclerite absent. Lateral tormal process: projection projection not developed (all remaining). Ligula: ciliate setae absent. Ligula membranous, not retroflexed, weakly emarginate. Hypopharyngeal sclerite H-shaped. Antenna 11-segmented or 10-segmented. Antennal club weakly asymmetrical, sensorial fields absent. Front coxal cavities externally open, internally open. Pronotum transverse. Prepectus present. Middle coxal cavities open. Elytra: long hairs absent. Epipleuron moderate. Elytral interlocking mechanism absent, carinae conspicuous. Elytral punctation regular, scales present. Wing: radial cell oblong (or reduced) or cell moved down, often small, wedge cell absent, cross vein MP3-4 present, cross vein AA1+2-3+4 absent. Front tibiae: spines along side moderate. Hooked spur present. Tarsal pattern 4-4-4 or 5-5-5. Claws: denticle absent. Parasternites number along ventrites III–VII: one. Spiculum gastrale absent. Tegmen composed of two parts. Coxitae divided.

##### Biology.

The species were collected by beating branches and sifting rotten wood and litter. Detritus and some insect remnants were found in the gut of *Afrocyrona dwesae*, whereas only insect fragments were found in the gut of *Afrocyrona ciskeiensis*. The species are probably predatory and partly or occasionally fungivorous. *Afrocyrona dwesae* may be unable to fly.

##### Distribution.

Three described species are known from South Africa: Eastern Cape and Transvaal. One or two more species have been recently found in the island of Sokotra.

**Map 12.**
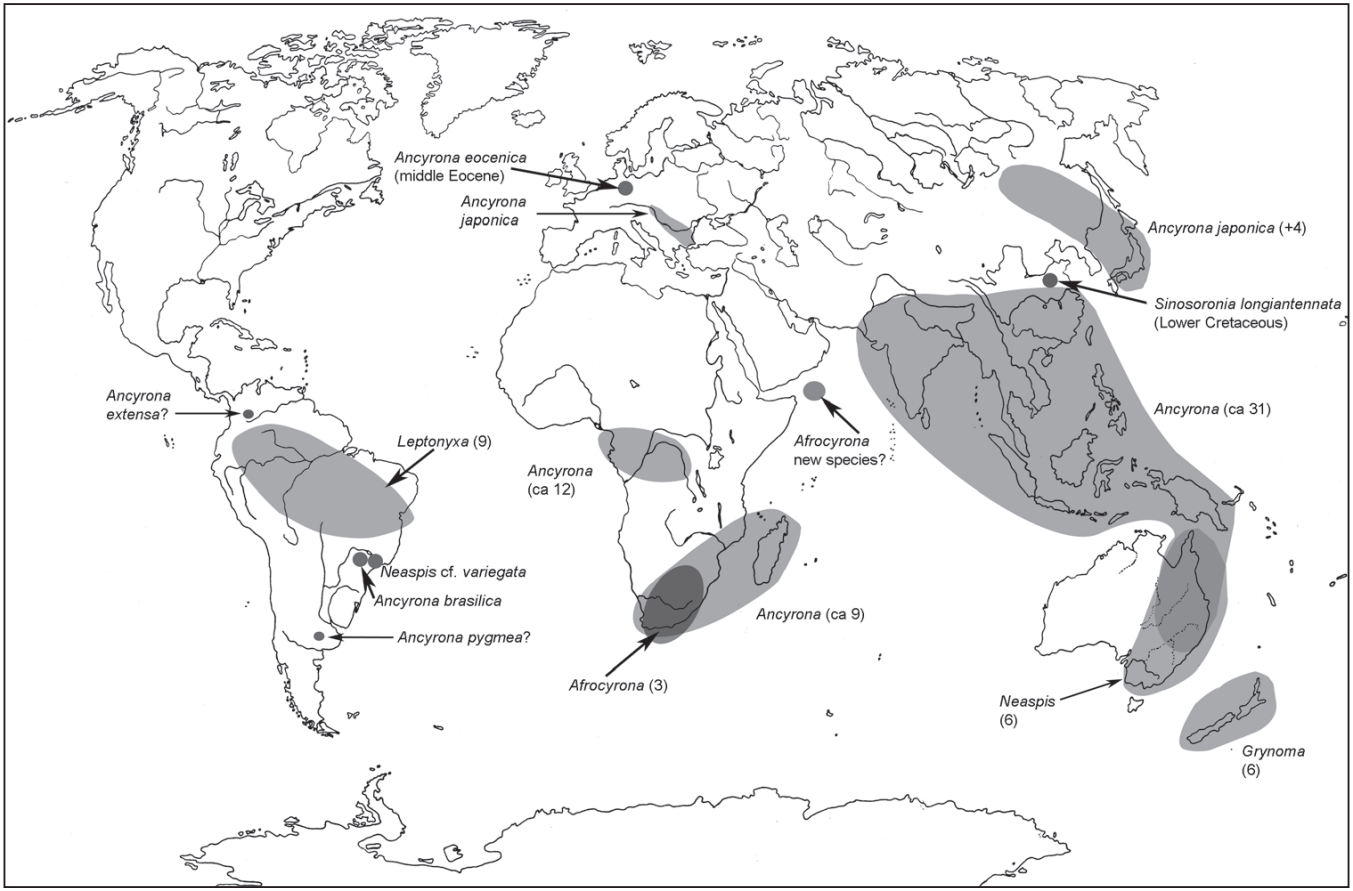
A distribution of the tribe Ancyronini.

##### Species:

*Afrocyrona ciskeiensis* Kolibáč, 2007; South Africa (JK)

Kolibáč, J. 2007b: 61

*Afrocyrona dwesae* Kolibáč, 2007; South Africa (JK)

Kolibáč, J. 2007b: 63

*Afrocyrona gussmannae* Kolibáč, 2007; South Africa (JK)

Kolibáč, J. 2007b: 64

#### Genus Ancyrona

Reitter, 1876

http://species-id.net/wiki/Ancyrona

**Figure 11.**
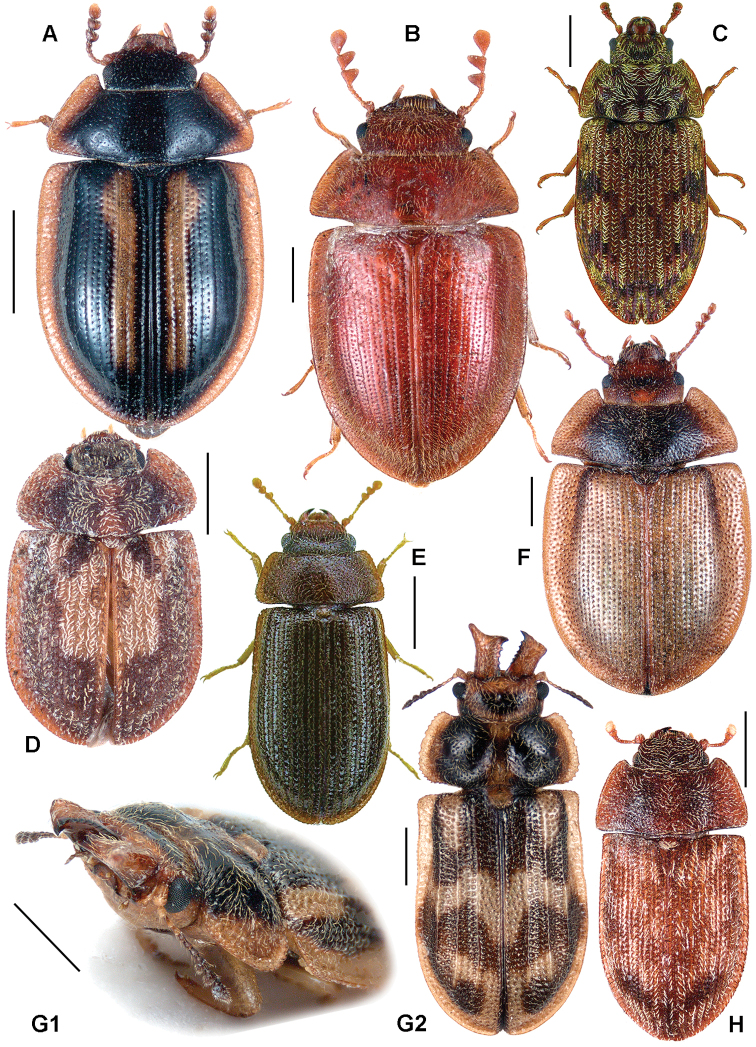
**A** *Ancyrona* (syn. *Latolaeva*) *bivittata* **B** *Ancyrona vicina* **C** *Ancyrona kosnovskorum* (= *fairmairei*?) **D** *Ancyrona* sp., *lewisi*-group, Malaysia **E** *Ancyrona japonica*, Slovakia **F** *Ancyrona gabonica* **G** *Leptonyxa fairmairei* **H** *Neaspis variegata*.

Figs 2 , 11 ; Map 12

Ancyrona Reitter, E. 1876: 51.

##### Type species.

*Ancyrona lewisi* Reitter, 1876 [designated by Kolibáč 1993]

Léveillé, A. 1910: 25. Lafer, G. Sh. 1992: 83. Matthews, E. G. 1992: 3 (key, Australia). Kolibáč, J. 2005: 45. Kolibáč, J. 2006: 111 (phylogeny). Kolibáč, J. 2007a: 365. Kolibáč, J. & Zaitsev, A. A. (2010): 55 (larva)

*Latolaeva* Reitter, 1876 (Type species: *Latolaeva ferrarii* Reitter, 1876; designated by Kolibáč 2005)

Léveillé, A. 1910: 25. Kolibáč, J. 2005: 63. Kolibáč, J. 2006: 111 (phylogeny). Kolibáč 2007b: 54 (synonymized). Reitter, E. 1876: 49

##### Description.

Body size: 3.1–8.0 mm. Body shape flat. Gular sutures narrow, subparallel at apex. Frontoclypeal suture present or broadly emarginate. Frons: longitudinal groove or depression absent. Cranium ventrally: tufts of long setae at sides present. Submentum: ctenidium absent. Antennal groove present. Eyes: size large, lateral. Eyes number: two. Epicranial acumination absent. Lacinial hooks: two. Galea: shape sub-clavate. Galea: ciliate setae absent. Mediostipes-Lacinia not fused. Palpifer: outer edge even. Mandibular apical teeth number: two, horizontally situated. Mola absent. Penicillus (at base) long setae. Pubescence above mola or cutting edge absent. Ventral furrow absent. Basal notch shallow or absent. Labrum-Cranium not fused. Epipharyngial sclerite absent. Lateral tormal process: projection projection not developed (all remaining). Ligula: ciliate setae absent. Ligula membranous, not retroflexed, weakly emarginate. Hypopharyngeal sclerite H-shaped. Antenna 10-segmented. Antennal club weakly asymmetrical, sensorial fields absent. Front coxal cavities externally open, internally open. Pronotum transverse. Prepectus present. Middle coxal cavities open. Elytra: long hairs absent. Epipleuron moderate. Elytral interlocking mechanism absent, carinae conspicuous. Elytral punctation regular, scales present. Wing: radial cell oblong (or reduced) or cell moved down, often small, wedge cell absent, cross vein MP3-4 present, cross vein AA1+2-3+4 absent. Front tibiae: spines along side moderate. Hooked spur present. Claws: denticle absent. Parasternites number along ventrites III–VII: one. Spiculum gastrale absent. Tegmen composed of two or three parts. Coxitae undivided.

Larva *Ancyrona diversa* Pic, 1921 (Kolibáč and Zaitsev 2010): Frontal arms Y-shaped. Epicranial stem minute, nearly absent. Endocarina present. Gular sutures conspicuous, convergent. Gula: anterior apodemes absent. Paragular sclerites absent. Hypostomal rods present. Stemmata number: two. Mandibular apical teeth number: two, horizontally situated. Lacinia mandibulae plumose. Mola absent. Maxillary palpi 3-segmented. Palpifer present. Pedunculate seta present. Mala simple. Mala: bidentate protrusion absent. Cardo-Stipes not fused. Cardo: size nearly as large as stipes. Ligula present. Labial palpi 2-segmented. Prementum in two parts, anterior margin even. Torma: two separate lateral sclerites and plate between them. Antennal joints 1, 2 elongate. Sensory appendix longer than half of joint 3. Thoracic sclerites pattern (dorsally) 1–2–0. Thoracic sclerites pattern (ventrally) 1–0–0. Trochanter oblong. Abdominal segment IX transversely divided. Tergite IX flat. Urogomphi present, hooked; median process present.

##### Biology.

Predatory. Adults can be beaten from dry branches or individually collected on fallen timber, where they hunt for other insects.

##### Distribution.

Africa in the south of Sahara, eastern Asia (from India to Japan and Russian Far East), Australia: Queensland, New South Wales (types of *Ancyrona aegra*, *Ancyrona amica*, *Ancyrona latebrosa*, *Ancyrona laticeps*, *Ancyrona vesca* checked), New Guinea (including undescribed species). Strangely enough, one Japanese species, *Ancyrona japonica*, was introduced to Europe and first recorded in Hungary (“Visegrad 1904”). It has now been collected several times in adjacent Slovakia, on branches of decaying oak. Two Tertiary fossil species are known from European Eocene.

##### Species groups

In a review of *Afrocyrona* and *Ancyrona*, I divided the latter genus in several informal species groups (Kolibáč 2007b). They are listed below together with a short diagnosis, although not all *Ancyrona* species are classified within the particular groups. This is only the first attempt to classify the rich and complex genus, so a monophyly of these groups should be checked and tried, then the groups may be established at the rank of subgenera).

###### *lewisi* species-group

**Species.** *Ancyrona lewisi*, *Ancyrona shibatai*, *Ancyrona haroldi*, and numerous undescribed species from south and eastern Asia and New Guinea.

**Distribution.** Japan, China, southeastern Asia.

**Diagnosis.** Small, flat, broad, oval species. Dorsal surface with colour pattern formed by scales or rigid decumbent setae.

###### *gabonica* species-group

**Species.** *Ancyrona gabonica*, *Ancyrona ferrarii*, *Ancyrona vicina*, *Ancyrona bivittata*, *Ancyrona feai*, *Ancyrona incensa*, *Ancyrona martini*, *Ancyrona plana*, and numerous undescribed species from eastern Asia and tropical Africa. Australian species *Ancyrona aegra*, *Ancyrona amica*, *Ancyrona latebrosa*, *Ancyrona laticeps*, *Ancyrona vesca* probably also belong to the group.

**Distribution.** Tropical Africa, south-eastern Asia and probably Australia.

**Diagnosis.** Extremely flat, broad, oval species without scales and lacking decumbent pubescence on dorsal surface; dorsal side quite bare or with inconspicuous or erect pubescence. Some species with longitudinal colour stripes on elytra; stripes not formed by pubescence or scales. Antennal club very loose, nearly serrate. Tegmen composed of two or three parts.

###### *japonica* species-group

**Species.** *Ancyrona japonica*, *Ancyrona diversa*.

**Distribution.** Japan, northern China, Ussuri. *Ancyrona japonica* also spread to south-eastern and central Europe (from Bulgaria through Hungary to Slovakia); first European record in 1904.

**Diagnosis.** Rather elongate unicolorous species with long, soft pubescence on dorsal surface. Tegmen composed of 1 or 2 parts (parameral piece fused with phallobase), male abdominal segments IX–X more or less reduced.

###### *colobicoides* species-group

**Species.** *Ancyrona colobicoides*, *Ancyrona fairmairei*, *Ancyrona kosnovskorum* (syn. *fairmairei*?), *Ancyrona minor*.

**Distribution.** Madagascar.

**Diagnosis.** Flat, elongate species with conspicuously elevate eyes and compact 3-segmented club. Yellowish elytra with dark black-brown pattern (stripes, spots), pubescence short and decumbent. Primitive tegmen composed of three parts. Elytra with distinct carinae. One specimen known to me from Madagascan copal (Cap d’Ambre).

###### *endroedyi* species-group

**Species.** *Ancyrona endroedyi*, *Ancyrona muellerae*, *Ancyrona caffra*(?).

**Distribution.** South Africa.

**Diagnosis.** Body quite elongate (as in *japonica* group), with long, soft, erect hairs or short, decumbent, scale-like setae. Dorsal surface unicolorous or with pattern formed by pubescence. Radial cell completely or partly reduced. Tegmen composed of two or three parts. Elytra with more or less conspicuous carinae.

###### Species:

*Ancyrona aegra* Olliff, 1885; “Nov. Gal. mer.” (AL)

Léveillé, A. 1910: 25

*Ancyrona amica* Olliff, 1885; S Australia (AL)

Léveillé, A. 1910: 25

*Ancyrona andrewesi* Léveillé, 1907; India (AL)

Léveillé, A. 1910: 25

*Ancyrona aurora* Léveillé, 1899; Congo gall. (AL)

Léveillé, A. 1910: 25

*Ancyrona bivittata* Léveillé, 1899; Cameroon (AL)

Léveillé, A. 1910: 25 (*Latolaeva bivittata* Léveillé, 1899). Kolibáč, J. 2007b: 55 (comb.; transferred from *Latolaeva* to *gabonica*-group)

*Ancyrona blaisei* Léveillé, 1907; Tonkin (AL)

Léveillé, A. 1910: 25

*Ancyrona bouchardi* Léveillé, 1902; Sumatra (AL)

Léveillé, A. 1910: 25

*Ancyrona brasilica* Perty, 1830; Brazil: Minas Geraes (AL)

Léveillé, A. 1910: 25 (*Latolaeva brasilica* Perty, 1830)

Note: Cleroidea? (see Reitter 1876: 51).

*Ancyrona brunnea* Léveillé, 1905; India (AL)

Léveillé, A. 1910: 25

*Ancyrona brunneolimbata* Léveillé, 1905; Fernando Po (AL)

Léveillé, A. 1910: 25

*Ancyrona caffra* Reitter, 1876; Cap (AL)

Reitter, E. 1876: 52. Léveillé, A. 1910: 25

*Ancyrona cassidoides* Reitter, 1876; Malacca (AL)

Léveillé, A. 1910: 25 (*Latolaeva cassidoides* Reitter, 1876). Reitter, E. 1876: 50 (*Latolaeva cassidoides* Reitter, 1876)

*Ancyrona ciliata* Murray, 1867; Old Calabar (Nigeria) (AL)

Léveillé, A. 1910: 25 (transferred from *Peltis* to *Ancyrona*). Reitter, E. 1876: 53 (*Peltis*)

*Ancyrona colobicoides* Fairmaire, 1868; Madagascar (AL, confirmed JK)

Léveillé, A. 1910: 30 (*Ostoma* (s.str.)). Kolibáč, J. 2007b: 57 (comb.; transferred from *Ostoma* to *colobicoides*-group)

Note: maybe conspecific with *Ancyrona kosnovskorum*.

*Ancyrona congolensis* Léveille, 1905; Congo gall. (AL)

Léveillé, A. 1910: 26

*Ancyrona crenata* Murray, 1867; Old Calabar (Nigeria) (AL)

Léveillé, A. 1910: 26. Reitter, E. 1876: 54 (*Peltis*)

*Ancyrona diversa* Pic, 1921; E Siberia (Ussuri) (JK)

Pic, M. 1921: 1 (*Ostoma*). Kolibáč, J. 1993a: 17 (comb.; transferred from *Ostoma* to *Ancyrona*). Kolibáč, J. 1999b: 12 (morphology). Kolibáč, J. 2007a: 365 (distribution). Kolibáč, J. 2007b: 56 (shifted to *japonica*-group). Kolibáč, J & Zaitsev, A. A. 2010: 53 (larva)

*Ancyrona elongata* Léveille, 1905; India (AL)

Léveillé, A. 1910: 26

*Ancyrona endroedyi* Kolibáč, 2007; SA: Transvaal (JK)

Kolibáč, J. 2007b: 58

† *Ancyrona eocenica* Schmied, Wappler & Kolibáč, 2009; Germany; Tertiary: middle Eocene

Schmied, H. et al. 2009: 18

*Ancyrona extensa* Reitter, 1877; Bolivia: Bogota (AL)

Léveillé, A. 1910: 26

*Ancyrona fairmairei* Léveillé, 1903; Madagascar (AL, confirmed JK)

Léveillé, A. 1910: 30 (*Ostoma* (s.str.)). Kolibáč, J. 2007b: 58 (comb.; transferred from *Ostoma* to *colobicoides*-group)

*Ancyrona feai* Léveillé, 1905; Congo gall., Cameroon (AL)

Léveillé, A. 1910: 26. Kolibáč, J. 2007b: 55 (shifted to *Ancyrona gabonica*-group)

*Ancyrona ferrarii* Reitter, 1876; Batchian Ins. (AL)

Léveillé, A. 1910: 25 (*Latolaeva*). Kolibáč, J. 2005: 63 (*Latolaeva*; redescription). Kolibáč, J. 2007b: 55 (comb.; transferred from *Latolaeva* to *gabonica*-group). Reitter, E. 1876: 50 (*Latolaeva*)

*Ancyrona francoisi* Léveillé, 1907; Tonkin (AL)

Léveillé, A. 1910: 26

*Ancyrona fryi* Léveillé, 1899; Assam, Perak, Sumatra (AL)

Léveillé, A. 1910: 26

*Ancyrona gabonica* Léveillé, 1899; Congo gall. (AL)

Léveillé, A. 1910: 26. Kolibáč, J. 2007b: 55 (shifted to *gabonica*-group)

*Ancyrona gestroi* Reitter, 1880; Australia, New Guinea (AL)

Léveillé, A. 1910: 26

*Ancyrona grouvellei* Léveille, 1899; Détr. Torres (AL)

Léveillé, A. 1910: 26

*Ancyrona haroldi* Reitter, 1877; Japan (AL, confirmed JK)

Léveillé, A. 1910: 26. Esaki, T. et al. 1951: 1062. Kolibáč, J. 2007a: 365. Kolibáč, J. 2007b: 54 (shifted to *lewisi*-group). Nakane, T. et al. 1963: 181

*Ancyrona higonia* Lewis, 1894; Japan (AL)

Nakane, T. et al. 1963: 182 (*Ostoma*)

Note: combined with *Ancyrona* here according to a photograph in Nakane, T. et al. (1963).

*Ancyrona horni* Léveillé, 1902; Sri Lanka (AL)

Léveillé, A. 1910: 26

*Ancyrona incensa* Oliff, 1883; Malacca (AL)

Léveillé, A. 1910: 25 (*Latolaeva*). Kolibáč, J. 2007b: 55 (comb.; transferred from *Latolaeva* to *gabonica*-group

*Ancyrona indica* Léveillé, 1907; India (AL)

Léveillé, A. 1910: 26

*Ancyrona japonica* Reitter, 1889; Asia: Japan; Europe: Bulgaria?, Czechia, Hungary (JK)

Reitter, E. 1889: 15 (*Ostoma*). Léveillé, A. 1910: 31 (*Ostoma*). Esaki, T. et al. 1951: 1062 (*Grynocharis*;comb.). Kolibáč, J. 1993a: 18 (comb.; transferred from *Grynocharis* to *Ancyrona*). Kolibáč, J. 1993b: 90 (check-list). Kolibáč, J. 1999: 12 (morphology). Kolibáč, J. 2007a: 365 (distribution). Kolibáč, J. 2007b: 56 (shifted to *japonica*-group). Lafer, G. Sh. 1992: 84 (*Grynocharis*). Nakane, T. et al. 1963: 182 (*Grynocharis*)

*Ancyrona javanica* Léveille, 1905; Java (AL)

Léveillé, A. 1910: 26

*Ancyrona kosnovskorum* Kolibáč, 2005; Madagascar (JK)

Kolibáč, J. 2005: 46. Kolibáč, J. 2007b: 58 (shifted to *colobicoides*-group)

Note: maybe synonym of *Ancyrona colobicoides*.

*Ancyrona lanuginosa* Motschulsky, 1863; Sri Lanka (AL)

Léveillé, A. 1910: 26. Reitter, E. 1876: 52

*Ancyrona latebrosa* Olliff, 1885; Queensland (AL)

Léveillé, A. 1910: 26

*Ancyrona laticeps* Olliff, 1885; Queensland, Nov. Gall. mer. (=NSW?) (AL)

Léveillé, A. 1910: 26

*Ancyrona lewisi* Reitter, 1876; Japan (AL)

Léveillé, A. 1910: 26. Kolibáč, J. 1993a: 16. Kolibáč, J. 2005: 45. Kolibáč, J. 2007b: 54 (shifted to *lewisi*-group). Lafer, G. Sh. 1992: 84. Reitter, E. 1876: 52

*Ancyrona maculipennis* Kraatz, 1878; S Africa (AL)

Léveillé, A. 1910: 26

*Ancyrona martini* Léveille, 1899; Natal (AL)

Léveillé, A. 1910: 26. Kolibáč, J. 2007b: 55 (shifted to *gabonica*-group)

*Ancyrona minor* Fairmaire, 1900; Madagascar (AL)

Léveillé, A. 1910: 31 (*Ostoma* (s.str.)). Kolibáč, J. 2007b: 58 (comb.; transferred from *Ostoma* to *colobicoides*-group)

*Ancyrona muellerae* Kolibáč, 2007; SA: Transvaal (JK)

Kolibáč, J. 2007b: 59 (*Ancyrona endroedyi*-group)

*Ancyrona nigrita* J. Thomson, 1858; Gabon (AL)

Léveillé, A. 1910: 26. Reitter, E. 1876: 53 (*Peltis*)

*Ancyrona obscura* Léveille, 1899; Sumatra, Ternate (AL)

Léveillé, A. 1910: 26

*Ancyrona orbicularis* Léveille, 1894; Sumatra, Ternate (AL)

Léveillé, A. 1910: 26

*Ancyrona ovalis* M’Leay, 1825; Borneo, Java (AL)

Léveillé, A. 1910: 25. Reitter, E. 1876: 49 (comb.; transferred from *Peltis* to *Latolaeva*)

*Ancyrona plana* Léveille, 1902; E Africa (AL)

Léveillé, A. 1910: 26. Kolibáč, J. 2007b: 55 (shifted to *gabonica*-group)

*Ancyrona pryeri* Olliff, 1883; Borneo (AL)

Léveillé, A. 1910: 26

*Ancyrona pygmea* Léveille, 1907; Argentina (?) (AL)

Léveillé, A. 1910: 26

*Ancyrona quadrimaculata* Reitter, 1877; Malacca (AL)

Léveillé, A. 1910: 25

*Ancyrona reitteri* Olliff, 1883; New Guinea, Ins. Aru (AL)

Léveillé, A. 1910: 26

*Ancyrona rufolineata* Léveille, 1899; Cameroon (AL)

Léveillé, A. 1910: 26

*Ancyrona shibatai* Nakane, 1963; Japan (varA)

Nakane, T. 1963: 46. Kolibáč, J. 2007a: 365. Kolibáč, J. 2007b: 54 (shifted to *lewisi*-group). Nakane, T. et al. 1963: 182

*Ancyrona simoni* Reitter, 1880; Ashantee (AL)

Léveillé, A. 1910: 26

*Ancyrona soror* Léveille, 1902; Sumatra (AL)

Léveillé, A. 1910: 26

*Ancyrona subrotundata* Motschulsky, 1863; Sri Lanka (AL)

Léveillé, A. 1910: 26. Reitter, E. 1876: 53 (*Ostoma*)

*Ancyrona vesca* Olliff, 1885; Australia (AL)

Léveillé, A. 1910: 27

*Ancyrona vicina* Léveille, 1899; Cameroon (AL)

Léveillé, A. 1910: 27. Kolibáč, J. 2007b: 55 (shifted to *gabonica*-group)

#### Genus Grynoma

Sharp, 1877

http://species-id.net/wiki/Grynoma

Fig. 10 ; Map 12

Grynoma Sharp, D. 1877: 267

##### Type species.

*Grynoma diluta* Sharp, 1877 [designated by Kolibáč 2005]

Léveillé, A. 1910: 29. Crowson, R. A. 1964a: 299 (as *Grynoma* Broun). Kolibáč, J. 2005: 59. Kolibáč, J. 2006: 111 (phylogeny)

##### Description.

Body size: about 5.0–5.5 mm. Body shape flat. Gular sutures wide, convergent at apex. Frontoclypeal suture present. Frons: longitudinal groove or depression absent. Cranium ventrally: tufts of long setae at sides absent. Submentum: ctenidium absent. Antennal groove absent. Eyes: size large, lateral. Eyes number: two. Epicranial acumination moderate. Lacinial hooks absent. Galea: shape sub-clavate. Galea: ciliate setae absent. Mediostipes-Lacinia partially fused. Palpifer: outer edge even. Mandibular apical teeth number: two, horizontally situated. Mola absent. Penicillus (at base) long setae. Pubescence above mola or cutting edge absent. Ventral furrow absent. Basal notch shallow or absent. Labrum-Cranium fused. Epipharyngial sclerite absent. Lateral tormal process: projection projection not developed (all remaining). Ligula: ciliate setae absent. Ligula membranous, not retroflexed, weakly emarginate. Hypopharyngeal sclerite consisting of two separate parts. Antenna 10-segmented. Antennal club weakly asymmetrical, sensorial fields absent. Front coxal cavities externally open, internally open. Pronotum transverse. Prepectus present. Middle coxal cavities open. Elytra: long hairs absent. Epipleuron moderate. Elytral interlocking mechanism absent, carinae reduced. Elytral punctation irregular, scales absent. Wing: radial cell moved down, often small, wedge cell absent, cross vein MP3-4 absent, cross vein AA1+2-3+4 absent. Front tibiae: spines along side moderate. Hooked spur absent, apical spurs not hooked or weakly hooked. Claws: denticle absent. Parasternites number along ventrites III–VII: one. Spiculum gastrale absent. Tegmen composed of three parts.

Larva: Paragular sclerites absent. Cardo-Stipes not fused. Cardo: size nearly as large as stipes. Torma H-shaped. Abdominal segment IX transversely divided. Tergite IX flat. Urogomphi present, hooked; median process present.

##### Biology.

Crowson (1964a) found “*only detrital material and vegetable fibres*” in the gut of adult *Grynoma varians*; however, there were “*insect fragments as well as detrital material*” in the gut of a larva of the same species. I assume that the adults and larvae are predatory.

##### Distribution.

New Zealand.

##### Species:

*Grynoma albosparsa* Broun, 1909; New Zealand (AL)

Léveillé, A. 1910: 29

*Grynoma ambiguum* Broun, 1880; New Zealand (AL)

Léveillé, A. 1910: 21 (*Leperina ambigua*). Leschen, R. A. B. & Lackner, T. 2013: 301 (comb. from *Leperina*)

*Grynoma diluta* Sharp, 1877; New Zealand (AL)

Léveillé, A. 1910: 29. Kolibáč, J. 2005: 59 (redescription)

*Grynoma fusca* Sharp, 1877; New Zealand (AL)

Léveillé, A. 1910: 29

*Grynoma regularis* Sharp, 1882; New Zealand (AL)

Léveillé, A. 1910: 29

*Grynoma varians* Broun, 1893; New Zealand (RAC)

Crowson, R. A. 1964a: 297 (adult and larva). Kolibáč, J. 2006: 107 (larva)

Note: The species was omitted by Léveillé (1910).

#### Genus Leptonyxa

Reitter, 1876

http://species-id.net/wiki/Leptonyxa

Fig. 11 ; Map 12

Leptonyxa Reitter, E. 1876: 54.

##### Type species.

*Leptonyxa brevicollis* Reitter, 1876 [designated by Kolibáč 2005]

Léveillé, A. 1910: 27. Kolibáč, J. 2005: 66. Kolibáč, J. 2006: 111 (phylogeny)

##### Remarks.

I have studied only two species of *Leptonyxa*, *Leptonyxa germaini* and *Leptonyxa fairmairei*. The mandibles of the males are so peculiar that I hesitated over whether the genus truly belongs in Trogossitidae (Kolibáč 2006). Moreover, the other mouthparts – the reduced labrum, labium with divided prementum and plain galea and lacinia without sclerotized spurs or thorns – are also so highly modified that it is difficult to see trogossitid features in these body parts at all. More extensive material of *Leptonyxa* should be examined to establish a definitive morphological description of the genus.

##### Description.

Body size: about 7.0 mm. Body shape flat. Gular sutures wide, convergent at apex. Frontoclypeal suture absent. Frons: longitudinal groove or depression absent. Cranium ventrally: tufts of long setae at sides present. Submentum: ctenidium absent. Antennal groove absent. Eyes: size large, lateral. Eyes number: two. Epicranial acumination absent. Lacinial hooks absent. Galea: shape elongate. Galea: ciliate setae absent. Mediostipes-Lacinia fused together. Palpifer: outer edge even. Mandibular apical teeth number: two, horizontally situated. Mola absent. Penicillus (at base) absent. Pubescence above mola or cutting edge present. Ventral furrow present. Basal notch shallow or absent. Labrum-Cranium fused. Epipharyngial sclerite absent. Lateral tormal process: projection projection not developed (all remaining). Ligula: ciliate setae absent. Ligula rigid, not retroflexed, weakly emarginate. Hypopharyngeal sclerite absent. Antenna 10-segmented. Antennal club weakly asymmetrical, sensorial fields absent. Front coxal cavities externally open, internally open. Pronotum transverse. Prepectus present. Middle coxal cavities open. Elytra: long hairs absent. Epipleuron moderate. Elytral interlocking mechanism absent, carinae conspicuous. Elytral punctation regular, scales absent. Wing: radial cell moved down, often small, wedge cell absent, cross vein MP3-4 absent, cross vein AA1+2-3+4 absent. Front tibiae: spines along side moderate. Hooked spur present. Claws: denticle absent. Parasternites number along ventrites III–VII: absent. Spiculum gastrale absent. Tegmen composed of three parts. Coxitae undivided.

##### Biology.

Nothing is known of the biology of these rare species. The last record known to me was made by a flight interception trap in Brazil. They are probably predatory.

##### Distribution.

Tropical South America: Bolivia, Colombia, Brazil.

##### Species:

*Leptonyxa boliviensis* Léveillé, 1895; Bolivia (AL)

Léveillé, A. 1910: 27

*Leptonyxa brevicollis* Reitter, 1876; Colombia (AL)

Reitter, E. 1876: 54. Léveillé, A. 1910: 27. Kolibáč, J. 2005: 66 (redescription)

*Leptonyxa costipennis* Reitter, 1876; Brazil (AL)

Reitter, E. 1876: 55. Léveillé, A. 1910: 27

*Leptonyxa fairmairei* Léveillé, 1892; Brazil (AL)

Léveillé, A. 1910: 27. Kolibáč, J. 2006: 152

*Leptonyxa germaini* Léveillé, 1895; Bolivia (AL)

Léveillé, A. 1910: 27

*Leptonyxa grouvellei* Léveillé, 1895; Brazil (AL)

Léveillé, A. 1910: 27

*Leptonyxa ornata* Léveillé, 1895; Brazil: Bahia (AL)

Léveillé, A. 1910: 27

*Leptonyxa sedilloti* Léveillé, 1888; Colombia (AL)

Léveillé, A. 1910: 27

*Leptonyxa variegata* Léveillé, 1907; Brazil (AL)

Léveillé, A. 1910: 27

#### Genus Neaspis

Pascoe, 1872

http://species-id.net/wiki/Neaspis

Fig. 11 ; Map 12

Neaspis Pascoe, F. P. 1872: 317.

##### Type species.

*Neaspis villosa* Pascoe, 1872 [by monotypy]

Léveillé, A. 1910: 25. Matthews, E. G. 1992: 4. Kolibáč, J. 2005: 70. Kolibáč, J. 2006: 111 (phylogeny). Reitter, E. 1876: 47

##### Description.

Body size: about 4.0 mm. Body shape flat. Gular sutures wide, convergent at apex. Frontoclypeal suture absent. Frons: longitudinal groove or depression absent. Cranium ventrally: tufts of long setae at sides absent. Submentum: ctenidium absent. Antennal groove absent. Eyes: size moderate. Eyes number: two. Epicranial acumination absent. Lacinial hooks absent. Galea: shape sub-clavate. Galea: ciliate setae absent. Mediostipes-Lacinia not fused. Palpifer: outer edge even. Mandibular apical teeth number: two, horizontally situated. Mola absent. Penicillus (at base) long setae. Pubescence above mola or cutting edge absent. Ventral furrow absent. Basal notch shallow or absent. Labrum-Cranium not fused. Epipharyngial sclerite absent. Lateral tormal process: projection projection not developed (all remaining). Ligula: ciliate setae absent. Ligula rigid, not retroflexed, weakly emarginate. Hypopharyngeal sclerite H-shaped. Antenna 10-segmented. Antennal club symmetrical, sensorial fields absent. Front coxal cavities externally open, internally open. Pronotum transverse. Middle coxal cavities closed. Elytra: long hairs absent. Epipleuron moderate. Elytral interlocking mechanism absent, carinae reduced. Elytral punctation regular, scales present. Wing: radial cell moved down, often small, wedge cell absent, cross vein MP3-4 absent, cross vein AA1+2-3+4 absent. Front tibiae: spines along side moderate. Hooked spur present. Claws: denticle absent. Spiculum gastrale absent. Tegmen composed of three parts. Coxitae undivided.

##### Biology.

The species are probably predatory. According to Matthews (1992), they live in dry sclerophyll and Eremaean zones.

##### Distribution.

The genus is autochthonous in Australia; *Neaspis squamata* from the Phillippines is probably mislabelled, misidentified or introduced (I did not examine the species). Recently, I studied a specimen of *Neaspis* cf. *variegata* collected in Brazil by Wygodzinski (coll. National Museum Prague), labelled as “Corcovado, Rio D.F.” (= Rio de Janeiro, former Districto Federal). This record is believed to be plausible. The presence (either autochthonous distribution or introduction) might be the grounds for certain improbable descriptions of South American *Ancyrona* species.

##### Species:

*Neaspis pusilla* Blackburn, 1891; South Australia (AL)

Léveillé, A. 1910: 25

*Neaspis sculpturata* Reitter, 1876; Australia (AL)

Léveillé, A. 1910: 25. Reitter, E. 1876: 48

*Neaspis serrata* Léveillé, 1907; Queensland (AL)

Léveillé, A. 1910: 25

*Neaspis squamata* Escherich, 1822; Philippines: Luzon (AL)

Léveillé, A. 1910: 25. Reitter, E. 1876: 49

Note: doubtful record.

*Neaspis variegata* MacLeay, 1873; Australia, one specimen from Brazil (varA)

Léveillé, A. 1910: 25. Kolibáč, J. 2005: 70 (redescription). Reitter, E. 1876: 47 (*Neaspis subtrifasciata* Reitter, 1876)

*Neaspis villosa* Pascoe, 1872; Australia (AL)

Léveillé, A. 1910: 25. Reitter, E. 1876: 48

#### † Genus Sinosoronia

Zhang, 1992

http://species-id.net/wiki/Sinosoronia

Map 12

Nitidulidae Zhang, J.-F. 1992: 333 [in Chinese], 336 [in English] (sub )

##### Type species:

*Sinosoronia longiantennata* Zhang, 1992 [by monotypy]

Kolibáč, J. 2006: 136 (Trogossitidae *incertae sedis*). Kolibáč, J. & Huang, D.-Y. 2008: 145 (Ancyronini). Ponomarenko, A. G. & Kireichuk, A. G. (2005–2008): http://www.zin.ru/animalia/Coleoptera/rus/paleosy2.htm (Peltidae). Zhang, J.-F. 1992: 333 [in Chinese], 336 [in English]. Schmied, H. et al. 2009: 26

##### Remarks.

*Sinosoronia* might be related to another Mesozoic genus, *Peltocoleops*. The latter genus was described as “Cleroidea *incertae sedis*” (Ponomarenko 1990) and classified within Lophocaterini by myself (Kolibáč 2006). The two genera differ distinctly in the shape of the antennal club. This is compact and 3-segmented, with segments weakly asymmetrical in *Peltocoleops* but loose, 2- or 3-segmented, with segments distinctly asymmetrical in *Sinosoronia*. It is therefore suggested that the latter genus be classified within the tribe Ancyronini, which has corresponding features in recent representatives. The large, elevated eyes observed in *Sinosoronia* also support such a classification (according to Kolibáč and Huang 2008).

The “posterior femur” in the original description is probably the hind coxa. The long antenna with a loose club resembles that of species of the *Ancyrona gabonica* species-group, while a similar shape of the pronotum may be found in the *colobicoides* species-group. Such an extremely small size of body is not known in recent Ancyronini but occurs in an concurrently described species from the late middle Eocene (Schmied et al. 2009). Apart from body size, the two species share large, elevated eyes and similar shape of pronotum. The time difference between these two very similar species is about 100 million years, much more than between the Eocene and the present time. Round body and body size might appear indicative of a group of the rentoniine genera. However, the body is much smaller (about 1 mm) and the antennae shorter with a symmetrical club in the rentoniins (Kolibáč 2005). If the asymmetrical club is considered an apomorphy, *Sinosoronia* may well be an ancestor of Ancyronini rather than Thymalini (according to Kolibáč and Huang 2008).

##### Original description.

“*Brown in colour. Head about as long as wide. Mandibles large but dentes indistinguishable. Eyes circular, expanded laterally but exterior margin ill-preserved. Antennae 1.2 times as long as head and pronotum together, several basal segments ill-preserved except for the thickened scape, each flagellum cylindrical, about twice as long as wide, club elongate, nearly one-third the length of antenna, slightly thickened apically. Pronotum 2.1 times as broad as long; anterior margin arched, its median part straight, curved forwards laterally, lateral margins arched, posterior margin sinuate, and closely connected to elytra. Scutellum about as long as wide. Elytra smooth, not striated, exterior and interior margins slightly arched, shoulder rounded, its terminal part distinctly exceeding apex of abdomen, each elytron 2.6 times as long as wide. Middle and posterior femora seemingly clubbed, both tibiae and tarsi absent. Total length 2.3 mm, width 1.3 mm.*” (Zhang 1992: 336.)

##### Distribution.

China: Shandong province; Mesozoic: Lower Cretaceous, Laiyang formation.

##### Species:

† *Sinosoronia longiantennata* Zhang, 1992; China: Shandong; Lower Cretaceous: Laiyang formation (varA)

Kolibáč, J. 2006: 136. Kolibáč, J. & Huang, D.-Y. 2008: 145. Ponomarenko, A. G. & Kireichuk, A. G. (2005–2008): http://www.zin.ru/animalia/Coleoptera/rus/paleosy2.htm. Schmied, H. et al. 2009: 26. Zhang, J.-F. 1992: 333 [in Chinese], 336 [in English]

#### Tribe Lophocaterini

Crowson, 1964

Lophocaterinae Crowson, R. A. 1964a: 297 ( ).

##### Type genus:

*Lophocateres* Olliff, 1883

Barron, J. R. 1971: 11, 12 (syn. Lophocateridae = Peltinae). Barron, J. R. 1975: 1119. Burakowski, B. et al. 1986: 119 (Lophocateridae). Kolibáč, J. 2006: 128 (diagnosis, stat. n.). Kolibáč, J. 2007a: 365. Kolibáč, J. 2010: 35. Lafer, G. Sh. 1992: 83 (key). Lawrence, J. F. & Newton, A. F., Jr. 1995: 868 (Lophocateridae). Lucht, W. 1998: 207 (key). Ślipiński, S. A. 1992: 442 (Lophocaterinae)

Lycoptini Casey, 1890 (Type genus: *Lycoptis* Casey, 1890)

Kolibáč, J. 2006: 128 (synonymized)

##### Remarks.

The main issue to be addressed for Lophocaterini is their possible paraphyly in relation to Ancyronini. The whole clade (lophocaterins + ancyronins) is monophyletic but the lophocaterins might be paraphyletic (i.e., non-holophyletic in the traditional Hennigian meaning) because ancyronins can only be advanced members of more primitive lophocaterins. See also “Remarks” in the Ancyronini section. Further, more detailed study is required to resolve the question. The generic composition of Decamerini and its position within Lophocaterinae should be examined along – not, however, before an associated larva of the decamerins is known.

##### A key to genera

(after Kolibáč 2010)

**Table:** 

1	Elytra with irregular punctation; lateral margins of pronotum broadly explanate, lateral edge sparsely denticulate	*Eronyxa*
–	Elytra regularly punctate; lateral margins of pronotum narrowly explanate, lateral edge almost entirely evenly rounded or densely denticulate	2
2	Antenna 7- or 9-segmented	3
–	Antenna 11-segmented	5
3	Antenna 7-segmented, club 1-segmented; mandible with mola	*Lycoptis*
–	Antenna 9-segmented, club 2- or 3-segmented; mandible without mola	4
4	Antennal club 2-segmented; mandible with prostheca near base of mandible formed by tuft of long setae; submental area lacking concave or depressed area; wing with oblong radial cell	*Grynocharina*
–	Antennal club 3-segmented; mandible without penicillus or prostheca; submental area concave; wing with small triangular radial cell displaced downwards	*Peltonyxa*
5	Lateral edge of pronotum densely denticulate; lacinia with one pigmented spine	*Indopeltis*
–	Lateral edge of pronotum evenly rounded or at most finely undulating; lacinia with two or three pigmented spines	6
6	Elytra with inconspicuous carinae; mola absent; ligula deeply emarginate; probably predatory. Larva: sensory appendix very short	*Promanus*
–	Elytra with conspicuous carinae; mola or remnant of mola present; ligula deeply or weakly emarginate. Mode of life: predatory (*Trichocateres*), herbivorous (*Lophocateres*), fungivorous (*Grynocharis*). Larva (*Lophocateres*, *Grynocharis*): length of sensory appendix about half or more than half that of antennal segment 3	7
7	Elytra with six carinae; tegmen without projecting phallobasic apodeme; lacinia with three pigmented, hooked spines; small species (less than 3 mm)	*Lophocateres*
–	Elytra with five or four distinct carinae; tegmen with projecting phallobasic apodeme; different pattern of pigmented lacinial spines; larger species (above 5 mm)	8
8	Elytra with four distinct (higher) carinae and another three to four lower carinae among them; pronotum and elytra without tufts of long hairs, with short decumbent or semi-erect pubescence only, or without conspicuous pubescence; lacinia with two pigmented, hooked spines; tibial apical spur pattern 2-2-2; larger species (about 4.5–10.5 mm)	*Grynocharis*
–	Elytra with only five distinct carinae; pronotum and elytra with tufts of long, yellow-orange hairs; lacinia with three pigmented spines at apex in pattern 1+2, apical spine large and hooked, two other spines much smaller; tibial apical spur pattern 1-1-1; smaller species (about 5 mm)	*Trichocateres*

#### Genus Eronyxa

Reitter, 1876

http://species-id.net/wiki/Eronyxa

**Map 13.**
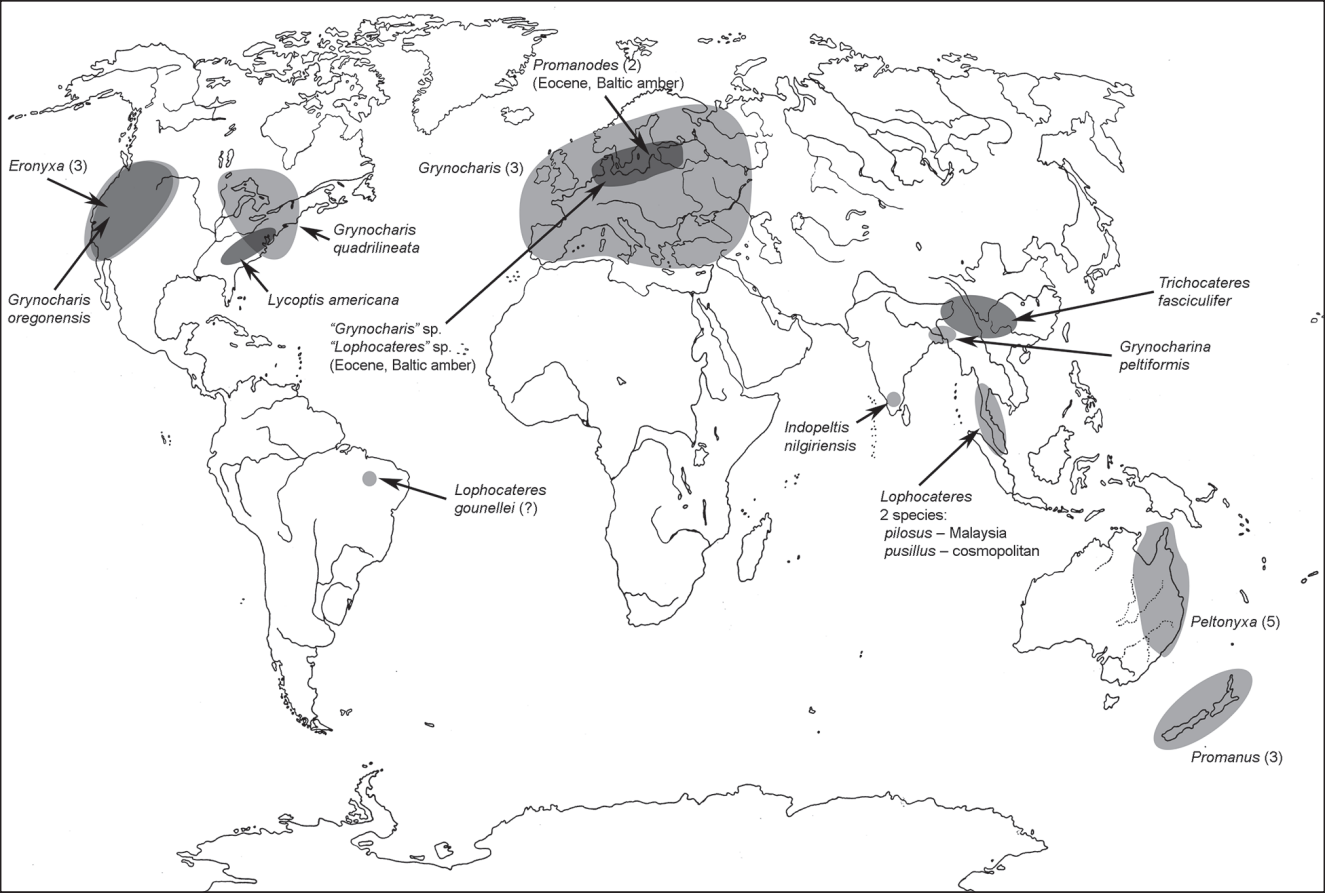
A distribution of the tribe Lophocaterini.

Fig. 10 ; Map 11 , 13

Eronyxa Reitter, E. 1876: 57.

##### Type species:

*Ostomodes dohrni* Reitter, 1876 [by monotypy]

Barron, J. R. 1971: 38. Reitter, E. 1876: 57. Kolibáč, J. 2005: 55 (redescription). Kolibáč, J. 2006: 111 (phylogeny). Kolibáč, J. 2010: 35 (key). Léveillé, A. 1910: 28

*Ostomodes* Reitter, 1877 (Type species: *Ostomodes dohrni* Reitter, 1876)

Barron, J. R. 1971: 38. Léveillé, A. 1910: 28. Schaeffer, C. F. A. 1915: 69. Schaeffer, C. F. A. 1918: 200. Casey, 1916: 284. Crowson, R. A. 1964a: 290. Crowson, R. A. 1966: 125

*Grynocharis* (pars.)

Barron, J. R. 1971: 38. Van Dyke, E. C. 1916: 73

##### Remarks.

The genus was formerly classified within Decamerini (Crowson 1964a and others). John Doyen (in Tait et al. 1990) described a larva of *Eronyxa expansus* and, considering its distinct similarity to known lophocaterine larvae, shifted *Eronyxa* to Lophocaterini. Although this classification was confirmed by myself (Kolibáč 2006, 2008), both character analyses showed *Eronyxa* in a basal position of the lophocaterine clade (= Lophocaterini + Ancyronini) along a border near the Decamerini-Lophocaterini split. However, the adult characters indicate a sister relationship with Decamerini [(*Eronyxa* (*Diontolobus* + *Decamerus*)) (Lophocaterini)] rather than Lophocaterini [(*Diontolobus* + *Decamerus*) (*Eronyxa* + Lophocaterini)]. See also “Remarks” in the Decamerini section.

##### Description.

Body size: about 3.5 mm. Body shape flat. Gular sutures wide, convergent at apex. Frontoclypeal suture present. Frons: longitudinal groove or depression absent. Cranium ventrally: tufts of long setae at sides absent. Submentum: ctenidium absent. Antennal groove absent. Eyes: size large, lateral. Eyes number: two. Epicranial acumination moderate. Lacinial hooks: two. Galea: shape very small. Galea: ciliate setae absent. Mediostipes-Lacinia not fused. Palpifer: outer edge even. Mandibular apical teeth number: two, horizontally situated. Mola present. Penicillus (at base) present (fine, often membranous). Pubescence above mola or cutting edge absent. Ventral furrow absent. Basal notch moderate. Labrum-Cranium not fused. Epipharyngial sclerite absent. Lateral tormal process: projection projections extending laterally and downwards (Eronyxa). Ligula: ciliate setae absent. Ligula membranous, not retroflexed, deeply emarginate. Hypopharyngeal sclerite absent. Antenna 11-segmented. Antennal club symmetrical, sensorial fields absent. Front coxal cavities externally open, internally open. Pronotum transverse. Prepectus present. Middle coxal cavities open. Elytra: long hairs absent. Epipleuron thin. Elytral interlocking mechanism absent, carinae reduced. Elytral punctation irregular, scales absent. Wing: radial cell moved down, often small, wedge cell absent, cross vein MP3-4 present, cross vein AA1+2-3+4 absent. Front tibiae: spines along side large. Hooked spur absent, apical spurs not hooked or weakly hooked. Claws: denticle absent. Parasternites number along ventrites III–VII: two. Spiculum gastrale absent. Tegmen composed of two parts. Coxitae divided.

Larva: Frontal arms V-shaped. Epicranial stem absent. Endocarina present. Gular sutures conspicuous, convergent. Paragular sclerites absent. Hypostomal rods absent. Stemmata number: two. Mandibular apical teeth number: two, horizontally even, vertically situated. Lacinia mandibulae plumose. Mola absent. Maxillary palpi 3-segmented. Palpifer present. Pedunculate seta absent. Mala simple. Mala: bidentate protrusion absent. Cardo-Stipes partially fused. Ligula present. Labial palpi 2-segmented. Prementum in two parts. Torma H-shaped. Antennal joints 1 and 2 elongate. Sensory appendix very small. Thoracic sclerites pattern (dorsally) 1-0-0. Thoracic sclerites pattern (ventrally) 1+0+0. Trochanter triangular. Abdominal segment IX transversely divided. Tergite IX flat. Urogomphi present, hooked; median process present.

##### Biology.

*Eronyxa expansus* was collected under the bark of *Libocedrus* (= *Calocedrus*) *decurrens*. The larva probably feeds on *Xylococculus macrocarpae* (Barron 1971, Tait et al. 1990). *Eronyxa pallidus* has been found on flowers, for example *Aruncus sylvester* and *Ceanothus cuneatus*, and an imago was reared from a stem of the latter plant. The third species, *Eronyxa angustus*, was also found on *Fraxinus* blossoms and on *Pinus ponderosa* (Barron 1971).

##### Distribution.

Western states of USA (California, Idaho, Nevada, Oregon) and Canada (British Columbia).

##### Species:

*Eronyxa angustus* Casey, 1916; USA: California, Idaho, Nevada, Oregon (JRB)

Barron, J. R. 1971: 42. Kolibáč, J. 2005: 55 (redescription)

*Eronyxa expansus* Van Dyke, 1916; USA: California (JRB)

Barron, J. R. 1971: 38. Kolibáč, J. 2006: 107 (larva). Leschen, R. A. B. 2000: 920 (biology). Tait, S. M. et al. 1990: 13 (larva)

*Eronyxa pallida* Motschulsky, 1863; Canada: British Columbia, USA: California, Oregon (JRB)

Léveillé, A. 1910: 28. Barron, J. R. 1971: 39 (syn. *Grynocharis pilosula* Crotch, 1873; synonymized by whom?). Barron, J. R. 1971: 39 (syn. *Ostomodes dohrni* Reitter, 1877; synonymized by Léveillé 1910?). Barron, J. R. 1971: 39 (syn. *Ostomodes lagrioides* Reitter, 1876; synonymized by whom?). Crowson, R. A. 1964a: 291 (*Ostomodes*). Kolibáč, J. 2005: 55 (redescription). Reitter, E. 1876: 58 (*Eronyxa lagrioides*)

#### Genus Grynocharina

Reitter, 1877

http://species-id.net/wiki/Grynocharina

Fig. 12 ; Map 13

Grynocharina Reitter, E. 1877: 132.

##### Type species.

*Grynocharina peltiformis* Reitter, 1877 [by monotypy]

Léveillé, A. 1910: 24. Kolibáč, J. 2005: 57 (redescription). Kolibáč, J. 2006: 111 (phylogeny). Kolibáč, J. 2010: 35 (key)

##### Remarks.

Only a single male specimen of the monotypic genus is known. *Grynocharina peltiformis* was originally placed in a basal position on the lophocaterine tree (Kolibáč 2006: 131) and classified within Lophocaterini. However, that tree was constructed after character weighting. A tree under equal weights contains *Grynocharina peltiformis* among the ancyronins in both analyses (Kolibáč 2006, 2008). I maintain the position of the species in Lophocaterini, following the original classification of 2006 and especially the key of 2010 (*l.c.*) Regarding the probable paraphyletic status of Lophocaterini, the question of ancyronine/lophocaterine placement of *Grynocharina peltiformis* is rendered highly irrelevant.

##### Description.

Body size: 3.0 mm. Body shape flat. Gular sutures wide, convergent at apex. Frontoclypeal suture present. Frons: longitudinal groove or depression absent. Cranium ventrally: tufts of long setae at sides absent. Submentum: ctenidium absent. Antennal groove present. Eyes: size large, lateral. Eyes number: two. Epicranial acumination moderate. Lacinial hooks: three. Galea: shape sub-clavate. Galea: ciliate setae absent. Mediostipes-Lacinia not fused. Palpifer: outer edge even. Mandibular apical teeth number: two, horizontally situated. Mola absent. Penicillus (at base) long setae. Pubescence above mola or cutting edge absent. Ventral furrow present, not ciliate. Basal notch shallow or absent. Labrum-Cranium not fused. Epipharyngial sclerite absent. Lateral tormal process: projection projection curved upwards (*Colydiopeltis*). Ligula: ciliate setae absent. Ligula membranous, not retroflexed, weakly emarginate. Hypopharyngeal sclerite H-shaped. Antenna 9-segmented. Antennal club weakly asymmetrical, sensorial fields absent. Front coxal cavities externally open, internally open. Pronotum transverse. Prepectus present. Middle coxal cavities open. Elytra: long hairs absent. Epipleuron thin. Elytral interlocking mechanism absent, carinae conspicuous. Elytral punctation regular, scales absent. Wing: radial cell oblong (or reduced), wedge cell absent, cross vein MP3-4 absent, cross vein AA1+2-3+4 absent. Front tibiae: spines along side moderate. Hooked spur present. Claws: denticle absent. Parasternites number along ventrites III–VII: two. Spiculum gastrale present. Tegmen composed of three parts.

##### Biology.

Unknown.

##### Distribution.

Burma (Myanmar).

##### Species:

*Grynocharina peltiformis* Reitter, 1877; Burma (AL)

Léveillé, A. 1910: 24. Kolibáč, J. 2005: 57 (redescription)

#### Genus Grynocharis

Thomson, 1862

http://species-id.net/wiki/Grynocharis

**Figure 12.**
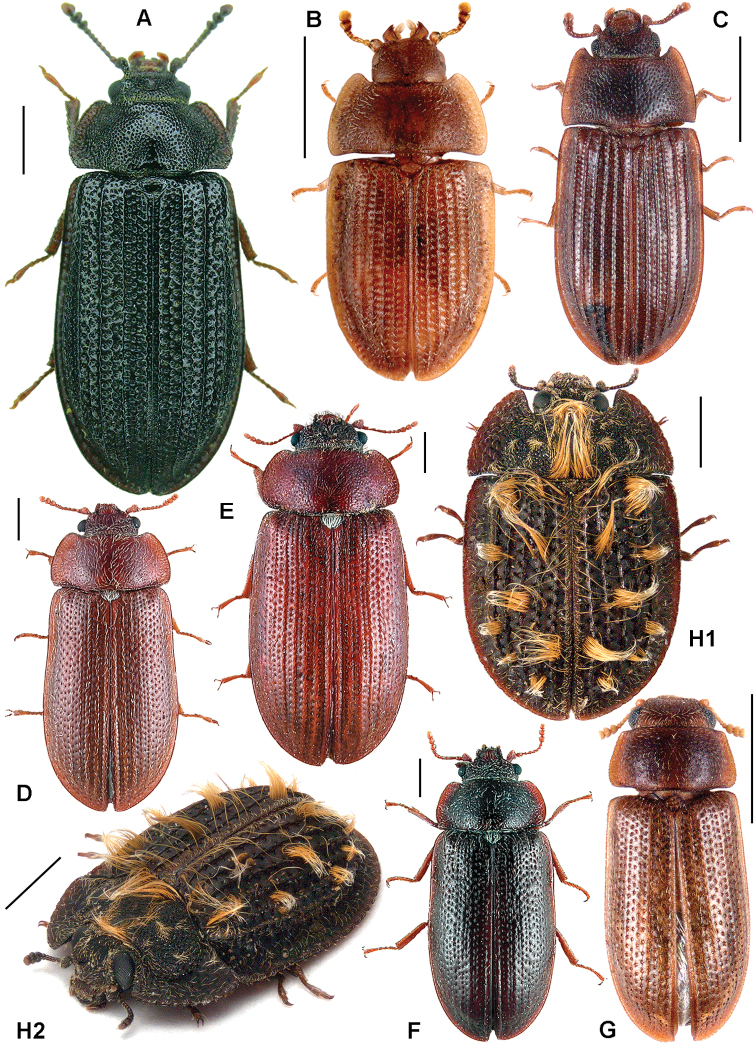
**A** *Grynocharis oblonga* **B** *Grynocharina peltiformis* **C** *Lophocateres pusillus* **D** *Promanus auripilis* **E** *Promanus subcostatus* **F** *Promanus depressus* **G** *Peltonyxa* sp., Australia, NSW **H** *Trichocateres fasciculifer*.

Figs 12 , 18 ; Map 13

Grynocharis Thomson, C. G. 1862: 71.

##### Type species.

*Silpha oblonga* Linnaeus, 1758 [by original designation and monotypy]

Léveillé, A. 1910: 31. Barron, J. R. 1971: 32. Kolibáč, J. 2005: 58 (redescription). Kolibáč, J. 2006: 111 (phylogeny). Kolibáč, J. 2007a: 365. Kolibáč, J. 2010: 35 (key). Lafer, G. Sh. 1992: 84. Larsson, S. G. 1978: 150 (Baltic amber fossil). Spahr, U. 1981: 74 (amber and copal fossils)

*Gaurambe* Thomson, 1859

Barron, J. R. 1971: 32 (syn. *Gaurambe* Thomson, 1859; misapplied)

##### Description.

Body size: about 5.5–8.0 mm. Body shape flat. Gular sutures wide, convergent at apex. Frontoclypeal suture broadly emarginate. Frons: longitudinal groove or depression absent. Cranium ventrally: tufts of long setae at sides absent. Submentum: ctenidium absent. Antennal groove present. Eyes: size moderate. Eyes number: two. Epicranial acumination deep. Lacinial hooks: two. Galea: shape sub-clavate. Galea: ciliate setae absent. Mediostipes-Lacinia not fused. Palpifer: outer edge even. Mandibular apical teeth number: two, horizontally situated. Mola reduced but present. Penicillus (at base) present (fine, often membranous). Pubescence above mola or cutting edge absent. Ventral furrow present, not ciliate. Basal notch shallow or absent. Labrum-Cranium not fused. Epipharyngial sclerite absent. Lateral tormal process: projection curved downwards, processes with bridge (*Peltis*). Ligula: ciliate setae absent. Ligula membranous, not retroflexed, weakly emarginate. Hypopharyngeal sclerite consisting of two separate parts. Antenna 11-segmented. Antennal club weakly asymmetrical, sensorial fields absent. Front coxal cavities externally open, internally open. Pronotum transverse. Prepectus present. Middle coxal cavities open. Elytra: long hairs absent. Epipleuron moderate. Elytral interlocking mechanism absent, carinae conspicuous. Elytral punctation regular, scales absent. Wing: radial cell moved down, often small, wedge cell absent, cross vein MP3-4 absent, cross vein AA1+2-3+4 absent. Front tibiae: spines along side moderate. Hooked spur present. Claws: denticle absent. Parasternites number along ventrites III–VII: two. Spiculum gastrale present. Tegmen composed of three parts.

Larva: Frontal arms V-shaped. Epicranial stem absent. Endocarina present. Stemmata number: two. Mandibular apical teeth number: two, horizontally situated. Lacinia mandibulae plumose. Mola absent. Maxillary palpi 3-segmented. Pedunculate seta absent. Mala simple. Mala: bidentate protrusion absent. Cardo-Stipes not fused. Cardo: size nearly as large as stipes. Ligula present. Labial palpi 2-segmented. Prementum in single part. Antennal joints 1 and 2 elongate. Sensory appendix medium sized (to half of joint 3). Thoracic sclerites pattern (dorsally) 1-2-2. Abdominal segment IX transversely divided. Tergite IX flat. Urogomphi present, hooked; median process present.

##### Biology.

Adults and larvae of *Grynocharis oblonga* live under bark or bark scales and in rotten wood of deciduous and coniferous trees (willow, birch, spruce, fir); they are fungivorous. In the USA, the species have been collected on *Libocedrus decurrens* and *Populus* (Barron 1971).

##### Distribution.

Europe including Russia to the Urals, Caucasus; USA excluding central and southern states, Canada: south-western and south-eastern states.

##### Species:

*Grynocharis caucasica* Motschulsky, 1863; Caucasus (JK)

Léveillé, A. 1910: 31 (*Ostoma*). Kolibáč, J. 2007a: 366 (nomen dubium)

*Grynocharis oblonga* Linnaeus, 1758; all Europe to Russia (varA)

Léveillé, A. 1910: 31 (*Ostoma* (subgen. *Grynocharis*)). Bahillo de la Puebla, P. & López-Colón, J. I. 2004: 129. Borowiec, L. 1983: 13. Burakowski, B. et al. 1986: 119. Conrad, R. 1995: 190. Gobbi, G. 1996: 65. Klausnitzer, B. 1976: 8. Klausnitzer, B. 1978: 178. Klausnitzer, B. 1996: 163. Kolibáč, J. 1993a: 21. Kolibáč, J. 1993b: 90. Kolibáč, J. 2005: 58 (redescription). Kolibáč, J. 2006: 107 (larva, phylogeny). Kolibáč, J. 2007a: 365 (distribution). Lafer, G. Sh. 1992: 84. Lemdahl, G. 2001: 39 (biology). Mitter, H. 1998: 561. Nilsson, S. G. 1997: 1 (biology). Pileckis, S. & Monsevičius, V. 1995: 272. Reitter, E. 1876: 63 (*Ostoma*). Vogt, H. 1967: 18

*Grynocharis oregonensis* Schaeffer, 1918; USA, Canada: western states (JRB)

Léveillé, A. 1910: 31 (*Ostoma* (subgen. *Grynocharis*) *oregonensis* Crotch, 1873). Barron, J. R. 1971: 34. Dajoz, R. 1997: 44 (biology)

*Grynocharis pubescens* Erichson, 1844; Georgia, South European Territory of Russia, „Caucasus”, Crimea (JK)

Léveillé, A. 1910: 31 (*Ostoma* (subgen. *Grynocharis*)). Lafer, G. Sh. 1992: 84. Kolibáč, J. 2006: 107. Kolibáč, J. 2007a: 365. Mamaev, B. M. 1976: 1656 (larva). Reitter, E. 1876: 63 (*Ostoma*)

*Grynocharis quadrilineata* Melsheimer, 1844; NE USA, Canada: Ontario, Quebec (JRB)

Léveillé, A. 1910: 31 (*Ostoma* (subgen. *Grynocharis*) *marginata* Melsheimer, 1844). Barron, J. R. 1971: 33 (syn. *Grynocharis marginata* Melsheimer, 1844, synonymized by Lacordaire 1854?). Reitter, E. 1876: 63 (*Ostoma*)

#### Genus Indopeltis

Crowson, 1966

http://species-id.net/wiki/Indopeltis

Map 13

Indopeltis Crowson, R. A. 1966: 126.

##### Type species:

*Indopeltis nilgiriensis* Crowson, 1966 [by original designation and monotypy]

Kolibáč, J. 2005: 61 (redescription). Kolibáč, J. 2006: 111 (phylogeny). Kolibáč, J. 2010: 35 (key)

##### Description.

Body size: about 5.5 mm. Body shape flat. Gular sutures wide, convergent at apex. Frontoclypeal suture absent. Frons: longitudinal groove or depression absent. Cranium ventrally: tufts of long setae at sides absent. Submentum: ctenidium absent. Eyes: size large, lateral. Eyes number: two. Epicranial acumination moderate. Lacinial hooks: one. Galea: shape elongate. Galea: ciliate setae absent. Mediostipes-Lacinia not fused. Palpifer: outer edge even. Mandibular apical teeth number: two, horizontally situated. Mola present. Penicillus (at base) absent. Pubescence above mola or cutting edge present. Ventral furrow present. Basal notch moderate. Labrum-Cranium not fused. Epipharyngial sclerite absent. Lateral tormal process: projection projection not developed (all remaining). Ligula: ciliate setae absent. Ligula membranous, not retroflexed, deeply emarginate. Hypopharyngeal sclerite H-shaped. Antenna 11-segmented. Antennal club weakly asymmetrical, sensorial fields absent. Front coxal cavities externally open, internally open. Pronotum transverse. Middle coxal cavities open. Elytra: long hairs absent. Epipleuron moderate. Elytral interlocking mechanism absent, carinae conspicuous. Elytral punctation regular, scales absent. Wing: radial cell moved down, often small, wedge cell absent, cross vein MP3-4 absent, cross vein AA1+2-3+4 absent. Front tibiae: spines along side moderate. Hooked spur present. Claws: denticle absent. Spiculum gastrale present. Tegmen composed of three parts.

##### Biology.

Unknown; Crowson (1966) speculated that the species was “*subcortical as in most Peltinae*”.

##### Distribution.

India: Tamil Nadu, Nilgiri Hills.

##### Species:

*Indopeltis nilgiriensis* Crowson, 1966; South India: Tamil Nadu (RAC)

Crowson, R. A. 1966: 126. Kolibáč, J. 2005: 61 (redescription). Kolibáč, J. 2006: 111 (phylogeny)

#### Genus Lophocateres

Olliff, 1883

http://species-id.net/wiki/Lophocateres

Figs 2 , 12 , 18 ; Map 13

Lophocateres Olliff, A. S. 1883c: 180.

##### Type species.

*Lophocateres nanus* Olliff, 1883 [by monotypy] (= *Lophocateres pusillus* Klug, 1833)

Léveillé, A. 1910: 27. Barron, J. R. 1971: 42. Crowson, R. A. 1964a: 299. Kolibáč, J. 2005: 67 (redescription). Kolibáč, J. 2006: 111 (phylogeny). Kolibáč, J. 2007a: 366. Kolibáč, J. 2010: 35 (key). Lafer, G. Sh. 1992: 84. Larsson, S. G. 1978: 150 (fossil, Baltic amber). Spahr, U. 1981: 74 (amber and copal fossils)

##### Description.

Body size: about 2.5–4.0 mm. Body shape flat. Gular sutures wide, convergent at apex. Frontoclypeal suture broadly emarginate. Frons: longitudinal groove or depression absent. Cranium ventrally: tufts of long setae at sides absent. Submentum: ctenidium absent. Antennal groove present. Eyes: size large, lateral. Eyes number: two. Epicranial acumination moderate. Lacinial hooks: three. Galea: shape sub-clavate. Galea: ciliate setae absent. Mediostipes-Lacinia not fused. Palpifer: outer edge even. Mandibular apical teeth number: two, horizontally situated. Mola present. Penicillus (at base) present (fine, often membranous). Pubescence above mola or cutting edge present. Ventral furrow absent. Basal notch moderate. Labrum-Cranium not fused. Epipharyngial sclerite absent. Lateral tormal process: projection projection curved upwards (*Colydiopeltis*). Ligula: ciliate setae absent. Ligula membranous, not retroflexed, weakly emarginate. Hypopharyngeal sclerite consisting of two separate parts. Antenna 11-segmented. Antennal club weakly asymmetrical, sensorial fields absent. Front coxal cavities externally open, internally open. Pronotum transverse. Prepectus present. Middle coxal cavities open. Elytra: long hairs absent. Epipleuron moderate. Elytral interlocking mechanism absent, carinae conspicuous. Elytral punctation regular, scales absent. Wing: radial cell moved down, often small, wedge cell absent, cross vein MP3-4 absent, cross vein AA1+2-3+4 absent. Front tibiae: spines along side moderate. Hooked spur present. Claws: denticle absent. Parasternites number along ventrites III–VII: one. Spiculum gastrale absent. Tegmen composed of three parts. Coxitae undivided.

Larva: Frontal arms V-shaped. Epicranial stem absent. Endocarina present. Gular sutures conspicuous, convergent. Gula: anterior apodemes absent. Paragular sclerites absent. Hypostomal rods absent. Stemmata number: two. Mandibular apical teeth number: two, horizontally situated. Lacinia mandibulae plumose. Mola absent. Maxillary palpi 3-segmented. Palpifer present. Pedunculate seta absent. Mala simple. Mala: bidentate protrusion absent. Cardo-Stipes not fused. Cardo: size nearly as large as stipes. Ligula present. Labial palpi 2-segmented. Prementum in single part, anterior margin even. Torma H-shaped. Antennal joints 1, 2 transverse. Sensory appendix larger than half of joint 3. Thoracic sclerites pattern (dorsally) 0+0+0. Thoracic sclerites pattern (ventrally) 2+0+0. Trochanter triangular. Abdominal segment IX transversely divided. Tergite IX flat. Urogomphi present, hooked; median process present.

##### Biology.

*Lophocateres pusillus* lives in storage facilities (warehouses, stores, barns, larders) and feeds on grains. The biology of wild populations is unknown.

##### Distribution.

*Lophocateres pusillus* is cosmopolitan, although the centre of its distribution, is in south-eastern Asia. The species *Lophocateres gounellei* is probably a misidentification or a synonym of *Lophocateres pusillus*. Some new species are known to me from Malaysia.

##### Species:

*Lophocateres gounellei* Léveillé, 1905; Brazil (AL)

Léveillé, A. 1910: 27

Note: dubious species – probably synonym of *Lophocateres pusillus* or different genus.

*Lophocateres pilosus* Olliff, 1883; Malaysia: Penang (AL)

Léveillé, A. 1910: 27

*Lophocateres pusillus* Klug, 1833; cosmopolitan (origin in SE Asia) (varA)

Léveillé, A. 1910: 27 (syn. *Lophocateres africanus* Motschulsky, 1863); Algeria (AL)

Léveillé, A. 1910: 27 (as *Lophocateres nanus* Olliff, 1883); Borneo (AL)

Léveillé, A. 1910: 27 (syn. *Lophocateres yvani* Allibert, 1847). Bahillo de la Puebla, P. & López-Colón, J. I. 2004: 129. Barron, J. R. 1971: 43 (syn. *Lophocateres nanus* Olliff, 1883). Barron, J. R. 1971: 43 (syn. *Peltis africanus* Motschulsky, 1863). Barron, J. R. 1971: 43 (syn. *Peltis yvani* Allibert, 1847). Borowiec, L. 1983: 14. Burakowski, B. et al. 1986: 119. Chang, T-C. & Liu, T.-Y. 1981: 116 (biology). Ghosh, S. & Haldar, D. P. 1989: 49 (biology). Ghosh, S. & Saha, K. 1992a: 181 (biology). Ghosh, S. & Saha, K. 1992b: 613 (biology). Ghosh, S. & Saha, K. 1995: 207 (biology). Halstead, D. G. H. 1968: 197 (biology). Klausnitzer, B. 1976: 7. Klausnitzer, B. 1978: 177. Klausnitzer, B. 1996: 163 (larva). Kolibáč, J. 1993a: 21. Kolibáč, J. 1999b: 12. Kolibáč, J. 2005: 67 (redescription). Kolibáč, J. 2006: 107 (larva). Kolibáč, J. 2007a: 366 (distribution). Lafer, G. Sh. 1992: 86. Nakane, T. et al. 1963: 182. Vogt, H. 1967: 16. Reitter, E. 1876: 63 (*Ostoma yvani*)

#### Genus Lycoptis

Casey, 1890

http://species-id.net/wiki/Lycoptis

Map 13

Lycoptis Casey, T. L. 1890: 311, 494.

##### Type species.

*Peltis americana* Motschulsky, 1863 [by monotypy]

Léveillé, A. 1910: 33. Barron, J. R. 1971: 120. Barron, J. R. 1975: 1117. Kolibáč, J. 2005: 67 (redescription). Kolibáč, J. 2006: 111 (phylogeny). Kolibáč, J. 2010: 35 (key)

##### Description.

Body size: 1.9–2.2 mm. Body shape flat. Gular sutures wide, convergent at apex. Frontoclypeal suture broadly emarginate. Frons: longitudinal groove or depression absent. Submentum: ctenidium absent. Antennal groove absent. Eyes: size large, lateral. Eyes number: two. Lacinial hooks: three. Galea: shape elongate. Galea: ciliate setae absent. Mediostipes-Lacinia partially fused. Palpifer: outer edge even. Mandibular apical teeth number: two, horizontally situated. Mola present. Penicillus (at base) absent. Pubescence above mola or cutting edge absent. Ventral furrow absent. Basal notch moderate. Labrum-Cranium not fused. Ligula: ciliate setae absent, not retroflexed, deeply emarginate. Antenna 7-segmented, sensorial fields absent. Front coxal cavities externally open, internally open. Pronotum transverse. Elytra: long hairs absent. Epipleuron moderate. Elytral interlocking mechanism absent, carinae conspicuous. Elytral punctation regular, scales absent. Wing: radial cell moved down, often small, wedge cell absent, cross vein MP3-4 absent, cross vein AA1+2-3+4 absent. Front tibiae: spines along side moderate. Hooked spur present. Claws: denticle absent. Spiculum gastrale present. Tegmen composed of two parts.

##### Biology.

*Lycoptis americana* is a very rare beetle, probably fungivorous. Barron (1975) noted that it was “*collected under bark of* Carya”.

##### Distribution.

USA: Georgia, Maryland, North Carolina, South Carolina (Barron 1975).

##### Species:

*Lycoptis americana* Motschulsky, 1863; USA: Georgia, Maryland, N Carolina, S Carolina (JRB)

Léveillé, A. 1910: 27 (*Lophocateres americanus* Motschulsky, 1863). Léveillé, A. 1910: 33 (*Lycoptis villosa* Casey, 1890). Barron, J. R. 1971: 120 (syn. *Lycoptis villosa* Casey, 1890). Barron, J. R. 1971: 120 (*Peltis americana* Motschulsky, 1863; comb.). Barron, J. R. 1975: 1120. Kolibáč, J. 2005: 67 (redescription)

#### Genus Peltonyxa

Reitter, 1876

http://species-id.net/wiki/Peltonyxa

Figs 1 , 12 ; Map 13

Peltonyxa Reitter, E. 1876: 46.

##### Type species.

*Peltonyxa deyrollei* Reitter, 1876 [by monotypy]

Léveillé, A. 1910: 24. Kolibáč, J. 2005: 76 (redescription). Kolibáč, J. 2006: 111 (phylogeny). Kolibáč, J. 2010: 35 (key). Matthews, E. G. 1992: 3

*Floricateres* Crowson, 1970 [Type species: *Floricateres pusillus* Crowson, 1970]

Crowson, R. A. 1970: 10. Kolibáč, J. 2005: 76 (syn. *Floricateres*)

##### Description.

Body size: about 3.5 mm. Body shape flat. Gular sutures wide, subparallel. Frontoclypeal suture broadly emarginate. Frons: longitudinal groove or depression absent. Cranium ventrally: tufts of long setae at sides absent. Submentum of males: ctenidium present. Antennal groove present. Eyes: size moderate. Eyes number: two. Epicranial acumination moderate. Lacinial hooks absent. Galea: shape very small. Galea: ciliate setae absent. Mediostipes-Lacinia not fused. Palpifer: outer edge even. Mandibular apical teeth number: two, horizontally situated. Mola absent. Penicillus (at base) absent. Pubescence above mola or cutting edge absent. Ventral furrow absent. Basal notch moderate. Labrum-Cranium not fused. Epipharyngial sclerite absent. Lateral tormal process: projection projection reduced or absent (*Promanus*). Ligula: ciliate setae absent. Ligula membranous, not retroflexed, deeply emarginate. Hypopharyngeal sclerite H-shaped. Antenna 9-segmented. Antennal club weakly asymmetrical, sensorial fields absent. Front coxal cavities externally open, internally open. Pronotum transverse. Prepectus present. Middle coxal cavities open. Elytra: long hairs absent. Epipleuron moderate. Elytral interlocking mechanism absent, carinae conspicuous or reduced. Elytral punctation regular, scales absent. Wing: radial cell moved down, often small, wedge cell absent, cross vein MP3-4 absent, cross vein AA1+2-3+4 absent. Front tibiae: spines along side moderate. Hooked spur present. Claws: denticle absent. Spiculum gastrale present. Tegmen composed of three parts. Coxitae divided.

##### Biology.

*Peltonyxa pusillus* was collected on the flowers of *Bursaria* (Crowson 1970). South Australian species occur in the sclerophyll and Eremaean zones (Matthews 1992).

##### Distribution.

Australia: South Australia, New South Wales, Victoria.

##### Species:

*Peltonyxa australis* Blackburn, 1891; S Australia (AL)

Léveillé, A. 1910: 24

*Peltonyxa deyrollei* Reitter, 1876; Australia (AL)

Léveillé, A. 1910: 24. Kolibáč, J. 2005: 76. Reitter, E. 1876: 46

*Peltonyxa invalida* Blackburn, 1902; Australia („Nov. Gall. mer.”) (AL)

Léveillé, A. 1910: 24

*Peltonyxa pubescens* Blackburn, 1891; Australia: Victoria (AL)

Léveillé, A. 1910: 24

*Peltonyxa pusillus* Crowson, 1970; Australia: NSW (RAC)

Crowson, R. A. 1970: 11 (*Floricateres*)

#### Genus Promanus

Sharp, 1877

http://species-id.net/wiki/Promanus

Fig. 12 ; Map 13

Promanus Sharp, D. 1877: 267.

##### Type species.

*Promanus depressus* Sharp, 1877 [by monotypy]

Léveillé, A. 1910: 29. Crowson, R. A. 1964a: 298. Kolibáč, J. 2005: 78 (redescription). Kolibáč, J. 2006: 111 (phylogeny). Kolibáč, J. 2010: 35 (key). Kolibáč, J. et al. 2010: 36

##### Description.

Body size: 6.8–8.8 mm. Body shape flat. Gular sutures wide, convergent at apex. Frontoclypeal suture broadly emarginate. Frons: longitudinal groove or depression absent. Cranium ventrally: tufts of long setae at sides absent. Submentum: ctenidium absent. Antennal groove present. Eyes: size large, lateral. Eyes number: two. Epicranial acumination absent. Lacinial hooks: two. Galea: shape sub-clavate. Galea: ciliate setae absent. Mediostipes-Lacinia fused together. Palpifer: outer edge even. Mandibular apical teeth number: two, horizontally situated. Mola absent. Penicillus (at base) present (fine, often membranous). Pubescence above mola or cutting edge absent. Ventral furrow absent. Basal notch shallow or absent. Labrum-Cranium not fused. Epipharyngial sclerite absent. Lateral tormal process: projection projection reduced or absent (*Promanus*). Ligula: ciliate setae absent. Ligula membranous, not retroflexed, deeply emarginate. Hypopharyngeal sclerite H-shaped. Antenna 11-segmented. Antennal club weakly asymmetrical, sensorial fields absent. Front coxal cavities externally open, internally open. Pronotum transverse. Prepectus present. Middle coxal cavities open. Elytra: long hairs absent. Epipleuron moderate. Elytral interlocking mechanism absent, carinae reduced. Elytral punctation regular, scales absent. Wing: radial cell moved down, often small, wedge cell absent, cross vein MP3-4 absent, cross vein AA1+2-3+4 absent. Front tibiae: spines along side moderate. Hooked spur present. Claws: denticle absent. Parasternites number along ventrites III–VII: two. Coxitae divided.

Larva: Frontal arms V-shaped. Epicranial stem absent. Endocarina present. Gular sutures conspicuous, convergent. Gula: anterior apodemes absent. Paragular sclerites absent. Hypostomal rods absent. Stemmata number: two. Mandibular apical teeth number: two, horizontally situated. Lacinia mandibulae plumose. Mola absent. Maxillary palpi 3-segmented. Pedunculate seta present. Mala: bidentate protrusion absent. Cardo-Stipes not fused. Cardo: size nearly as large as stipes. Labial palpi 2-segmented. Prementum in single part, anterior margin even. Antennal joints 1 and 2 elongate. Sensory appendix very small. Abdominal segment IX transversely divided. Tergite IX flat. Urogomphi present, hooked; median process present.

##### Biology.

The adults and larvae are predatory. Crowson (1964a) found insect fragments in the gut of both stages.

##### Distribution.

New Zealand.

##### Species:

*Promanus auripilis* Broun, 1893; New Zealand (AL)

Léveillé, A. 1910: 29. Kolibáč, J. et al. 2010: 36 (redescription)

*Promanus depressus* Sharp, 1877; New Zealand (AL)

Léveillé, A. 1910: 29. Crowson, R. A. 1964a: 298 (larva). Kolibáč, J. 2005: 78 (redescription). Kolibáč, J. et al. 2010: 36

*Promanus subcostatus* Broun, 1909; New Zealand (AL)

Léveillé, A. 1910: 29. Kolibáč, J. et al. 2010: 36 (redescription)

#### † Genus Promanodes

Kolibáč, Schmied, Wappler & Kubisz, 2010

http://species-id.net/wiki/Promanodes

Map 13

Promanodes Kolibáč, J. et al. 2010: 31.

##### Type species.

*Promanodes serafini* Kolibáč, Schmied, Wappler & Kubisz, 2010 [by monotypy and author’s designation]

Schmied, H. et al. 2009: 105 (distribution). Kolibáč, J. 2011: 58

##### Description.

Body size: 3.1–5.1 mm. Procoxal cavities nearly closed; maxillary palps with securiform terminal joint; extraordinarily elongate terminal segment of labial palps (distinctly longer or as long as two preceding segments together); slender and elongate tarsi in all pair of legs (approximately as long as tibiae); tibiae without “hooked” apical spine; procoxal cavities nearly closed or maybe perfectly closed in the new species; six visible abdominal ventrites; 10-segmented antennae; flat body; antennal club large and loose, 3-segmented; mesocoxae weakly transverse; elytra with distinct carinae. (Genus diagnosis after Kolibáč 2011.)

The new genus is very similar to the recent *Promanus*, the both genera share deep and incurvate frontoclypeal suture, distinctly elevated eyes, weakly or no way projecting anterior pronotal corners, elytra with weak or inconspicuous carinae widest at about 2/3 of length, radial cell obliquely situated, dorsal body surface sparsely pubescent or nearly bare, femora conspicuously clavate, very small trochanters, and especially abdomen with six visible ventrites. Body length 3–5 mm. (According to Kolibáč et al. 2010.)

##### Distribution.

(Map 13.) Baltic amber: Poland, East Baltic coast(?); Tertiary: Eocene.

##### Species:

† *Promanodes alleni* Kolibáč, 2011; Baltic amber: East Baltic coast(?); Tertiary: Eocene (JK)

Kolibáč, J. 2011: 59

† *Promanodes serafini* Kolibáč, Schmied, Wappler & Kubisz, 2010; Baltic amber: Poland; Tertiary: Eocene (varA)

Schmied, H. et al. 2009: 105 (distribution). Kolibáč, J. et al. 2010: 36. Kolibáč, J. 2011: 59

#### Genus Trichocateres

Kolibáč, 2010

http://species-id.net/wiki/Trichocateres

Fig. 12 ; Map 13

Trichocateres Kolibáč, J. 2010: 35.

##### Type species.

*Trichocateres fasciculifer* Kolibáč, 2010 [by monotypy and author’s designation]

##### Description.

Body size: 5.2–5.5 mm. With general characteristics of the tribe Lophocaterini (body oval, frontoclypeal suture deeply arcuate, antennal club weakly asymmetrical, mandibular mola present, base of mandible with membranous appendage/penicillus, prostheca composed of tuft of setae, lacinia with spines). It is most closely related to *Lophocateres*, *Indopeltis* and *Grynocharis* (mandibular mola present, elytral carinae well-developed).

Similarities of *Trichocateres* with *Lophocateres* and *Indopeltis*: the mandible with membranous penicillus and distinct prostheca; the wing venation with cross-veins MP3-4 and AA1+2-3+4 absent; and the metendosternite with robust stalk and widely separated anterior tendons. It resembles *Indopeltis* in aedeagus with projecting phallobasic apodeme, eyes similarly shaped, relatively large and situated dorsally, and lateral edge of pronotum undulating, whereas *Lophocateres* parallels include labrum with tormal processes branched at base and maxilla with mediostipes not fused with lacinia. Excluding the characters mentioned, *Trichocateres* differs from all three abovementioned genera chiefly in tibial spur pattern 1-1-1, two sharp grooves in prosternal process and tufts of long hairs on elytra and pronotum. (Genus diagnosis after Kolibáč 2010.)

##### Biology.

The circumstances of collection are not exactly known; the specimens were knocked down from branches or fallen timber. Remnants of insect cuticle were found in the gut of the Assam specimen, the remains of an insect larva in the gut of the Laos specimen.

##### Distribution.

India: Assam, northern Laos.

##### Species:

*Trichocateres fasciculifer* Kolibáč, 2010; NE India: Assam, N Laos (JK)

Kolibáč, J. 2010: 36

#### Species *incertae sedis*

***Calitys africana* Boheman, 1848** (Cucujoidea?)

Léveillé, A. 1910: 24

**Note:** I studied only one non-type specimen determined as *Calitys africana* in the Museé d’Histoire Naturelle in Geneva. The specimen does not belong in Cleroidea

***Latolaeva brasilica* Perty, 1830** (Cucujoidea?)

Reitter, E. 1876: 51

**Note.** I have not studied any specimens of the species. The autochthonous distribution of *Latolaeva* or *Ancyrona* in South America is unprobable, although introduction is possible. Species of the two genera could be misidentified for an autochthonous or introduced Australian *Neaspis* or *Peltonyxa* (see “Distribution” for *Neaspis*)

***Ostoma australis* Boisduval, 1835**

Léveillé, A. 1910: 31

***Ostoma higonia* Lewis, 1894**

Léveillé, A. 1910: 31

***Ostoma pubescens* Escherich, 1822**

Léveillé, A. 1910: 32

#### Taxa occassionally or temporarily classified within Trogossitidae

##### Coleoptera *incertae sedis*

###### † Genus Anhuistoma

Lin, 1985

http://species-id.net/wiki/Anhuistoma

Trogossitidae Lin, Q.-B. 1985: 309 ( ).

####### Type species.

*Anhuistoma hyla* Lin, 1985 [designated by author and by monotypy]

Kolibáč, J. & Huang, D.-Y. 2008: 136 (Coleoptera *incertae sedis*)

####### Remarks.

This beetle was originally described in Trogossitidae and later removed from the superfamily as Coleoptera *incertae sedis*.

####### Original description of the genus.

“*A broadly elliptic beetle of small size; pronotum broadly hemiorbicular* (hemispherical?); *elytra short and broad, ornamented with many longitudinal striae; legs short, three pairs of coxae clearly separated, fore-coxa transverse, mid-coxa rounded, both posterior coxae transverse and connected with each other; abdomen with 5 visible sternites.*” (Lin 1985: 309).

####### Original description of the species.

“*The body of a small beetle with head and legs missing, 3.5 mm long and 2.3 mm wide. Body broadly elliptic. Pronotum in hemiorbicular* (hemispherical?) *form, slightly broader than long; anterior margin of pronotum concave, posterior margin of pronotum as wide as the anterior. Anterior coxa transverse and disjointed. Mid-thorax slightly smaller than metathorax. Both mid-coxae rounded and disjointed from one another. Posterior coxae transversely connected. Elytra much broader at base, gradually narrowing toward apex, rounded at apical angle; surface covered with several longitudinal striae. Abdomen with 5 visible sternites.*” (Lin 1985: 309).

####### Species:

† *Anhuistoma hyla* Lin, 1985; China: Anhui province; Mesozoic: Lower-Middle Jurassic

Lin, Q.-B. 1985: 309 (Trogossitidae). Kolibáč, J. 2006: 136 (Trogossitidae *incertae sedis*). Kolibáč, J. & Huang, D.-Y. 2008: 137 (Coleoptera *incertae sedis*)

###### † Genus Peltocoleops

Ponomarenko, 1990

http://species-id.net/wiki/Peltocoleops

Cleroidea Ponomarenko, A. G. 1990: 73 ( ).

####### Type species.

*Peltocoleops onokhojensis* Ponomarenko, 1990 (by monotypy and original designation)

Kolibáč, J. 2006: 129 (Lophocaterini). Ponomarenko, A. G. & Kireichuk, A. G. 2005–2008: http://www.zin.ru/animalia/Coleoptera/rus/paleosy2.htm (Peltidae). Schmied, H. et al. 2009: 25 (Trogossitidae: Peltinae)

####### Remarks.

The genus was established in Cleroidea, with no indication of an exact family classification (Ponomarenko 1990). Later, Ponomarenko and Kireichuk (2005–2008) listed it in Peltidae. I classified *Peltocoleops onokhojensis* within Lophocaterini but this opinion has to be revised here because it was based on mistranslation of antennal characteristics. The symmetrical club (not “weakly asymmetrical”), widely separated mesocoxae and obliquely situated metacoxae (Ponomarenko 1990: p. 73, 74; fig. 70b) exclude classification of the beetle within Cleroidea. It is therefore listed as Coleoptera *incertae sedis* herein.

####### Original diagnosis of the genus

(translation from Russian). “*Medium sized, oval, flat beetle. Head short, gular sutures divergent backwards* [sic], *antennae long with indistinct symmetrical club. Pronotum wide and short, its base not narrower than elytra, front coxae large, transverse, not projecting, with exposed trochantin, prosternal process extending behind coxae, very slightly widened. Middle coxal cavities closed by mesepimeron. Middle coxal cavities large, rounded, with exposed trochantin. Metathorax transverse, weakly convex along join of legs* [sic], *hind trochantins* [sic] *oblique. Abdomen with 5 visible sternites, their bases bordered* (?). *Legs relatively long, outer side of middle tibiae with row of firm spines.*”

####### Original description of the species

(translation from Russian). “*Head twice shorter than wide, tempora shorter than eyes, gula shorter than wide. Antennomere 6 reaches backwards base* [sic] *of pronotum. Pronotum evenly rounded anteriorly, 2.5 times shorter than wide, anterior margin straight, about twice narrower than basal margin. Prothorax short, shorter than front coxae, prosternal process distinctly runs behind coxae. Middle coxae relatively widely separated. Metathorax about 2.5 times shorter than wide along its basal margin. Hind coxa is longest along middle of body and 3.5 times shorter than wide* [sic], *coxae distinctly shortened at lateral end. The first and the last abdominal sternites conspicuously longer than others. Elytra widest at basal portion, glabrous. Middle femora scarcely project out of body outline, tibiae widened, with longitudinal sulcus with row of firm spines (or setae) along outer side, tibiae longer than femora.*” “*Length 7.2, width 4.1, length of elytra 5.2 mm.*”

####### Distribution.

Russia: Transbaikalia, Onokhovo, Onokhovsky Graben, Baleysky distr., Chitinskaya obl.; Mesozoic: Early Cretaceous, Lower Neocomian, Leskovskaya Beds.

####### Species:

† *Peltocoleops onokhojensis* Ponomarenko, 1990; Russia: Transbaikalia; Early Cretaceous, Lower Neocomian (varA)

Ponomarenko, A. G. 1990: 73. Ponomarenko, A. G. & Kireichuk, A. G. 2005–2008: http://www.zin.ru/animalia/Coleoptera/rus/paleosy2.htm. Schmied, H. et al. 2009: 26

#### Superfamily Cucujoidea *incertae sedis*

##### † Subfamily Meligethiellinae

Kireichuk & Ponomarenko, 1990

###### Remarks.

Because Meligethiellinae is excluded from Cleroidea herein, the synonymization of the subfamily with Peltinae: Thymalini is no longer valid. Meligethiellinae should be classified within traditional Cucujoidea and it is probably related to the extant Nitidulidae. However, this issue lies beyond the scope of this communication and should be addressed by those working on Cucujoidea.

##### † Genus Meligethiella

Medvedev, 1969

http://species-id.net/wiki/Meligethiella

Nitidulidae Medvedev, L. N. 1969: 119 ( ).

###### Type species.

*Meligethiella soroniiformis* Medvedev, 1969 (by monotypy and original designation)

Kireichuk, A. G. & Ponomarenko, A. G. 1990: 81 (Peltidae: Meligethiellinae). Kolibáč, J. 2006: 126 (Thymalini). Ponomarenko, A. G. & Kireichuk, A. G. 2005–2008: http://www.zin.ru/animalia/Coleoptera/rus/paleosy2.htm. Schmied, H. et al. 2009: 25

###### Remarks.

The genus was originally described in Nitidulidae (Medvedev 1969) and latter shifted to Peltidae by Kireichuk and Ponomarenko (1990) in the new extinct subfamily Meligethiellinae along with the genera *Juralithinus* and *Ostomalynus*. I accepted the classification within Trogossitidae: Peltinae. However, I synonymized Meligethiellinae and moved all three genera into the extant Thymalini (Kolibáč 2006). After more careful examination of their descriptions and original illustration, I found the classification of *Meligethiella* and *Ostomalynus* within Trogossitidae or even Cleroidea hardly possible while *Juralithinus* characters are fully compatible with the definition of Peltinae. The most important characters that exclude *Meligethiella* from Trogossitidae are (1) widely separated mesocoxae, especially in combination with (2) irregularly punctate elytra and (3) groove for prosternal process in mesosternum.

###### Original description of the genus

(translation from Russian). “*Diagnosis: Body broadly oval, head not distinctly retracted into pronotum, pronotum just about narrower than elytra, 3–4 times shorter than elytra, middle coxae widely separated; mesothorax with groove for apex of prosternal process; elytra perfectly cover abdominal apex, smooth or with irregular punctation; femoral lines in metathorax* [paracoxal sutures?] *mostly well-developed; first visible abdominal sternite longer than following one.* [Measurements 3.7–4.7 mm.]”

###### Distribution.

Russia: Bouriatskaya Autonomnaya Respublika; W Transbaikalia – Chitinskaya oblast; Kazakhstan: Chimkentskaya oblast, Baissa. Mesozoic: Late Jurassic: Karabastayskaya formation; Lower Cretaceous: Turginskaya formation; middle Neocomian: Zazinskaya formation.

###### Species:

† *Meligethiella glabra* Kireichuk & Ponomarenko, 1990; Russia: Transbaikalia, Chitinskaya obl.; Lower Cretaceous, Turginskaya formation (varA)

Kireichuk, A. G. & Ponomarenko, A. G. 1990: 82. Ponomarenko, A. G. & Kireichuk, A. G. 2005–2008: http://www.zin.ru/animalia/Coleoptera/rus/paleosy2.htm. Schmied, H. et al. 2009: 25

† *Meligethiella kovalevi* Kireichuk & Ponomarenko, 1990; Kazakhstan: Chimkentskaya obl.; Late Jurassic: Karabastayskaya formation (varA)

Kireichuk, A. G. & Ponomarenko, A. G. 1990: 81. Ponomarenko, A. G. & Kireichuk, A. G. 2005–2008: http://www.zin.ru/animalia/Coleoptera/rus/paleosy2.htm. Schmied, H. et al. 2009: 25

† *Meligethiella soroniiformis* Medvedev, 1969; Russia: Bouriatskaya Autonom. Rep., Baissa; Lower Cretaceous: Zazinskaya formation (varA)

Kireichuk, A. G. & Ponomarenko, A. G. 1990: 81. Medvedev, L. N. 1969: 120. Ponomarenko, A. G. & Kireichuk, A. G. 2005–2008: http://www.zin.ru/animalia/Coleoptera/rus/paleosy2.htm. Schmied, H. et al. 2009: 25

##### † Genus Ostomalynus

Kireichuk & Ponomarenko, 1990

http://species-id.net/wiki/Ostomalynus

Peltidae Kireichuk, A. G. & Ponomarenko, A. G. 1990: 82 ( : Meligethiellinae).

###### Type species.

*Ostomalynus ovalis* Kireichuk & Ponomarenko, 1990 (by monotypy and original designation)

Kolibáč, J. 2006: 126 (Thymalini). Ponomarenko, A. G. & Kireichuk, A. G. 2005–2008: http://www.zin.ru/animalia/Coleoptera/rus/paleosy2.htm. Schmied, H. et al. 2009: 25

###### Remarks.

The genus was described in Meligethiellinae by Kireichuk & Ponomarenko (1990) and latter shifted to Thymalini by (Kolibáč 2006, Schmied et al. 2009). It is apparently related to *Meligethiella*, so both genera must be removed together from Trogossitidae and Cleroidea.

###### Original description.

“*Body oviform, strongly narrowed posteriorly. Pronotum short, five times shorter than elytra, prothorax very short, nearly lenticular, with strong longitudinal keel along centre, middle coxae widely separated, anterior part of mesothorax with groove for apex of prosternal process, elytra reaching behind end of abdomen, dorsally with thin lines beneath them longitudinal rows of punctures can be visible.* [Measurements: length 5.3, width 2.8 mm.]”

###### Distribution.

Russia: Transbaikalia, Chitinskaya oblast, Nerchinsko-Zavodskoy district, Pavlovka village. Mesozoic: Early Cretaceous, Lower Neocomian, Gidarinyskaya formation.

###### Species:

† *Ostomalynus ovalis* Kireichuk & Ponomarenko, 1990; Russia: Transbaikalia; Lower Cretaceous, Gidarinyskaya formation

Kireichuk, A. G. & Ponomarenko, A. G. 1990: 83. Ponomarenko, A. G. & Kireichuk, A. G. 2005–2008: http://www.zin.ru/animalia/Coleoptera/rus/paleosy2.htm. Schmied, H. et al. 2009: 25

#### Superfamily Cucujoidea *incertae sedis*

##### † Genus Palaeoendomychus

Zhang, 1992

http://species-id.net/wiki/Palaeoendomychus

Endomychidae Zhang, J.-F. 1992: 337 ( ).
Palaeoendomychus gymnus [Type species: Zhang, 1992]
Cucujoidea Kolibáč, J. & Huang, D.-Y. 2008: 139 ( *incertae sedis*). Ponomarenko, A. G. & Kireichuk A. G. (2005–2008): http://www.zin.ru/animalia/Coleoptera/rus/paleosy2.htm (Trogossitidae). Schmied, H. et al. 2009: 26.

###### Remarks.

The author (Zhang 1992: 337) compares the genus with pantropical *Stenotarsus* Perty, 1832 which contains about 250 recent species that are highly variable in body shape. A combination of characters similar to those of *Palaeoendomychus* may be found, for example, in Endomychidae and Nitidulidae (Kolibáč and Huang 2008).

###### Original description.

“*Body minute and compact, oval, hairless. Head deeply sunk into pronotum. Eyes rather large, widely separated, slightly prominent. Antennae short, flagellum cylindrical. Pronotum transverse, short* (Chin.: raised at centre), *without membrane* [sic] *in front, but with lateral sides* (Chin. “edges”) *broadly flattened, posterior angles prominent. Scutellum small, narrowly triangular, distinctly longer than broad. Elytra wide, dehiscent, as wide at base as pronotum, and both closely connected to one another, humeral angles rounded, terminal angles prominent, surface with striae. Legs normal, with tarsi short and narrow* (Chin. 3 tarsomeres visible), *first and second tarsal segments triangular, each about as long as wide, third cylindrical, noticeably longer than wide.*” (Zhang 1992: 334, 337.) After Kolibáč and Huang (2008).

Note: The Chinese and English texts are somewhat different from one another. Important items from the Chinese are translated in parentheses.

###### Species:

† *Palaeoendomychus gymnus* Zhang, 1992; China: Shandong; Mesozoic: Lower Cretaceous

Zhang, J.-F. 1992: 337 (Endomychidae). Kolibáč, J. 2006: 136 (Trogossitidae *incertae sedis*). Kolibáč, J. & Huang, D.-Y. 2008: 139 (Cucujoidea *incertae sedis*). Ponomarenko, A. G. & Kireichuk A. G. (2005–2008): http://www.zin.ru/animalia/Coleoptera/rus/paleosy2.htm (Trogossitidae). Schmied, H. et al. 2009: 26

#### Family Salpingidae

##### Genus *Paralindria* Olliff, 1883

[**Type species**: *Paralindria bipartita* Olliff, 1883]

Olliff, S. 1883b: 57 (“... *be placed between* [Alindria] *and* Melambia.”). Reitter, E. 1890: 264 [“*Paralindria* Oliff, 1883 = *Serrotibia* Reitt. (Stett. Ztg. 1877, 339)]

*Paralindria bipartita* Olliff, 1883; Ecuador

Reitter, E. 1890: 264 [“*Paralindria bipartita* Oliff. = *Serrotibia bicolor* Reitt., 1. c. pag. 341”]

**Note.** The synonymy is widely accepted and the genus *Serrotibia* is classified within Salpingidae at present.

#### Family Chrysomelidae

##### Genus *Peltoschema* Reitter, 1880

[**Type species**: *Peltoschema filicorne* Reitter, 1880]

Reitter, E. 1880: 4 (Trogossitidae). Léveillé, A. 1910: 28 (Trogossitidae)

**Note.** The classification of the genus within Chrysomelidae has been well known for a long time, e.g. Daccordi (2003a, b), Daccordi and De Little (2003). Species of the genus are distributed in Australia.

#### Family Derodontidae

##### Genus *Peltastica* Mannerheim, 1852

[**Type species:** *Peltastica tuberculata* Mannerheim, 1852]

Léveillé, A. 1910: 28 (Trogossitidae). Reitter, E. 1876: 60 (Trogossitidae)

*Peltastica tuberculata* Mannerheim, 1852; Alaska (AL)

Léveillé, A. 1910: 28 (Trogossitidae). Reitter, E. 1876: 60 (Trogossitidae)

*Peltastica amurensis* Reitter, 1879; Siberia (AL)

Léveillé, A. 1910: 28 (Trogossitidae)

*Peltastica reitteri* Lewis, 1884; Japan (AL)

Léveillé, A. 1910: 28 (Trogossitidae)

**Note.** The classification of the genus within Derodontidae has been well known for a long time.

#### Family Helotidae

*Helota* MacLeay, 1825; eastern Asia

Reitter, E. 1876: 5 (Trogositidae: subfamilia Helotidae; *sic*!)

#### Family Monotomidae

? *Nemozoma nigripenne* Reitter, 1876; Colombia (varA)

Léveillé, A. 1910: 6 (*Nemosoma* (subgen. *Monesoma*)). Reitter, E. 1876: 14 (*Nemozoma nigripennis*)

syn. *Thione championi* Sharp, 1899?

Lepesme, P. & Paulian, R. 1944: 138. According to their notes, they studied the holotype of *Nemosoma (Monesoma) nigripenne* Lév. [sic] and consider it “[...] *est d’apres le type, un Cucujidae et est identique a* Thione Championi *Sharp*”. The matter may be unfamiliar to monotomid workers as I have found no remarks on this synonymy in the literature.

##### Genus *Shoguna* Lewis, 1889

**Note.** Albert Winkler listed the genus among Trogossitidae in his catalogue (1924–1932) and probably considered it a relative of *Nemozoma* (Winkler 1927). It is classified within Monotomidae at present.

#### Family Synteliidae

##### Genus *Syntelia* Westwood, 1864

[**Type species:** *Syntelia indica* Westwood, 1864] (genus originally described in Trogossitidae)

Reitter, E. 1876: 23 (Trogossitidae). Westwood, J. O. 1864: 11. (Trogossitidae)

*Syntelia davidis* Fairmaire, 1889; China: Sichuan

*Syntelia histeroides* Lewis, 1882; Russia: Far East, Japan

*Syntelia indica* Westwood, 1864; Nepal, Northeast India

Reitter, E. 1876: 23 (Trogossitidae). Westwood, J. O. 1864: 11

*Syntelia mexicana* Westwood, 1864; Mexico: Oaxaca

Reitter, E. 1876: 23 (Trogossitidae). Westwood, J. O. 1864: 11

*Syntelia westwoodi* Salle, 1873; Mexico

Reitter, E. 1876: 23 (Trogossitidae)

#### Family Zopheridae

##### Genus *Holopleuridia* Reitter, 1876

[**Type species:** *Holopleuridia maculosa* Reitter, 1876]

Kolibáč, J. 2005: 87 (transferred to Tenebrionoidea). Léveillé, A. 1910: 27 (Trogossitidae). Reitter, E. 1876: 56 (Trogossitidae)

*Holopleuridia maculosa* Reitter, 1876; “La Luzera” (Colombia?)

Reitter, E. 1876: 56 (Trogossitidae)

##### *Nematidium* Erichson, 1845

[**Type species**: unknown]

*Nematidium tenuissima* (Reitter, 1876); Colombia

Reitter, E. 1876: 16 (*sub* Trogossitidae: *Filumis* Reitter, 1876)

## Phylogeny of the family Trogossitidae

Recently, several important studies have centred upon the phylogeny of Cucujiformia, including Cleroidea and Trogossitidae.

Beutel and Pollock (2000) analyzed 20 larval head characters in 22 taxa (8 cleroid, 10 cucujiform, 4 outgroup) and advocated the monophyly of Cleroidea, but pointed out the paraphyly of traditional Cucujoidea. Trogossitidae were represented by *Calitys* and *Temnoscheila*, which genera were found in a sister relationship (*Calitys* was perhaps meant by the authors to be a representative of Peltinae). However, the inclusion of *Phloiophilus* into the trogossitids, as is presented herein, would be considered paraphyletic (but not polyphyletic) according to this study.

Hunt et al. (2007) used an extensive molecular data set of dozens Coleoptera specimens. His Cleroidea are also monophyletic but with the inclusion of Byturidae/Biphyllidae as a sister group of *Phloiophilus*. Trogossitidae are paraphyletic in the final tree: Trogossitinae + ((Lophocaterinae + Peltinae) + rest of Cleroidea). On the other hand, the most parsimonious tree of all 1880 studied beetle genera based on two mitochondrial genes shows the following phylogeny: [(*Diplocoelus* + *Biphyllus*) + (*Byturus* + (*Phloiophilus* + *Priasilpha*))] + [((*Kolibacia* + *Temnoscheila* + *Nemozoma* + *Tenebroides*) + (*Ancyrona* + *Lophocateres* + *Grynocharis*)) + (*Peltis grossa* + *Peltis ferrugineum*)] + (Cleridae + Melyridae *sensu lato*). This means that Trogossitidae (minus *Phloiophilus*) are perfectly monophyletic in the tree. It is also notable that Lophocaterinae are a sister group of Trogossinae there. (Note: *Leperina* is used for *Kolibacia tibialis*, *Trogossita* for *Temnoscheila*, *Ostoma* for *Peltis ferrugineum* in the mentioned paper.)

The most recent system of Trogossitidae is based on my morphological studies of 2005 (adults) and 2006 (larvae). The most important result is a confirmation of both Crowson’s ideas on the relationship of *Calitys* with Trogossitinae and *Protopeltis* with the *Rentonium*-group. On the other hand, his opinion about the relationship between the lophocaterins and the trogossitins is called in question. However, it is correct to say that larval characters actually connect the two groups while the majority of adult characters tend to defeat them in favour of a sister relationship of the lophocaterins and trogossitins. Both major clades were analyzed separately under equal weights and then with the use of successive reweighting (Kolibáč 2006).

I employed 31 traditional morphological characters (16 adult, 15 larval) for 15 taxa (8 cleroid, 6 cucujoid, 1 derodontoid) in the analysis of 2008. Five cucujoid families (minus Phloeostichidae: *Hymaea*) were found to be monophyletic as well as Cleroidea. The group of cucujoid families (Cerylonidae, Coccinelidae, Endomychidae, Cucujidae, Byturidae, Phloeostichidae) were found to be paraphyletic with regard to the monophyletic Cleroidea. Trogossitidae were also found to be paraphyletic in this analysis. However, *Phloiophilus* was placed within a branch composed of trogossitid representatives, probably as a sister group of *Thymalus*. In the following analysis, *Phloiophilus* was analysed together with 43 trogossitid genera (88 characters of which 56 were adult and 32 larval). A strict consensus tree of 48 equally parsimonious trees indicates a possible relation of *Phloiophilus* with Peltinae and justifies independent subfamilial status for Lophocaterinae (the tree is reproduced herein, Fig. 26). It must be pointed out that 32 of 48 trees supported a sister relationship between the lophocaterins and the peltins. Only 16 trees supported a relationship between the lophocaterins and the trogossitins. This is perfectly consistent with the results of Hunt et al. (2007) and Kolibáč (2006).

The most extensive modern paper on the beetle phylogeny based on morphological data is that by Lawrence et al. (2011). The authors used more than 500 characters and analyzed 366 genera of 165 beetle families. The final resulting tree is, in the section relevant to us, far different from that by Hunt et al. (2007) and it is beyond the scope of this contribution to analyze differences at superfamilial level. Trogossitidae are found to be paraphyletic (*Acalanthis* + *Temnoscheila*, *Eronyxa* + *Grynoma* are placed in separate branches), even polyphyletic because *Thymalus* is placed in a cluster together with *Lamingtonium* and the nitidulid families, while *Rentonellum* as a sister group of *Smicrips* together with *Laemophloeus* and *Propalticus* lie in a very distant part of the cladogram. *Phloiophilus* was not studied in the paper.

Bocáková et al. (2011) used *Peltis ferrugineum*, *Grynocharis oblonga* and *Temnoscheila japonica* among outgroups in their molecular phylogeny of Melyridae *sensu lato.* *Peltis* and *Grynocharis* again form a sister group while the position of *Temnoscheila* is paraphyletic with regard to the rest of Cleroidea.

A work by Gunter et al. (2013) focuses on the molecular phylogeny of Cleridae. However, a relatively large set of trogossitid representatives were also studied. The resulting tree is very interesting: *Phloiophilus* is included in the trogossitine cluster between *Larinotus*, *Leperina* and *Temnoscheila* while *Rentonellum*, *Peltis*, *Grynocharis*, *Ancyrona* and *Neaspis* form a second cluster with a sister relationship to the rest of Cleroidea (similar to Hunt et al. 2007).

The most updated studies are those by the Tree of Life team and R. A. B. Leschen and co-authors, unpublished as yet. Their preliminary, mutually different, results were presented in the XXIVth International Congress of Entomology in Daegu (McKenna et al. 2012, Leschen et al. 2012).

Although the outcomes of the works above differ in details, major ideas may be summarized as follows:

1) The superfamily Cleroidea is a monophyletic group.
2) A part of the traditional Cucujoidea is probably paraphyletic with regard to Cleroidea. Cleroidea will probably, therefore, be extended to include several other “cucujoid” families.
3) Lophocaterinae probably constitutes a sister group to Peltinae.
4) Trogossitinae, as a basal group, may form a sister taxon to the major bulk of Cleroidea (although none of the molecular studies included the Metaxinidae-Chaetosomatidae-Thanerocleridae cluster, which might be in sister relationship with the trogossitines). This thesis, however, can hardly be justified by just the morphological evidence. If the subfamily Trogossitinae is actually confirmed paraphyletic, it should be classified at family rank again, as well as Peltidae composed of Peltinae and Lophocaterinae.
5) The exact position of *Phloiophilus* is uncertain; it is a basal group of Cleroidea, probably related to Trogossitidae *sensu lato.*

**Figure 13.**
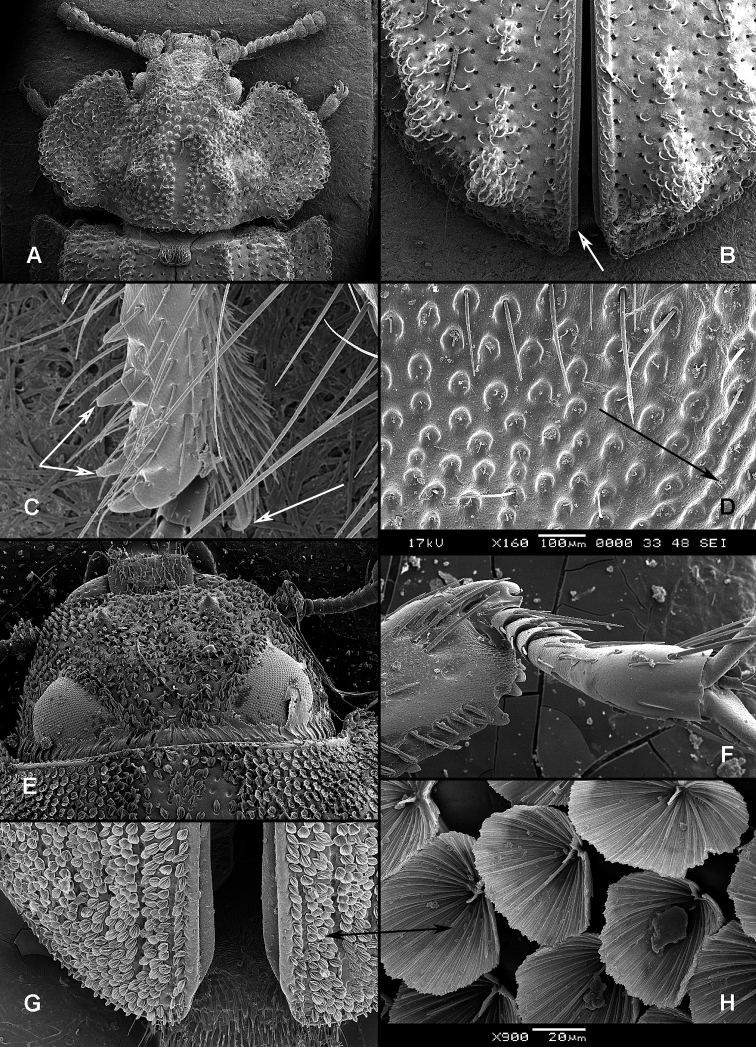
**A–B** *Calitys scabra* (**A** head and pronotum **B** elytral apex with interlocking mechanism) **C–D** *Acalanthis quadrisignata* (**C** apex of protibia with hooked spur and lateral spines **D** sculpture of frons with punctures conjoined into wrinkles) **E–H** *Gymnocheilis* sp., Cameroon (**E** head **F** protibia **G** elytral apex **H** detail of scales).

**Figure 14.**
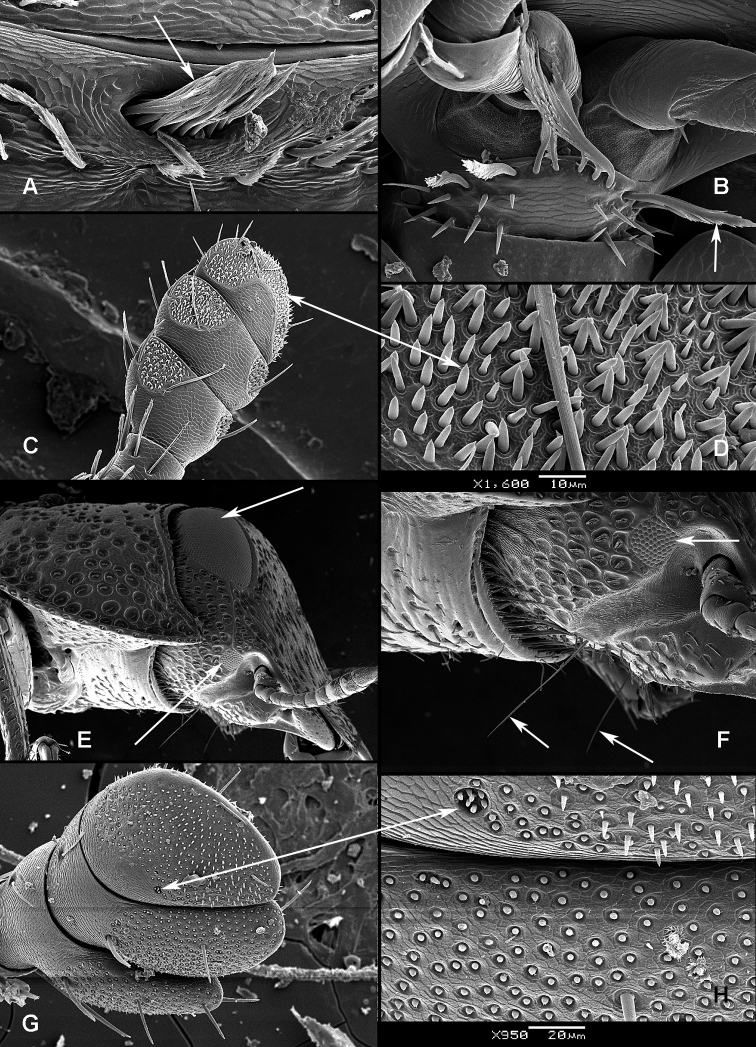
**A–F** *Anacypta* sp., Assam (**A** ctenidium **B** labium with ciliate setae **C** antennal club with sensorial fields **D** detail of sensillae **E** head laterally with divided eye **F** detail of sensorial setae and ventral eye) **G–H** *Gymnocheilis* sp., Cameroon (**G** antennal club **H** detail of sensillae).

**Figure 15.**
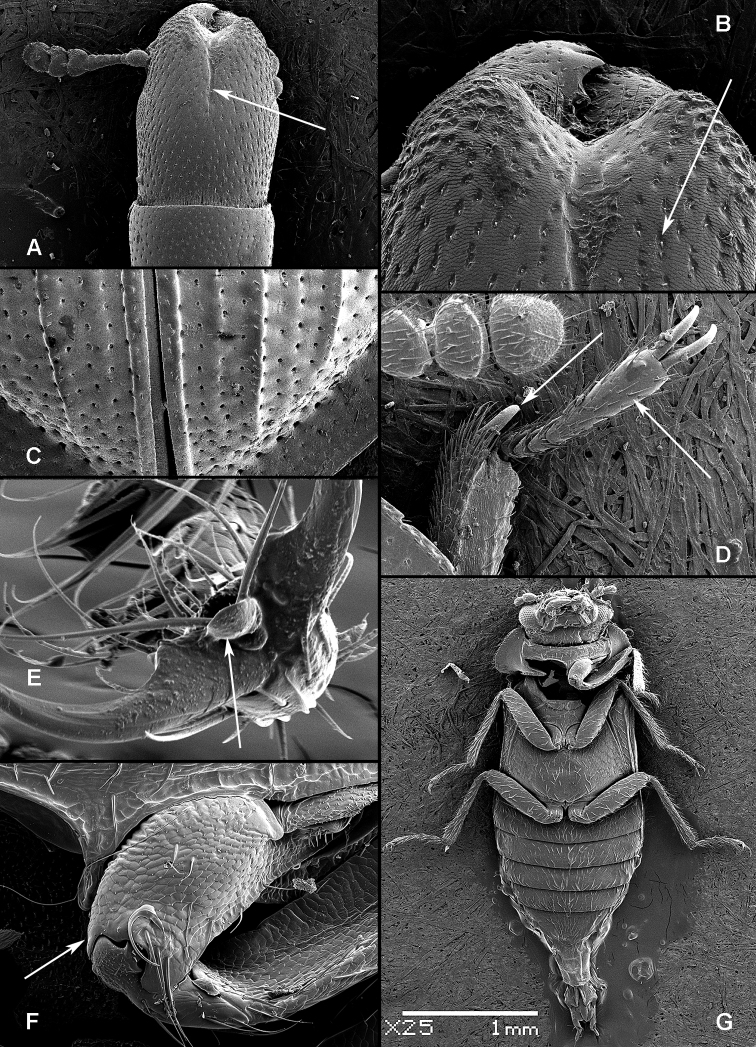
**A–B** *Nemozoma elongatum* (**A** cranium with longidutinal groove **B** detail of elongate punctures) **C–D** *Peltis ferruginea* (**C** elytral apex **D** antennal club, hooked spur and protarsus with large 5th tarsomere) **E–G** *Phloiophilus edwardsi* (**E** metatarsal claws and empodium **F** projecting procoxae **G** ventral surface).

**Figure 16.**
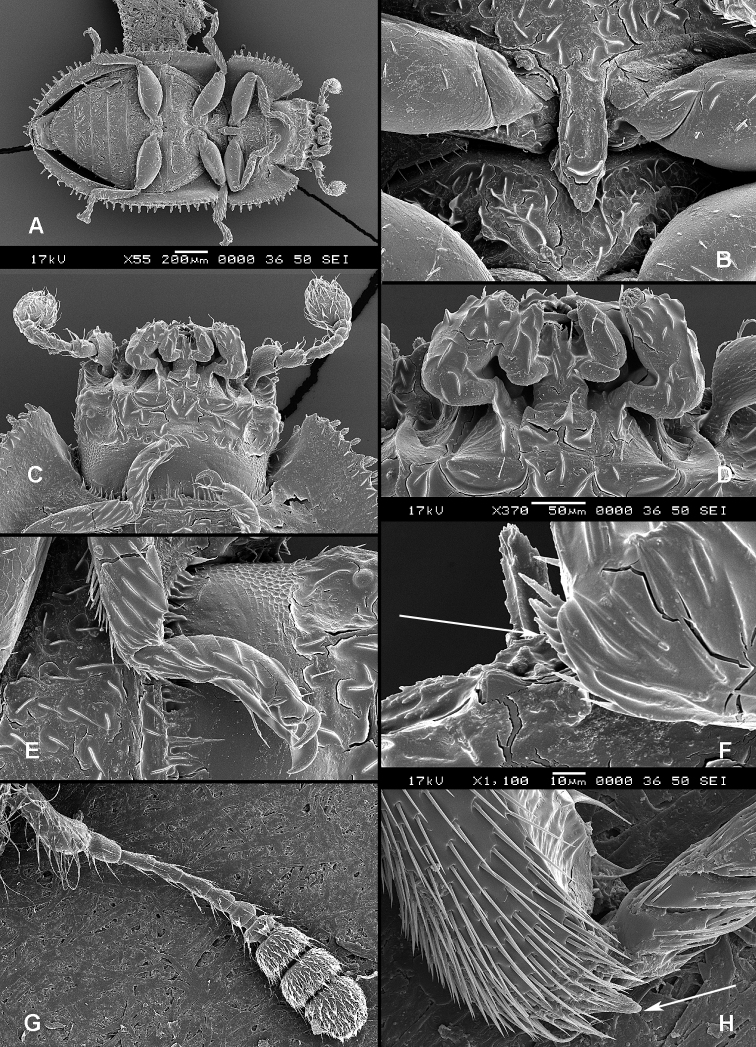
**A–F** *Colydiopeltis compactum* (**A** ventral surface **B** procoxal area **C** head ventrally **D** detail of mouthparts **E** protarsus **F** apex of metatibia with row of spines) **G–H** *Thymalus limbatus* (**G** antenna **H** apex of protibia with straight spur).

**Figure 17.**
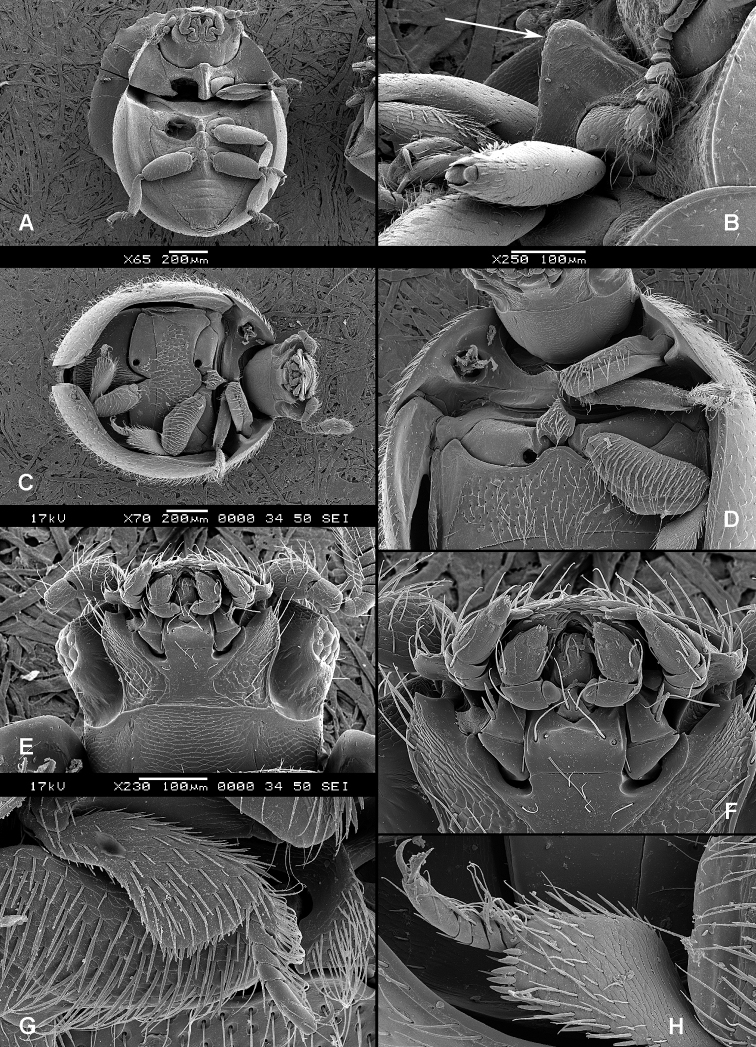
**A–B** “*Rentonellum*” *loebli* (**A** ventral surface **B** prosternal intercoxal process) **C** *cf. Globorentonium plaumanni*, Brazil (**C** ventral surface **D** pro- and mesothorax ventrally **E** head ventrally **F** detail of mouthparts **G** protibia with tarsus **H** mesotibia with tarsus).

**Figure 18.**
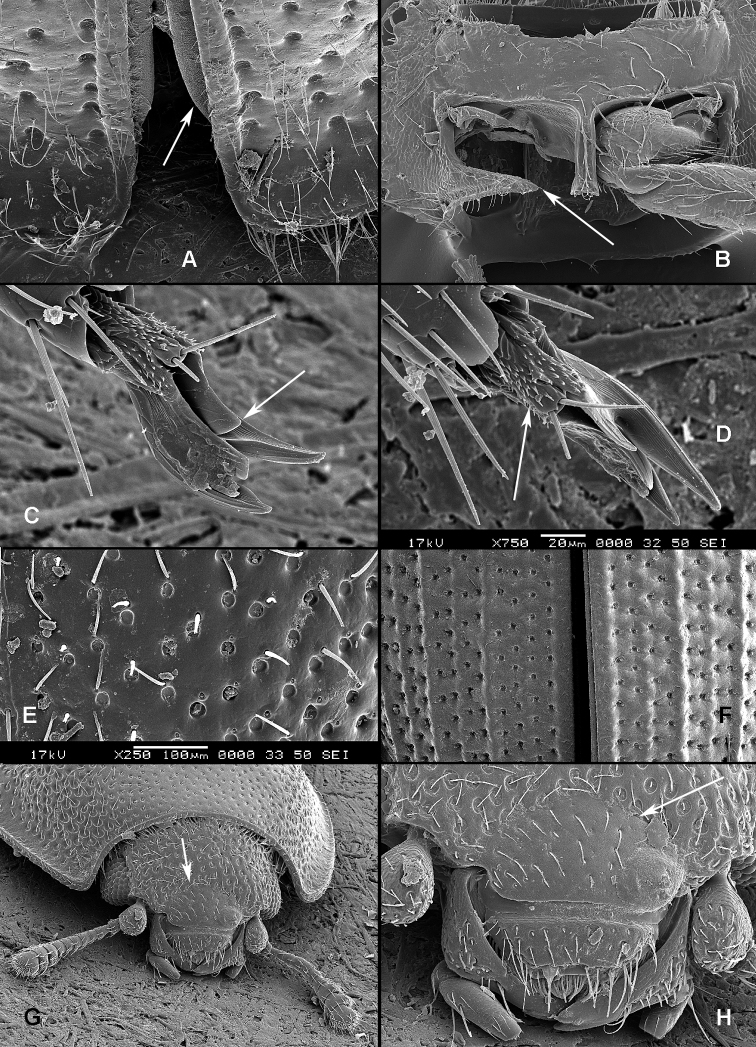
**A** *Thymalus limbatus*, elytral apex with interlocking mechanism **B–E** *Decamerus haemorhoidalis* (**B** prothorax with partially closed procoxal cavities **C** metatarsal claws with denticle **D** protarsus with projecting empodium **E** irregular punctation of elytra) **F** *Grynocharis oblonga*, elytral sculpture **G–H** *Lophocateres pusillus* (**G** head in frontal view with deeply emarginate frontoclypeal suture **H** detail).

**Figure 19.**
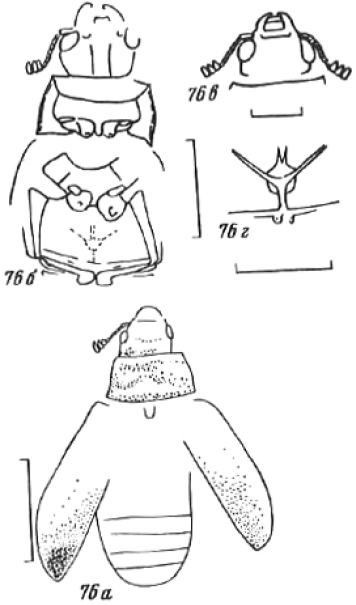
*Cretocateres mongolicus* Ponomarenko, 1986. A reproduction of the original table.

**Figure 20.**
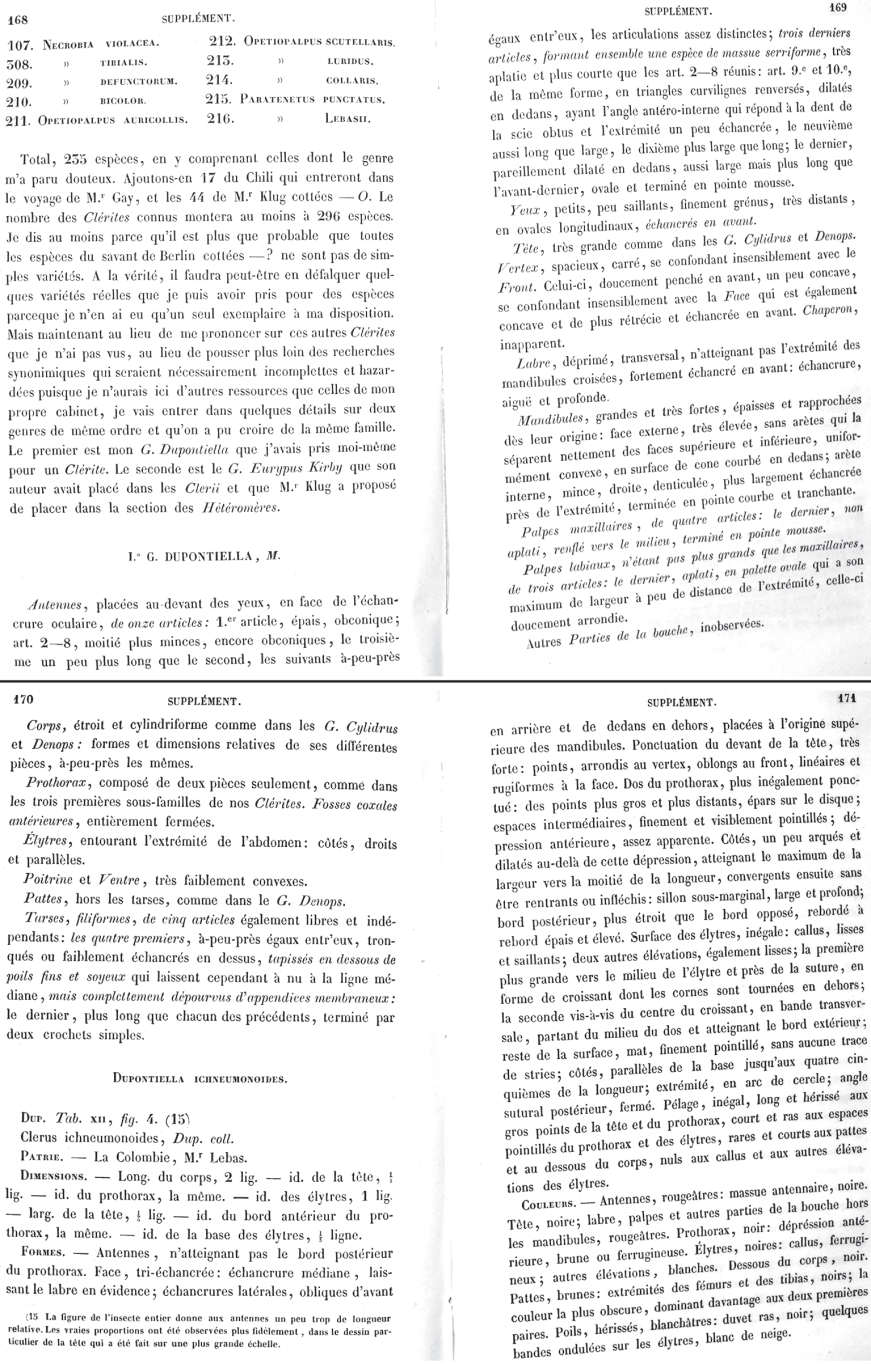
A facsimile of Spinola 1844: 168–171. The original descriptions of the genus *Dupontiella* and the species *Dupontiella ichneumonoides*.

**Figure 21.**
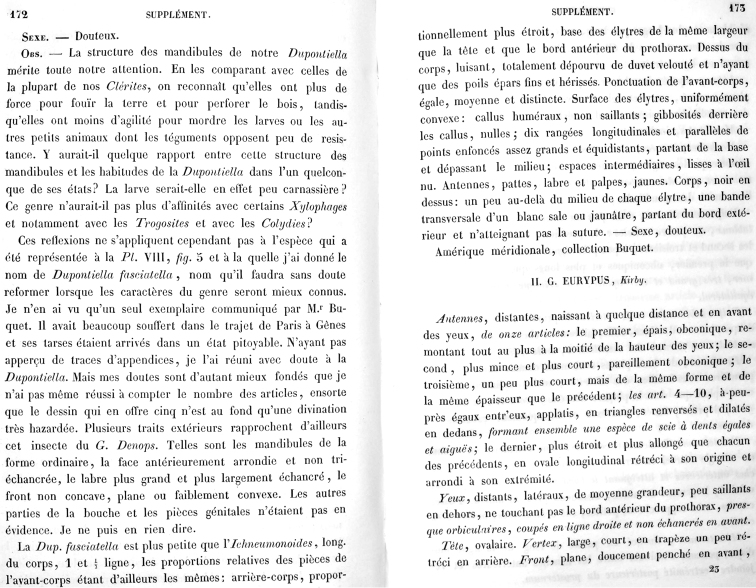
A facsimile of Spinola 1844: 172–173. The original descriptions of the species *Dupontiella ichneumonoides* and *Dupontiella fasciatella*.

**Figure 22.**
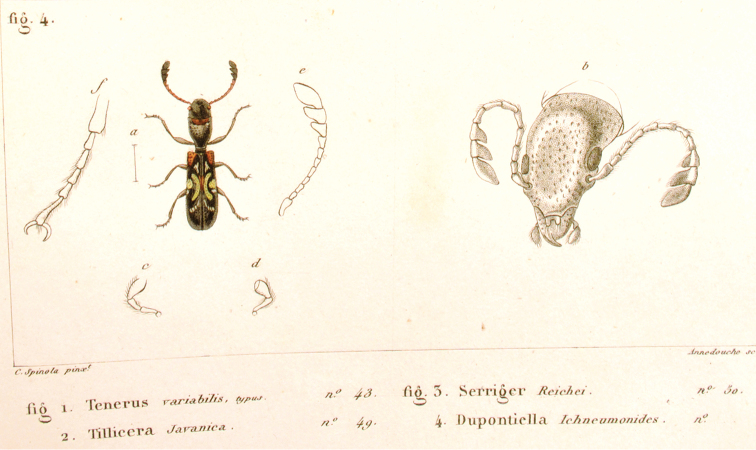
A facsimile of Spinola 1844: Tab XII, fig. 4 (15). The original illustrations of *Dupontiella ichneumonoides*.

**Figure 23.**
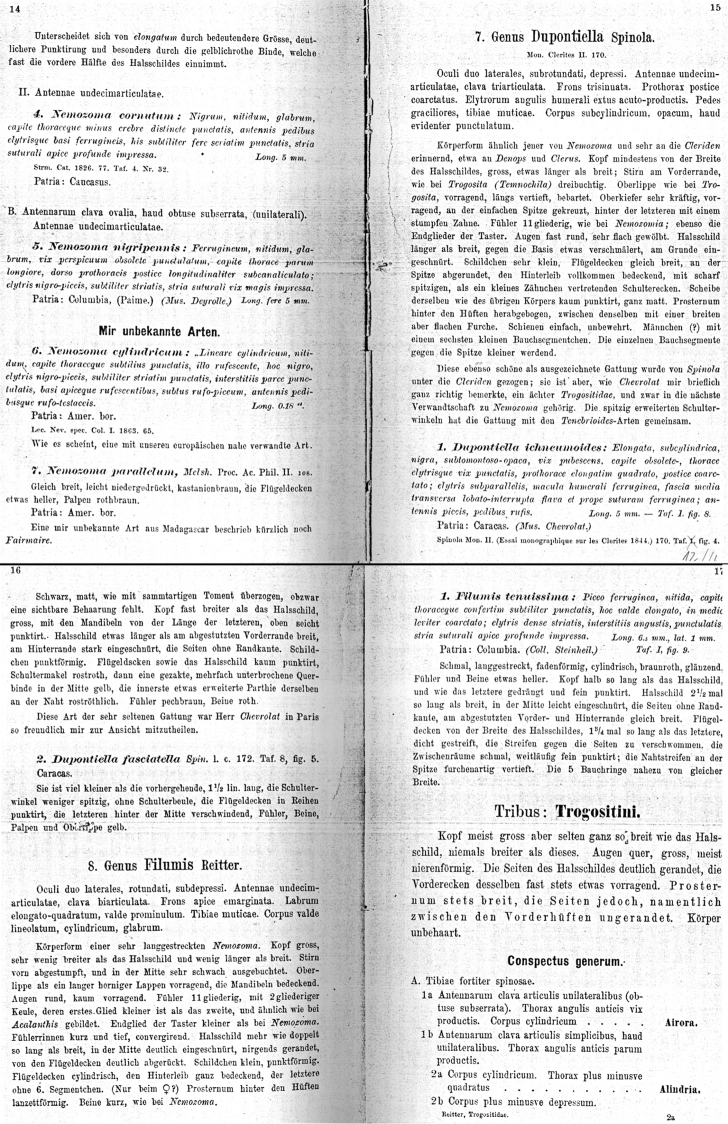
A facsimile of Reitter 1876: 14–17. Author’s remarks on the genus *Dupontiella* and the both species, *Dupontiella ichneumonoides* and *Dupontiella fasciatella*.

**Figure 24.**
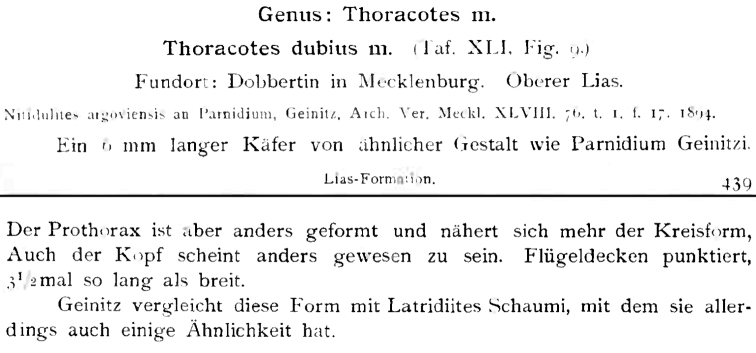
A facsimile of Handlirsch 1906: 438–439. The original description of *Thoracotes dubius*.

**Figure 25.**
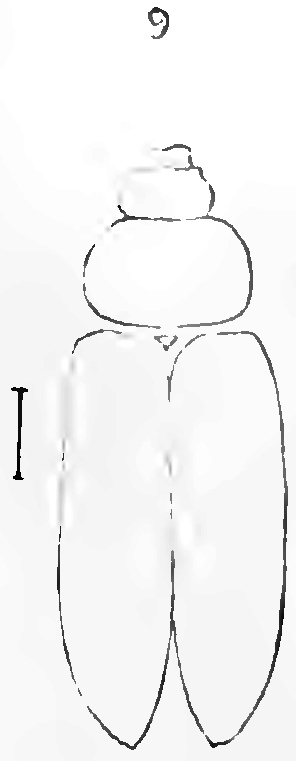
A facsimile of Handlirsch 1906: Tab. XLI, fig. 9. The original illustration of *Thoracotes dubius*.

**Figure 26.**
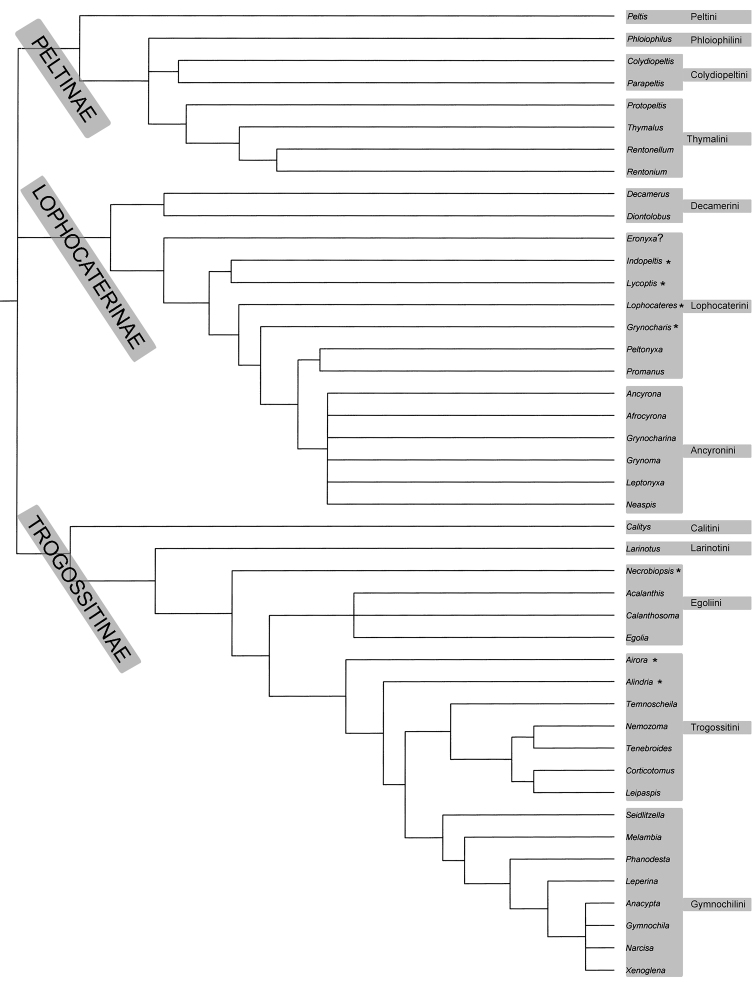
A phylogenetic tree of Trogossitidae adapted and modified from original drawing by Kolibáč (2008). Paraphyletic taxa denoted with the asterisk. Published with kind permission of Pro Entomologia, Basel, a publisher of Entomologica Basiliensia et Collectionis Frey.

## Supplementary Material

XML Treatment for Trogossitidae

XML Treatment for Calityini

XML Treatment for Calitys

XML Treatment for Larinotini

XML Treatment for Larinotus

XML Treatment for Egoliini

XML Treatment for Acalanthis

XML Treatment for Calanthosoma

XML Treatment for Egolia

XML Treatment for Necrobiopsis

XML Treatment for Paracalanthis

XML Treatment for Gymnochilini

XML Treatment for Anacypta

XML Treatment for Gymnocheilis

XML Treatment for Kolibacia

XML Treatment for Leperina

XML Treatment for Narcisa

XML Treatment for Phanodesta

XML Treatment for Seidlitzella

XML Treatment for Xenoglena

XML Treatment for Trogossitini

XML Treatment for Airora

XML Treatment for Alindria

XML Treatment for Corticotomus

XML Treatment for Cretocateres

XML Treatment for Dupontiella

XML Treatment for Elestora

XML Treatment for Eotenebroides

XML Treatment for Eupycnus

XML Treatment for Euschaefferia

XML Treatment for Leipaspis

XML Treatment for Melambia

XML Treatment for Nemozoma

XML Treatment for Parallelodera

XML Treatment for Temnoscheila

XML Treatment for Tenebroides

XML Treatment for Thoracotes

XML Treatment for Lithostomatini

XML Treatment for Lithostoma

XML Treatment for Peltinae

XML Treatment for Juralithinus

XML Treatment for Sinopeltis

XML Treatment for Peltini

XML Treatment for Peltis

XML Treatment for Colydiopeltini

XML Treatment for Colydiopeltis

XML Treatment for Parapeltis

XML Treatment for Phloiophilini

XML Treatment for Phloiophilus

XML Treatment for Thymalini

XML Treatment for Australiodes

XML Treatment for Globorentonium

XML Treatment for Parentonium

XML Treatment for Protopeltis

XML Treatment for Rentonellum

XML Treatment for Rentonidium

XML Treatment for Rentonium

XML Treatment for Thymalus

XML Treatment for Lophocaterinae

XML Treatment for Cretamerus

XML Treatment for Decamerini

XML Treatment for Antixoon

XML Treatment for Decamerus

XML Treatment for Diontolobus

XML Treatment for Ancyronini

XML Treatment for Afrocyrona

XML Treatment for Ancyrona

XML Treatment for Grynoma

XML Treatment for Leptonyxa

XML Treatment for Neaspis

XML Treatment for Sinosoronia

XML Treatment for Lophocaterini

XML Treatment for Eronyxa

XML Treatment for Grynocharina

XML Treatment for Grynocharis

XML Treatment for Indopeltis

XML Treatment for Lophocateres

XML Treatment for Lycoptis

XML Treatment for Peltonyxa

XML Treatment for Promanus

XML Treatment for Promanodes

XML Treatment for Trichocateres

XML Treatment for Anhuistoma

XML Treatment for Peltocoleops

XML Treatment for Meligethiellinae

XML Treatment for Meligethiella

XML Treatment for Ostomalynus

XML Treatment for Palaeoendomychus

## References

[B1] Abbott I (1993) Review of the ecology and control of the introduced bark beetle Ips grandicollis (Eichhoff) (Coleoptera: Scolytidae) in Western Australia, 1952–1990. CALMScience 1(1): 35-46.

[B2] Alexander KNA (1997) The dead-wood beetles of Stourhead, including *Thymalus limbatus* (Fabricius) (Peltidae) new to Wiltshire. Coleopterist 4(3): 90.

[B3] Arbogast RT Byrd RV (1982) The egg of the cadelle, *Tenebroides mauritanicus* (L.) (Coleoptera: Trogositidae): fine structure of the chorion. Entomological News 93(3): 61-66.

[B4] Arias E et al. (2009) A review of the Chilean Egoliini (Coleoptera: Trogossitidae) with description of a new species of Necrobiopsis Crowson. Zootaxa 2170: 37-45.

[B5] Audisio P Gobbi G Liberti G Nardi G (1995) Coleoptera Polyphaga IX (Bostrichoidea, Cleroidea, Lymexyloidea). In: Minelli A Ruffo S La Posta S (Eds) Checklist delle specie della fauna italiana 54 Calderini, Bologna, 27 pp.

[B6] Baader EJ (1989) Pityogenes spp. (Col., Scolytidae): Untersuchungen uber verhaltenssteuernde Duftstoffe und deren Anwendung im Waldschutz. Journal of Applied Entomology 107(1): 1-31. doi: 10.1111/j.1439-0418.1989.tb00223.x

[B7] Bacianskas V (2009) Protected species of beetles (Coleoptera) found in Lithuania in 2004–2009. Rare for Lithuania Insect Species Records and Descriptions 21: 30-31.

[B8] Bahillo de la Puebla P López-Colón JI (1999) Coleoptera: Fam. 37–38: Cleridae, Trogossitidae. In: Catalogus de la entomofauna aragonesa No. 20: 20 pp.

[B9] Bahillo de la Puebla P López-Colón JI (2004) La familia Trogossitidae Latreille, 1802 en la Península Ibérica (Coleoptera, Cleroidea). Estudios del Museo de Ciencias Naturales de Álava 18–19: 127–152.

[B10] Baier P (1991) Zur Biologie des Borkenkäferraubers *Nemosoma elongatum* (L.) (Col.: Ostomidae). Zeitschrift fuer Angewandte Zoologie 78(4): 421-431. doi: 10.1111/j.1439-0418.1994.tb00706.x

[B11] Baier P (1994) Untersuchungen zur abundanzdynamischen Relevanz der Beifange von *Nemosoma elongatum* (L.) (Col., Ostomidae) in *Chalcoprax bekoderten* Flugbarrierefallen fur *Pityogenes chalcographus* (L.) (Col., Scolytidae). Journal of Applied Entomology 117(1): 51-57.

[B12] Barret RE (1932) New Coleoptera from California. Pan-Pacific Entomologist 8: 171-172.

[B13] Barron JR (1971) A revision of the Trogositidae of America North of Mexico (Coleoptera: Cleroidea). Memoirs of the Entomological Society of Canada 75: 1-143. doi: 10.4039/entm10375fv

[B14] Barron JR (1975) A review of the genus *Lycoptis* Casey (Coleoptera: Trogositidae). Canadian Entomologist 107: 1117-1122. doi: 10.4039/Ent1071117-10

[B15] Barron JR (1996) Review of Nearctic species of Ostoma (Coleoptera: Cleroidea, Trogositidae). Annals of the Entomological Society of America 89(2): 193-202.

[B16] Bashford R (1991) Wood-boring Coleoptera and associated insects reared from *Acacia dealbata* Link in Tasmania. Australian Entomological Magazine 18(3): 103-110.

[B17] Bashford R (1994) Life history and mortality of the longicorn *Epithora dorsalis* MacLeay (Coleoptera: Cerambycidae) in Tasmania. Australian Entomologist 21(4): 125-136.

[B18] Basilewsky P (1956) Contributions a l’étude de la faune entomologique du Ruanda-Urundi (Mission P. Basilewsky 1953). Coleoptera. Annales Museum Congo belge (Sér. 8), Sci. Zool. 51: 388-393.

[B19] Bercedo Paramo P Arnaiz Ruiz L Bahillo de la Puebla P Lopez-Colon JI (2006) Record of the presence of *Thymalus limbatus* (Fabricius, 1797) in the autonomous region of Castilla y Leon (Coleoptera, Trogossitidae). Boletin de la SEA 39: 180.

[B20] Beutel RG Pollock DA (2000) Larval head morphology of *Phycosecis litoralis* (Pascoe) (Coleoptera: Phycosecidae) with phylogenetic implications. Invertebrate Taxonomy 14: 825-835. doi: 10.1071/IT00030

[B21] Beutel RG Ślipiński SA (2001) Comparative study of head structures of larvae of Sphindidae and Protocucujidae (Coleoptera: Cucujoidea). European Journal of Entomology 98: 219-232.

[B22] Billings RF Cameron RS (1984) Kairomonal responses of Coleoptera, Monochamus titillator (Cerambycidae), *Thanasimus dubius* (Cleridae), and *Temnochila virescens* (Trogositidae), to behavioral chemicals of southern pine bark beetles (Coleoptera: Scolytidae). Environmental Entomology 13(6): 1542-1548.

[B23] Billings RF (1985) Southern pine bark beetles and associated insects. Effects of rapidly-released host volatiles on response to aggregation pheromones. Zeitschrift Für Angewandte Entomologie 99(5): 483-491.

[B24] Bocáková M Constantin R Bocák L (2011) Molecular phylogenetics of the melyrid lineage (Coleoptera: Cleroidea). Cladistics 27: 1-13. 10.1111/j.1096-0031.2011.00368.x 34861761

[B25] Boone CKS Diana LS Kenneth FR (2008) The enemy of my enemy is still my enemy: competitors add to predator load of a tree-killing bark beetle. Agricultural and Forest Entomology 10(4): 411-421. doi: 10.1111/j.1461-9563.2008.00402.x

[B26] Boosten G (1983) Coléoptères de Belgique. 11. Bulletin and Annales de la Societe Royale Belge d’Entomologie 119(10–12): 290-293.

[B27] Borowiec L (1983) Zeszyt 69. Paweznikowate – Peltidae. In: Klucze do oznaczania owadów Polski. Czesc XIX. Panstwowe wydawnictwo naukowe, Warszawa-Wroclaw, 16 pp.

[B28] Bouchard P Bousquet Y Davies A Alonso-Zarazaga M Lawrence J Lyal C Newton A Reid C Schmitt M Slipinski A Smith A (2011) Family-group names in Coleoptera (Insecta). Zookeys 88: 1-972. doi: 10.3897/zookeys.88.807 PMC3088472 21594053

[B29] Böving AG Craighead FC (1931) An illustrated synopsis of the principal larval forms of the order Coleoptera. Entomologica Americana, 1–351. doi: 10.5962/bhl.title.6818

[B30] Brookes A (1932) A new genus and six new species of Coleoptera. Transactions and Proceedings of the New Zealand Institute 63: 25-33.

[B31] Broun T (1909) Notes on Coleoptera from the Chatham Islands. Transactions of the New Zealand Institute 41: 145-150.

[B32] Broun T (1915) Descriptions of new genera and species of Coleoptera. Bulletins of New Zealand Institute 1: 267-346.

[B33] Brustel H (2009) Antrodia spp. – polypore hosts of rare *Quilnus* spp. (Heteroptera Aradidae) and *Calitys scabra* (Coleoptera Trogositidae) and discovery of a high entomological site in the Pyrenees: the Rioumajou valley. Entomologiste 65(5): 227-232.

[B34] Burakowski B Mroczkowski M Stefanska J (1986) Chrzaszcze, Coleoptera. Dermestoidea, Bostrichoidea, Cleroidea, Lymexylonoidea. In: Katalog Fauny Polski. XXIII, tom 11 PWN Warszawa, 244 pp.

[B35] Carter HJ Zeck EH (1937) A monograph of the Australian Colydiidae. Proceedings of Linnean Society of New South Wales 62: 181–208, pls 8, 9.

[B36] Casey TL (1890) Coleopterological notices. II. Annals of the New York Academy of Sciences 5: 307-504. doi: 10.1111/j.1749-6632.1890.tb57008.x

[B37] Casey TL (1916) Some random studies among the Clavicornia. Memoirs on the Coleoptera 7: 35–292.

[B38] Casey TL (1924) Addition to the known Coleoptera of North America. Memoirs on the Coleoptera 11: 1-347.

[B39] Chang T-C Liu T-Y (1981) Insect pests of stored paddy in natural – ventilation and forced ventilation storehouses. National Science Council Monthly 9(2): 116-119.

[B40] Chûjô M Lee CE (1994) Trogositidae, Languriidae, Tenebrionidae and Alleculidae from Korea (incl. Chejudo Is.) (Coleoptera). Esakia 34: 187-193.

[B41] Conrad R (1995) The second contribution to the beetle fauna of Thuringia (Insecta, Coleoptera). Thueringer Faunistische Abhandlungen 2: 190-195.

[B42] Costa C Vanin SA Casari-Chen SA (1988) Larvas de Coleoptera do Brasil. Museu de Zoologia, Universidade de Sao Paulo, 282 pp.+165 tabs.

[B43] Crowson RA (1955) The natural classification of the families of Coleoptera. Nathaniel Lloyd + Co., Ltd., London, 187 pp.

[B44] Crowson RA (1964a) A review of the classification of Cleroidea (Coleoptera), with descriptions of two genera of Peltidae and of several new larval types. Transaction of the Royal entomological Society of London 116: 275-327.

[B45] Crowson RA (1964b) The habits and life cycle of *Phloiophilus edwardsi* Steph. (Coleoptera: Phloiophilidae). Proceedings of the Royal entomological Society of London (A) 39: 151-152.

[B46] Crowson RA (1966) Further observations on Peltidae (Coleoptera: Cleroidea), with definitions of a new subfamily and of four new genera. Proceedings of the Royal entomological Society of London 35: 119-127.

[B47] Crowson RA (1967) The natural classification of the families of Coleoptera. Addenda et Corrigenda. Entomologist’s Monthly Magazine 212: 209-214.

[B48] Crowson RA (1970) Further observations on Cleroidea (Coleoptera). Proceedings of the Royal entomological Society of London (B) 39: 1-20.

[B49] Crowson RA (1972) On the systematic value of the alimentary canal in Cleridae. Systematic Zoology 21: 339-340. doi: 10.2307/2412171

[B50] Cunev J (1999) Chrobáky (Coleoptera) Hornej Oravy (severné Slovensko). Acta Rerum naturalium Musei naturalis Slovakiae 45: 53-94.

[B51] Daccordi M De Little DW (2003) New taxa of Chrysomelinae of Tasmania (Coleoptera Chrysomelidae: Chrysomelinae). Museo Regionale di Scienze Naturali Monografie (Turin) 35: 343-378.

[B52] Daccordi M (2003a) New species of Chrysomelinae from New South Wales (Coleoptera Chrysomelidae: Chrysomelinae). Museo Regionale di Scienze Naturali Monografie (Turin) 35: 379-412.

[B53] Daccordi M (2003b) New taxa of Chrysomelinae of Queensland (Coleoptera Chrysomelidae: Chrysomelinae). Museo Regionale di Scienze Naturali Monografie (Turin) 35: 413-460.

[B54] Dahlsten DL (2004) Attraction of Ips pini (Coleoptera: Scolytinae) and its predators to natural attractants and synthetic semiochemicals in northern California: Implications for population monitoring. Environmental Entomology 33(6): 1554-1561. doi: 10.1603/0046-225X-33.6.1554

[B55] Dejean PF MA (1835) Catalogue des coléoptères de la collection de M. le comte Dejean, Deuxième édn [Livraison 4]. Méquignon-Marvis Père et Fils, Paris.

[B56] Dajoz R (1991) Une espèce nouvelle de Cylidrella de Californie. Son intérêt écologique et biogéographique. Nouvelle Revue Entomologique (n.s.) 7: 245-250.

[B57] Dajoz R (1997) Description de *Corticotomus testaceus* n. sp. (Coleoptera Trogositidae) et notes sur trois espèces de l’Ouest des Etats-Unis. Bull. mens. Soc. linn. Lyon 66: 40-44.

[B58] Delobel A Tran M (1993) Les Coléoptères des denrées alimentaires entreposées dans les régions chaudes. Faune Tropicale 32: 1-425.

[B59] DeMars CJ Dahlstena DL Sharpnacka NX Rowneya DL (1986) Tree utilization and density of attacking and emerging populations of the western pine beetle (Coleoptera: Scolytidae) and its natural enemies, Bass Lake, California, 1970–1971. Canadian Entomologist 118(9): 881-900. doi: 10.4039/Ent118881-9

[B60] Dippel C (1991) Zur Biologie des Borkenkäferprädators *Nemosoma elongatum*. Naturwissenschaften 78(10): 473-474. doi: 10.1007/BF01134390

[B61] Dippel C (1995) Zur Bionomie des Borkenkäferantagonisten *Nemosoma elongatum* L. (Col., Ostomidae). Mitteilungen der Deutschen Gesellschaft fuer Allgemeine und Angewandte Entomologie 10(1–6): 67-70.

[B62] Dippel C (1996) Investigations on the life history of *Nemosoma elongatum* L. (Col., Ostomidae), a bark beetle predator. Journal of Applied Entomology 120(7): 391-395. doi: 10.1111/j.1439-0418.1996.tb01626.x

[B63] Dippel C Heidger C Nicolai V Simon M (1997) The influence of four different predators on bark beetles in European forest ecosystems (Coleoptera: Scolytidae). Entomologia Generalis 21(3): 161-175.

[B64] Dominguez-Sanchez B et al. (2008) Kairomonal response of coleopterans associated with Dendroctonus frontalis and two Ips species (Coleoptera: Curculionidae) in forest of Chiapas, Mexico. Revista Mexicana de Biodiversidad 79(1): 175-183.

[B65] Dow RP (1912) A new variety of *Trogosita virescens*. Journal of New York Entomological Society 20: 70-71.

[B66] Downie NM Arnett RH Jr (1996) The beetles of northeastern North America. Volume 2: Polyphaga: Series Bostrichiformia through Curculionoidea. Sandhill Crane Press, Gainesville, i–x, 891–1721.

[B67] Dziadik-Turner C Kogaa D Mai MS Kramer KJ (1981) Purification and characterization of two [beta]-N-acetylhexosaminidases from the tobacco hornworm, *Manduca sexta* (L.) (Lepidoptera: Sphingidae). Archives of Biochemistry and Biophysics 212(2): 546-560. doi: 10.1016/0003-9861(81)90398-2 7325679

[B68] Endrödy-Younga S (1960) Neue Angaben zur Klärung des Systems der Familie Clambidae und Beschreibung einer neuen Liodiden-Gattung (Coleoptera). Annales historico-naturales Musei Nationalis Hungarici 52: 239-245.

[B69] Erber D Wheater CP (1987) The Coleoptera of the Selvagem Islands, including a catalogue of the specimens in the Museu municipal do Funchal. Boletim do Museu Municipal do Funchal 39: 156-187.

[B70] Erichson WF (1842) Beitrag zur Insecten-Fauna von Vandiemensland, mit besonderer Berücksichtigung der geographischen Verbreitung der Insecten. Archiv für Naturgeschichte 8: 83–287, 2 pls.

[B71] Erichson WF (1844) Einige Nachträge su meinem Versuch einer systematischen Eintheilung der Nitidularien. Germar’s Zeitschrift für die Entomologie 5: 438-458.

[B72] Esaki T et al. (1951) Iconographia Insectorum Japonicorum. Editio secunda reformata, Hokuryukan, Tokyo.

[B73] Fabricius JC (1801) Systema Eleutheratorum secundum ordines, genera, species, adjectis synonymis, locis, observationibus, descriptionibus. Vol. I Bibliopolii Academici Novi, Kiliae.

[B74] Fairmaire L (1881) Coléopteres des iles Viti (Fidgi). Annales de la Société Entomologique de France Sér. 6, 1: 243–318.

[B75] Fall HC (1910) Miscellaneous notes and descriptions of North American Coleoptera. Transaction of the American entomological Society 36: 89-197.

[B76] Faustini DI Halstead DGH (1982) Setiferous structures of male Coleoptera. Journal of Morphology 173: 43-72. doi: 10.1002/jmor.1051730106 30096963

[B77] Fettig CJ Dabney CP (2006) Seasonal abundance of *Temnochila chlorodia* (Mannerheim) (Coleoptera: Trogositidae) collected in western pine beetle pheromone-baited traps in northern California. Journal of Entomological Science 41(1): 75-83.

[B78] Fettig CJ et al. (2004) Field response of Dendroctonus valens (Coleoptera: Scolytidae) and a major predator, *Temnochila chlorodia* (Coleoptera: Trogositidae), to host kairomones and a Dendroctonus spp. pheromone component. Journal of Entomological Science 39(4): 490-499.

[B79] Fettig CJ Borys RR Dabney CP McKelvey SR Cluck DR Sherl L (2005) Disruption of red turpentine beetle attraction to baited traps by the addition of California fivespined ips pheromone components. Canadian Entomologist 137(6): 748-752. doi: 10.4039/n05-042

[B80] Fettig CJ Borys RR Dabney CP McKelvey SR Cluck DR Smith SL (2007) The response of Dendroctonus valens and *Temnochila chlorodia* to Ips paraconfusus pheromone components and verbenone. Canadian Entomologist 139(1): 141-145. doi: 10.4039/n06-013

[B81] Fjellberg A Hansen SO (1997) *Peltis grossa* (L.) – still present in Norway (Coleoptera, Trogositidae). Fauna Norvegica Series B44(1): 77. doi: 10.4039/n06-013

[B82] Foster DE (1976) North American Thaneroclerinae larvae (Coleoptera: Cleridae). The Coleopterists Bulletin 30: 75-80.

[B83] Franz H (1981) Ein neuer *Thymalus* aus Tunesien, (Ostomidae, Col.). Koleopterologische Rundschau 55: 51-52.

[B84] Fursov NI (1930) Novyye vidy zhestkokrylykh, semeistva Ostomidae [New species of Coleoptera belonging to the genus *Nemosoma* (Ostomidae)]. Trudy Instituta Drevesiny (Leningrad) 1929: 181-183.

[B85] Gaylord MLW (2008) Influence of temperature on spring flight initiation for southwestern ponderosa pine bark beetles (Coleoptera: Curculionidae, Scolytinae). Environmental Entomology 37(1): 57-69. doi: 10.1603/0046-225X(2008)37[57:IOTOSF]2.0.CO;2 18348797

[B86] Ghose S Haldar DP (1989) Role of environmental factors in the incidence of two new species of apicomplexan parasites, *Hirmocystis lophocateri* sp. n. and *Hirmocystis triboli* sp. n. from coleopteran insects. Acta Protozoologica 28(1): 49-60.

[B87] Ghosh S Saha K (1992a) Experimental application of *Nosema* sp. on *Lophocateres pusillus* (K.) (Coleoptera: Curculionidae). Acta Protozoologica 31(3): 181-184.

[B88] Ghosh S Saha K (1992b) Effects of *Nosema* sp. on the molting of a stored-grain pest *Lophocateres pusillus* (Coleoptera: Trogositidae). Environment and Ecology (Kalyani) 10(3): 613-617.

[B89] Ghosh S Saha K (1995) Impact of temperature and relative humidity on the incidence of a microsporan parasite, *Nosema* sp. I, infecting coleopteran stored grain pest, *Lophocateres pusillus*. Journal of Entomological Research (New Delhi) 19(3): 207-214.

[B90] Gimmel M Leschen RA B (in press) A new species of mycophagous *Rentonium* (Coleoptera: Cleroidea: Trogossitidae) based on larvae and adults, and a catalogue of Rentoniinae. Journal of Natural History.

[B91] Gobbi G (1983) Appunti su Coleotteri Cleroidei predatori di xilofagi (Coleoptera, Trogositidae, Cleridae, Melyridae). Bolletino Associazione Romana di Entomologia 38: 51-62.

[B92] Goheen DJ (1985) Visitation frequencies of some insect species on Ceratocystis wageneri infected and apparently healthy ponderosa pines. Canadian Entomologist 117(12): 1535-1543. doi: 10.4039/Ent1171535-12

[B93] Gollkowski V (1988) *Tenebrioides mauretanicus* L. und *T. fuscus* Goeze im Vogtland (Col., Ostomidae). Entomologische Nachrichten und Berichte 32(1): 42.

[B94] Gorham HS (1886) Insecta. Coleoptera. Malacodermata. Supplement. In: Godman FD Salvin O (Eds) Biologica Centrali-Americana. Volume III. Part 2. London, Dulau, Co., B. Quaritsch: 313-372.

[B95] Gourves J (2006) Contribution to the study of Ostomidae from Morocco (Coleoptera). Contribution a l’etude des Ostomidae du Maroc (Coleoptera). Revue de l’Association Roussillonnaise d’Entomologie 15(2): 56-60.

[B96] Gray DW (2002) Field response of Ips paraconfusus, *Dendroctonus brevicomis*, and their predators to 2-methyl-3-buten-2-ol, a novel alcohol emitted by ponderosa pine. Journal of Chemical Ecology 28(8): 1583-1597. doi: 10.1023/A:1019924428970 12371811

[B97] Gray GR (1832) Notices of new genera and species. The Animal Kingdom Arranged in Conformity with its Organization; by the Baron Cuvier, Member of the Institute of France, &c. &c. &c, Vol.14, In: Griffith E (Ed) The Class Insecta Arranged by the Baron Cuvier, Vol. 1, Whittaker, Treacher, and Co., London, viii + 1–570.

[B98] Griffith E Pidgeon E (1832) The Class Insecta Arranged by the Baron Cuvier, with Supplementary Additions to Each Order. And Notices of New Genera and Species by George Gray, Esq, Vol. 1 Whittaker, Treacher, and Co., London.

[B99] Grouvelle A (1913) H. Sauter‘s Formosa-Ausbeute (Rhysodidae, Nitidulidae, Ostomidae, Colydiidae, Passandridae, Cucujidae, Cryptophagidae, Diphyllidae, Lathridiidae, Mycetophagidae, Dermestidae). Archiv für Naturgeschite 79A: 33–73.

[B100] Gueorguiev G Georgiev G Mirchev M Tsankov T (2003) Four new coleopteran species (Insecta: Coleoptera) for Bistrishko Branishte biosphere reserve in the Vitosha Mountain, Bulgaria. Acta Zoologica Bulgarica 55(1): 107-112.

[B101] Gunter NL Leavengood JM Jr Bartlett JS Chapman EG Cameron SL (2013) A molecular phylogeny of the checkered beetles and a description of Epiclininae a new subfamily (Coleoptera: Cleroidea: Cleridae). Systematic Entomology 38: 626-636. doi: 10.1111/syen.12019

[B102] Gürlich S Suikat R Ziegler W (1995) Katalog der Käfer Schleswig-Holsteins und des Niederelbegebietes. Verhandlungen des Vereins für naturwissenschaftliche Heimatforschung zu Hamburg 41: 1-111.

[B103] Hallan J (2007–2012) Biology Catalogue. Texas A&M University http://insects.tamu.edu/research/collection/hallan/test/Arthropoda/Insects/Coleoptera/Family/Trogossitidae.txt [Last updated: March 24, 2008, accessed November 2009]

[B104] Handlirsch A (1906–1908) Die fossilen Insekten und die Phylogenie der rezenten Formen. Ein Handbuch für Paläontologen und Zoologen. Engelmann, Leipzig, 1430 pp.

[B105] Hansen SO Borgersen B (1991) *Calitys scabra* (Thunberg 1784) (Col., Trogositidae) rediscovered in Norway, new species for Vestfold. Fauna Norvegica Series B38(1): 40.

[B106] Hatch MH (1962) The beetles of the Pacific Northwest. III: Pselaphidae and Diversicornia I. University of Washington Publications in Biology 16: iii–iii.

[B107] Hawkeswood TJ (1991) Notes on the biology of *Leperina cirrosa* Pascoe (Coleoptera: Trogossitidae). Victorian Entomologist 21(6): 159-161.

[B108] Hawkeswood TJ (1992) Observations on the biology of the Australian beetle *Leperina cirrosa* Pascoe (Coleoptera: Trogossitidae). Bulletin and Annales de la Societe Royale Belge d’Entomologie 128(7–9): 229-233.

[B109] Hayes JL Johnson PL Eglitis A Scott DW Spiegel L Schmitt CL Smith SE (2008) Response of bark and woodboring beetles to host volatiles and wounding on western juniper. Western Journal of Applied Forestry 23(4): 206-215.

[B110] Heer O (1862) Beitraege zur Insekenfauna Oeningens. Coleoptera-Geodephagen, Hydrocanthariden, Gyriniden, Brachelytren, Clavicornen, Lamellicornen und Buprestiden. Natuurkundige verhandelingen van de Hollandsche maatschappij der wetschappen te Haarlem 2: 1-90.

[B111] Herger P (1998) Record of *Tenebroides fuscus* (Goeze, 1777) in the high montane zones of the Napf area (Coleoptera, Trogositidae). Entomologische Berichte Luzern 39: 105-106.

[B112] Heuer H Vite JP (1984a) Chalcogran: unique kairomone-governed predator-prey relations among ostomid and scolytid beetles. Naturwissenschaften 71(4): 214-215. doi: 10.1007/BF00490438

[B113] Heuer H Vite JP (1984b) Prey spectrum of *Nemosoma elongatum* L. (Coleoptera: Ostomidae) governed by kairomone response. International Congress of Entomology Proceedings 17: 586.

[B114] Hieke F Pietrzeniuk E (1984) Die Bernstein-Käfer des Museums fur Naturkunde, Berlin (Insecta, Coleoptera). Mitteilungen aus dem zoologischen Museum in Berlin 60: 297-326.

[B115] Hilszczanski J (2006) *Nemosoma* caucasicum Menetries, 1832 (Coleoptera: Trogossitidae) – nowy dla fauny Polski gatunek chrzaszcza. Wiadomoszczi entomologiczne 25: 29-32.

[B116] Holub A Snizek M (1997) Faunistic record from the Czech Republic – 72. Coleoptera: Trogositidae. Klapalekiana 33(3–4): 150.

[B117] Holzer E (1995) Erstnachweise und Wiederfunde für die Käferfauna der Steiermark (Coleoptera). Mitteilungen der Abteilung für Zoologie am Landesmuseum Joanneum in Graz 49: 23-47.

[B118] Hope FW (1840) The coleopterist’s manual, part the third, containing various families, genera, and species, of beetles, recorded by Linneus and Fabricius. Also, descriptions of newly discovered and unpublished insects. Bridgewater, Bowdery, Kerby, London, 191 pp, 3pls.

[B119] Huber C Kobel E (1994) Zum Vorkommen von *Tenebroides fuscus* (Goeze, 1777) in der Schweiz (Coleoptera, Trogositidae). Mitteilungen der Schweizerischen Entomologischen Gesellschaft 67(1–2): 1-5.

[B120] Hunt T Bergsten J Levkanicova Z Papadopoulou A St.John O Wild R Hammond PM Ahrens D Balke M Caterino MS Gomez-Zurita J Ribera I Barraclough TG Bocakova M Bocak L Vogler AP (2007) A Comprehensive Phylogeny of Beetles Reveals the Evolutionary Origins of a Superradiation. Science 318: 1913-1916. doi: 10.1126/science.1146954 18096805

[B121] Iablokoff-Khnzorian SM (1975) Bemerkungen über paläarktische Buntkäfer (Coleoptera, Cleridae). Entomol. Blätter 71: 141-148.

[B122] Illiger JK W (1807) Zusätze, Berichtigungen und Bemerkungen zu Fabricii Systema Eleutheratorum. Magazin für Insektenkunde 6: 296-317.

[B123] Inouye M Nobuchi A (1957) Studies on the natural enemies of the bark beetles and borers. Scientific Reports of the Hokkaido Forestry Experimental Station 8: 190-204. [in Japanese, English summary]

[B124] Jakobson GG (1915) Zhuki Rossii i Zapadnoy Evropy. Rukovodstvo k opredeleniu zhukov. Vypusk 11 AF Devrjen, St.-Peterbourg, 865–1024.

[B125] Kampsu G (2005) Studies on the biology and distribution of cadelle, *Tenebroides mauritanicus* (L.) (Coleoptera: Trogossitidae) in Bursa, Turkey. Journal of Entomology 2(1): 17-20. doi: 10.3923/je.2005.17.20

[B126] Karalius S Monsevicius V (1992) 34 new and 3 rare for Lithuania species of Coleoptera found in 1973–1991. In: Jonaitis V (Ed) New and rare for Lithuania insect species: records and descriptions of 1992. Institue of Ecology, Vilnius, 5-11.

[B127] Kiesenwetter EAH von (1863) Naturgeschichte der Insecten Deutschland. Erste Abtheilung. Coleoptera. Vierter Band Nicolaische Verlagsbuchhandlung, Berlin, vi+ 746pp.

[B128] Kirby W (1837) The Insects. In: Richardson J (Ed) Fauna Boreali-Americana, or the Zoology of the Northern Parts of British America,... Part 4. JFletcher, Norwich, 325 pp. + 8 pls.

[B129] Kireichuk AG Ponomarenko AG (1990) Iskopaemye zhuki semeistv Peltidae i Nitidulidae (Coleoptera). Paleontologicheskii zhurnal 1990: 78–88 + Tab. VII.

[B130] Klausnitzer B (1976) Über die Larven der Ostomidae (Col.). Entomologische Nachrichten, Dresden 20: 4-9.

[B131] Klausnitzer B (1978) Melyridae, Cleridae, Ostomidae. In: Ordnung Coleoptera (Larven). Akademie Verlag, Berlin, 378 pp: 123–6, 127–130, 173–8.

[B132] Klausnitzer B (1996) 60–64. Familie. Trogositidae, Peltidae, Phloiophilidae, Lophocateridae, Cleridae. In: Klausnitzer B (Ed) Die Larven der Käfer Mitteleuropas. Band 3. Goecke, Evers, Krefeld. Im Gustav Fischer Verlag, Jena, Stuttgart, 144-187.

[B133] Klimaszewski J Watt JC (1997) Coleoptera: family group review and keys to identification. Fauna of New Zealand 37: 1-199.

[B134] Kobayashi K (1980) The Insecta impaled by the bull-headed shrike Lanius bucephalus bucephalus (Temmink and Schulegel) and the weekly variation of the number of impaled prey. Gensei 37: 1-6.

[B135] Koch K (1993) Dritter Nachtrag zur Käferfauna der Rheinprovinz. Teil 3: Ostomidae – Scolytidae. Decheniana 146: 203-271.

[B136] Kohnle U Vite JP (1984) Bark beetle predators: strategies in the olfactory perception of prey species by clerid and trogositid beetles. Zeitschrift für angewandte Entomologie 98(5): 504-508.

[B137] Kolibáč J (1987) Morphological comparison of type (or model) genera of the subfamilies od Cleridae (Coleoptera, Cleridae). Mitteilungen der Münchner Entomologischen Gesellschaft 77: 103-135.

[B138] Kolibáč J (1993a) Observations on Ancyrona Reitter, 1876, with a key to Central European Trogositidae (Coleoptera, Trogositidae). Nachrichtenblatt der bayerischen Entomologen 42: 16-22.

[B139] Kolibáč J (1993b) Phloiophilidae. Trogositidae. Cleridae. In: Jelínek J et al. (1993) Check-list of Czechoslovak Insects IV (Coleoptera). (Seznam československých brouků). Folia Heyrovskyana Supplementum 1 (Praha), 89–90.

[B140] Kolibáč J (1996) Coleoptera: Cleroidea 1 (Phloiophilidae, Trogositidae and Cleridae). In: Rozkošný R Vaňhara J (Eds) Terrestrial Invertebrates of the Pálava Biosphere Reserve of UNESCO, III. Folia Facultatis Scienciarium Naturalium Universitatis Masarykianae Brunensis, Biologia 94: 471–474.

[B141] Kolibáč J (1999) Comparative morphology of mandible, epipharynx and alimentary canal in larval and adult Cleroidea (Coleoptera). Acta Musei Moraviae, Scientiae biologicae 84: 11-69.

[B142] Kolibáč J (2002) Description of a larva of *Thymalus limbatus* (Fabricius, 1787) (Coleoptera, Trogositidae). Acta Musei Moraviae, Scientiae biologicae 87: 55-59.

[B143] Kolibáč J (2003) Trogositidae. In: Fauna Europaea. http://www.faunaeur.org

[B144] Kolibáč J (2004) Metaxinidae fam.nov., a new family of Cleroidea (Coleoptera). Entomologica Basiliensia 26: 239-268.

[B145] Kolibáč J (2005) A review of the Trogositidae. Part 1: Morphology of the genera (Coleoptera, Cleroidea). Entomologica Basiliensia et Collectionis Frey 27: 39-159.

[B146] Kolibáč J (2006) A review of the Trogossitidae. Part 2: Larval morphology, phylogeny and taxonomy (Coleoptera, Cleroidea). Entomologica Basiliensia et Collectionis Frey 28: 105-153.

[B147] Kolibáč J (2007a) Trogossitidae. In: Löbl I Smetana A (Eds) Catalogue of Palaearctic Coleoptera. Vol. 4 Apollo Books, Stenstrup, 364-366.

[B148] Kolibáč J (2007b) Further observations on the tribe Ancyronini Kolibáč, 2006 (Coleoptera, Trogossitidae, Peltinae). Entomologica Basiliensia et Collectionis Frey 29: 53-76.

[B149] Kolibáč J (2008) Morphology, taxonomy and phylogeny of *Phloiophilus edwardsi* Stephens, 1830 (Coleoptera, Cleroidea). Entomologica Basiliensia et Collectionis Frey 30: 105-133.

[B150] Kolibáč J (2009) Some nomenclatorial notes on the family Trogossitidae (Coleoptera, Cleroidea). Entomologica Basiliensia et Collectionis Frey 31: 127-129.

[B151] Kolibáč J (2010) *Trichocateres fasciculifer*, a new genus and species of Trogossitidae: Lophocaterini (Coleoptera). Zootaxa 2353: 34-42.

[B152] Kolibáč J (2011) *Promanodes alleni* sp. n., the second species of the Tertiary genus *Promanodes* Kolibáč, Schmied, Wappler et Kubisz, 2010, with improved diagnosis of the genus and remarks on its phylogeny (Coleoptera: Trogossitidae). Zootaxa 2928: 57-63.

[B153] Kolibáč J Huang D-Y (2008) Taxonomic status of the Mesozoic genera *Anhuistoma* Lin, 1985, Eotenebroides Ren, 1995, *Lithostoma* Martynov, 1926, *Palaeoendomychus* Zhang, 1992 and Sinosoronia Zhang, 1992 (Coleoptera). Entomologica Basiliensia et Collectionis Frey 30: 135-148.

[B154] Kolibáč J Leschen RAB (2010) Trogossitidae Fabricius, 1801. In: Leschen R Beutel RG Lawrence JF (Eds) Handbuch der Zoologie/Handbook of Zoology. Band 4: Arthropoda, 2. Halfte: Insecta. Part 39: Coleoptera, Beetles Vol. 2 W. de Gruyter, Berlin-New York, 241-247.

[B155] Kolibáč J Majer K Švihla V (2005) Cleroidea. Brouci nadčeledi Cleroidea Česka, Slovenska a sousedních oblastí. Beetles of the superfamily Cleroidea in the Czech and Slovak republics and neighbouring areas. Clarion, Praha, 186 pp.

[B156] Kolibáč J Schmied H Wappler T Kubisz D (2010) A description of *Promanodes* serafini gen. et sp. n. from Baltic amber, with a review of related New Zealand Promanus Sharp, 1877 (Coleoptera: Trogossitidae). Zootaxa 2620: 29-44.

[B157] Kolibáč J Zaitsev AA (2010) A description of a larva of Ancyrona diversa Pic, 1921 and its phylogenetic implications (Coleoptera: Trogossitidae). Zootaxa 2451: 53-62.

[B158] Kryzhanovskij OL (1965) 38. Fam. Ostomatidae (Trogositidae) – Schitovidki. In: Gurjeva EL Kryzhanovskij OL (Eds) The Insects of the European Part of USSR, Volume II: Beetles and Strepsiptera. Nauka, Moscow, Leningrad, 239-240. [in Russian]

[B159] Kukalová-Peck J Lawrence JF (1993) Evolution of the hind wing in Coleoptera. Canadian Entomologist 125: 181-258. doi: 10.4039/Ent125181-2

[B160] Kuschel G (1990) Beetles in a Suburban Environment: ANew Zealand AQ20 Case Study. The Identity and Status of Coleoptera in the Natural and Modified Habitats of Lynfield, Auckland (1974–1989). DSIRPlant Protection Report 3.

[B161] Lacordaire Z (1857) Fam. 41: Clérides. In: Histoire Naturelle des Insectes. – Genera des Coléoptères IV. Paris, 493 pp.

[B162] Lafer GSh (1992) Peltidae (Lophocateridae). In: Keys to the identification of insects of the Soviet Far East. 3 – Coleoptera, or beetles. Part 2. Nauka, Saint-Petersburg, 82–86.

[B163] Larsson SG (1978) Baltic Amber – a Palaeobiological Study. Entomonograph Vol. 1 Scandinavian Science Press, Ltd. Klampenborg, Denmark, 192 pp.

[B164] Latreille PA (1802) Histoire Naturelle, Générale et Particuliere des Crustacés et Insectes. Tom troisieme: 133. (x+467pp+1).

[B165] Latreille PA (1806) Genera crustaceorum et insectorum secundum ordinem naturalem in familias disposita, iconibus exemplisque plurimus explicata. Tomus primus Parisiis et Argentorati, Amand König, xvii+ 302pp, xvi pls.

[B166] Lawrence JF Newton AF Jr (1982) Evolution and classification of beetles. Annual Review of Ecology and Systematics 13: 261-290. doi: 10.1146/annurev.es.13.110182.001401

[B167] Lawrence JF Newton AF Jr (1995) Families and subfamilies of Coleoptera (with selected genera, notes, references and data on family-group names). In: Pakaluk J Ślipiński SA (Eds) Biology, Phylogeny, and Classification of Coleoptera. Papers Celebrating the 80th Birthday of Roy A. Crowson. Muzeum i Instytut Zoologii PAN, Warszawa, 779-1006.

[B168] Lawrence JF Britton EB (1994) Australian beetles. Melbourne University Press, 192 pp. doi: 10.1111/j.1440-6055.1980.tb00989.x

[B169] Lawrence JF (1980) A new genus of indo-australian Gempylodini with notes on the constitution of the Colydiidae (Coleoptera). Journal of Australian entomological Society 19: 293-310.

[B170] Lawrence JF (1982) Coleoptera. In: Synopsis and Classification of Living Organisms. McGraw-Hill Book Company, Inc., 482–553.

[B171] Lawrence JF (1991) Order Coleoptera. In: Stehr FW (Ed) Immature Insects. Vol. 2 Kendall/Hunt Publishing Company, Dubuque, Iowa, 144-237.

[B172] Lawrence JF Hastings A Dallwitz M Paine T (1993) Beetle larvae of the world. In: Lawrence JF Hastings A Dallwitz M Paine T (Eds) CSIRO Publications, CD-ROM, ISBN 0-643-05506.

[B173] Lawrence JF Hastings AM Dallwitz MJ Paine TA Zurcher EJ (1999a) Beetles of the World: A key and information system for families and subfamilies. CD-ROM, Version 1.0 for MS-Windows, CSIRO Publishing, Melbourne.

[B174] Lawrence JF Hastings AM Dallwitz MJ Paine TA Zurcher EJ (1999b) Beetle larvae of the world: Descriptions, illustrations, identification, and information retrieval for families and sub-families. CD-ROM, Version 1.1 for MS-Windows, CSIRO Publishing, Melbourne.

[B175] Lawrence JF Ślipiński A (2013) Globo *rentonium*, a new genus of rentoniine Trogossitidae (Coleoptera: Cleroidea) from Australia and Brazil. Zootaxa 3710(3): 257-270. doi: 10.11646/zootaxa.3710.3.4 26106688

[B176] Lawrence JF Ślipiński A Seago SE Thayer MK Newton AF Marvaldi AE (2011) Phylogeny of the Coleoptera based on morphological characters of adults and larvae. Annales Zoologici 61: 1-217. doi: 10.3161/000345411X576725

[B177] Lawson SA Morgan FD (1992) Rearing of two predators, *Thanasimus dubius* and *Temnochila virescens*, for the biological control of Ips grandicollis in Australia. Entomologia Experimentalis et Applicata 65(3): 225-233. doi: 10.1111/j.1570-7458.1992.tb00675.x

[B178] Lawson SA Morgan FD (1993) Prey specificity of adult *Temnochila virescens* F. (Col., Trogositidae), a predator of Ips grandicollis Eichh. (Col., Scolytidae). Journal of Applied Entomology 115(2): 139-144. doi: 10.1111/j.1439-0418.1993.tb00373.x

[B179] Lemdahl G (2001) Records of *Grynocharis oblonga* L. (Coleoptera) on pine (*Pinus sylvestris*). Fynd av avlang flatbagge *Grynocharis oblonga* L. (Coleoptera) pa tall. Entomologisk Tidskrift 122(1–2): 39-40.

[B180] Leng CW (1920) Catalogue of the Coleoptera of America, north of Mexico. Mount Vernon, N.Y. 470 pp.

[B181] Lepesme P Paulian R (1944) Les *Nemosoma* et genres voisins. Revue francaise d’Entomologie 10: 136–141.

[B182] Leschen RAB (2000) Beetles feeding on bugs (Coleoptera, Hemiptera): repeated shifts from mycophagous ancestors. Invertebrate Taxonomy 14: 917-929. doi: 10.1071/IT00025

[B183] Leschen RAB (2002) 72. Trogossitidae Latreille, 1802. In: Arnett RH Thomas MC Skelley PE Frank JH (Eds) American Beetles. Vol. 2. Polyphaga: Scarabaeoidea through Curculionoidea. CRCPress, Boka Raton-London-New York-Washington D.C., 263-266.

[B184] Leschen RAB Lackner T (2013) Gondwanan Gymnochilini (Coleoptera: Trogossitidae): generic concepts, review of New Zealand species and long-range Pacific dispersal. Systematic Entomology 38: 278-304. doi: 10.1111/j.1365-3113.2012.00661.x

[B185] Leschen RAB Lawrence JF Ślipiński AS Gimmel M (2012) Phylogenetic relationships of Cleroidea. XXIVth International Congress of Entomology, August 19–25, 2012, Daegu, Korea.

[B186] Léveillé A (1877) Description d’une nouvelle espèce de trogositidae. Bulletin de la Société entomologique de France 1877: cxi–cxii.

[B187] Léveillé A (1888) Catalogue de la famille des Temnochilides. Annales de la Société entomologique de France Sér. 6, 8: 29–448.

[B188] Léveillé A (1910) Temnochilidae. In: Schenkling S (Ed) Coleopterorum Catalogus 11, W. Junk, Berlin, 40 pp.

[B189] Lin Q-B (1985) Insect fossils from the Hanshan formation at Hanshan county, Anhui province. Acta entomologica sinica 24: 300–311, 2 pls.

[B190] Löbl I Smetana A (Eds) (2010) Catalogue of palaearctic Coleoptera. Vol. 6: Chrysomeloidea. 924 pp.

[B191] Lohse GA (1979) 30. Familie: Melyridae, 3. U.Fam. (Familie 30a): Phloeophilinae. In: Freude H Harde KW Lohse GA (Eds) Die Käfer Mitteleuropas. Band 6 Goecke, Evers, Krefeld, 83.

[B192] Lucht W (1981) Coleoptera Westfalica: Familia Trogositidae. Abhandlungen aus dem Landesmuseum für Naturkunde zu Muenster 43(3): 35-42.

[B193] Lucht W (1998) Supplements and amendments to the volumes 6, 7, 8 and 13 (Supplement 2). Family Trogossitidae, Peltidae, Lophocateridae, Lymexylidae. Erganzungen und Berichtigungen zu den Banden 6, 7, 8 und 13 (2. Supplement). Familie Trogositidae [Trogossitidae], Peltidae, Lophocateridae, Lymexylidae. In: Die Käfer Mitteleuropas. Bd. 15: 4 Supplementband. [The beetles of Central Europe. Vol. 15: 4. Supplement.] Goecke, Evers, Krefeld, 398 pp.

[B194] Luna de Carvalho E (1979) Guia pratico para a identificacao de alguns insectos de armazens e produtos armazenados. 2.a Parte. Junta de Investigacoes Cientifica do Ultramar, Lisbon, 73–191.

[B195] Machado A Oromí P (2000) Elenco de los Coleoptéros de las Islas Canarias. [Catalogue of the Coleoptera of the Canary Islands.] Instituto de estudios Canarios, La Laguna.

[B196] Majer K (1986) Komentovaný katalog československých druhů čeledi Phloiophilidae a Melyridae (excl. Malachiinae). (Coleoptera, Cleroidea). Zborník Slovenského národního Múzea, Prírodné Vedy 32: 113-129.

[B197] Majer K (1994) A review of the classification of the Melyridae and related families (Coleoptera, Cleroidea). Entomologica Basiliensia 17: 319-390.

[B198] Mamaev BM (1976) Review of larvae of the family Trogossitidae (Coleoptera) in the fauna of the USSR. Zoologicheskiy Zhurnal 55: 1648-1658.

[B199] Marsden MA Kline LN Furniss MM (1981) Modeling seasonal abundance of Douglas-fir beetle in relation to entomophagous insects and location in trees. US Forest Service General Technical Report INT 111: 1-22.

[B200] Martynov AV (1926) KPoznaniyu Iskopaemykh Nasekomykh Yurskikh Slantsev Turkestana. 5. O Nekotorykh Formakh Zhukov (Coleoptera). Ann. Soc. Paléont. Russie [Ezhegodnik Russkogo Paleontologicheskogo Obshchestva] 5: 1-39.

[B201] Massey CL Lucht DD Schmid JM (1977) Roundheaded pine beetle. US forest service forest insect and disease leaflet 155: 1-8.

[B202] Mateu J (1972) Les Insectes xylophages des Acacia dans les régions sahariennes. In: Publicacoes do Instituto de Zoologia “Dr.Augusto Nobre“, Imprensa Portuguesa, Porto, 714 pp.

[B203] Matthews EG (1992) A guide to the genera of beetles of South Australia. Part 6 Polyphaga: Lymexyloidea, Cleroidea and Cucujoidea. In: Thurmer J (Ed) Special Educational Bulletin Series (No 9), South Australian Museum, Adelaide, 75 pp.

[B204] Mayor A (2007) Phloiophilidae. In: Löbl I Smetana A (Eds) Catalogue of Palaearctic Coleoptera. Vol. 4 Apollo Books, Stenstrup, 363-364.

[B205] McCravy KW Nowak JT Douce GK Berisford CW (2000) Evaluation of multiple-funnel and slot traps for collection of southern pine bark beetles and predators. Journal of Entomological Science 35(1): 77-82.

[B206] McKenna DD Wild A Kanda K Maddison D Farrell BD (2012) Reconstructing the Beetle Tree of Life. XXIVth International Congress of Entomology, August 19–25, 2012, Daegu, Korea.

[B207] Medvedev LN (1969) Novye mezozoyskiye zhestkokrylye (Cucujoidea) Azii. Paleontologicheskiy Zhurnal 1969: 119-125.

[B208] Merkl O (1993) Beetles with various antennae. 6. Diversicornaria 6. Clavicornia 1. Magyarorszag Allatvilaga 169: 1-27.

[B209] Meunier F (1921) Die Insektenreste aus den Lutetien von Messel bei Darmstadt. Abhandlungen der Hessischen Geologischen Landesanstalt 7: 1-16.

[B210] Meunier F (1921) Die Insectenreste aus den Lutetien von Messel bei Darmstadt. Abhandlungen der Hessischen Geologischen Landesanstalt 7: 1-16.

[B211] Milkowski M Wojas T (2008) Two new localities of *Nemosoma* caucasicum Menetr. (Coleoptera: Trogossitidae) in Poland. Wiadomosci Entomologiczne 27(3): 172.

[B212] Miller DR Gibson KE Raffa KF Seybold SJ Teale SA Wood DL (1997) Geographic variation in response of pine engraver, Ips pini, and associated species to pheromone, lanierone. Journal of Chemical Ecology 23(8): 2013-2031. doi: 10.1023/B:JOEC.0000006486.39056.48

[B213] Mitter H (1983) Die Verbreitung der Familie Ostomidae in Oberösterreich (Coleoptera, Ostomidae). Nachrichtenblatt der Bayerischen Entomologen 32(2): 52-54.

[B214] Mitter H (1998) Notizen zur Biologie und Verbreitung der Ostomidae in Oberösterreich (Coleoptera, Ostomidae). Stapfia 55: 559-565.

[B215] Miyatake M (1977) A new genus of the subfamily Thaneroclerinae from Fiji (Coleoptera: Cleridae). Transactions of Shikoku Entomological Society 13: 105-107.

[B216] Miyatake M (1985) Trogossitidae. In: Kurosawa Y Hisamatsu S Sasaji H (Eds) The Coleoptera of Japan in Color, 3 Hoikusha, Osaka, 147–150, pl. 24 [in Japanese]

[B217] Moragues G (1981) Nouvelle capture de *Nemosoma* cornutum en France (Col. Ostomatidae). Entomologiste (Paris) 37(6): 262-263.

[B218] Mörs T (1995) Die Sedimentationsgeschichte der Fossillagerstätte Rott und ihre Alterseinstufung anhand neuer Säugetierfunde (Oberoligozän, Rheinland). Courier Forschungsinstitut Senckenberg 187: 1-129.

[B219] Müller OF (1764) Hafniae et Lipsiae, F. Gleditschi. Fauna insectorum Fridrichsdalina, sive methodica descriptio insectorum agri Fridrichsdalensis cum characteribus genericis et specificis, nominibus trivialibus, locis natulibus, iconibus allegatis, novisque pluribus speciebus additis, xxiv+ 96pp.

[B220] Nakane T (1963) Cleroidea. Trogossitidae. Fragmenta Coleopterologica 12: 46.

[B221] Nakane T (1985) New or little-known Coleoptera from Japan and its adjacent regions. XXXVIII. Fragmenta Coleopterologica 38–40: 153–164.

[B222] Nakane T Ohbayashi K Nomura S Kurosawa Y (1963) Cleroidea. In: Iconographia Insectorum Japonicorum Colore naturali edita. Vol. II (Coleoptera). Hokuryukan, Tokyo, Japan, 181–186.

[B223] Nikitsky NB (1974) Morphology of larvae and mode of life of *Nemosoma* (Coleoptera, Trogossitidae), predator of bark beetles in the Nort-west Caucasus. Zoologicheskiy Zhurnal 53: 563-566.

[B224] Nikitsky NB (1980) Insect predators of bark beetles and their ecology. Nauka, Moscow, 237 pp.

[B225] Nikitsky NB (1992) Trogositidae. In: Ler PA (Ed) Opredelitel nasekomykh Dalnego Vostoka SSSR. Tom III. Zhestkokrylye, ili zhuki. [Keys to the identification of insects of the Soviet Far East. 3 – Coleoptera, or beetles. Part 2.] Nauka, Saint-Petersburg, 79-81.

[B226] Nikitsky NB Semenov VB Dolgin MM (1998) The beetles of the Prioksko-Terrasny Biosphere Reserve – xylobiontes, mycetobiontes, and Scarabaeidae (with the review of the Moscow Region fauna of these groups). Supplement 1. (With remarks on nomenclature and systematics of some Melandryidae of the World fauna). Sbornik Trudov Zoologicheskogo Muzeya MGU 36: 1-60.

[B227] Nikitsky NB Semenov VB (2001) To the knowledge of the beetles (Coleoptera) of the Moscow region. Byulleten’ Moskovskogo Obshchestva Ispytatelei Prirody Otdel Biologicheskii 106(4): 38-49.

[B228] Nilsson SG (1997) *Grynocharis oblonga* L. (Coleoptera: Trogositidae) – a specialized wood beetle with a relict distribution. Entomologisk Tidskrift 118(1): 1-9.

[B229] Noreika N (2009) New records of rare species of Coleoptera found in Ukmerge district in 2004–2005. New and Rare for Lithuania Insect Species Records and Descriptions 21: 68-71.

[B230] Olliff AS (1883a) Descriptions of new species of Clavicorn Coleoptera. Cistula Entomologica 3: 58-59.

[B231] Olliff AS (1883b) Descriptions of two larvae and new genera and species of Clavicorn Coleoptera, and a synopsis of the genus *Helota*, MacLeay. Cistula entomologica (Pars xxvii): p. 57 + Pl. 11, Fig. 6.

[B232] Olliff AS (1883c) Remarks on a small collection of Clavicorn Coleoptera from Borneo, with descriptions of new species. Transactions of the Entomological Society of London 1883: 173-186.

[B233] Page JM (1981) Drought-accelerated parasitism of conifers in the mountain ranges of northern California. Environmental Conservation 8(3): 217-226. doi: 10.1017/S0376892900027661

[B234] Pajares JA Ibeas F Díez JJ Gallego D (2004) Attractive responses by *Monochamus galloprovincialis* (Col., Cerambycidae) to host and bark beetle semiochemicals. Journal of Applied Entomology 128(9–10): 633-638.

[B235] Pankow W (2010) *Nemozoma caucasicum* Menetries, 1832 (Coleoptera, Trogositidae) new in Germany. Mitteilungen Entomologischer Verein Stuttgart 45(2): 87.

[B236] Pascoe FP (1863) Notices of new or little-known genera and species of Coleoptera. Part IV The Journal of Entomology 2: 26–56, pls II, III.

[B237] Pascoe FP (1868) Contributions to a knowledge of the Coleoptera. Part I (a paper read February 17, 1968, by Mr. F. P. Pascoe, with brief diagnoses of some of the most interesting of the new genera and species). The Proceedings of the Entomological Society of London 1868: xi–xiii.

[B238] Pascoe FP (1872) Notes on Coleoptera, with descriptions of new genera and species. Part II. Annals and magazine of natural history 4: 317–326, pl. xv. doi: 10.1080/00222937208696709

[B239] Paulian R (1998) Les insectes de Tahiti. [The insects of Tahiti.] Editions Boubee, Paris, 1–332.

[B240] Peris D Kolibáč J Delclòs X (in press) *Cretamerus vulloi* gen. et sp. n., the oldest bark-gnawing beetle (Coleoptera: Trogossitidae) from the Cretaceous amber. Journal of Systematic Palaeontology. [accepted Sept. 9, 2013]

[B241] Peyerimhoff PM de (1918) Nouveaux Coléopteres du Nord-Africain (Vingt-sixieme note): Faune du Cedre et du Sapin de Numidie Bulletin de la Société entomologique de France (Séance du 28 novembre 1917) 1917: 329–330.

[B242] Pic M (1921) Notes diverses, descriptions et diagnoses. L‘Échange, Revue Linnéenne 37: 1-4.

[B243] Pic M (1924) Coleoptera-Clavicornia et autres de Juan Fernandez. The Natural History of Juan Fernandez and Easter Island 3: 377-380.

[B244] Pic M (1926) Phloeophilidae, Rhadalidae, Prionoceridae. In: Jung W (Ed) Coleopterorum Catalogus 87, Berlin.

[B245] Pileckis S Monsevicius V (1995) Keršvabaliniai (Cleroidea). In: Lietuvos Fauna. Vabalai 1. Mokslo ir enciklopediju leidykla, Vilnius, 271–281.

[B246] Piller M Mitterpacher L (1783) Iter per Posegnam Sclavoniae provinciam mensibus Junio, et Julio Anno MDCCXXXII suspectum. Budae, Typis regiae universitatis, 147 pp + 16pls.

[B247] Plata-Negrache P Prendes-Ayala C (1981) Los Ostomidae (Col.) del Archipiélago Canario. Boletín de la Asociación Española de Entomología 4: 225-234.

[B248] Ponomarenko AG Kireichuk AG (2004–2008) Taxonomic list of fossil beetles of the suborder Scarabaeina (part 2 of catalogue). http://www.zin.ru/Animalia/Coleoptera/eng/paleosy2.htm

[B249] Ponomarenko AG (1985) Yurskiye nasekomye Sibiri i Mongolii. [Coleoptera from the Jurassic of Siberia and Western Mongolia.] Trudy Paleontologicheskogo Instituta 211: 47-87.

[B250] Ponomarenko AG (1986) [Insects in the Early Cretaceous ecosystems of the West Mongolia. Descriptions of fossils Scarabaeida (= Coleoptera).] Sovmestnaya Sov-Mongolskaya Paleontoleontologicheskaya Ekspediciya, 101 pp.

[B251] Ponomarenko AG (1990) Nadsemeystvo Cleroidea Latreille, 1802. In: Raznitsyn AP (Ed) Pozdnemezozoyskiye nasekomye Vostochnogo Zabaykaliya.Nauka Press, Moscow, 72-74.

[B252] Pospischil R (2003) The bread beetle *Tenebroides mauretanicus*. Der Schwarze Getreidenager *Tenebroides mauretanicus*. Praktische Schaedlingsbekaempfer 55(7–8): 4-5.

[B253] Purrini K Ormieres R (1979) Über eine neue Neogregarine, *Farinocytis *tenebrioides** n. sp. des Getreidenagers. *Tenebrioides mauretanicus* L. (Coleoptera, Ostomidae). Zoologischer Anzeiger 202(5–6): 437-443.

[B254] Ratti E (1997) Catalogo dei Coleotteri della Laguna di Venezia. VIII – Trogossitidae, Cleridae, Lymexylidae. Bollettino del Museo civico di Storia Naturale di Venezia 47: 177-185.

[B255] Reeve JD Strom BI Rieske LK Ayres BD Costa A (2009) Geographic variation in prey preference in bark beetle predators. Ecological Entomology 34(2): 183-192. doi: 10.1111/j.1365-2311.2008.01055.x

[B256] Reitter E (1875) Revision der Gattung Trogosita Oliv. (Temnochila Westw.). Verhandlungen des naturforschenden Vereines in Brünn 13: 3-44.

[B257] Reitter E (1875) Süd- und Mittel-Amerikanischen Arten der Gattung *Tenebrioides* Pill. et Mitterp. (Trogosita Strm., Er., Redt., Thoms., Horn). Verhandlungen des naturforschenden Vereines in Brünn 13: 65-79.

[B258] Reitter E (1876) Systematische Eintheilung der Trogositidae. (Familia Coleopterorum). Verhandlungen des naturforschenden Vereines in Brünn 14: 3-66.

[B259] Reitter E (1877) Beiträge zur Kenntniss aussereuropäischer Coleopteren. Mittheilungen des Münchener Entomologischen Vereins 1877: 126-140.

[B260] Reitter E (1880) Neun neue Clavicornien (Coleoptera). Verhandlungen des naturforschenden Vereines in Brünn 18: 1-6.

[B261] Reitter E (1890) Coleopterologische Notizen. XXXIX (November 1890). Wiener Entomologische Zeitung 9(30): 264-267.

[B262] Reitter E (1911) Fauna Germanica. Die Käfer des Deutschen Reiches. III. Band KG Lutz Verlag, Stuttgart, 436 pp. + 48 tabs.

[B263] Reitter E (1922) Bestimmungs-Tabellen der europäischen Coleopteren. VI. Heft. Enthaltend die Familien Colydiidae, Rhysodidae, Trogositidae. Zweite, ganzlich umgearbeitete und auf die palaearktische Fauna ausgedehnte Auflage. E. Reitter, Troppau, 73 pp.

[B264] Ren D (1995) Fossil Insects. In: Ren D Lu L-W Guo J-G Ji S-A (Eds) Fauna and Stratigraphy of Jurassic-Cretaceous in Beijing and the adjacent areas.Seizmic Publishing House, Beijing, 47-121.

[B265] Ross DW Daterman GE (1998) Pheromone-baited traps for Dendroctonus pseudotsugae (Coleoptera: Scolytidae): influence of selected release rates and trap designs. Journal of Economic Entomology 91(2): 500-506.

[B266] Šablevičius B Ferenca R (1995) 14 new and 3 rare for Lithuania species of Coleoptera found in 1987–1994. Institute of Ecology, Lithuanian Entomological Society, Vilnius 1995, 184pp.

[B267] Sakamoto JM (2007) Notes on the occurrence of *Nemosoma* attenuatum Van Dyke, 1915 (Coleoptera: Trogossitidae), in California with a literature review and museum survey of *Nemosoma* spp. Pan-Pacific Entomologist 83(4): 342-351. doi: 10.3956/2007-19.1

[B268] Sarikaya O Avci M (2009) Predators of Scolytinae (Coleoptera: Curculionidae) species of the coniferous forests in the Western Mediterranean Region, Turkey. Turkiye Entomoloji Dergisi 33(4): 253-264.

[B269] Schaeffer CF A (1915) Change of generic names. Journal of the New York entomological Society 23: 68-69.

[B270] Schaeffer CF A (1918) On some genera and species of the family Ostomidae. Journal of the New York entomological Society 26: 190-201.

[B271] Schaeffer CF A (1920) Ostomidae. In: Leng CW (Ed) Catalogue of the Coleoptera of America, north of Mexico. Mont Vernon, N.Y., 193-194.

[B272] Schawaller W (1993) Taxonomie und Larvalmorphologie palaarktischer Leperina (Coleoptera: Trogossitidae). Stuttgarter Beiträge zur Naturkunde, Serie A (Biologie) 500: 1-9.

[B273] Schmied H Wappler T Kolibáč J (2009) A new bark-gnawing beetle (Coleoptera, Trogossitidae) from the middle Eocene of Europe, with a checklist of fossil Trogossitidae. Zootaxa 1993: 17-26.

[B274] Schmied H Wappler T Kolibáč J Kubisz D (2009) New Zealand beetle in Baltic Amber – Biogeography and Systematics of a new fossil bark-gnawing beetle (Trogossitidae). Terra Nostra, Schriften der GeoUnion Alfred Wegener-Stiftung 3: 105-106.

[B275] Šefrová H Laštůvka Z (2005) Catalogue of alien animal species in the Czech Republic. Acta Universitatis Agriculturae et Silviculturae Mendelianae Brunensis 53: 151–170.

[B276] Sharp D (1877) Descriptions of some new species and indications of new genera of Coleoptera from New Zealand. Entomologists Monthly Magazine 13: 265-272.

[B277] Sharp D (1891) Fam. Nitidulidae, Trogositidae, Synteliidae. In: Godman FD Salvin O (Eds) Biologia Centrali-Americana. Zoologia. Insecta. Coleoptera. Vol. II, Part 1 Dulau and Co., London, xii+717+1 pp, 19 pls.

[B278] Skatulla U Feicht E (1992) Untersuchungen zum Anflugverhalten des Kupferstechers (*Pityogenes chalcographus* L.) und einiger Beifange an Pheromonfallen mit Hilfe eines neuartigen elektronischen Messgerates. Anzeiger fuer Schaedlingskunde Pflanzenschutz Umweltschutz 65(1): 4-7. doi: 10.1007/BF01905974

[B279] Ślipiński SA (1992) Larinotinae – A new subfamily of Trogossitidae (Coleoptera), with notes on the constitution of Trogossitidae and related families of Cleroidea. Revue suisse de Zoologie 99: 439-463.

[B280] Solier AJ J (1849) Orden III. Coleoptera. In: Gay C Historia fisica y politica de Chile segun documentos adquiridos en esta republica durante doce anos de residencia en ella y publicada bajo los auspicios del supremo gobierno. Tomo quarto. Zoologia. Museo de Historia Natural de Santiago, Paris-Santiago, 105–511.

[B281] Spahr U (1981) Systematischer Katalog der Bernstein- und Kopal-Käfer (Coleoptera). Stuttgarter Beiträge für Naturkunde (Ser. B) 80: 1-107.

[B282] Spinola M (1844) Essai monographique sur les Clérites Coléoptéres. Tomes 1, 2 Impr. de fréres Ponthenier, Genes, 386 pp.

[B283] Stephens JF (1830) Illustrations of British entomology or, a synopsis of indigenous insects: containing their generic and specific distinctions with an account of their metamorphoses, times of appearance, localities, food, and economy, as far as practicable. Volume III, Mandibulata Baldwin and Cradock, London, 447 pp + pls xvi–xix.

[B284] Stresemann E Hannemann H Klausnitzer B Senglaub K (1989) Exkursionsfauna fur die Gebiete der DDR und der BRD. Band 2/1. Wirbellose: Insekten – Erster Teil. 8 Auflage Volk und Wissen Volkseigener Verlag, Berlin, 504 pp.

[B285] Struble GR Carpelan LH (1941) External sex characters of two important native predators of the mountain pine beetle in sugar pine. Pan-Pacific Entomologist 17: 153-156.

[B286] Svitra G Aliukonis A (2009) Data on five protected species of beetles (Coleoptera) recorded in Lithuania In 2007–2009. New and Rare for Lithuania Insect Species Records and Descriptions 21: 72-75.

[B287] Swezey SL Dahlsen DL (1982) Effects of insecticides on selected natural enemies of the western pine beetle (*Dendroctonus brevicomis*). Entomological Society of America Pacific Branch Annual Meeting 65: 142.

[B288] Szafraniec S (1997) Beetles (Coleoptera) new to the Babia Gora Mountain. Wiadomosci Entomologiczne 15(4): 207-215.

[B289] Szwalko P Kubisz D (1994) New records of *Tenebroides fuscus* (Goeze) (Coleoptera: Trogossitidae) in Poland. Acta Entomologica Silesiana 2(2): 46.

[B290] Tait SM Dahlsten DL Gill RJ Doyen JT (1990) Life history of the Incense Cedar Scale, Xylococculus macrocarpae (Homoptera: Margarodidae), on Incense Cedar in California with a description of the larvae of one of its common predators, Eronyxa expansus Van Dyke (Coleoptera: Trogositidae). Hilgardia 58: 1-19.

[B291] Teson A Dagoberto EL (1979) ‘Asociacion’ de insectos y acaros con Megachile gomphrenae y *M. pallefacta* (Hymenoptera) de la provincia de Buenos Aires. Revista de la Sociedad Entomologica Argentina 38(1–4): 127-131.

[B292] Theunert R (2006) A new record of *Thymalus limbatus* F., 1787 in Westphalia after over 125 years (Col., Peltidae). Mitteilungen der Arbeitsgemeinschaft Westfaelischer Entomologen 22(3): 113-114.

[B293] Thomson CG (1859) Skandinaviens Coleoptera, synoptiskt bearbetade. I. Tom, Tryckt uti Berlingska Boktryckeriet, Lund, 290 pp.

[B294] Thomson CG (1862) Skandinaviens Coleoptera, synoptiskt Bearbetade. Tom IV Lundbergska Boktryckeriet, Lund, 269 pp.

[B295] Tsinkevich VA (1997) Review of family Trogossitidae (Coleoptera) the fauna of Belarus. Vestnik Belorusskogo Gosudarstvennogo Universiteta Seriya 2 Khimiya Biologiya Geografiya 1: 27–29, 76.

[B296] Uchida A (1980) Studies on the life cycle of cestode parasitic in the small intestine of Japanese quail. Bulletin of Azabu University Veterinary Medicine 1(1): 61-73.

[B297] Valcarcel JP Prieto Pilona F (2001) New records of Coleoptera for Galicia (N.W. Iberian Peninsula). Boletin de la SEA 28: 109-110.

[B298] Van Dyke EC (1915) Some new beetles in the families Ostomidae (Trogositidae) and Cleridae from California. Bulletin of the Brooklyn entomological Society 10: 25–33.

[B299] Van Dyke EC (1916) Supplementary notes and descriptions of North American Ostomidae, Cleridae, and Cossonus (Col.) Bulletin of the Brooklyn entomological Society 11(4): 71-79.

[B300] Van Dyke EC (1920) New name for *Nemosoma* punctulata. Bulletin of the Brooklyn entomological Society 15: 85.

[B301] Van Dyke EC (1944) New species of North American Ostomidae (Coleoptera). Pan-Pacific Entomologist 20: 147-153.

[B302] Villemant C Ramzi H (1997) Predators of *Lymantria dispar* (Lep. Lymantriidae) egg masses: spatio-temporal variation of their impact during the 1988–89 pest generation in the mamora cork oak forest (Morocco). Entomophaga 40(3–4): 441-456. doi: 10.1007/BF02373731

[B303] Vogt H (1967) 48. Familie: Ostomidae. In: Die Käfer Mitteleuropas. Band 7. Goecke, Evers, Krefeld, 14–18.

[B304] Waltz RD (2002) Reports of rarely collected or previously unreported Indiana Coleoptera (Insecta): Cerambycidae, Cerophytidae, Hydraenidae, Hydrophilidae, Nitidulidae, Scarabaeidae, Staphylinidae, Trogossitidae. Proceedings of the Indiana Academy of Science 111(2): 177-181.

[B305] Weidner H (1993) Bestimmungstabellen der Vorratsschadlinge und des Hausungeziefers Mitteleuropas. 5., überarbeitete und erweiterte Auflage. [Identification tables of stored product and household pests of Central Europe. 5th revised edition.] Stuttgart, Jena, New York, i–xi, 1–328.

[B306] Westcott RL Kelly S Fullerton S (2007) A new prey record for *Temnochila virescens* (Fabricius): Actenodes acornis (Say) (Coleoptera: Trogositidae, Buprestidae). Coleopterists Bulletin 61(4): 573. doi: 10.1649/0010-065X(2007)61[573:ANPRFT]2.0.CO;2

[B307] Westwood JO (1830) On the affinities of the genus Clinidium of Kirby. Zoological Journal 5: 213-237.

[B308] Westwood JO (1864) Descriptions of new species of Coleoptera. Proceedings of the Entomological Society of London (1864): 11–12.

[B309] Whitehouse NJ (1997) Insect faunas associated with *Pinus sylvestris* L. from the Mid-Holocene of the Humberhead Levels, Yorkshire, U.K. Quaternary Proceedings 5: 293-303.

[B310] Wickham HF (1910) New fossil Coleoptera from Florissant, with notes on some already described. American Journal of Science 24: 47-51. doi: 10.2475/ajs.s4-29.169.47

[B311] Wickham HF (1913) Fossil Coleoptera from the Wilson ranch near Florissant, Colorado. Bulletin of the State University of Iowa 57: 3–29 +7pl.

[B312] Wickham HF (1916) A new brachyelytrous trogositid beetle from Colorado. Psyche 23: 146-148. doi: 10.1155/1916/35697

[B313] Wielink P Felix R Spijkers H Teunissen AP JA (2010) *Phloiophilus edwardsi*i in ‘De Kaaistoep’ near Tilburg (Coleoptera: Phloiophilidae). Entomologische Berichten 70: 17-20.

[B314] Wigger H (1993) Okologische Bewertung von Rauber-Beifangen in Borkenkäfer-Lockstoffallen. Anzeiger für Schaedlingskunde Pflanzenschutz Umweltschutz 66(4): 68-72. doi: 10.1007/BF01903073

[B315] Wigger H (1994) Die Reaktion der Frasskapazitat des Borkenkäferraubers *Nemosoma elongatum* L. (Col., Ostomidae) im Imaginalstadium auf unterschiedliches Beuteangebot in kunstlichen Gangen. Anzeiger für Schaedlingskunde Pflanzenschutz Umweltschutz 67(1): 8-13. doi: 10.1007/BF01906562

[B316] Wigger H (1996) Population-dynamics and predator-prey-interaction between the predator *Nemosoma elongatum* and the bark beetle species *Pityogenes chalcographus* (Coleoptera: Ostomidae, Scolytidae). Populationsdynamik und Rauber-Beute-Beziehung zwischen dem Borkenkäfer-Rauber *Nemosoma elongatum* und dem Kupferstecher *Pityogenes chalcographus* (Coleoptera: Ostomidae, Scolytidae). Entomologia Generalis 21(1–2): 55-67.

[B317] Williams KK McMillin JD DeGomez TE (2009) 11. Relative and seasonal abundance of three bark beetle predators (Coleoptera: Trogositidae, Cleridae) across an elevation gradient in ponderosa pine forests of north central Arizona. Western North American Naturalist 69(3): 351-363. doi: 10.3398/064.069.0309

[B318] Winkler A (1927) (1925–1932) Catalogus Coleopterorum Regionis Palaearcticae. Albert Winkler, Wien, 880 pp.

[B319] Wollaston TV (1862) On the Euphorbia-infesting Coleoptera of the Canary Islands. Transactions of the Entomological Society of London, 3rd series 1: 136–164.

[B320] Zhang J-F (1992) Fossil Coleoptera from Laiyang, Shandong Province, China. Acta entomologica sinica 35: 331-338.

[B321] Yoshitomi H Lee C-F (in press) A revision of the genus *Kolibacia* Leschen and Lackner (Coleoptera, Trogossitidae, Trogossitinae). Entomological Science.

[B322] Yu Y Leschen RA B Ślipiński A Ren D Pang H (2012) The first fossil bark-gnawing beetle from the Middle Jurassic of Inner Mongolia, China (Coleoptera: Trogossitidae). Annales Zoologici (Warszawa) 62: 245-252. doi: 10.3161/000345412X652765

[B323] Zherichin VV (1978) Razvitie i smena melovych i kainozoiskich faunisticeskich kompleksov (tracheinye i chelicerovye). Trudy paleontologicheskogo instituta, Nauka, Moskva, 165: 200 pp.

[B324] Zhou J-L Darrell R Niwa W Christine G (2001) Kairomonal response of *Thanasimus undatulus*, *Enoclerus sphegeus* (Coleoptera: Cleridae), and *Temnochila chlorodia* (Coleoptera: Trogositidae) to bark beetle semiochemicals in eastern Oregon. Environmental Entomology 30(6): 993-998. doi: 10.1603/0046-225X-30.6.993

